# Wavelength‐Gated Photochemical Synthesis of Phenalene Diimides

**DOI:** 10.1002/anie.202016632

**Published:** 2021-03-18

**Authors:** Florian Feist, Sarah L. Walden, Jessica Alves, Susanna V. Kunz, Aaron S. Micallef, Aidan J. Brock, John C. McMurtrie, Tanja Weil, James P. Blinco, Christopher Barner‐Kowollik

**Affiliations:** ^1^ Centre for Materials Science Queensland University of Technology (QUT) 2 George St. Brisbane QLD 4000 Australia; ^2^ School of Chemistry and Physics Queensland University of Technology (QUT) 2 George St. Brisbane QLD 4000 Australia; ^3^ Max Planck Institute for Polymer Research (MPIP) Ackermannweg 10 55128 Mainz Germany; ^4^ Macromolecular Architectures Institute for Chemical Technology and Polymer Chemistry Karlsruhe Institute of Technology (KIT) Engesserstrasse 18 76131 Karlsruhe Germany; ^5^ Institute of Nanotechnology Hermann-von-Helmholtz-Platz 1 76344 Eggenstein-Leopoldshafen Germany

**Keywords:** Diels–Alder reaction, *ortho*-quinodimethane, persistent neutral radicals, phenalene diimide, wavelength-gated synthesis

## Abstract

Herein, we pioneer a wavelength‐gated synthesis route to phenalene diimides. Consecutive Diels–Alder reactions of methylisophthalaldehydes and maleimides afford hexahydro‐phenalene‐1,6‐diol diimides via 5‐formyl‐hexahydro‐benzo[f]isoindoles as the intermediate. Both photoreactions are efficient (82–99 % yield) and exhibit excellent diastereoselectivity (62–98 % d.r.). The wavelength‐gated nature of the stepwise reaction enables the modular construction of phenalene diimide scaffolds by choice of substrate and wavelength. Importantly, this synthetic methodology opens a facile avenue to a new class of persistent phenalenyl diimide neutral radicals, constituting a versatile route to spin‐active molecules.

## Graphical abstract

Methylisophthalaldehydes form two highly reactive *ortho*‐quinodimethane dienophiles in a step‐wise and wavelength gated fashion. Trapping with maleimides proceeds with high stereoselectivity under the formation of phenalene diimide scaffolds. Subsequent elimination and oxidation affords a new class of persistent phenalenyl diimide neutral radicals.

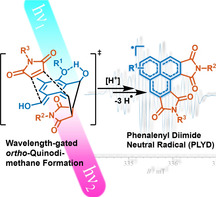

## Introduction

Polycyclic aromatic (di)‐imides are a ubiquitous structural motif owing to the combination of an electron‐rich aromatic system and a strong electron‐withdrawing moiety.^[1]^ These properties facilitate charge transportation and have thus been exploited for a wide array of applications, such as organic light‐emitting diodes,^[2]^ organic field‐effect transistors,^[3]^ and organic photovoltaic devices.^[4]^

Another important polycyclic aromatic hydrocarbon is the phenalene motif. Phenalenyl structures, in which three aryl rings share three C−C bonds, contain an uneven number of carbon atoms and, in the neutral state, possess an uneven number of electrons rendering them neutral radicals. Phenalenes exhibit unique electronic properties and the remarkable ability to form stable anions, cations, and radicals through the redox chemistry of their non‐bonding molecular orbital (NBMO).^[5]^ The rigid and planar phenalenyl unit represents a delocalized neutral radical with the highest spin density in the α‐position—analogous to a basic open‐shell graphene fragment.^[5, 6]^ For these reasons, the phenalenyl system is an important structural motif in organic spin chemistry^[6a]^ with applications in organocatalysis,^[6b]^ molecular conductors,^[7]^ photovoltaics,^[8]^ and magnetic materials^[9]^ and has been identified for use in synthetic quantum information devices.^[10]^ Organic molecules hosting persistent free radicals possess inherent spin‐related properties that can be utilized for spin‐active electronic devices.^[11]^ Common synthetic routes for phenalene derivatives proceed via phenalenones or 2,3‐dihydrophenalenones as intermediates.^[5]^ To our knowledge, no phenalene decorated with diimide moieties has been reported.

In general, free radicals are reactive and therefore are difficult to generate, handle, and isolate. To be suitable for device applications, neutral radicals are required to be sufficiently stable to be processed and stored under ambient conditions. Primarily, two modifications can be exploited to improve the persistence of neutral free radicals in polycyclic aromatic systems, such as phenalenyls. First, electronic stabilization, by the introduction of heteroatoms, or second, kinetic stabilization, via spin delocalization and/or steric hindrance. Given most delocalized neutral radicals require multi‐step syntheses and tedious workup procedures, simple tuning of the properties is not always feasible.

**Scheme 1:**
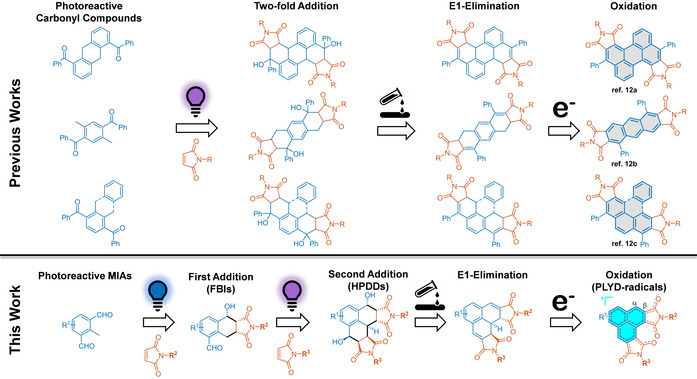
Top: Previously reported synthesis routes for polycyclic aromatic diimides via photoinduced Diels–Alder reaction of *o*‐QDMs and maleimides and subsequent elimination and oxidation reactions.^[12]^ It is important to note that diimides with two differing R groups could not be prepared by this method. Bottom: Herein presented wavelength‐gated, step wise synthesis of phenalenyl diimide scaffolds. The first addition of one equivalent of maleimide to photoreactive 2‐methylisophthalaldehydes (MIAs) at higher wavelength leads to the formation of 5‐formyl‐hexahydro‐benzo[*f*]isoindoles (FBIs). Subsequent addition of a second equivalent of maleimide at lower wavelength affords hexahydro‐phenalene‐1,6‐diol diimides (HPDDs). Acid‐catalyzed E1 elimination and subsequent 3‐electron oxidation leads to phenalenyl diimide neutral radicals (PLYD). The annulated ring systems marked grey cannot host a neutral radical. In contrast, the radical hosting phenalene ring system is highlighted turquoise.

The synthesis of polycyclic aromatic diimides via Diels–Alder reaction of maleimides and light‐generated *ortho*‐quinodimethanes *(o*‐QDMs) was established by Meador et al.,^[12]^ involving multi‐step synthesis of the precursors (5 to 7 steps), non‐stoichiometric reagent ratios, harsh UV light, and limited reaction efficiency (49–81 % yield). Despite the challenges, anthracene diimides, pyrene diimides, benzo[*e*]pyrene diimides, and perylene diimides were obtained (Scheme 1) and these reactions were later successfully applied for the synthesis of polyimides.^[13]^ In these pioneering studies, the authors hypothesized the potential for consecutive formation of two equivalents of *o*‐QDM, however successful step‐wise trapping of the *o*‐QDMs with different maleimides was not reported. Unprecedented ability to tune the diimide properties could be achieved with the incorporation of two disparate diimide functionalities, however previous synthetic routes to heterobifunctional polycyclic aromatic diimides required complex, multi‐step syntheses.^[1]^

The click‐chemistry concept introduced by Kolb, Finn, and Sharpless focusses on combining molecular building blocks via highly efficient ligation reactions.^[14]^ The modular nature of this approach has been demonstrated in many studies, especially in the fields of bio‐^[15]^ and polymer chemistry.^[16]^ The click concept can be further enhanced by exploiting the spatio‐temporal control of photochemical reactions.^[17]^ Already an indispensable tool in organic synthesis,^[18]^ photochemistry provides additional spatial and temporal control over chemical transformations, enabling the fabrication of nano‐structured materials.^[19]^ By exploiting the energy contained in different wavelengths of light, distinct reaction pathways can be accessed, affording the ability to perform complex synthetic steps independently, which would be difficult using conventional reaction conditions.^[20]^

We herein utilize the light‐induced formation of highly reactive dienes (*o*‐QDMs) from dialdehydes and subsequent Diels–Alder reaction with maleimide dienophiles to generate phenalene diimide scaffolds. The activation of the two carbonyl groups of a single chromophore in a consecutive, wavelength‐gated fashion represents the first example of such a ligation system and allows for the modular assembly of complex polycyclic molecules. The steric bulk of the imide groups allows for the generation of persistent phenalenyl diimide neutral radicals. Our concise approach eliminates the barrier of complex synthetic procedures in organic spin chemistry^[6a]^ via the judicious design and tailoring of electronic spin structures.

## Results and Discussion

Our synthetic strategy for phenalenyl diimide neutral radicals (PLYD) entails the generation of a fused ring system via the stepwise, light‐induced Diels–Alder reaction of methylisophthalaldehydes (MIA) and maleimides, followed by an acid‐catalyzed E1 elimination and oxidation (Scheme 1). There is currently no reported route to phenalene derivatives via Diels–Alder reactions or hexahydro‐phenalene‐1,6‐diols. In addition, the generation of fused ring systems involving *o*‐QDMs in the formation of the bridgehead atom is rarely reported in the literature, however a few examples with modest yields do exist.^[21]^

An efficient route to generate *o*‐QDMs is the photoinduced intramolecular hydrogen abstraction of a benzylic proton by an *ortho*‐formyl or benzoyl group.^[18]^ The photoinduced enolization of *o*‐methyl benzaldehydes (*o*‐MBAs) with a hydrogen bridge donor adjacent to the formyl group is known to allow for highly selective ligation reactions, even enabling the challenging synthesis of sequence‐defined macromolecules.^[22]^ Herein, we combine these design principles in a single photoreactive 2‐methylisophthalaldehyde entity. We demonstrate the wavelength‐gated formation of *o*‐QDMs involving a single methyl group and trapping these highly reactive intermediates with maleimide.

**Figure 1:**
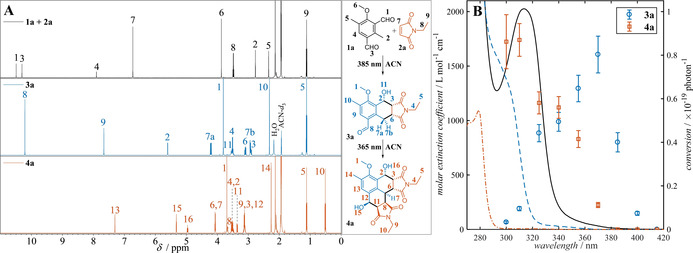
A) ^1^H NMR spectra and assigned resonances of the reaction between MIA **1 a** and maleimide **2 a** affording cycloadduct **3 a** under irradiation with a 385 nm LED and subsequently to form cycloadduct **4 a** under irradiation with a 365 nm LED (LED‐emission spectra Supporting Information Figure S1). The NMR spectra were recorded after removing the solvent from the respective batch photoreactions without further purification (excess *N*‐ethylmaleimide is removed in vacuo). For detailed characterization data (^13^C, 2D‐NMR spectra, and LC–HRMS) of all compounds as well as experimental details refer to the Supporting Information Figures S20, S21, S27, S28, S36–S40, S90, S96 and Supporting Information Section VI. B) Molar extinction coefficients of **1 a** (solid black), **3 a** (dashed blue) and **4 a** (dash–dot brown) overlaid with the wavelength‐dependent reactivity of the substrate **1 a** (1.6 mmol L^−1^ of **1 a** with 2.20 equiv of **2 a** in CD_3_CN) after irradiation with (9.4±0.2)×10^18^ photons of each wavelength from a 20 Hz, 7 ns tunable OPO laser. The conversions are determined by ^1^H NMR spectroscopy (Supporting Information Figures S87 and S88).

Initially, the non‐symmetric 2,5‐dimethylisophthalaldehyde **1 a** was synthesized in two steps (55 % overall yield, refer to Supporting Information Section II) via a Duff reaction and served herein as a model substrate for the photoreactions displayed in Figure 1. MIA **1 a** was irradiated in the presence of *N*‐ethyl maleimide **2 a** (2.20 equiv) with a 385 nm LED. 5‐Formyl‐hexahydro‐benzo[*f*]isoindole (FBI) **3 a** was formed as the major product (Figure 1 A). Subsequent irradiation with a 365 nm LED led to the formation of hexahydro‐phenalene‐1,6‐diol diimide **4 a**, unexpectedly resulting in a blue fluorescence of the reaction mixture under UV irradiation (**4 a**, *Φ*_F_=15±2 %, *λ*_F_,_max_=450 nm). 365 nm LED irradiation of a mixture of **1 a** and **2 a** (2.20 equiv) affords **4 a** directly. Further experiments utilizing a tunable laser as monochromatic light source, revealed the wavelength‐gated nature of the photoreaction (for details refer to Supporting Information Section IV). Reaction solutions consisting of **1 a** and **2 a** (2.20 equiv) were irradiated with an identical number of photons with wavelengths varying from 300 nm to 415 nm and the conversion to the monoadduct **3 a** and diadduct **4 a** was determined by ^1^H NMR spectroscopy. For comparison of the respective conversions with the UV/Vis absorbance spectra of the chromophores refer to Figure 1 B. At longer wavelengths (≥385 nm), the addition of one equivalent of *N*‐ethyl maleimide afforded the cycloadduct **3 a** exclusively. As the photon energy increases, the addition of a second equivalent of *N*‐ethyl maleimide can be observed below 375 nm and eventually the cycloadduct **4 a** becomes the dominant product in the reaction solution below 340 nm. To quantify this observation the reaction quantum yields for the first addition at 385 nm (*Φ*_R1,385 nm_ is 38±7 %), and for the second addition at 350 nm (*Φ*_R2,350 nm_ is 8.8±1.2 %) were determined (for details refer to Supporting Information Section IV), underpinning the wavelength‐dependency of the observed conversions. As a result, the reaction selectivity to form either monoadduct **3 a** or double adduct **4 a** can simply be controlled by the choice of wavelength. For example, by reducing the equivalence of maleimide to 1.02 equiv, and irradiating with 385 nm, the product **3 a** can be formed exclusively (>99 % yield of isolated product). In a situation with no wavelength selectivity, a 1:2:1 mixture of **1 a**, **3 a**, and **4 a** would be the expected outcome of this experiment. The wavelength‐selective nature of the two consecutive reactions is, in part, attributed to a 20 nm hypsochromic shift of the n–π* absorption band of **3 a** compared to **1 a**.

**Figure 2:**
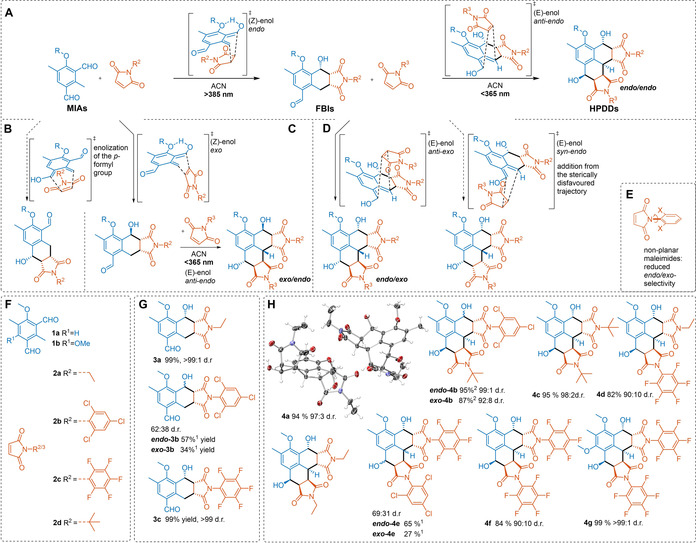
A) Proposed mechanism of the light‐induced (*Z*)‐enol formation of MIA **1 a**, transition state of the subsequent Diels–Alder reaction with maleimides forming *endo*‐FBIs and light‐induced (*E*)‐enol formation of FBIs, transition state of subsequent Diels–Alder reaction with second maleimide resulting in the formation of *endo*/*endo*‐HPDDs. B) Not observed FBI regioisomers by initial enolization of the *p*‐formyl instead of the *o*‐formyl moiety. C) *Exo*‐addition to MIA forming *exo*‐FBI and subsequent anti‐*endo* addition. This reaction sequence is realized in HPDD *exo*‐**4 b**. D) Observed *anti*‐*exo* addition transition state forming the *endo*/*exo* diastereomer, realized in **exo‐4 e** and not observed *syn*‐*endo* addition from the sterically disfavoured trajectory. E) Influence of the non‐planarity of maleimide substrate **2 c** on the *endo*/*exo* selectivity of the Diels–Alder reaction. F) Substrates: MIAs **1 a**, **1 b** and maleimides **2 a**–**2 d**. G) Isolated FBIs **3 a**, *endo*/*exo*‐**3 b**, and **3 c**. H) Isolated HPDDs **4 a**–**4 g**. Triclinic crystal structure (ellipsoids with 50 % probability) of HPDD **4 a**, displayed as pair of enantiomers within the asymmetric unit.^[33]^ Stereocentres in the molecules depicted in (C–H) represent one enantiomer of the obtained racemic mixture. ^1^ Yield of isolated product after diastereomer separation. ^2^ Yield of isolated product from two separate reactions (substrates ***endo*** **‐3 b** and ***exo*** **‐3 b**).

With the ability to now introduce two disparate imide functionalities in a simple synthetic methodology, the mechanism, and scope within which this reaction can be applied, was investigated. While various electron‐deficient dienophiles should participate in these cycloadditions, symmetrical maleimides were chosen in order to limit the diastereomeric complexity. An overview of these results is shown in Figure 2. In Supporting Information Sections II and III the synthetic details are elaborated. For the synthesis of the FBIs, MIA **1 a** and 1.02 equiv of the respective maleimide (**2 a**–**c**) were irradiated with a 385 nm LED. To convert the FBIs to hexahydrophenalene‐1,6‐diol diimides (HPDDs) another 1.20 equiv of the respective maleimide was added and the mixture irradiated with a 365 nm LED. For the HPDDs consisting of two similar maleimides (**4 a**, **4 c**, **4 f**), the MIA **1 a** and 2.25 equiv of the respective maleimide were irradiated with a 365 nm LED. An additional symmetric MIA **1 b** was synthesized via Duff reaction. The photoreaction of **1 b** with maleimide **2 c** was then conducted under irradiation with a 385 nm LED to afford **4 g** in excellent yield (99 %).

As mentioned above, through the choice of symmetrical maleimides the number of possible diastereomeric products is limited. Even still, the HPDDs **4 a**–**g** all feature seven adjacent stereocentres, formed by the two consecutive Diels–Alder reactions. The trajectory for the second maleimide addition can potentially lead to both *syn*‐ or *anti*‐products (Figure 2 D). Furthermore *endo*‐ and *exo*‐addition can occur for both maleimide additions. All the aforementioned reaction pathways combined would lead to a mixture of various diastereomers. For a more detailed discussion of the stereochemistry and a collation of all relevant transition states and diastereomers, refer to Supporting Information Section VII and Scheme S1 and S2. In general however, Diels–Alder reactions of *o*‐QDMs have a high level of diastereoselectivity (*endo*‐selective) at ambient temperature, especially in polar solvents such as acetonitrile.^[23]^ By variation of the dienophiles, this selectivity is altered, especially if bulky substituents stand out of the double‐bond plane and hinder the formation of the *endo*‐transition state (Figure 2 E).

For the addition of the first equivalent of planar maleimides **2 a** and **2 c**, over 99 % *endo*‐selectivity was observed in the respective products **3 a** and **3 c**. By comparison, the maleimide **2 b**, which exhibits a rotational barrier of the C−N bond, due to the *ortho*‐chloro substitution, afforded a mixture of the *endo*‐ and *exo*‐product (***endo*** **‐3 b**/***exo*** **‐3 b**), which were separable by chromatography. The stereochemical orientation of the first addition strongly influences the trajectory of the second addition as a consequence of the bridgehead benzylic position. In none of the cases was a product observed in which both maleimides face in the same direction, resulting from a *syn*‐addition from the hindered trajectory. By deliberately exploiting the non‐planarity of maleimide **2 b**, and the previously observed influence on the diastereoselectivity, *endo/exo* substitution patterns for the first (***endo*** **‐3 b**/***exo*** **‐3 b**) and second maleimide addition (***endo*** **‐4 e**/***exo*** **‐4 e**) were accessible. Thus, from the *anti*‐orientation of the bridgehead hydrogen atom, and the newly formed hydroxy group for both *endo*‐ and *exo*‐additions (***endo*** **‐4 e**/**exo‐4 e**), one can conclude that the (*E*)‐enol must be the reactive intermediate in the second cycloaddition. This finding is in agreement with the Woodward–Hofmann rules and the commonly accepted photochemical mechanism.^[24]^

Comparisons between the various synthesized HPDDs allows for other conclusions to be drawn in regard to the stereochemistry. For example, by comparing HPDDs **4 a** and **4 c**, we can deduce that the steric bulk of the N‐substituent of the maleimide only has a minor influence on the stereochemical outcome of the second addition. And by comparing **4 f** and **4 g**, one can observe that the methoxy donor adjacent to the second formyl group, present in MIA **1 b**, enhances the stereoselectivity, presumably by forming a hydrogen bridge with the *ortho*‐quinodimethane OH. The influence of the initially added maleimide was found to have a negligible effect on the stereoselectivity, as seen by comparing HPDDs **4 b** and **4 c**, however, a reduced *endo*‐selectivity for the second addition is observed when *exo*‐FBIs are utilised for the second addition (e.g. **4 b**). Finally, to gain confirmation of the structure, **4 a** was crystallized from the crude reaction mixture of an up‐scaled continuous flow photoreaction. Single‐crystal XRD (for details refer to Supporting Information Section X) revealed the absolute configuration of the molecule, confirming the proposed mechanism presented in Figure 2 A. Furthermore, *exo*‐**4 a** was isolated in a marginal quantity of 2 %.

In addition, the importance of symmetry in the MIAs for the wavelength‐gated nature of the Diels–Alder additions was also investigated. The alkoxygroup(s) in MIAs **1 a** and **1 b** are known to form an intramolecular hydrogen bond in the (*Z*)‐enol.^[24]^ A direct comparison of the lifetime of photogenerated enols in *ortho*‐OMe‐substituted and unsubstituted *o*‐MBAs,^[25]^ as well as the observed increase in the reaction efficiency and selectivity of *ortho*‐OMe‐substituted *o*‐methyl benzoyl cyanides compared with their unsubstituted analogues,^[26]^ afford the conclusion that alkoxy groups in *ortho*‐position to the carbonyl group facilitate the enolization process.

For non‐symmetric **1 a**, the first *o*‐QDM is formed upon irradiation involving the *ortho*‐formyl group; whereas the *para*‐formyl group solely reacts when the first formyl group is converted (Figure 2 A). Enolization of the *para*‐formyl group, and the respective cycloadduct (Figure 2 B), was not observed for any substrate combination. For the *C*_2*v*_‐symmetric MIA **1 b** both formyl groups are equivalent and activated in the *ortho*‐position to alkoxy groups and, as a result, stepwise control over the two consecutive Diels–Alder reactions via disparate wavelengths is forfeited as both additions are occurring at 385 nm. We therefore assume that the absence of an alkoxy group adjacent to the second formyl group in the FBIs decreases the quantum yield of the second enolization and/or the lifetime of the (*E*)‐enol. This reduced reaction rate consequentially facilitates the wavelength‐gated consecutive addition and explains the observed regioselectivity conclusively. To summarize, the reaction we have presented here allows control over the consecutive addition of two equivalents of disparate maleimides to a single photoreactive entity, generating a molecule with 7 stereocentres under remarkable stereochemical selectivity (62–99 % yields of isolated product, 80–98 % d.r.). The modular nature of this chemistry and the control over functionality allow tailoring of the resulting molecules.

**Figure 3:**
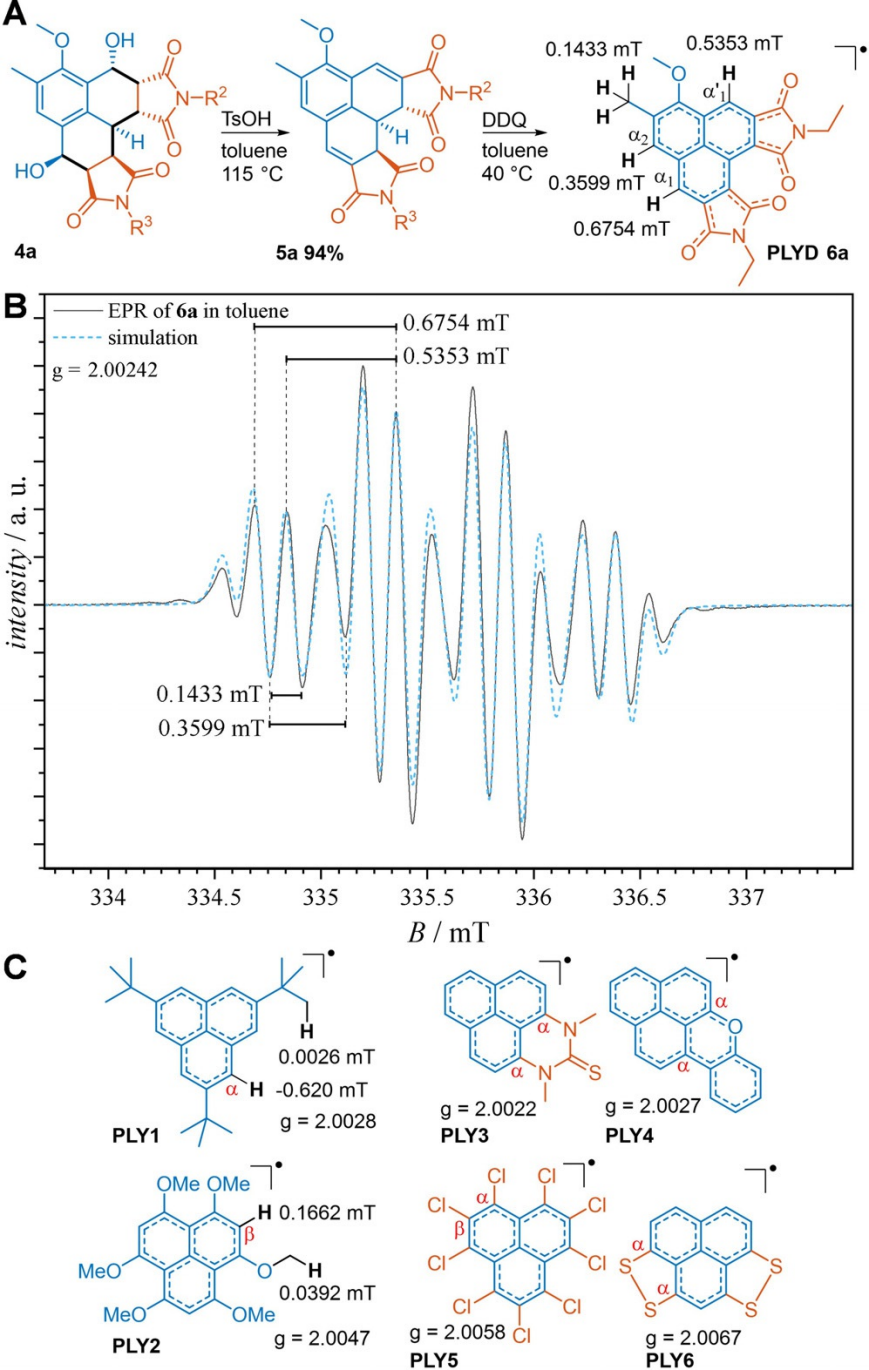
A) Synthesis of phenalenyl diimide neutral radical **6 a** from HPDD **4 a**. B) EPR spectrum of **6 a** in toluene at 25 °C (black line) and simulation of the spectrum (blue dotted line) C) Most stable phenalenyl diimide neutral radical **6 g**. D) Selected examples of persistent phenalenyl neutral radicals (**PLY1‐6**) from literature.[^10^, ^27^, ^28^, ^29^, ^30^, ^31^]

To obtain the desired phenalenyl diimide neutral radicals (PLYDs), the phenalene scaffold of the HPDDs was further modified after the photoreaction (Figure 3). To this end, the two hydroxy groups were removed via acid catalysis and the intermediate was subsequently oxidized. In the process of establishing this route, several attempts to convert **4 a** to **5 a** and finally **6 a** were made, and additional valuable transformations of **4 a** were established (for continuative discussion, refer to Supporting Information Section VII). Finally, **6 a** was isolated and characterized via EPR spectroscopy. It was found that **6 a** is stable in deoxygenated solution and in the solid state, yet decomposes to a phenalenone derivative under ambient conditions within several days. The EPR spectrum of **6 a** is depicted in Figure 3 B along with a simulated spectrum based on the denoted hyperfine coupling constants (hfccs). The g‐factor and the high hfcc values for α_1_ and α_1_′ emphasize the highly delocalized spin density in this radical, especially in comparison with previously reported **PLY1**, which is only kinetically stabilized by three β‐*tert*‐butyl groups.^[27]^ Despite this observation, the formation of σ‐dimers from **6 a** was not detected at ambient temperatures. The α‐OMe group did not result in any significant hyperfine coupling, contrary to earlier reports for various phenalenyl radicals, for example **PLY2**.^[10]^ Any superhyperfine coupling arising from the OMe group is most likely obscured by the signal bandwidth in this case.

The most effective way to stabilize phenalenyl‐based neutral radicals is to delocalize the spin density to an adjacent π‐system, for instance realized in **PLY3** ^[28]^ and **PLY4**.^[29]^

Conjugative or inductive effects of heteroatom substituents are also a possibility for enhancing the stability of the radical, as demonstrated by **PLY5** ^[30]^ and **PLY6**.^[31]^ A recent theoretical study of different phenalenyl radicals revealed that the best thermodynamic stabilization is provided by either π‐conjugated or lone‐pair substituents with low electronegativity, for instance ‐CN or ‐NH_2_, respectively, when attached to a α‐position.^[32]^ **6 a** represents the first example where the stabilization of a phenalene radical is provided by pendant imide or maleimide groups. We hypothesize that a push–pull effect of the α‐OMe and the α‐imine units of the two maleimides provides additional stabilization. To confirm this, three additional PLYD radicals were synthesized from HPDDs using an identical procedure (refer to Supporting Information Section VII, g‐factors and hffcs are summarized in Table S2). By introducing electron‐withdrawing aryl groups on the maleimides and an additional OMe group in the α_2_ position, a PLYD radical derived from **4 g** was obtained which possessed increased stability at ambient conditions for several weeks allowing for additional LCMS and cyclic voltammetry analysis (refer to Supporting Information Section VII). Also, a heterobifunctional PLYD radical derived from **4 d** could be easily obtained. An additional systematic study of the structure–property relationship of the herein presented phenalenyl diimide neutral radicals and detailed DFT calculations of the molecular orbitals are envisaged for the future.

## Conclusion

The methodology presented herein combines the high efficiency of click chemistry with the spatiotemporal control of light‐induced reactions, enabling the wavelength‐gated generation of heterobifunctional phenalene diimides. The wavelength dependency of the two consecutive *ortho*‐quinodimethanes was studied in detail employing a monochromatic light source. The diastereoselectivity of the cycloadditions was investigated by combining planar and non‐planar maleimide substrates and independently confirmed via single‐crystal XRD. The approach represents the first wavelength‐selective bond forming of a single chromophore and holds key potential for the light‐driven assembly of complex architectures.

Subsequent transformation of the phenalene diimides to a new class of persistent neutral radicals (PLYDs) was established and the produced spin‐active molecules were studied. Our approach thus greatly simplifies the synthetic procedures in organic spin chemistry and allows for the design of tailor‐made electronic spin structures.

## Conflict of interest

The authors declare no conflict of interest.

## Supporting information

As a service to our authors and readers, this journal provides supporting information supplied by the authors. Such materials are peer reviewed and may be re‐organized for online delivery, but are not copy‐edited or typeset. Technical support issues arising from supporting information (other than missing files) should be addressed to the authors.

Supplementary
