# Supplementary material for: Wavelength‐Gated Photochemical Synthesis of Phenalene Diimides
Source: Angew Chem Int Ed Engl. 2021 Mar 18;60(18):10402–8. doi: 10.1002/anie.202016632 (PMC8251713; doi:10.1002/anie.202016632)
Supplement: Supplementary file 1 — Supplementary [file ANIE-60-10402-s001.pdf]

Angewandte Chemie
Eine Zeitschrift der Gesellschaft Deutscher Chemiker

Supporting Information

Wavelength-Gated Photochemical Synthesis of Phenalene Diimides

Florian Feist, Sarah L. Walden, Jessica Alves, Susanna V. Kunz, Aaron S. Micallef, Aidan J. Brock, John C. McMurtrie, Tanja Weil, James P. Blinco, and Christopher Barner-Kowollik**

anie_202016632_sm_miscellaneous_information.pdf

Author Contributions

F.F. Conceptualization: Lead; Data curation: Lead; Formal analysis: Lead; Investigation: Lead; Methodology: Lead; Project administration: Lead; Resources: Lead; Software: Equal; Validation: Lead; Visualization: Lead; Writing – original draft: Equal; Writing – review & editing: Lead
S.W. Data curation: Equal; Formal analysis: Supporting; Methodology: Supporting; Software: Lead; Supervision: Equal; Visualization: Supporting; Writing – original draft: Equal; Writing – review & editing: Equal
J.A. Conceptualization: Supporting; Methodology: Supporting; Writing – review & editing: Supporting
S.K. Conceptualization: Supporting; Writing – review & editing: Supporting
A.M. Formal analysis: Supporting; Investigation: Supporting; Methodology: Supporting; Validation: Supporting; Writing – review & editing: Supporting
A.B. Data curation: Supporting; Formal analysis: Supporting; Validation: Supporting
J.M. Resources: Supporting; Writing – review & editing: Supporting
T.W. Supervision: Supporting; Validation: Supporting; Writing – review & editing: Supporting
J.B. Conceptualization: Lead; Funding acquisition: Supporting; Supervision: Lead; Writing – original draft: Supporting; Writing – review & editing: Supporting.
C.B.-K. Conceptualization: Lead; Funding acquisition: Lead; Supervision: Lead; Writing – original draft: Supporting; Writing – review & editing: Supporting.

Table of Contents

I. Materials and Methods....3
II. Synthetic Procedures for 2-methylisophthalalehydes (MIAs) and Maleimides....5
III. Procedures for the Photoreactions....8
IV. Tunable Laser Studies....16
V. Procedures for the Transformations of HPDDs....20
VI. Procedures for the Generation of PLYD Neutral Radicals....26
VII. Results and Discussion....28
VIII. EPR spectra and Simulations....33
IX. Cyclic Voltammetry....37
X. Single Crystal XRD....39
XI. UV-VIS Spectra....40
XII. Fluorescence Spectra and Fluorescence Quantum Yields....40
XIII. NMR Spectra....44
XIV. LC-HRMS Traces, Spectra and Fragmentation Patterns....116
XV. References....151
XVI. Author Contributions....151

I. Materials and Methods

Materials: The following chemicals were used as received without further purification:
Trifluoracetic acid (99+ %, Thermo Fischer), 2,5-dimethylphenol (99% Sigma-Aldrich), hexamethylenetetramine (99+ % Sigma-Aldrich), hydrochloric acid (4 N, reagent grade, Chem-Supply), potassium carbonate (anhydrous for analysis, 99+%), *N*-ethylmaleimide (98+% HPLC, Sigma-Aldrich), tert-butyl maleimide (98%, Combi-Blocks), 2,4,6-trichlorophenyl maleimide (95 %, combi-blocks), pentafluoroaniline (97 %, combi-blocks), maleic anhydride (99% Sigma-Aldrich),), para-toluensulfonic acid monohydrate (99%, Chem-Supply), 2,3-Dichloro-5,6-dicyano-1,4-benzoquinone (DDQ, 99%, Sigma-Aldrich), 2,5-dimethylresorcineol (Combi-Blocks, 97 %) toluene (HPLC-grade, Thermo Fischer), sodium sulfate (99.5 %, Chem-Supply), acetonitrile (HPLC-grade, Fisher), methanol (analytical reagent, Ajax Finechem), chloroform (analytical reagent, Fisher), cyclohexane (CH, analytical reagent, Ajax Finechem), ethyl acetate (EE, analytical reagent, Fisher), dichloromethane (DCM, analytical reagent, Fisher), acetonitrile-d^3 (99.8 %D, Cambridge Isotope Laboratories), chloroform-d (99.8 %D, Cambridge Isotope Laboratories), dimethylsulfoxide-d^6 (99.9 %D, Cambridge Isotope Laboratories).

Flash Chromatography: Flash chromatography was performed on a Interchim XS420+ flash chromatography system consisting of a SP-in-line filter 20-µm, an UV-VIS detector (200-800 nm) and a SofTA Model 400 ELSD (55 °C drift tube temperature, 25 °C spray chamber temperature, filter 5, EDR gain mode) connected via a flow splitter (Interchim Split ELSD F04590). The separations were performed using an Interchim dry load column and a Interchim Puriflash Silica HP 30 µm column. The crude materials were deposited on celite 545 prior to chromatography.

Preparative HPLC: Preparative HPLC was performed on a *Interchim PF5.250* HPLC system consisting of a SP-in-line filter 20-µm, an UV-VIS detector (200-800 nm) and a *Nano-IELSD* (45 °C drift tube temperature,) connected via a dynamic flow splitter flow splitter. The separations were performed using a *direct injection* via an injection valve and an *Interchim Uptisphere Silica HP 5* µm column with 21.2 mm diameter and 250 mm length equipped with a pre-column filled with 5 µm silica.

NMR Spectroscopy: ^{1}H-,^{13}C-, ^{19}F-, ^{1}H/^{1}H-COSY-, ^{1}H/^{13}C HSQC- and ^{1}H/^{13}C-HMBC spectra were recorded on a Bruker System 600 Ascend LH, equipped with a BBO-Probe (5 mm) with z-gradient (^{1}H: 600.13 MHz, ^{13}C: 150.90 MHz,). All measurements were carried out in deuterated solvents. The chemical shift (δ) is recorded in parts per million (ppm) and relative to the residual solvent protons.[2] The measured coupling constants were calculated in Hertz (Hz). To analyze the spectra, the software MESTRENOVA 11.0 was used. The resonances are quoted as follows: s = singlet, bs = broad singlet, d = doublet, t = triplet, q = quartet, quin = quintet, dd = doublet of doublets and m = multiplet. Resonance assignments are based on COSY, HSQC and HMBC measurements. The herein synthesized products are racemic. Only one enantiomer is depicted and named.

LC-MS Measurements: LC-MS measurements were performed on an UltiMate 3000 UHPLC system (Dionex, Sunnyvale, CA, USA) consisting of a pump (LPG 3400SZ, autosampler WPS 3000TSL) and a temperature-controlled column department (TCC 3000). Separation was performed on a C18 HPLC-column (Phenomenex Luna 5µm, 100 Å, 250 × 2.0 mm) operating at 40 C. A gradient of ACN:H_2O 10:90 – 80:20 *v/v* (additive 10 mmol L^{-1} $NH_4CH_3CO_2$) at a flow rate of 0.20 mL·min^{-1} during 15 min was used as the eluting solvent. The flow was split in a 9:1 ratio, where 90 % (0.18 mL·min^{-1}) of the eluent were directed through the UV-detector (VWD 3400, Dionex, detector wavelengths 215, 254, 280, 360 nm) and 10 % (0.02 mL·min^{-1}) were infused into the electrospray source. Spectra were recorded on an LTQ Orbitrap Elite mass spectrometer (Thermo Fisher Scientific, San Jose, CA, USA) equipped with a HESI II probe. The instrument was calibrated in the *m/z* range 74-1822 using premixed calibration solutions (Thermo Scientific). A constant spray voltage of 3.5 kV, a dimensionless sheath gas and a dimensionless auxiliary gas flow rate of 5 and 2 were applied, respectively. The capillary temperature and was set to 300 °C, the S-lens RF level was set to 68, and the aux gas heater temperature was set to 125 °C.

UV-VIS Spectroscopy: UV/vis spectra were recorded on a *Shimadzu* UV-2700 spectrophotometer equipped with a CPS-100 electronic temperature-controlled cell positioner. Samples were measured in *Hellma Analytics* quartz high precision cells with a path length of 10 mm at ambient temperature. For extinction coefficients 3 samples were prepared, measured and used for the calculation.

Fluorescence Spectroscopy: The fluorescence spectra and kinetics were measured using a Cary Eclipse Fluorescence Spectrophotometer from Agilent Technologies. The sample preparation and the experimental parameters (excitation

wavelength, excitation and emission slit, scan rate and detector voltage are described for each individual experiment. Samples were measured at ambient temperature using quartz cuvettes with 1 cm path-length.

Fourier-Transform Infrared Spectroscopy (FTIR): Infrared spectra were obtained on a Bruker research spectrometer VERTEX 80 using an ATR mount with a ZnSe crystal and are reported as follows: frequency of absorption (cm^{-1}), intensity of absorption (s = strong, m = medium, w = weak, br = broad).

Elemental Analysis: N/C/H Elemental analysis was performed *via* thermal combustion with an Elementar Vario Microcube Instrument (Elementar Analysensysteme GmbH, D-63505 Langenselbold) at a temperature of about 1100 °C. Prior to analysis, the samples were pestled and dried in high-vacuum for 72 h to remove all volatiles.

Batch Photoreactions: The batch photoreactions were performed using 20 mL crimp-cap vials and LEDs with an emission centered at 365 and 385 nm. The LEDs (Luminus Devices, Inc. SBM-120-UV-F34-H365-22 (365 nm); SBM-120-UV-F34-L385-22 (385 nm), were powered by a variable power supply (750 mA current) and positioned in a distance of 3 cm measured from the center of the vials. The LEDs and vials were cooled by a stream of compressed air to maintain ambient temperature.

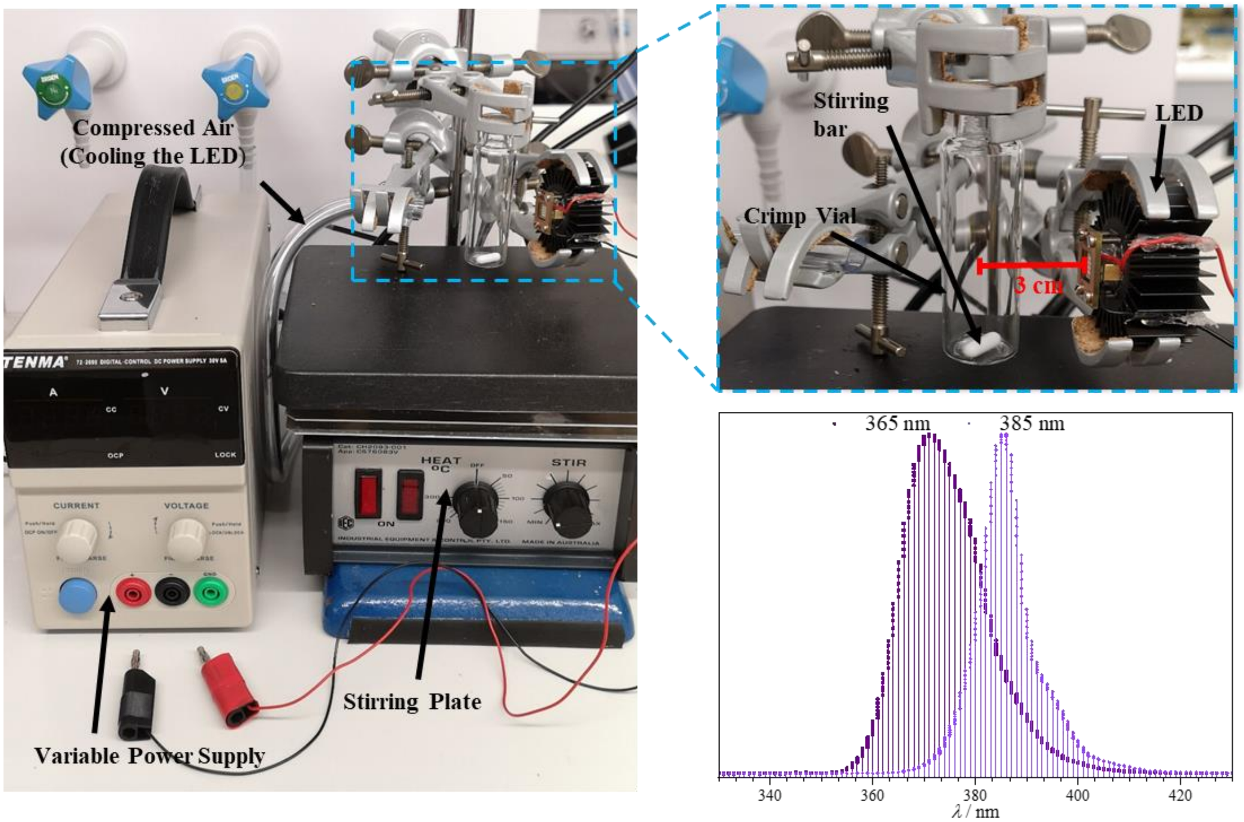

Figure S1 Setup for the irradiation of 20 mL crim-cap vials for the batch experiments emission spectra of the light sources. The LEDs and the reaction mixture were cooled using a steady flow of compressed air at ambient temperatures.

Flow Photoreactions: Photoreactions under continuous flow conditions were performed using a Vapourtec E-series platform (peristaltic pumps) in combination the UV-150 module and the VSD006 cooling module. The module consists of a temperature-controlled irradiation chamber, a transparent fluorinated ethylene polymer (FEP) reactor coil (1.3 mm inner diameter, 0.15 mm wall thickness, 10 mL PN: 50-1288) and a LED assembly (360 to 390 nm, peak 365 nm, total power output of 16 W, PN: 50-4036). The temperature is controlled employing pre-cooled nitrogen (heat exchange in the cooling module).

Single-crystal XRD: Single-crystal diffraction data were collected on an XtaLAB Synergy Dualflex diffractometer (Rigaku) equipped with Pilatus3 R CdTe 300K detector (Dectris) at 150.00(10) K, using microfocused MoK_{α} (λ = 0.71073) radiation generated from a sealed tube. Data were indexed integrated and reduced within CrysAlis Pro[1]. Structure solution was undertaken with SHELXT[2]. Data was refined via a least-squares method against F^2 using SHELXL[3], implemented within the Olex2 user interface[4]. Hydrogen atoms were placed in idealized positions and refined using a riding model on appropriate

atoms. All non-hydrogen atoms with occupancies greater than 50% were refined anisotropically, while low-occupancy disordered solvent molecules were refined using an isotropic model.

EPR Spectroscopy and Simulations: EPR spectra were recorded on a Magnettech MiniScope MS400 spectrometer at ambient temperature. The g-factors were obtained by addition of the internal reference Cu(acac)$_2$, g=2.126 in toluene[5] to the solution. The simulations/fitting of the EPR spectra was performed using the EASYSPIN toolbox for MATLAB® [6]. The optimization parameters are given with the results.

Cyclovoltammetry (CV): Cyclic voltammograms were recorded at ambient temperature with a Biologic® SP-150 potentiostat under inert conditions after degassing with a stream of nitrogen. All measurements were carried out in 2.5 mL sample aliquots using a standard 3 electrode electrochemical cell. A glassy carbon electrode with a surface area of 7.85×10^{-3} cm^2 was used as the working electrode with rigorous cleaning using an aluminum pad for gentle surface abrasion for surface particulates removal and washed with acetone and deionized water. A solid platinum wire counter electrode and silver/silver chloride leak-tight reference electrode were used in each experiment, both cleaned using acetone and deionized water before use.

II. Synthetic Procedures for 2-methylisophthalalehydes (MIAs) and Maleimides

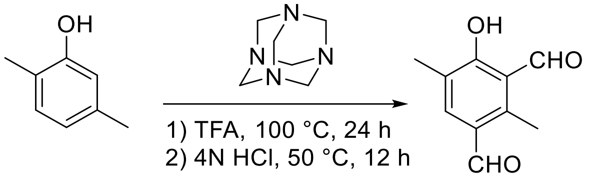

4-hydroxy-2,5-dimethylisophthalaldehyde: In a 250 mL round bottom flask, a solution of 2,5-dimethylphenol (5.00 g, 40.93 mmol, 1.00 eq) in 32.7 mL TFA was prepared and hexamethylenetetramine (20.80 g, 163.71 mmol, 4.00 eq) was added. The resulting viscous solution was stirred under inert atmosphere at 100 °C in an oil bath for 24h. Afterwards, the reaction mixture was cooled to ambient temperature and 72 mL 4 N HCl were added. The mixture was heated to 50°C whilst passing through nitrogen for 12 h. Finally, the resulting solution was cooled at ambient temperature, diluted with 50 mL water and cooled in a refrigerator at 7 °C overnight. The resulting precipitate is filtered off, washed with 10 mL cold water and dried in vacuum. The resulting crude product was either purified *via* flash chromatography (EE:CH 10:90-20:80 *v/v*) or sublimated under reduced pressure at 60 °C. The product is obtained as slightly yellow crystals (4.30 g, 59 % yield).

The NMR spectra are consistent with earlier reported results[7], refer to **Figure S18** and **Figure S19**.

^{1}H NMR (600 MHz, Chloroform-*d*) δ: 12.98 (s, 1H), 10.48 (s, 1H), 10.24 (s, 1H), 7.86 (s,1H), 2.93 (s, 3H), 2.27 (s, 3H).
^{13}C NMR (151 MHz, Chloroform-*d*) δ: 195.58, 190.06, 166.38, 144.16, 140.00, 126.25, 126.12, 117.81, 15.05, 12.14.
FTIR (neat) cm^{-1}: 2923 (w, br), 1672 (s), 1633 (s), 1576 (m), 1413 (m), 1378 (m), 1344 (m), 1304 (m),
1282 (s), 1261 (s), 1205 (m), 1139 (m), 1049 (m), 1034 (s), 979 (m), 933 (w), 874 (w), 829 (s), 759 (m), 726 (m), 688 (m), 658 (m), 623 (m).
Melting Point: 160 °C (162°C Lit.[7])
Elemental Analysis: calculated for $C_{10}H_{10}O_3$: C: 67.41, H: 5.66 − found: C:67.32, H: 4.96.

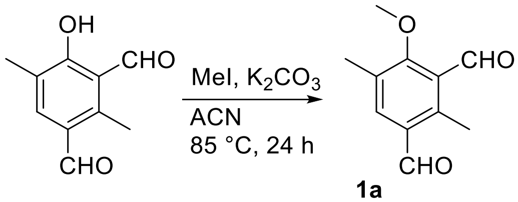

1a

4-methoxy-2,5-dimethylisophthalaldehyde (1a): 4-Hydroxy-2,5-dimethylisophthal-aldehyde (3.00 g, 16.84 mmol, 1.00 eq) was dissolved in 100 mL dry acetonitrile under inert atmosphere. Methyl iodide (1.57 mL, 3.58 g, 1.50 eq) was added *via* syringe. Anhydrous K_2CO_3 (2.70 g, 21.05 mmol, 1.25 eq) was then added and the suspension stirred at 85 °C for 24 h until complete consumption of the starting material. Afterwards the reaction mixture is cooled to ambient temperature, 150 mL 0.1 N HCl and 250 mL ethyl acetate were added, the organic phase separated, the aqueous phase washed twice with 50 mL ethyl

acetate and the combined organic phases washed with brine, dried over $MgSO_4$, the volatiles removed under reduced pressure and the residual crude product was purified *via* flash chromatography (isocratic cyclohexane: ethyl acetate 85:15 *v/v*). The product was obtained as colorless crystals (3.04 g, 94 % yield).

^{1}H NMR (600 MHz, Chloroform-d) δ: 10.54 (s, 1H), 10.37 (s, 1H), 7.90 (s, 1H), 3.88 (s,3H), 2.83 (s, 3H), 2.35 (s, 3H).
^{13}C NMR (151 MHz, Chloroform-d) δ: 192.90, 190.86, 166.80, 142.19, 138.03, 131.25,130.19, 129.36, 62.99, 15.58, 14.25.
FTIR (neat) cm^{-1}: 3053 (w), 2932 (m), 2982 (m) 2770 (m), 2727 (w), 1686 (s), 1592 (m), 1567 (s), 1470 (s), 1437 (m), 1405 (m), 1372 (s), 1279 (s), 1242 (s), 1211 (s), 1148 (w), 1082 (s), 1060 (s), 1008 (m), 984 (s), 913 (s), 873 (s), 759 (s), 683 (m), 634 (s), 584 (s), 550 (m), 501 (m).
Melting Point: 82 °C
Elemental Analysis: calculated for $C_{11}H_{12}O_3$: C: 68.74, H: 6.29 − found: C: 68.64, H: 5.46.

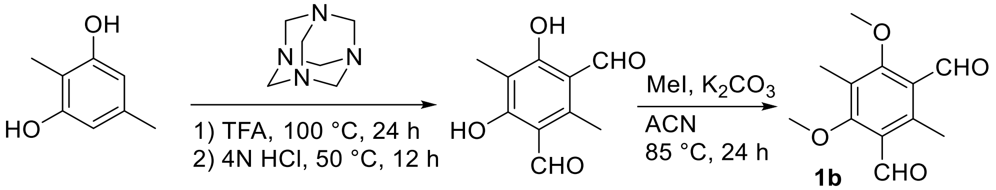

1b

4,6-dimethoxy-2,5-dimethylisophthalaldehyde (1b): In a 25 mL round bottom flask, a solution of 2,5-dimethylresorcineol (500 mg, 3.62 mmol, 1.00 eq) in 2.9 mL TFA was prepared and hexamethylenetetramine (1.84 g, 14.58 mmol, 4.00 eq) was added. The resulting viscous solution was stirred under inert atmosphere and at to 100 °C in an oil bath for 24h. Afterwards, the reaction mixture was cooled at ambient temperature and 6.5 mL 4 N HCl were added. The mixture was heated to 50°C whilst passing through nitrogen for 12 h. Finally, the resulting solution was cooled at ambient temperature, diluted with 50 mL water and cooled in a refrigerator at 7 °C overnight. The resulting precipitate was filtered off, washed with 10 mL cold water and dried in vacuum. The resulting crude product was dissolved in 15 mL dry acetonitrile under inert atmosphere. Methyl iodide (675 µL, 1.54 g, 3.00 eq) was added *via* syringe. Subsequently, anhydrous K_2CO_3 (974 mg, 7.60 mmol, 2.1 eq) was added and the suspension stirred at 85 °C for 24 h until complete consumption of the starting material. Afterwards, the reaction mixture was cooled to ambient temperature, 30 mL 0.1 N HCl and 50 mL ethyl acetate were added, the organic phase separated, the aqueous phase washed twice with 50 mL ethyl acetate and the combined organic phases washed with brine, dried over $MgSO_4$, the volatiles removed under reduced pressure and the residual crude product was purified *via* flash chromatography (isocratic cyclohexane: ethyl acetate 80:20 *v/v*). The product was obtained as beige solid (257 mg, 32 % yield).

^{1}H NMR (600 MHz, ACN-*d*$_3$) δ: 10.42 (s, 2H), 3.85 (s, 6H), 2.64 (d, J = 0.7 Hz, 3H),2.23 (d, J = 0.7 Hz, 3H).
^{13}C NMR (151 MHz, ACN-*d*$_3$) δ: 193.34, 167.94, 142.60, 127.04, 124.89, 63.57, 16.17, 9.14.
FTIR (neat) cm^{-1}: 2945 (m), 2793 (m), 2766 (w), 1695 (s), 1684 (s), 1556 (s), 1450 (m), 1383 (m), 1285 (s), 1192 (m), 1105 (s), 1082 (s), 989 (m), 957 (s), 864 (s), 779 (m), 723 (m), 690 (m).
Melting Point: 125 °C
Elemental Analysis: calculated for $C_{12}H_{14}O_4$: C: 64.85, H: 6.35 − found: C: 64.71, H: 5.50.

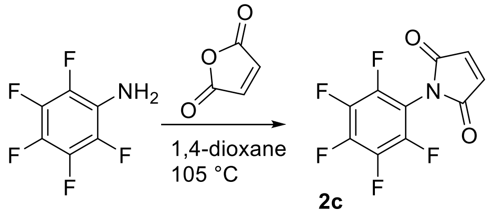

2c

1-(perfluorophenyl)-1*H*-pyrrole-2,5-dione (2c): The synthesis of 1-(perfluorophenyl)-1*H*-pyrrole-2,5-dione was carried out following a modified literature procedure.[8]

5.00 g (27.31 mmol, 1.00 eq) pentafluoro aniline were dissolved in 9 mL dry 1,4-dioxane and added to a solution of 2.70 g (27.58 mmol, 1.01 eq) maleic anhydride in 14 mL dry 1,4-dioxane in a 100 mL Schlenk flask. The mixture was afterwards heated to 105 °C under argon atmosphere. Afterwards, approximately 2/3 of the solvent were removed under reduced pressure using a cooling trap and the mixture was cooled to ambient temperature. 25 mL anhydrous acetic acid was added,

and the reaction mixture heated to 125 °C for 18 h. The volatiles were removed under reduced pressure at 80 °C using a cooling trap. The beige solid product was purified *via* sublimation under reduced pressure (1 mbar, 120 °C bath temperature). The sublimate was dissolved in 50 mL chloroform, the white residue filtered off and the filtrate concentrated under reduced pressure. Subsequently, the slightly yellow residue was further purified by recrystallization from ethanol/hexane 1:4 *v/v* (approx. 120 mL, crystallization at -18 °C overnight) The pure product was obtained as colorless crystals (4.68 g, 63.0 % yield). The impurity of the ring opened intermediate, reported in the literature procedure[8] could be avoided by the herein described process.

^{1}H NMR (600 MHz, $CDCl_3$) δ: 6.98 (s, 2H).
^{13}C NMR (151 MHz, $CDCl_3$) δ: 166.91, 146.98 (ddq, *J* = 165.18, 12.4, 4.1 Hz), 142.30 (dtt, *J* = 258.2, 13.2, 4.5 Hz) , 139.22 – 137.12 (m), 135.46, 106.61 – 106.31 (m).
^{19}F NMR (565 MHz, $CDCl_3$) δ: -142.80 – -143.08 (m), -151.01 (t, *J* = 21.3 Hz), -160.55 – -161.28 (m).
FTIR (neat) cm^{-1}: 3090 (w), 1729 (s), 1511 (s), 1361 (m), 1305 (m), 1171 (w), 1148 (m), 1142 (m), 1078 (m), 1049 (m), 1032 (w) 980 (s), 844 (m), 821 (m), 778 (w), 726 (m), 690 (s), 640 (m) 577 (w).
Melting Point : 108-109 °C

III. Procedures for the Photoreactions

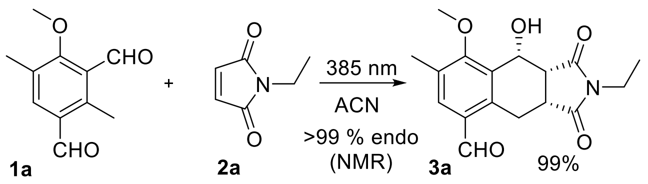

(3a*R*,9*R*,9a*R*)-2-ethyl-9-hydroxy-8-methoxy-7-methyl-1,3-dioxo-2,3,3a,4,9,9a-hexahydro-1*H*-benzo[*f*]isoindole-5-carbaldehyde (3a): 4-Methoxy-2,5-dimethylisophthalaldehyde (**1a**, 28.8 mg, 0.15 mmol, 1.00 eq) and *N*-ethyl maleimide (**2a**, 19.14 mg, 0.153 mmol 1.02 eq) were dissolved in 15 mL dry acetonitrile (20 mL crimp vial, 10 mmol L^{-1}). The solution was degassed by passing through N_2 for 15 min and irradiated for 40 min (385 nm LED). Next, the solvent was removed under reduced pressure and the crude product was purified *via* flash chromatography (isocratic, DCM:MeOH 99:1 *v/v*). The racemic *endo*-product was obtained as colorless crystals (47.1 mg, 99 % yield).

^{1}H NMR (600 MHz, ACN-d_3) δ: 10.22 (s, 1H), 7.67 (d, *J* = 0.9 Hz, 1H), 5.61 (d, *J* = 3.9 Hz, 1H), 4.22 (dd, *J* = 15.2, 8.3 Hz, 1H), 3.82 (s, 3H), 3.56 (s, 1H), 3.52 (qd, *J* = 7.2, 2.7 Hz, 2H), 3.11 (ddd, *J* = 10.8, 9.7, 8.3 Hz, 1H), 2.94 (dd, *J* = 9.7, 3.9 Hz, 1H), 2.92 (ddd, *J* = 15.2, 10.7, 1.1 Hz, 1H), 2.33 (d, *J* = 0.9 Hz, 3H), 1.13 (t, *J* = 7.2 Hz, 3H).
^{13}C NMR (151 MHz, ACN-d_3) δ: 192.53, 180.84, 177.67, 160.62, 140.40, 134.92, 133.93, 130.98, 130.86, 62.57, 62.55, 47.25, 38.16, 34.18, 22.38, 16.05, 13.25.
LC-HRMS: (refer to **Figure S90, Table S3** and **Scheme S6**)
FTIR (neat) cm^{-1}: 3481 (m, br), 2999 (w), 2979 (w), 2952 (m), 2920 (w), 2876 (w), 1767 (m), 1688 (s), 1672 (s), 1599 (m), 1576 (m), 1475 (m), 1443 (m), 1402 (s), 1377 (m), 1347 (s), 1292 (m), 1276 (s), 1249 (m), 1224 (s), 1192 (m), 1180 (m), 1136 (m), 1086 (m), 1060 (s), 1032 (m), 1006 (s), 956 (m), 931 (m), 887 (m), 854 (w), 823 (m), 800 (w), 791 (w), 756 (m), 711 (m), 684 (m), 650 (m), 611 (m).
Melting Point: 156-157 °C
Elemental Analysis: calculated for $C_{17}H_{19}NO_5$: C: 64.34, H: 6.04 − found: C: 64.71, H: 5.59.

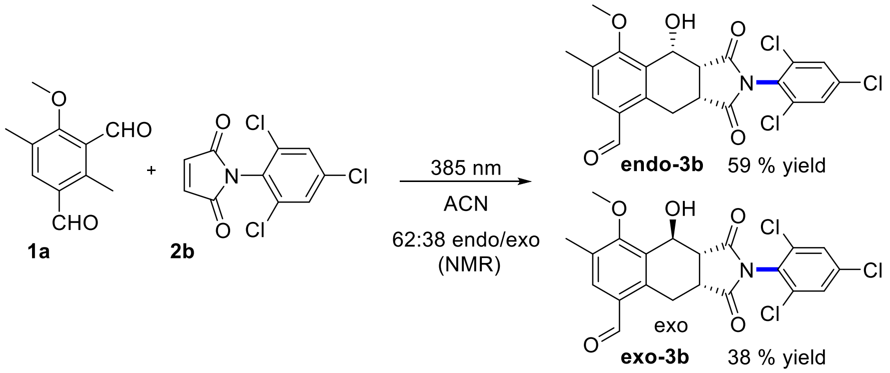

(3a*R*,9*R*,9a*R*)-9-hydroxy-8-methoxy-7-methyl-1,3-dioxo-2-(2,4,6-trichlorophenyl)-2,3,3a,4,9,9a-hexahydro-1*H*-benzo[*f*]isoindole-5-carbaldehyde (*endo*-3b) and (3a*R*,9*R*,9a*R*)-9-hydroxy-8-methoxy-7-methyl-1,3-dioxo-2-(2,4,6-trichlorophenyl)-2,3,3a,4,9,9a-hexahydro-1*H*-benzo[*f*]isoindole-5-carbaldehyde (*exo*-3b): 4-Methoxy-2,5-dimethyl-isophthalaldehyde (**1a**, 28.8 mg, 0.15 mmol, 1.00 eq) and 2,4,6-trichlorophenyl maleimide (**2b**, 42.15 mg, 0.153 mmol 1.02 eq) were dissolved in 15 mL dry acetonitrile (20 mL crimp vial, 10 mmol L^{-1}). The solution was degassed by passing through N_2 for 15 min and irradiated for 40 min (385 nm LED). Afterwards, the solvent was removed under reduced pressure and the crude product was purified *via* preparative HPLC (gradient, DCM:MeOH 99:1-95:5 *v/v*). The racemic *endo*-product was obtained as colorless crystals (41.1 mg, 59 % yield). The racemic *exo*-product was obtained as colorless crystals (26.7 mg, 38 % yield).

* Two additional peaks caused by the rotational barrier of the C_{Ar}-N bond, induced by the two ortho-Cl atoms (highlighted in blue).

(*endo*-3b)
¹H NMR (600 MHz, ACN-d_3) δ: 10.24 (s, 1H), 7.71 (d, *J* = 0.9 Hz, 1H), 7.67 (q, *J* = 2.2 Hz, 2H), 5.74 (dd, *J* = 4.4, 4.0 Hz, 1H), 4.32 (dd, *J* = 15.0, 8.0 Hz, 1H), 3.84 (s, 3H), 3.83 (dd, *J* = 4.5, 1.0 Hz, 1H), 3.45 (ddd, *J* = 11.0, 10.1, 7.9 Hz, 1H), 3.33 (ddd, *J* = 10.1, 4.0, 1.0 Hz, 1H), 3.16 (ddd, *J* = 15.1, 11.0, 1.1 Hz, 1H), 2.35 (d, *J* = 0.9 Hz, 3H).
¹³C NMR (151 MHz, ACN-d_3) δ: 192.56, 178.23, 175.31, 160.64, 139.83, 137.24, 136.25, 135.73, 135.19, 133.81, 131.25, 130.91*, 130.08, 129.81*, 128.88, 62.63, 62.38, 48.01, 39.14, 22.55, 16.08.
LC-HRMS: (refer to **Figure S91**, **Figure S92** and **Table S4**)
FTIR (neat) cm^{-1}: 3510 (w, br), 3368 (w, br), 2953 (w, br), 2926 (w, br), 1722 (s) 1678 (s), 1599 (w), 1572 (m), 1556 (m), 1470 (m), 1421 (w), 1389 (m), 1369 (m), 1288 (m), 1265 (m), 1240 (w), 1198 (m), 1180 (m), 1041 (m), 995 (m), 937 (w), 878 (m), 858 (m), 820 (m), 806 (m), 741 (m),698 (w), 683 (w), 650 (w).
Melting Point: 153 °C
Elemental Analysis: calculated for $C_{21}H_{16}Cl_3NO_5$: C: 53.81, H: 3.44, N: 2.99 − found: C: 53.46 H: 2.80 N: 2.97.

(*exo*-3b)
¹H NMR (600 MHz, ACN-d_3) δ: 10.14 (s, 1H), 7.67 (d, *J* = 0.9 Hz, 1H), 7.58 (d, *J* = 2.2 Hz, 1H), 7.41 (d, *J* = 2.2 Hz, 1H), 5.70 (dd, *J* = 3.7, 2.5 Hz, 1H), 4.37 (dd, *J* = 15.3, 1.9 Hz, 1H), 3.87 (dd, *J* = 9.0, 2.6 Hz, 1H), 3.79 (d, *J* = 3.7 Hz, 1H), 3.77 (s, 3H), 3.76 – 3.72 (m, 1H), 3.30 (ddd, *J* = 15.4, 7.2, 1.2 Hz, 1H), 2.30 (t, *J* = 0.8 Hz, 3H).
¹³C NMR (151 MHz, ACN-d_3) δ: 192.45, 178.09, 175.44, 161.48, 138.99, 137.30, 136.17, 135.46, 135.08, 132.48, 131.63*, 131.22, 129.81*, 129.73, 128.29, 63.46, 62.45, 48.82, 39.43, 23.21, 16.06.
LC-HRMS: (refer to **Figure S93**, **Figure S94** and **Table S5**.)
FTIR (neat) cm^{-1}: 3492 (w, br), 3347 (w, br), 2930 (w, br), 2856 (w, br), 1716 (s), 1601 (w), 1573 (m), 1556 (m), 1470 (s), 1371 (m), 1290 (m), 1178 (s, br), 1038 (m), 1014 (m), 997 (m), 895 (m), 858 (m), 821 (s), 804 (m), 758 (m), 702 (m), 667 (2), 642 (2), 605 (w).
Melting Point: 161 °C
Elemental Analysis: calculated for $C_{21}H_{16}Cl_3NO_5$: C: 53.81, H: 3.44, N: 2.99 − found: C: 53.85 H: 3.75 N: 2.95.

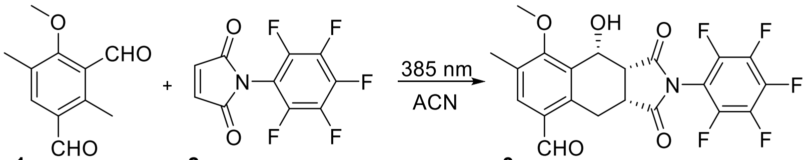

(3a*R*,9*R*,9a*R*)-9-hydroxy-8-methoxy-7-methyl-1,3-dioxo-2-(perfluorophenyl)-2,3,3a,4,9,9a-hexahydro-1*H*-benzo[*f*]isoindole-5-carbaldehyde (3c): 4-Methoxy-2,5-dimethylisophthal-aldehyde (**1a**, 28.8 mg, 0.15 mmol, 1.00 eq) and 1-(perfluorophenyl)-*1H*-pyrrole-2,5-dione (**2c**, 40.26 mg, 0.153 mmol 1.02 eq) were dissolved in 15 mL dry acetonitrile (20 mL crimp vial, 10 mmol L^{-1}). The solution was degassed by passing through N_2 for 15 min and irradiated for 40 min (385 nm, 0.75 A, 2 cm distance). Afterwards, the solvent was removed under reduced pressure and the crude product was purified *via* flash chromatography (isocratic, DCM:MeOH 99:1 *v/v*). The racemic *endo*-product was obtained as colorless solid (67.16 mg, 99 % yield).

¹H NMR (600 MHz, ACN-d_3) δ: 7.35 (s, 1H), 5.36 (t, *J* = 4.2 Hz, 1H), 5.09 (td, *J* = 7.7, 1.2 Hz, 1H), 3.99 (dd, *J* = 8.6, 2.7 Hz, 1H), 3.95 (d, *J* = 7.3 Hz, 1H), 3.88 (dd, *J* = 8.6, 7.7 Hz, 1H), 3.78 (ddd, *J* = 5.6, 2.8, 1.0 Hz, 1H), 3.74 (dd, *J* = 8.7, 5.7 Hz, 1H), 3.73 (s, 3H), 3.57 – 3.45 (m, 2H), 3.23 (dd, *J* = 4.3, 0.9 Hz, 1H), 3.08 (ddd, *J* = 8.6, 4.0, 0.9 Hz, 1H), 2.29 (s, 3H), 1.12 (t, *J* = 7.2 Hz, 3H).
¹³C NMR (151 MHz, ACN-d_3) δ: 192.60, 178.24, 175.09, 160.66, 145.50-145.27 (m), 144.12-143.59 (m), 142.45-141.99 (m), 140.17-139.76 (m), 139.50, 138.48-138.14 (m), 135.49, 133.44, 131.19, 130.92, 62.63, 62.60, 48.25, 38.65, 22.40, 16.09.
¹⁹F NMR (151 MHz, ACN-d_3) δ: -143.73 (dtd, *J* = 21.5, 6.3, 1.9 Hz), -144.63 (dtd, *J* = 21.5, 6.5, 1.9 Hz), -153.64 (t, *J* = 20.5 Hz), -163.23 (dtd, *J* = 56.6, 21.1, 6.5 Hz).
LC-HRMS: (refer to **Figure S95**, **Scheme S7** and **Table S6**)
FTIR (neat) cm^{-1}: 3520 (w, br), 3435 (w, br), 2943 (w, br), 2858 (w, br), 1795 (w), 1726 (s), 1682 (m), 1601 (w), 1572 (w), 1518 (s), 1479 (m), 1445 (m), 1414 (w), 1367 (m), 1292 (m), 1236 (m), 1177 (m), 1142 (m),1119 (m), 1096 (m), 1076 (m), 1036 (m), 1020 (m), 989 (s), 959 (m), 941 (w), 897 (w), 868 (w), 829 (w), 762 (m), 737 (w), 690 (w), 631 (w).
Melting Point: 189 °C (partial decomposition)

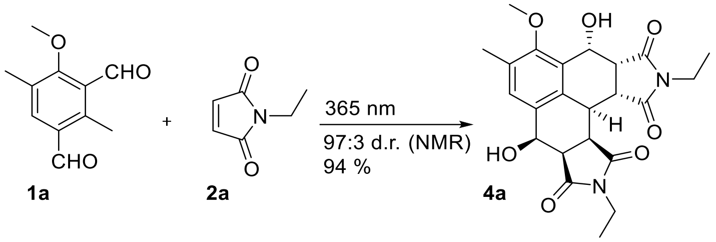

3a*R*,4*R*,8*R*,8a*R*,11a*R*,11b*R*,11c*R*)-2,10-diethyl-4,8-dihydroxy-5-methoxy-6-methyl-3a,8,8a,11a,11b,11c-hexahydro-1*H*-pyrrolo[3',4':2,3]naphtho[1,8-*ef*]isoindole-1,3,9,11(2*H*,4*H*,10*H*)-tetraone (4a): 4-Methoxy-2,5-dimethylisophthalaldehyde (**1a**, 28.8 mg, 0.15 mmol, 1.00 eq) and *N*-ethyl maleimide (**2a**, 42.23 mg, 0.338 mmol 2.25 eq) were dissolved in 15 mL dry acetonitrile (20 mL crimp vial, 10 mmol L^{-1}). The solution was degassed by passing through N_2 for 15 min and irradiated for 70 min (365 nm, 0.75 A, 2 cm distance). Subsequently, the solvent was removed under reduced pressure and the crude product was purified *via* flash chromatography (gradient, DCM:MeOH 99:1-95-5 *v/v*). The racemic *endo*-product **4a** was obtained as colorless amorph solid (62.4 mg, 94 % yield).

^{1}H NMR (600 MHz, DMSO-d_6) δ: 7.21 (s, 1H), 5.63 (d, *J* = 5.3 Hz, 1H), 5.41 – 5.28 (m, 1H), 5.20 (t, *J* = 4.1 Hz, 1H), 4.88 (td, *J* = 5.5, 2.6 Hz, 1H), 3.97 (dd, *J* = 8.5, 2.8 Hz, 1H), 3.65 (s, 3H), 3.56 – 3.51 (m, 2H), 3.50 – 3.38 (m, 3H), 3.11 – 3.00 (m, 3H), 2.20 (s, 3H), 1.07 (t, *J* = 7.2 Hz, 3H), 0.51 (t, *J* = 7.1 Hz, 3H).
^{13}C NMR (151 MHz, DMSO-d_6) δ: 178.96, 177.71, 177.21, 175.02, 153.88, 133.18, 129.60, 128.67, 128.32, 125.79, 65.88, 61.50, 59.34, 45.29, 45.27, 45.07, 39.15, 32.80, 32.21, 27.63, 15.78, 12.71, 12.29.
LC-HRMS: refer to **Figure S96**, **Scheme S8** and **Table S7**
FTIR (neat) cm^{-1}: 3395 (m, br), 2981 (w), 2952 (w), 2938 (w), 1764 (w), 1695 (s), 1677 (s), 1580 (w), 1441 (m), 1405 (m), 1377 (m), 1344 (m), 1315 (m), 1296 (m), 1257 (w), 1220 (m), 1134 (m), 1099 (m), 1082 (m), 1046 (m), 1017 (m), 985 (m), 960 (w), 926 (m), 917 (m), 909 (m), 894 (w), 874 (m), 849 (m), 817 (w), 793 (m), 783 (m), 759 (m), 721 (w), 714 (w), 692 (m), 681 (w), 671 (m), 657 (m), 618 (w), 605 (m), 592 (m), 580 (m).
Melting Point: Decomposition above 240 °C.
Elemental Analysis: calculated for $C_{23}H_{26}N_2O_7$: C: 62.43, H: 5.92, N: 6.33 − found: C: 61.95 , H: 5.19 , N: 6.35.

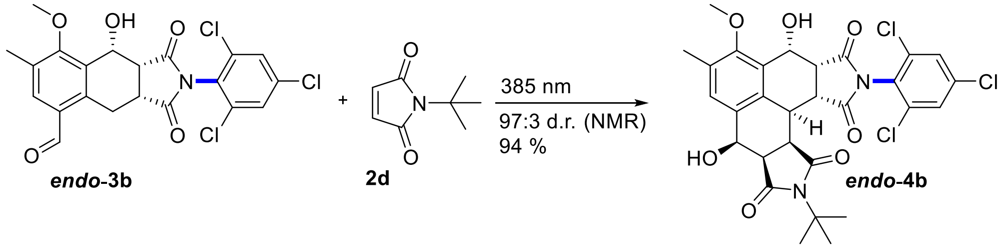

(3a*R*,4*R*,8*R*,8a*R*,11a*R*,11b*R*,11c*R*)-10-(tert-butyl)-4,8-dihydroxy-5-methoxy-6-methyl-2-(2,4,6-trichlorophenyl)-3a,8,8a,11a,11b,11c-hexahydro-1*H*-pyrrolo[3',4':2,3]naphtho[1,8-*ef*]isoindole-1,3,9,11(2*H*,4*H*,10*H*)-tetraone (*endo*-4b): (3a*R*,9*R*,9a*R*)-9-Hydroxy-8-methoxy-7-methyl-1,3-dioxo-2-(2,4,6-trichlorophenyl)-2,3,3a,4,9,9a-hexahydro-1*H*-benzo[*f*]isoindole-5-carbaldehyde (***endo*-3b**, 28.1 mg, 0.06 mmol, 1.00 eq) and *N*-tertbutyl maleimide (**2d**, 11.5 mg, 0.075 mmol 1.25 eq) were dissolved in 6 mL dry acetonitrile (10 mL crimp vial, 10 mmol L^{-1}). The solution was degassed by passing through N_2 for 15 min and irradiated for 50 min (365 nm, 0.75 A, 2 cm distance). Afterwards, the solvent was removed under reduced pressure to approximately 1 mL. Then the product was crystallized from the solution overnight at 7 °C, filtered and the solid washed with hexanes. The racemic *endo*-product **4b** was obtained as colorless crystal needles (35 mg, 95 % yield). * Two additional peaks caused by the rotational barrier of the C_{Ar}-N bond, caused by the two *ortho*-Cl atoms (highlighted in blue).

^{1}H NMR (600 MHz, DMSO-d_6) δ: 7.97 (d, *J* = 2.2 Hz, 1H), 7.90 (d, *J* = 2.3 Hz, 1H), 7.28 (s, 1H), 5.68 (t, *J* = 4.7 Hz, 2H), 5.41 – 5.31 (m, 1H), 4.89 (q, *J* = 4.5, 3.7 Hz, 1H), 4.28 (dd, *J* = 9.1, 2.9 Hz, 1H), 3.69 (s, 3H), 3.62 (d, *J* = 3.4 Hz, 1H), 3.40 – 3.28 (m, 3H), 2.25 (s, 3H), 1.10 (s, 9H).
^{13}C NMR (151 MHz, DMSO-d_6) δ: 178.53, 176.51, 175.83, 174.68, 153.85, 135.56, 135.48*, 134.47, 133.40, 129.17, 129.12, 128.68*, 128.62, 128.50, 127.91, 125.94, 65.82, 61.63, 59.06, 56.49, 45.95, 45.37, 44.59, 40.36, 28.20, 27.42, 15.80.
LC-HRMS: refer to **Figure S98**, **Scheme S9** and **Table S9**

FTIR (neat) cm^{-1}: 3495 (w, br), 3404 (w, br), 2926 (w, br), 2854 (w), 1798 (w), 1730 (s), 1715 (s), 1576 (w), 1556 (w), 1518 (s), 1470 (m), 1367 (m), 1319 (m), 1300 (m), 1267 (w), 1236 (m), 1198 (m), 1169 (m), 1144 (m), 1113 (m), 1080 (m), 1057 (w), 1009 (m), 993 (s), 957 (w), 872 (m), 854 (m), 820 (m), 806 (m), 793 (m), 764 (m), 710 (w),694 (w), 636 (m).
Melting Point: Decomposition above 240 °C.
Elemental Analysis: calculated for $C_{29}H_{27}Cl_3N_2O_7$: C; 56.01, H: 4.38, N: 4.50 − found: C: 55.82, H: 3.87, N: 4.52.

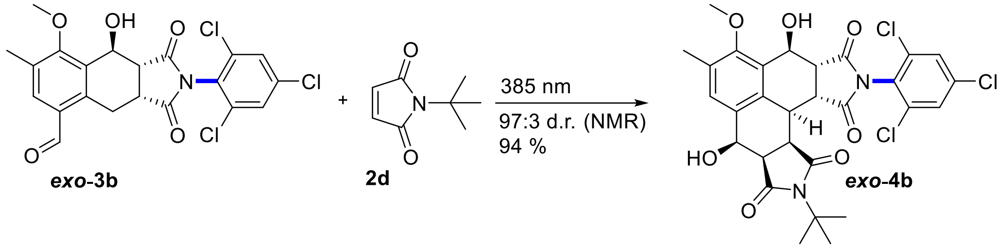

(3a*R*,4*S*,8*R*,8a*R*,11a*R*,11b*R*,11c*R*)-10-(tert-butyl)-4,8-dihydroxy-5-methoxy-6-methyl-2-(2,4,6-trichlorophenyl)-3a,8,8a,11a,11b,11c-hexahydro-1*H*-pyrrolo[3',4':2,3]naphtho[1,8-*ef*]isoindole-1,3,9,11(2*H*,4*H*,10*H*)-tetraone (*exo*-4b): (3a*R*,9*R*,9a*R*)-9-Hydroxy-8-methoxy-7-methyl-1,3-dioxo-2-(2,4,6-trichlorophenyl)-2,3,3a,4,9,9a-hexahydro-1*H*-benzo[*f*]isoindole-5-carbaldehyde (***exo*-3b**, 23.4 mg, 0.05 mmol, 1.00 eq) and *N*-tertbutyl maleimide (**2d**, 9.6 mg, 0.063 mmol 1.25 eq) were dissolved in 5 mL dry acetonitrile (10 mL crimp vial, 10 mmol L^{-1}). The solution was degassed by passing through N_2 for 15 min and irradiated for 60 min (365 nm, 0.75 A, 2 cm distance). Then the product was crystallized from the solution overnight at 7 °C, filtered and the solid washed with hexanes. Afterwards, the solvent was removed under reduced pressure and the crude product was purified *via* flash chromatography (gradient, DCM:MeOH 99:1-95-5 *v/v*). The racemic *exo*-product **4b** was obtained as colorless amorph solid (26.9 mg, 87 % yield).
* Two additional peaks caused by the rotational barrier of the C_{Ar}-N bond, caused by the two *ortho*-Cl atoms (highlighted in blue).

^{1}H NMR (600 MHz, DMSO-*d*$_6$) δ: 7.96 (d, *J* = 2.2 Hz, 1H), 7.90 (d, *J* = 2.2 Hz, 1H), 7.29 (s, 1H), 5.42 (dd, *J* = 5.5, 2.4 Hz, 1H), 5.35 (d, *J* = 6.5 Hz, 1H), 5.09 (d, *J* = 5.5 Hz, 1H), 4.76 (t, *J* = 5.9 Hz, 1H), 4.46 (dd, *J* = 8.3, 5.9 Hz, 1H), 4.02 (dd, *J* = 8.3, 2.4 Hz, 1H), 3.73 (s, 3H), 3.42 – 3.36 (m, 2H), 3.12 (t, *J* = 5.5 Hz, 1H), 2.24 (s, 3H), 1.10 (s, 9H).
^{13}C NMR (151 MHz, DMSO-*d*$_6$) δ: 177.59, 176.83, 176.57, 174.01, 155.46, 135.91, 134.51, 134.43*, 133.94, 129.04, 128.91, 128.90, 127.05, 126.57, 125.85, 125.83*, 66.40, 61.19, 56.94, 56.85, 47.79, 44.77, 42.77, 36.55, 33.25, 27.36, 15.72.
LC-HRMS: refer to **Figure S99** and **Table S10**
FTIR (neat) cm^{-1}:3491 (m), 3072 (w), 2978 (w, br), 2934 (m, br), 1798 (w), 1724 (s), 1680 (s), 1572 (m), 1556 (m), 1470 (m), 1423 (m), 1410 (m), 1360 (m), 1348 (m), 1335 (m), 1317 (m), 1302 (m), 1265 (m), 1242 (m), 1232 (s), 1194 (m), 1157 (s), 1111 (m), 1099 (m), 1076 (m),1055 (m), 1020 (m), 1007 (m), 883 (w), 895 (w), 964 (w), 941 (w), 918 (w), 868 (m), 837 (m), 820 (m), 806 (m), 783 (w), 750 (m), 737 (m), 700 (m), 679 (m), 652 (m), 635 (m), 615(w).
Melting Point: Decomposition above 240 °C.
Elemental Analysis: calculated for $C_{29}H_{27}Cl_3N_2O_7$: C; 56.01, H: 4.38, N: 4.50 − found: C: 55.10, H: 3.85, N: 4.56.

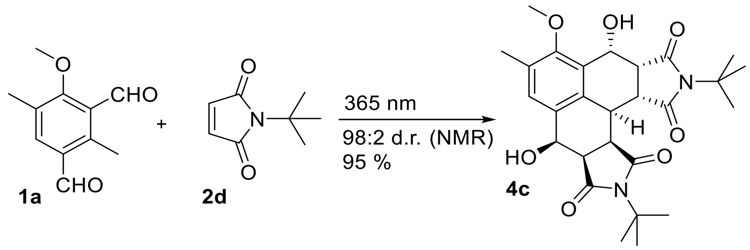

(3a*R*,4*R*,8*R*,8a*R*,11a*R*,11b*R*,11c*R*)-2,10-di-tert-butyl-4,8-dihydroxy-5-methoxy-6-methyl-3a,8,8a,11a,11b,11c-hexahydro-1*H*-pyrrolo[3',4':2,3]naphtho[1,8-*ef*]isoindole-1,3,9,11-(2*H*,4*H*,10*H*)-tetraone (4c): 4-Methoxy-2,5-dimethylisophthalaldehyde (**1a**, 19.2 mg, 0.10 mmol, 1.00 eq) and *N*-ethyl maleimide (**2d**, 34.5 mg, 0.225 mmol 2.25 eq) were dissolved in 10 mL dry acetonitrile (20 mL crimp vial, 10 mmol L^{-1}). The solution was degassed by passing through N_2 for 15 min and irradiated for 70 min (365 nm, 0.75 A, 2 cm distance). Next, the solvent was removed under reduced pressure to approximately 1 mL. Then the product was crystallized from the solution overnight at 7 °C, filtered and the solid washed with hexanes. The racemic *endo*-product 4c was obtained as colorless crystal plates (34.5 mg, 98 % yield).

^{1}H NMR (600 MHz, DMSO-d_6) δ: 7.24 (s, 1H), 5.59 (d, J = 5.2 Hz, 1H), 5.33 (dd, J = 4.2, 1.0 Hz, 1H), 5.18 (t, J = 4.0 Hz, 1H), 4.84 (ddd, J = 7.7, 5.2, 1.1 Hz, 1H), 3.76 (dd, J = 8.8, 2.2 Hz, 1H), 3.64 (s, 3H), 3.52 – 3.44 (m, 1H), 3.33 (s, 2H), 3.20 (dd, J = 8.5, 5.0 Hz, 1H), 2.83 (ddd, J = 8.8, 3.9, 1.0 Hz, 1H), 2.22 (s, 3H), 1.52 (s, 9H), 1.05 (s, 9H).
^{13}C NMR (151 MHz, DMSO-d_6) δ: 179.92, 178.28, 178.10, 175.91, 153.90, 133.34, 129.44, 128.64, 128.19, 125.50, 66.08, 61.49, 59.60, 56.86, 56.32, 45.42, 45.28, 44.75, 39.46, 28.10, 27.79, 27.31, 15.77.
LC-HRMS: refer to **Figure S100**, **Scheme S10** and **Table S11**
FTIR (neat) cm^{-1}: 3350 (m, br), 2978 (m), 2935 (m, br), 1767 (w), 1690 (s), 1581 (w), 1470 (m), 1398 (w), 1354 (s), 1335 (m), 1292 (m), 1265 (m), 1231 (m), 1204 (m), 1151 (s), 1117 (m), 1067 (m), 1041 (m), 1020 (m), 1001(m), 949 (m), 930 (m), 910 (m), 893 (w), 870 (w), 839 (m), 812 (w), 791 (m), 771 (m), 731 (w), 710 (w), 667 (m), 656 (m).
Melting Point: >260 °C (decomposition)
Elemental Analysis: calculated for $C_{27}H_{34}N_2O_7$: C; 65.04, H: 6.87, N: 5.62 − found: C: 64.34, H: 6.04, N: 5.77.

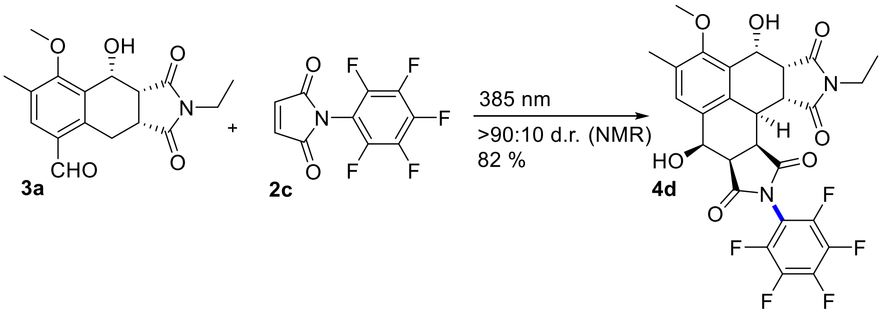

(3a*R*,4*R*,8*R*,8a*R*,11a*R*,11b*S*,11c*R*)-2-ethyl-4,8-dihydroxy-5-methoxy-6-methyl-10-(per-fluorophenyl)-3a,8,8a,11a,11b,11c-hexahydro-1*H*-pyrrolo[3',4':2,3]naphtho[1,8-*ef*]iso-indole-1,3,9,11(2*H*,4*H*,10*H*)-tetraone (4d): (3a*R*,9*R*,9a*R*)-2-Ethyl-9-hydroxy-8-methoxy-7-methyl-1,3-dioxo-2,3,3a,4,9,9a-hexahydro-1*H*-benzo[*f*]isoindole-5-carbaldehyde (***exo*-3a**, 23.8 mg, 0.075 mmol, 1.00 eq) and 1-(perfluorophenyl)-*1H*-pyrrole-2,5-dione (**2c**, 24.7 mg, 0.94 mmol 1.25 eq) were dissolved in 7.5 mL dry acetonitrile (20 mL crimp vial, 10 mmol L^{-1}). The solution was degassed by passing through N_2 for 15 min and irradiated for 60 min (365 nm, 0.75 A, 2 cm distance). Then the product was crystallized from the solution overnight at 7 °C, filtered and the solid washed with hexanes. Subsequently, the solvent was removed under reduced pressure and the crude product was purified *via* flash chromatography (gradient, DCM:MeOH 99:1-95-5 *v/v*). The racemic *exo*-product **4d** was obtained as colorless amorph solid (39.2 mg, 82 % yield).
* the rotational barrier (highlighted in blue) of the C_{Ar}-N bond, caused by the two *ortho*-F atoms results in the splitting of the fluorine signals. Consequentially the carbon-signals of the two pentafluorophenyl groups are broadened and could not be assigned.

^{1}H NMR (600 MHz, ACN-d_3) δ: 7.35 (s, 1H), 5.36 (t, J = 4.2 Hz, 1H), 5.09 (td, J = 7.7, 1.2 Hz, 1H), 3.99 (dd, J = 8.6, 2.7 Hz, 1H), 3.95 (d, J = 7.3 Hz, 1H), 3.88 (dd, J = 8.6, 7.7 Hz, 1H), 3.78 (ddd, J = 5.6, 2.8, 1.0 Hz, 1H), 3.74 (dd, J = 8.7, 5.7 Hz, 1H), 3.73 (s, 3H), 3.57 – 3.45 (m, 2H), 3.23 (dd, J = 4.3, 0.9 Hz, 1H), 3.08 (ddd, J = 8.6, 4.0, 0.9 Hz, 1H), 2.29 (s, 3H), 1.12 (t, J = 7.2 Hz, 3H).
^{13}C NMR (151 MHz, ACN-d_3) δ: 179.85, 177.95, 176.48, 174.61, 156.07, 133.88, 131.08, 130.01, 129.61, 127.37, 67.49, 62.46, 61.26, 47.40, 47.14, 45.95, 40.01, 34.14, 29.24, 16.21, 13.13.
^{19}F NMR (565 MHz, ACN-d_3) δ: -144.51 (dtd, J = 21.5, 6.3, 2.1 Hz), -145.09 – -145.24 (m), -153.19 (t, J = 20.5 Hz), -163.09 (dtd, J = 34.7, 21.3, 6.6 Hz).
LC-HRMS: refer to **Figure S101** and **Table S12**
FTIR (neat) cm^{-1}: 3479 (w, br), 3308 (w, br), 2928 (w, br), 2853 (w), 1767 (m), 1755 (m), 1691 (s), 1524 (s), 1501 (m), 1462 (m), 1448 (m), 1408 (m), 1379 (w),1346 (m),1323 (m),1308 (w), 1286 (w), 1254 (m), 1225 (m), 1140 (m), 1109 (m), 1074 (m), 1003 (s), 964 (m), 943 (m), 905 (m), 862 (w), 845 (m), 827 (w), 795 (m), 750 (w), 735 (w), 717 (w), 692 (w), 636 (m).
Melting Point: Decomposition above 200 °C.

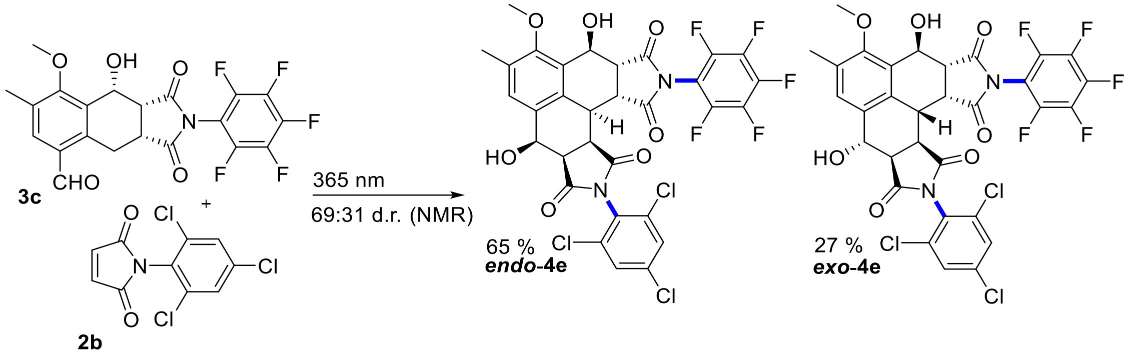

(3a*R*,4*S*,8*R*,8a*R*,11a*R*,11b*S*,11c*R*)-4,8-dihydroxy-5-methoxy-6-methyl-2-(perfluorophenyl)-10-(2,4,6-trichlorophenyl)-3a,8,8a,11a,11b,11c-hexahydro-1H-pyrrolo[3',4':2,3]naphtho-[1,8-*ef*]isoindole-1,3,9,11(2*H*,4*H*,10*H*)-tetraone (*endo*-4e) and (3a*R*,4*S*,8*S*,8a*R*,11a*R*,11b*R* ,11c*R*)-4,8-dihydroxy-5-methoxy-6-methyl-2-(perfluorophenyl)-10-(2,4,6-trichlorophenyl)-3a,8,8a,11a,11b,11c-hexahydro-1H-pyrrolo[3',4':2,3]naphtho-[1,8-*ef*]isoindole-1,3,9,11(2*H*, 4*H*,10*H*)-tetraone (*exo*-4e): (3a*R*,9*R*,9a*R*)-9-Hydroxy-8-methoxy-7-methyl-1,3-dioxo-2-(perfluorophenyl)-2,3,3a,4,9,9a-hexahydro-1*H*-benzo[*f*]isoindole-5-carbaldehyde (**3c,** 31.9 mg, 0.09 mmol, 1.00 eq) and 1-(2,4,6-trichlorophenyl)-1*H*-pyrrole-2,5-dione (**2b**, 24.2 mg, 0.94 mmol 1.25 eq) were dissolved in 7.0 mL dry acetonitrile (20 mL crimp vial, 10 mmol L^{-1}). The solution was degassed by passing through N_2 for 15 min and irradiated for 60 min (365 nm, 0.75 A, 2 cm distance). Subsequently, the solvent was removed under reduced pressure and the crude product was purified *via* flash chromatography (gradient, DCM:MeOH 99:1-95-7 *v/v*). The racemic *endo*-product **4e** (33.2 mg, 65 % yield) and racemic *exo*-product **4e** (13.8 mg, 27 % yield) were obtained as beige amorph solid.

* the rotational barrier (highlighted in blue) of the C_{Ar}-N bond, caused by the two *ortho*-F atoms results in the splitting of the fluorine signals. Consequentially the carbon-signals of the two pentafluorophenyl groups are broadened and could not be assigned.

(*endo*-4e)
^{1}H NMR (600 MHz, ACN-d_3) δ: 7.68 (q, *J* = 2.2 Hz, 2H), 7.53 (s, 1H), 5.59 (dd, *J* = 4.1, 3.6 Hz, 1H), 4.95 – 4.86 (m, 1H), 4.18 (d, *J* = 5.6 Hz, 1H), 4.01 (ddd, *J* = 9.1, 5.1, 0.5 Hz, 1H), 3.82 (dd, *J* = 8.8, 4.9 Hz, 1H), 3.80 (s, 3H), 3.73 (dd, *J* = 4.0, 1.1 Hz, 1H), 3.49 (t, *J* = 9.2 Hz, 1H), 3.45 (ddd, *J* = 9.1, 3.6, 1.1 Hz, 1H), 3.19 (t, *J* = 9.6 Hz, 1H), 2.36 (s, 3H).
^{13}C NMR (151 MHz, ACN-d_3) δ: 176.85, 176.13, 175.96, 174.79, 155.65, 137.49, 136.00, 135.90, 135.47, 131.01, 130.97, 130.27, 129.98, 129.95, 128.27, 127.75, 67.45, 62.57, 61.53, 48.86, 47.28, 46.51, 43.20, 29.75, 16.27.
^{19}F NMR (565 MHz, ACN-d_3) δ: -142.57 (dtd, *J* = 21.5, 6.4, 2.2 Hz), -145.02 (dtd, *J* = 21.8, 6.3, 2.1 Hz), -154.10 (t, *J* = 20.7 Hz), -163.40 (dtd, *J* = 27.1, 21.3, 6.3 Hz).
LC-HRMS: refer to **Figure S102**, **Figure S103**, **Scheme S11** and **Table S13**
FTIR (neat) cm^{-1}: 3493 (w, br), 3396 (w, br), 3078 (w), 2928 (w, br), 2854 (w), 1796 (w), 1730 (s), 1715 (s), 1574 (w), 1556 (w), 1518 (s), 1470 (m), 1367 (m), 1300 (m), 1236 (w), 1198 (m), 1169 (m), 1144 (m), 1113 (m), 1080 (m), 1057 (w), 1009 (m), 993 (s), 957 (w), 939 (w), 903 (w), 872 (w), 854 (m), 820 (m), 806 (w), 793 (w), 764 (m), 733 (w), 710 (w), 694 (w), 656 (w), 636 (m).
Melting Point: Decomposition above 200 °C.

(*exo*-4e)
^{1}H NMR (600 MHz, ACN-d_3) δ: 7.58 (d, *J* = 2.2 Hz, 1H), 7.44 (d, *J* = 2.2 Hz, 2H), 5.45 (t, *J* = 4.1 Hz, 1H), 5.12 (td, *J* = 7.8, 1.2 Hz, 1H), 4.48 (dd, *J* = 8.7, 2.3 Hz, 1H), 4.04 (d, *J* = 7.8 Hz, 1H), 3.93 (dd, *J* = 9.0, 7.8 Hz, 1H), 3.87 (ddd, *J* = 5.7, 2.3, 1.1 Hz, 1H), 3.83 (dd, *J* = 9.0, 5.7 Hz, 1H), 3.74 (s, 3H), 3.64 (dd, *J* = 4.2, 1.0 Hz, 1H), 3.42 (ddd, *J* = 8.7, 3.9, 0.9 Hz, 1H), 2.30 (s, 3H).
^{13}C NMR (151 MHz, ACN-d_3) δ: 177.07, 176.93, 175.44, 175.11, 156.22, 137.46, 135.39, 135.29, 134.62, 131.27, 129.82, 129.67, 129.60, 129.41, 128.06, 127.94, 67.67, 62.62, 60.98, 46.82, 46.50, 46.20, 40.55, 29.10, 16.18.
^{19}F NMR (565 MHz, ACN-d_3) δ: -141.64 (dtd, *J* = 21.6, 6.3, 2.3 Hz), -144.56 – -144.66 (m), -154.07 (t, *J* = 20.7 Hz), -163.48 (dtd, *J* = 89.3, 21.4, 6.4 Hz).
LC-HRMS: refer to **Figure S104**, **Figure S105**, **Table S14** and **Scheme S12**
FTIR (neat) cm^{-1}: 3495 (w, br), 3283 (w, br), 3080 (w), 2926 (w), 2858 (w), 1786 (m), 1718 (s), 1657 (w), 1574 (w), 1556 (w), 1522 (s), 1470 (s), 1369 (m), 1335 (m), 1321 (m), 1304 (m), 1263 (w), 1236 (m), 1173 (s), 1142 (m), 1113 (m), 1078 (m), 993 (s), 959 (m), 922 (m), 903 (m), 858 (m), 822 (m), 748 (w), 729 (w), 710 (w), 656 (m), 633 (m).
Melting Point: Decomposition above 200 °C.

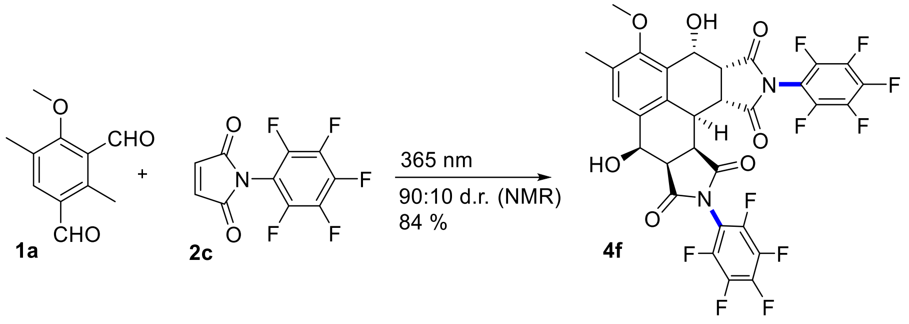

(3a*R*,4*R*,8*R*,8a*R*,11a*R*,11b*R*,11c*R*)-4,8-dihydroxy-5-methoxy-6-methyl-2,10-bis(perfluoro-phenyl)-3a,8,8a,11a,11b,11c-hexahydro-1*H*-pyrrolo[3',4':2,3]naphtho[1,8-*ef*]iso-indole-1,3, 9,11(2*H*,4*H*,10*H*)-tetraone (4f): 4-Methoxy-2,5-dimethylisophthalaldehyde (**1a**, 19.2 mg, 0.10 mmol, 1.00 eq) and 1-(perfluorophenyl)-1*H*-pyrrole-2,5-dione (**2c**, 59.2 mg, 0.205 mmol 2.25 eq) were dissolved in 10 mL dry acetonitrile (20 mL crimp vial, 10 mmol L^{-1}). The solution was degassed by passing through N_2 for 15 min and irradiated for 70 min (385 nm, 0.5 A, 2 cm distance) Subsequently, the solvent was removed under reduced pressure. The racemic *endo*-product **4f** was obtained as colorless amorph solid (60.4 mg, 84 % yield).
* the rotational barrier (highlighted in blue) of the C_{Ar}-N bond, caused by the two *ortho*-F atoms results in the splitting of the fluorine signals. Consequentially the carbon-signals of the two pentafluorophenyl groups are broadened and could not be assigned.

^{1}H NMR (600 MHz, ACN-d_3) δ: 7.39 (s, 1H), 5.46 (d, *J* = 3.8 Hz, 1H), 5.12 (d, *J* = 7.7 Hz, 1H), 4.41 (dd, *J* = 8.7, 2.6 Hz, 1H), 3.99 (s, 1H), 3.92 – 3.86 (m, 2H), 3.82 (dd, *J* = 8.7, 5.4 Hz, 1H), 3.75 (s, 3H), 3.61 (d, *J* = 17.0 Hz, 1H), 3.41 (dd, *J* = 8.7, 3.8 Hz, 1H), 2.31 (s, 3H).

^{13}C NMR (151 MHz, ACN-d_3) δ: 177.07, 176.50, 175.10, 174.44, 156.10, 133.91, 131.33, 129.48, 129.17, 127.59, 67.43, 62.53, 61.02, 47.23, 47.11, 46.90, 40.80, 29.24, 16.25.

^{19}F NMR (565 MHz, ACN-d3) δ: -141.61 – -141.80 (m), -144.38 – -144.57 (m), -144.59 – -144.75 (m), -145.16 (dt, J = 22.0, 6.6 Hz), -153.09 (t, J = 20.5 Hz), -154.07 (t, J = 20.6 Hz), -163.05 (dtd, J = 53.7, 21.1, 6.4 Hz), -163.49 (dtd, J = 84.9, 21.2, 6.4 Hz).

LC-HRMS: refer to **Figure S106**, **Table S15** and **Scheme S13**

FTIR (neat) cm^{-1}: 3356 (w, br), 3177 (w, br), 2924 (m), 2854 (w), 1796 (w), 1776 (w), 1724 (s), 1715 (s), 1659 (m), 1632 (m), 1583 (w), 1518 (s), 1468 (m), 1423 (w), 1412 (w), 1366 (m), 1329 (w), 1304 (m), 1265 (w), 1231 (w), 1186 (m), 1171 (m), 1138 (s), 1055 (w), 1032 (m), 1024 (m), 991 (s), 953 (m), 935 (m), 924 (m), 901 (m), 878 (m), 862 (m), 845 (m), 824 (w), 791 (w), 764 (m), 748 (m), 725 (m), 710 (w), 700 (m),669 (m), 656 (m), 635 (m).

Melting Point: Decomposition above 220 °C.

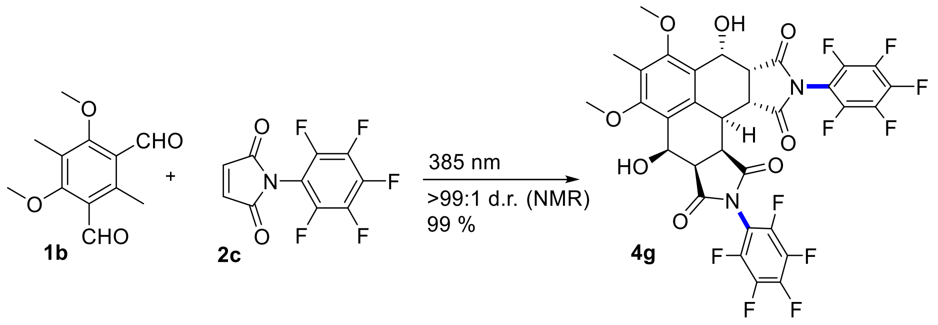

(3a*R*,4*R*,8*R*,8a*R*,11a*R*,11c*R*)-4,8-dihydroxy-5,7-dimethoxy-6-methyl-2,10-bis(perfluoro-phenyl)-3a,8,8a,11a,11b,11c-hexahydro-1*H*-pyrrolo[3',4':2,3]naphtho[1,8-*ef*]isoindole-1,3,9,11(2*H*,4*H*,10*H*)-tetraone (4g): 4,6-Dimethoxy-2,5-dimethylisophthalaldehyde (**1b**, 22.2 mg, 0.10 mmol, 1.00 eq) and 1-(perfluorophenyl)-1*H*-pyrrole-2,5-dione (**2c**, 53.9 mg, 0.205 mmol 2.05 eq) were dissolved in 10 mL dry acetonitrile (20 mL crimp vial, 10 mmol L^{-1}). The solution was degassed by passing through N_2 for 15 min and irradiated for 20 min (385 nm, 0.5 A, 2 cm distance) Subsequently, the solvent was removed under reduced pressure. The racemic *endo*-product **4g** was obtained as colorless amorph solid (74.1 mg, 99 % yield).

* the rotational barrier (highlighted in blue) of the C_{Ar}-N bond, caused by the two *ortho*-F atoms results in the splitting of the fluorine signals. Consequentially the carbon-signals of the two pentafluorophenyl groups are broadened, however could be assigned in this case due to better solubility of **4g** compared to **4d** and **4f**.

^{1}H NMR (600 MHz, DMSO-d_6) δ: 5.88 (dd, *J* = 4.0, 1.3 Hz, 1H), 5.81 (d, *J* = 4.7 Hz, 1H), 5.50 (t, *J* = 3.7 Hz, 1H), 5.23 (dd, *J* = 4.6, 3.8 Hz, 1H), 4.92 (dd, *J* = 10.9, 9.8 Hz, 1H), 4.28 (dd, *J* = 9.7, 7.4 Hz, 1H), 4.05 – 3.98 (m, 1H), 3.77 (s, 3H), 3.76 (s, 3H), 3.57 (ddd, *J* = 7.4, 3.9, 0.8 Hz, 1H), 3.42 (ddd, *J* = 9.8, 3.4, 1.3 Hz, 1H), 2.21 (d, *J* = 0.9 Hz, 3H).
^{13}C NMR (151 MHz, DMSO-d_6) δ: 176.89, 174.51, 174.14, 173.51, 156.66, 155.69, 144.20 – 143.50 (m), 142.92 – 141.77 (m), 141.08 – 140.17 (m), 138.28 (d, *J* = 71.9 Hz), 137.19 – 136.29 (m), 135.99, 131.43, 126.21, 125.47, 122.37, 107.55 – 106.96 (m), 61.72, 61.53, 61.30, 59.02, 47.86, 47.00, 38.52, 37.23, 29.47, 9.44.
^{19}F NMR (565 MHz, DMSO-d_6) δ: -139.63 (d, *J* = 23.6 Hz), -142.23 (d, *J* = 23.6 Hz), -144.19 (d, *J* = 23.9 Hz), -144.38 (d, *J* = 24.3 Hz), -151.69 (t, *J* = 23.0 Hz), -152.49 (t, *J* = 23.1 Hz), -162.03 (td, *J* = 23.5, 6.4 Hz), -162.24 (td, *J* = 23.4, 6.1 Hz), -162.50 (td, *J* = 23.6, 6.1 Hz), -162.70 (td, *J* = 23.3, 5.8 Hz).
LC-HRMS: refer to **Figure S107**, **Table S16** and **Scheme S14**
FTIR (neat) cm^{-1}: 3454 (w, br), 3287 (w, br), 2951 (w, br), 2851 (w), 1776 (m), 1726 (m), 1657 (m), 1583 (w), 1518 (s), 1460 (m), 1414 (w), 1367 (m), 1331 (m), 1304 (m), 1265 (w), 1231 (w), 1180 (m), 1146 (m), 1107 (s), 1082 (m), 1018 (m), 991 (s), 966 (m), 910 (m), 891 (m), 862 (w), 841 (w), 820 (w), 802 (m), 787 (w), 760 (w), 712 (w), 635 (w),609 (w).
Melting Point: Decomposition above 200 °C.

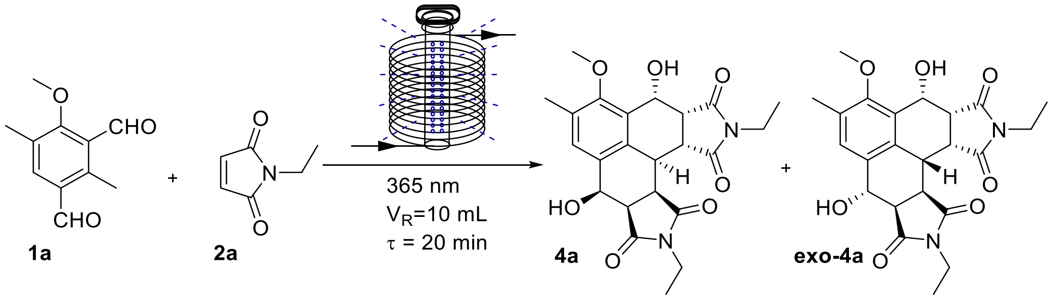

Photoflow reaction: (3a*R*,4*R*,8*R*,8a*R*,11a*R*,11b*R*,11c*R*)-2,10-diethyl-4,8-dihydroxy-5-methoxy-6-methyl-3a,8,8a,11a,11b,11c-hexahydro-1*H*-pyrrolo[3',4':2,3]naphtho[1,8-*ef*]iso-indole-1,3,9,11(2*H*,4*H*,10*H*)-tetraone (4a) and 3a*R*,4*R*,8*S*,8a*R*,11a*R*,11b*R*,11c*R*)-2,10-diethyl-4,8-dihydroxy-5-methoxy-6-methyl-3a,8,8a,11a,11b,11c-hexahydro-1*H*-pyrrolo-[3',4':2,3]naphtho[1,8-*ef*]isoindole-1,3,9,11(2*H*,4*H*,10*H*)-tetraone (*exo*-4a): 4-Methoxy-2,5-dimethylisophthalaldehyde (**1a**, 96.11 mg, 0.50 mmol, 1.00 eq) and *N*-ethyl maleimide (**2a**, 140.77 mg, 1.125 mmol 2.25 eq) were dissolved in 50 mL dry acetonitrile (10 mmol L^{-1}). The solution was degassed by passing through N_2 for 25 min and irradiated in a flow reactor at ambient temperature with a 10 W 365 nm LED (V_R 10 mL, flow rate 0.5 mL min^{-1}, retention time τ=20 min). The reactor output was collected, the solvent removed under reduced pressure to a volume of approximately 5-8 mL and left at ambient temperature for 24h. The obtained crystals were filtered and washed with *n*-hexanes. (203.54 mg, 92 % yield). The residual mother liquor was purified *via* preparative HPLC (gradient, DCM:MeOH 99:1-95-5 *v/v*). The racemic *exo*-product **4a** was obtained as colorless amorph solid (4.4 mg, 2 % yield).

(*exo*-4a)
^{1}H NMR (600 MHz, ACN-d_3) δ: 7.30 (s, 1H), 5.31 (dd, *J* = 12.2, 5.5 Hz, 1H), 4.92 (td, *J* = 8.0, 1.2 Hz, 1H), 4.75 (dd, *J* = 8.4, 3.6 Hz, 1H), 3.92 (d, *J* = 8.3 Hz, 1H), 3.75 (s, 3H), 3.61 – 3.49 (m, 5H), 3.39 (d, *J* = 12.2 Hz, 1H), 3.32 (dd, *J* = 9.5, 5.5 Hz, 1H), 3.09 (ddt, *J* = 33.1, 13.6, 6.9 Hz, 2H), 2.25 (s, 3H), 1.15 (t, *J* = 7.2 Hz, 3H), 0.40 (t, *J* = 7.2 Hz, 3H).
^{13}C NMR (151 MHz, ACN-d_3) δ 183.05, 178.31, 177.99, 177.25, 156.87, 133.82, 130.99, 129.80, 128.94, 127.05, 67.84, 62.30, 61.07, 46.36, 46.12, 41.59, 35.26, 34.03, 33.94, 29.62, 16.03, 13.23, 12.61.
LC-HRMS: refer to **Figure S97** and **Table S8**
FTIR (neat) cm^{-1}: 3449 (m), 3346 (w, br), 2982 (w), 2939 (w), 2858 (w), 1769 (m), 1676 (s), 1583 (w), 1441 (m), 1404 (m), 1387 (m), 1375 (m), 1342 (m), 1288 (m), 1261 (w), 1229 (s), 1205 (m), 1153 (w), 1136 (m), 1113 (s), 1090 (m), 1082 (m), 1068 (w), 1045 (m), 1024 (m), 997 (m), 976 (w), 941 (w), 920 (m), 891 (w), 868 (w), 852 (w), 829 (m), 811 (w), 779 (m), 752 (w), 729 (w), 696 (m), 685 (w), 662 (m), 646 (m), 619 (m), 607 (w).
Melting Point: Decomposition above 240 °C.
Elemental Analysis: calculated for $C_{23}H_{26}N_2O_7$: C: 62.43, H: 5.92, N: 6.33 − found: C: 62.19 , H: 4.99 , O: 6.35.

IV. Tunable Laser Studies

All laser experiments were conducted using the apparatus shown in **Figure S2**. The light source was an Opotek Opolette 355 OPO, producing 7 ns, 20 Hz pulses. The output beam was initially passed through a beam expander (-50 mm and 100 mm lens combination) to ensure it is large enough to uniformly irradiate the entire sample volume. The beam then passes through an electronic shutter and directed upwards using a UV silica right angle prism. Finally, the beam enters the sample, suspended in an aluminum block, from below. The laser energy deposited into the sample was measured above the aluminum block before and after experiments using a Coherent EnergyMax thermopile sensor (J-25MB-LE) to account for any power fluctuations during irradiation.

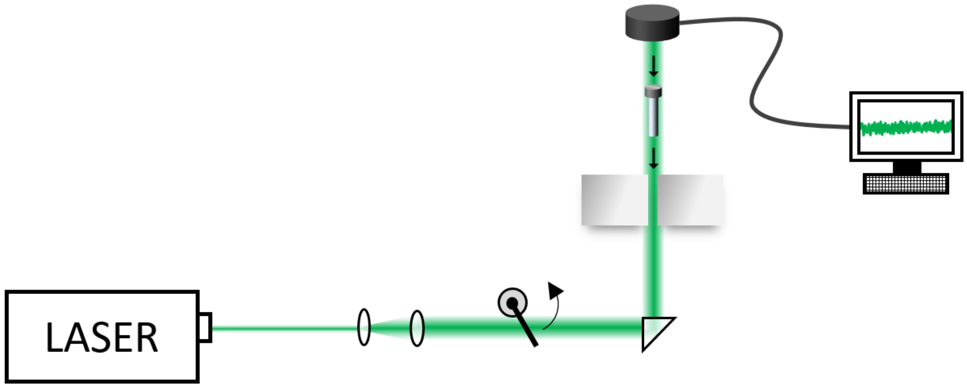

Figure S2 Schematic diagram of apparatus used for laser experiments.

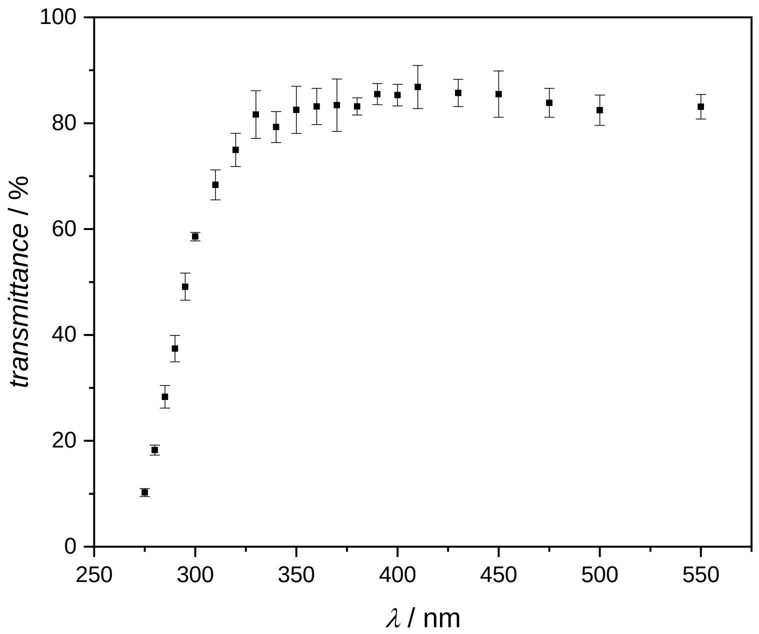

Figure S3 Transmittance of the bottom of the glass vials used in this study. The transmittance values shown and used here were obtained analogously to a method reported previously.[9] The glass vials were cut at a height of 3 mm.

For laser measurements, all samples were prepared in a 0.7 mL glass crimp vials (ID 6.2 mm) capped with a rubber/PTFE septum. The wavelength dependent glass transmittance, essential for quantitative measurements, is presented in **Figure S3**. Precise photons numbers were determined from the laser pulse energy using the following relation

$$N_p = \frac{E_{pulse}\lambda f_{rep} t}{hc\,[T_\lambda/100]} \quad (1)$$

where E_{pulse} is the measured pulse energy above the aluminum block, λ is the wavelength of the incident radiation, f_{rep} is the laser repetition rate, t is the irradiation time, h is Planck's constant, c is the speed of light and T_λ is the wavelength dependent glass transmission presented in **Figure S3**. Once an initial measurement is completed and the photon number is known, the required energies at other wavelengths can be found by rearranging Equation 1 to give

$$E_{pulse} = \frac{N_p hc[T_\lambda/100]}{\lambda f_{rep} t} \quad (2)$$

Reaction Quantum Yield Simulations: The reaction quantum yield was determined by measuring kinetics of the conversion over time and performing least squares fits of the experimentally determined conversions to the library of theoretical conversions simulated in Matlab®, (Eqns 3a-3d) at each time interval. For a range of different values of the quantum yield Φ, using the starting concentration $c(t_0)$, the molar absorptivity ε and the photon flux ψ, the conversion after each laser pulse (n) was determined using the previous time step (n-1) by

$$c_{SM}(t_n) = c_{SM}(t_{n-1}) - \psi[\phi_{prod} + \phi_{SP}]\ 10^{-\epsilon_{SM}c_{SM}(t_{n-1})L} \quad (3a)$$
$$c_B(t_n) = c_B(t_{n-1}) - \psi\ \phi_{prod}\ 10^{-\epsilon_{SM}c_{SM}(t_{n-1})L} \quad (3b)$$
$$c_{prod}(t_n) = c_{prod}(t_{n-1}) + \psi\ \phi_{prod}\ 10^{-\epsilon_{SM}c_{SM}(t_{n-1})L} - \psi\ \phi_{prod2}\ 10^{-\epsilon_{prod}c_{prod}(t_{n-1})L} \quad (3c)$$
$$c_{prod2}(t_n) = c_{prod2}(t_{n-1}) + \psi\ \phi_{prod2}\ 10^{-\epsilon_{prod}c_{prod}(t_{n-1})L} \quad (3d)$$

where the subscripts *SM*, *B*, *prod* and *prod2* refer to the starting material, reaction partner, product and side reaction from the product, respectively. Given the long irradiation times, diffusion was not taken into account in this simulation. A library of simulations, with quantum yield values varying between 0 and 1, was generated for each sample, using the parameters outlined in **Table S1**. Least squares fitting of the simulation library to the experimental data was then performed to determine the best fit simulation, and hence quantum yield.

Table S1 Quantum Yield Simulation Parameters

SIMULATION PARAMETER	1A + 2A → 3A	3A + 2A → 4A
EXCITATION WAVELENGTH (nm)	385	350
Ψ (PHOTONS/PULSE)	1.06×10^{15}	5.77×10^{14}
L (cm)	1.66	1.66
$C_{SM}(T_0)$ (mol L^{-1})	8.2×10^{-3}	3.4×10^{-3}
E_{SM} (L mol^{-1} cm^{-1})	9.67	101
REACTANT B EQUIVALENCE	2.2	1.4
E_B (L mol^{-1} cm^{-1})	0	57
E_{PROD} (L mol^{-1} cm^{-1})	0	3.49
**DETERMINED QUANTUM YIELD (%)**	**38 ± 7**	**8.8 ± 1.2**

Reaction Quantum Yield Measurements: The reaction quantum yield was determined from least squares fitting of experimental kinetic measurements to simulations. For kinetic measurements of the first cycloaddition, a stock solution was prepared with 7.83 mg of **1a** and 11.07 mg of **2a** in 5 mL of CD_3CN (8.2 mmol L^{-1}, 2.2 eq.). 0.5 mL of the stock solution was placed into a 0.7 mL crimp vial and sealed with a PTFE/rubber septum before being flushed with N_2 for 5 mins. After degassing, the samples were irradiated with 385 nm irradiation for the specified period of time with a constant photon flux of (2.8 ± 0.3) × 10^{16} photons cm^{-2} s^{-1}. After irradiation, ^{1}H-NMR spectroscopy was performed on the sample solutions and the conversions were calculated from the integrals specified in **Figure S87**.

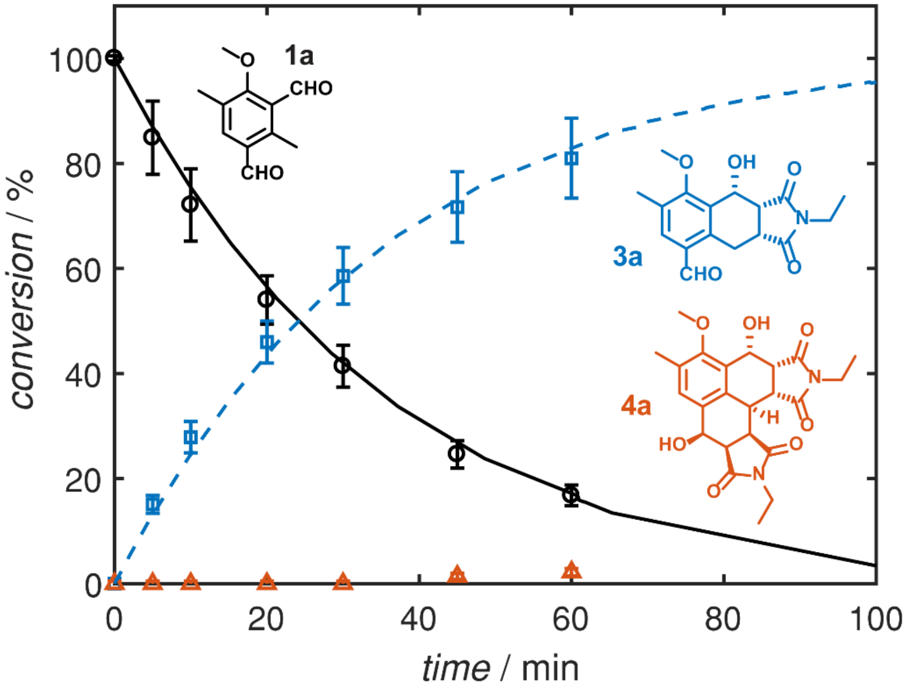

Figure S4 Experimental time-dependent conversions of **1a** and **2a** to form **3a** in CD_3CN and corresponding best-fit theoretical simulations used to determine reaction quantum yield.

For kinetic measurements of the second cycloaddition, a stock solution was prepared with 5.44 mg of **3a** and 2.93 mg of **2a** in 5 mL of CD_3CN (3.4 mmol L^{-1}, 1.4 eq.). 0.5 mL of the stock solution was placed into a 0.7 mL crimp vial and sealed with a PTFE/rubber septum before being flushed with N_2 for 5 mins. After degassing, the samples were irradiated with 350 nm irradiation for the specified period of time with a constant photon flux of $(2.94 \pm 0.05) \times 10^{16}$ photons cm^{-2} s^{-1}. After irradiation, ^{1}H-NMR spectroscopy was performed on the sample solutions and the conversions were calculated from the integrals specified in **Figure S88**.

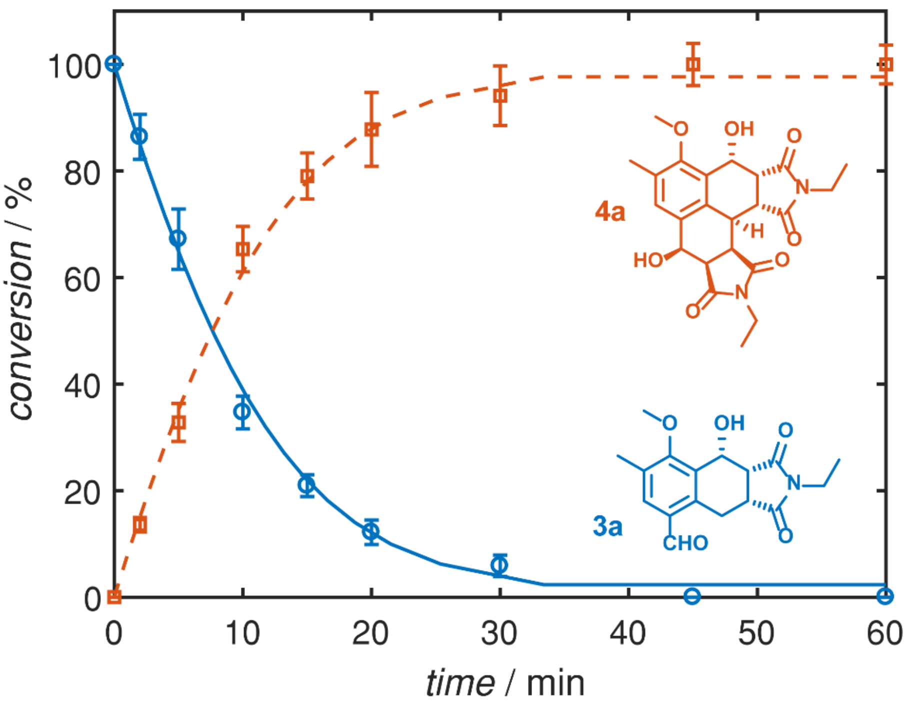

Figure S5 Experimental time-dependent conversions of **3a** and **2a** to form **4a** in CD_3CN and corresponding best-fit theoretical simulations used to determine reaction quantum yield.

Action Plot Measurements: For action plot measurements of the first cycloaddition (**1A + 2A → 3A**), a stock solution was prepared with 4.7 mg of **1a** and 6.9 mg of **2a** in 15 mL of acetonitrile (1.6 mmol L^{-1}, 2.2 eq.). 0.5 mL of the stock solution was placed into a 0.7 mL crimp vial and sealed with a PTFE/rubber septum before being flushed with N_2 for 5 mins. After degassing, the samples were irradiated for 20 minutes with a constant photon flux of (9.4 ± 0.2) × 10^{18} photons cm^{-2} s^{-1} at the specified wavelength. After irradiation the samples were evaporated and redispersed in 0.5 mL of CD_3CN. ^1H-NMR spectroscopy was performed on the sample solutions and the conversions were calculated from the integrals specified in **Figure S89**.

V. Procedures for the Transformations of HPDDs

In the following section, the transformations of HPDDs are described. These reactions were investigated to establish a viable route towards the PLYD neutral radicals, described in section V.

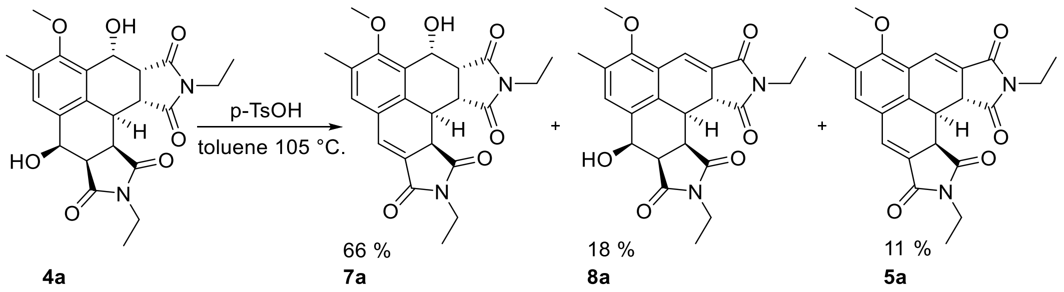

Synthesis of (11a*R*,11b*R*,11c*R*)-2,10-diethyl-5-methoxy-6-methyl-11b,11c-dihydro-1*H*-pyrrolo[3',4':2,3]naphtho[1,8-*ef*]isoindole-1,3,9,11(2*H*,10*H*,11a*H*)-tetraone (5a), (3a*R*,4*R*, 11a*R*,11b*S*,11c*R*)-2,10-diethyl-4-hydroxy-5-methoxy-6-methyl-3a,11a,11b,11c-tetrahydro-1*H*-pyrrolo[3',4':2,3]naphtho[1,8-*ef*]isoindole-1,3,9,11(2*H*,4*H*,10*H*)-tetraone (7a) and (3a*R*, 4*R*,11a*R*,11b*R*,11c*R*)-2,10-diethyl-4-hydroxy-7-methoxy-6-methyl-3a,11a,11b,11c-tetra-hydro-1*H*-pyrrolo[3',4':2,3]naphtho[1,8-*ef*]isoindole-1,3,9,11(2*H*,4*H*,10*H*)-tetraone (8a): (3a*R*,4*R*,8*R*,8a*R*,11a*R*,11b*R*,11c*R*)-2,10-Diethyl-4,8-dihydroxy-5-methoxy-6-methyl-3a,8,8a,11a, 11b,11c-hexahydro-1*H*-pyrrolo[3',4':2,3]naphtho[1,8-*ef*]iso-indole-1,3,9,11(2*H*,4*H*,10*H*)-tetraone (**4a,** 75 mg, 0.170 mmol ,1.00 eq) and *p*-TsOH monohydrate (3.22 mg, 17 µmol) were dissolved in 17 mL toluene and the solution was degassed by passing through N_2 for 20 min in a crimp vial. Then the vial was immersed into an oil bath and heated at 105 °C for 16 h. Afterwards the reaction solution was cooled to ambient temperature, the toluene was removed under reduced pressure and the residual product mixture of **5a**, **7a** and **8a** separated *via* preparative HPLC (gradient *n*-hexane: ethyl acetate 70:30-0:100 *v/v*). **7a** (47.5 mg, colorless solid, 66 % yield), **8a** (13.0 mg, colorless solid, 18 % yield) and **5a** (7.6 mg, beige solid, 11 % yield) were obtained.

(7a)
^{1}H NMR (600 MHz, ACN-*d*$_3$) δ: 7.31 (s, 1H), 7.29 (d, *J* = 2.6 Hz, 1H), 5.43 (dd, *J* = 3.8, 3.1 Hz, 1H), 3.78 (s, 3H), 3.71 (dd, *J* = 8.7, 4.3 Hz, 1H), 3.59 (qd, *J* = 7.3, 5.0 Hz, 2H), 3.56 – 3.44 (m, 3H), 3.37 (dd, *J* = 3.8, 1.0 Hz, 1H), 3.29 (dd, *J* = 15.4, 2.6 Hz, 1H), 3.07 (ddd, *J* = 8.7, 3.1, 1.0 Hz, 1H), 2.29 (s, 3H), 1.18 (t, *J* = 7.2 Hz, 3H), 1.12 (t, *J* = 7.2 Hz, 3H).
^{13}C NMR (151 MHz, ACN-*d*$_3$) δ: 179.92, 177.75, 174.93, 168.22, 157.94, 134.18, 133.62, 131.89, 131.24, 130.01, 129.23, 129.00, 62.58, 62.30, 46.54, 45.17, 41.31, 34.20, 34.04, 32.13, 15.95, 13.42, 13.13.
LC-HRMS: refer to **Figure S117** and **Table S24**
FTIR (neat) cm^{-1}: 3492 (w, br), 2982 (w), 2943 (w), 2853 (w), 1782 (w), 1761 (m), 1686 (s), 1664 (s), 1562 (m), 1443 (m), 1408 (m), 1396 (m), 1373 (m), 1350 (m), 1339 (m), 1329 (m), 1277 (m), 1261 (w), 1242 (m), 1217 (s), 1163 (m), 1140 (m), 1086 (w), 1067 (m), 1043 (m), 993 (m), 962 (w), 945 (m), 939 (m), 912 (m),847 (m), 831 (w), 797 (w), 783 (m), 746 (m), 700 (m), 669 (m), 656 (m), 638 (m).
Melting Point: Decomposition above 240 °C.
Elemental Analysis: calculated for $C_{23}H_{24}N_2O_6$: C: 65.08, H: 5.70, N: 6.60 − found: C: 65.30, H: 5.37, N: 6.42.

(8a)
^{1}H NMR (600 MHz, ACN-*d*$_3$) δ: 7.36 (s, 1H), 7.32 (d, *J* = 3.1 Hz, 1H), 5.08 (dd, *J* = 12.5, 3.1 Hz, 1H), 4.75 (ddd, *J* = 8.8, 7.5, 1.1 Hz, 1H), 4.22 (d, *J* = 8.9 Hz, 1H), 3.79 (dd, *J* = 8.9, 6.4 Hz, 1H), 3.74 (s, 3H), 3.62 (dd, *J* = 8.8, 7.6 Hz, 1H), 3.58 (q, *J* = 7.2 Hz, 2H), 3.42 (dd, *J* = 12.3, 5.8 Hz, 1H), 3.26 (q, *J* = 7.2 Hz, 2H), 2.25 (s, 3H), 1.18 (t, *J* = 7.2 Hz, 3H), 0.74 (t, *J* = 7.2 Hz, 3H).
^{13}C NMR (151 MHz, ACN-*d*$_3$) δ: 178.96, 178,92, 176.93, 168.98, 157.77, 134.67, 131.75, 131.61, 128.83, 128.20, 125.33, 122.43, 67.66, 63.03, 45.06, 42.73, 39.42, 34.13, 33.99, 33.56, 15.98, 13.41, 12.88.
LC-HRMS: refer to **Figure S118** and **Table S25**
FTIR (neat) cm^{-1}: 3458 (w, br),2937 (w), 2880 (w), 2851 (w), 1778 (m), 1763 (m), 1699 (s), 1560 (m), 1466 (m), 1439 (m), 1393 (m), 1375 (m), 1344 (m), 1333 (m), 1283 (w), 1258 (w), 1231 (m), 1209 (m), 1175 (m), 1151 (w), 1115 (w), 1092 (m), 1051 (s), 1022 (m), 995 (m), 980 (m), 941 (w), 918 (w), 874 (w), 841 (w), 833 (w), 802 (m), 752 (m), 737 (s), 681 (m), 658 (m), 642 (w), 631 (w).
Melting Point: Decomposition above 190 °C.
Elemental Analysis: calculated for $C_{23}H_{24}N_2O_6$: C: 65.08, H: 5.70, N: 6.60 − found: C: 65.35, H: 5.11, N: 6.34.

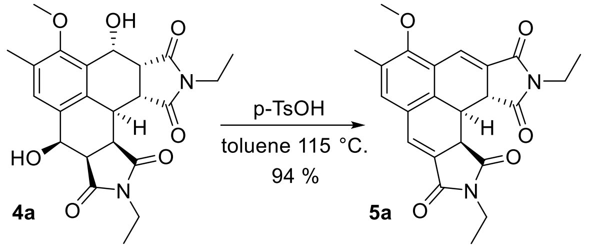

(11a*R*,11b*R*,11c*R*)-2,10-diethyl-5-methoxy-6-methyl-11b,11c-dihydro-1*H*-pyrrolo[3',4':2,3]-naphtho[1,8-*ef*]isoindole-1,3,9,11(2*H*,10*H*,11a*H*)-tetraone (5a): (3a*R*,4*R*,8*R*,8a*R*,11a*R*,11b*R*, 11c*R*)-2,10-Diethyl-4,8-dihydroxy-5-methoxy-6-methyl-3a,8,8a,11a,11b,11c-hexahydro-1*H*-pyrrolo[3',4':2,3]naphtho[1,8-*ef*]iso-indole-1,3,9,11(2*H*,4*H*,10*H*)-tetraone (**4a,** 75 mg, 0.170 mmol ,1.00 eq) and *p*-TsOH monohydrate (3.22 mg, 17 µmol, 10 mol%) were dissolved in 17 mL toluene and the solution was degassed by passing through N_2 for 20 min in a crimp vial. Then the vial was immersed into an oil bath and heated at 115 °C for 24 h. Afterwards the reaction solution was cooled to ambient temperature, the toluene was removed under reduced pressure and the residual crude product was isolated *via* flash chromatography (gradient *n*-hexane: ethyl acetate 70:30-25:85 *v/v*). **5a** was obtained as beige solid (64.7 mg, 94 % yield).

^{1}H NMR (600 MHz, ACN-d_3) δ 7.38 (d, *J* = 2.8 Hz, 1H), 7.21 (s, 1H), 7.13 (d, *J* = 2.8 Hz, 1H), 3.79 – 3.70 (m, 5H), 3.63 – 3.52 (m, 4H), 3.47 (t, *J* = 13.1 Hz, 1H), 2.24 (s, 3H), 1.22 – 1.12 (m, 6H).
^{13}C NMR (151 MHz, ACN-d_3) δ 174.59, 174.45, 168.28, 168.22, 158.98, 134.57, 134.50, 132.02, 130.62, 129.19, 128.71, 127.83, 126.90, 123.35, 62.44, 43.78, 43.52, 36.74, 34.12, 34.06, 15.76, 13.46[1].
[1] The two methyl groups (positions 8, 16, refer to **Figure S64**) are superimposed.
LC-HRMS: refer to **Figure S110** and **Table S18**
FTIR (neat) cm^{-1}: 2924 (w, br), 2876 (w), 2851 (w), 1776 (m), 1761 (m), 1697 (s, br), 1560 (m), 1466 (m), 1452 (m), 1439 (m), 1394 (m), 1373 (m), 1344 (m), 1333 (m), 1283 (w), 1258 (w), 1231 (m), 1209 (m), 1175 (m), 1151 (w), 1115 (w), 1092 (m), 1049 (s), 1020 (m), 995 (m), 980 (m), 939 (m), 918 (m), 874 (w), 841 (w), 833 (w), 802 (m), 754 (m), 737 (m), 681 (m), 658 (m), 642 (w), 631 (w), 604 (w).
Melting Point: Decomposition above 190 °C.
Elemental Analysis: calculated for $C_{23}H_{22}N_2O_5$: C: 67.97, H: 5.46, N: 6.89 − found: C: 67.54, H: 4.98, N: 6.91.

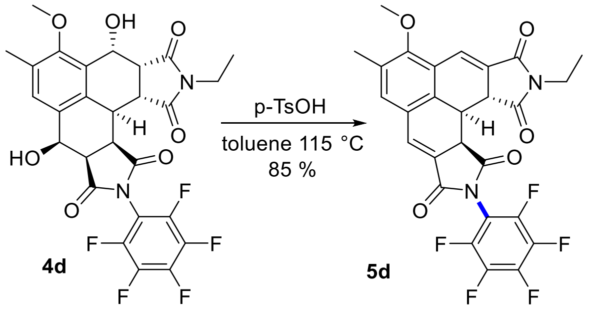

Synthesis of (11a*R*,11b*S*,11c*R*)-2-ethyl-5-methoxy-6-methyl-10-(perfluorophenyl)-11b,11c-dihydro-1*H*-pyrrolo[3',4':2,3]naphtho[1,8-*ef*]isoindole-1,3,9,11(2*H*,10*H*,11a*H*)-tetraone (5d): (3a*R*,4*R*,8*R*,8a*R*,11a*R*,11b*S*,11c*R*)-2-Ethyl-4,8-dihydroxy-5-methoxy-6-methyl-10-(per-fluorophenyl)-3a,8,8a,11a,11b,11c-hexahydro-1*H*-pyrrolo[3',4':2,3]naphtho[1,8-*ef*]iso-indole-1,3,9,11(2*H*,4*H*,10*H*)-tetraone (**4d**, 39 mg, 0.067 mmol ,1.00 eq) and *p*-TsOH monohydrate (1.28 mg, 7 µmol, 10 mol %) were dissolved in 6.8 mL toluene and the solution was degassed by passing through N_2 for 20 min in a crimp vial. Then the vial was immersed into an oil bath and heated at 115 °C for 24 h. Afterwards the reaction solution was cooled to ambient temperature, the toluene was removed under reduced pressure and the residual crude product was isolated *via* flash chromatography (gradient *n*-hexane: ethyl acetate 70:30-35:65 *v/v*). **5d** was obtained as yellowish solid (31 mg, 85 % yield).

^{1}H NMR (600 MHz, Chloroform-*d*) δ 7.65 (d, *J* = 2.6 Hz, 1H), 7.44 (d, *J* = 2.8 Hz, 1H), 7.22 (s, 1H), 4.16 (dd, *J* = 12.6, 2.8 Hz, 1H), 3.83 (s, 3H), 3.79 (t, *J* = 13.1 Hz, 1H), 3.76 – 3.65 (m, 3H), 2.33 (s, 3H), 1.25 (t, *J* = 7.2 Hz, 3H).
^{13}C NMR (151 MHz, Chloroform-*d*) δ 173.18, 170.90, 167.07, 164.26, 159.20, 144.82-144.41 (m), 143.18-142.76 (m), 141.54-141.19 (m), 139.0-138.68 (m), 137.26-137.00 (m), 134.47, 132.88, 131.88, 131.48, 128.58, 126.76, 126.38, 124.81, 124.12, 107.3-107.07 (m), 61.94, 43.51, 43.04, 36.36, 33.83, 15.70, 13.20.

^{19}F NMR (565 MHz, Chloroform-*d*) δ -141.45 (dt, *J* = 22.8, 6.8 Hz), -142.30 (dt, *J* = 22.8, 7.0 Hz), -150.95 (t, *J* = 21.3 Hz), -160.63 (td, *J* = 22.0, 6.7 Hz), -160.83 (td, *J* = 22.0, 6.7 Hz).
LC-HRMS: refer to **Figure S111**, **Figure S112**, **Table S19**, **Table S20** and **Scheme S15**
FTIR (neat) cm^{-1}: 2957 (w), 2924 (w), 2853 (w), 1794 (w), 1761 (m), 1732 (m), 1699 (s), 1666 (m), 1597 (w), 1560 (w), 1516 (s), 1479 (m), 1460 (w), 1439 (w), 1391 (m), 1373 (m), 1348 (m), 1290 (m), 1283 (m), 1263 (m), 1240 (m), 1213 (m), 1175 (m), 1142 (m), 1117 (m), 1092 (m), 1059 (m), 1014 (w), 1003 (m), 984 (s), 918 (m), 879 (w), 849 (m), 804 (m), 798 (m), 748 (w), 735 (m), 712 (w), 683 (m), 658 (w), 646 (m), 627 (m).
Melting Point: Decomposition above 200 °C.

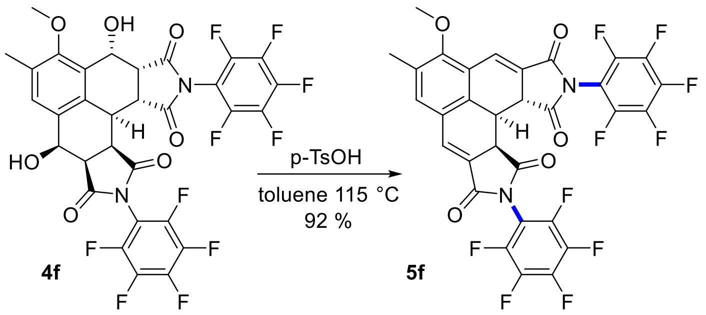

(11aR,11bR,11cR)-5-methoxy-6-methyl-2,10-bis(perfluorophenyl)-11b,11c-dihydro-1H-pyrrolo[3',4':2,3]naphtho[1,8-ef]isoindole-1,3,9,11(2H,10H,11aH)-tetraone (5f): (3a*R*,4*R*,8*R*, 8a*R*,11a*R*,11b*R*,11c*R*)-4,8-Dihydroxy-5-methoxy-6-methyl-2,10-bis(perfluoro-phenyl)-3a,8,8a, 11a,11b,11c-hexahydro-1*H*-pyrrolo[3',4':2,3]naphtho[1,8-*ef*]iso-indole-1,3,9,11(2*H*,4*H*,10*H*)-tetraone (**4f**, 50 mg, 0.070 mmol ,1.00 eq) and *p*-TsOH monohydrate (1.32 mg, 7 µmol, 10 mol %) were dissolved in 7 mL toluene and the solution was degassed by passing through N_2 for 20 min in a crimp vial. Then the vial was immersed into an oil bath and heated at 115 °C for 24 h. Afterwards the reaction solution was cooled to ambient temperature, the toluene was removed under reduced pressure and the residual crude product was isolated *via* flash chromatography (gradient *n*-hexane: ethyl acetate 70:30-35:65 *v/v*). **5f** was obtained as yellowish solid (44 mg, 92 % yield).

^{1}H NMR (600 MHz, Chloroform-*d*) δ 7.84 (d, *J* = 2.8 Hz, 1H), 7.47 (d, *J* = 2.8 Hz, 1H), 7.29 (s, 1H), 4.15 (dd, *J* = 13.0, 2.8 Hz, 1H), 4.11 (dd, *J* = 13.2, 2.8 Hz, 1H), 3.99 (t, *J* = 13.1 Hz, 1H), 3.86 (s, 3H), 2.36 (s, 3H).
^{13}C NMR (151 MHz, Chloroform-*d*) δ 170.70, 170.58, 164.19, 164.14, 159.69, 145.96 – 143.88 (m), 143.52 – 142.23 (m), 141.56 (t, *J* = 15.7 Hz), 138.94 (t, *J* = 11.9 Hz), 137.29 (t, *J* = 22.3 Hz), 135.41, 132.62, 132.39, 131.64, 127.56, 127.20, 126.35, 125.78, 125.20, 107.18 (t, *J* = 16.2 Hz), 62.31, 43.73, 43.59, 36.69, 15.90.
^{19}F NMR (565 MHz, Chloroform-*d*) δ -141.45 (dq, *J* = 22.4, 7.3 Hz), -142.39 (tt, *J* = 22.7, 6.6 Hz), -150.76 (dt, *J* = 29.0, 21.5 Hz), -160.47 (dtd, *J* = 44.3, 21.8, 6.7 Hz), -160.76 (tdd, *J* = 21.9, 14.8, 6.6 Hz).
LC-HRMS: refer to **Figure S113**and **Table S21**
FTIR (neat) cm^{-1}: 2970 (w), 2939 (w), 2881 (w), 1799 (w), 1728 (s), 1666 (m), 1556 (w), 1516 (s), 1474 (m), 1408 (w), 1387 (w), 1356 (m), 1294 (m), 1265 (m), 1240 (m), 1177 (m), 1142 (m), 1111 (s), 1082 (m), 1065 (w), 1011 (w), 987 (s), 922 (w), 883 (m), 864 (m), 831 (s), 795 (m), 733 (m), 708 (w), 642 (m), 623 (w).
Melting Point: Decomposition above 190 °C.

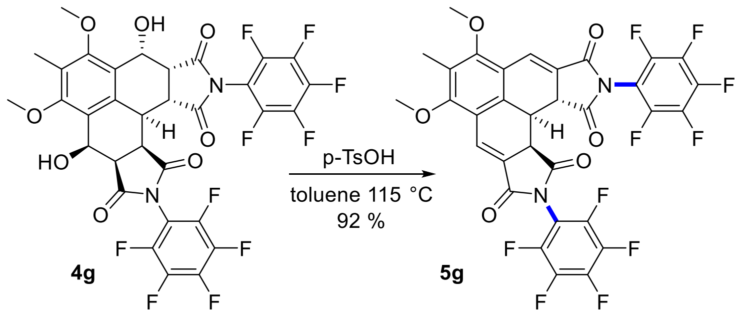

Synthesis of (11a*R*,11c*R*)-5,7-dimethoxy-6-methyl-2,10-bis(perfluorophenyl)-11b,11c-dihydro-1*H*-pyrrolo[3',4':2,3]naphtho-[1,8-*ef*]isoindole-1,3,9,11(2*H*,10*H*,11a*H*)-tetraone (5g): (3a*R*,4*R*,8*R*,8a*R*,11a*R*,11c*R*)-4,8-Dihydroxy-5,7-dimethoxy-6-methyl-2,10-bis(perfluoro-phenyl)-3a,8,8a,11a,11b,11c-hexahydro-1*H*-

pyrrolo[3',4':2,3]naphtho[1,8-*ef*]isoindole-1,3,9,11-(2*H*,4*H*,10*H*)-tetraone (**4g**, 70 mg, 0.094 mmol ,1.00 eq) and *p*-TsOH monohydrate (1.80 mg, 9 µmol, 10 mol %) were dissolved in 9.5 mL toluene and the solution was degassed by passing through N_2 for 20 min in a crimp vial. Then the vial was immersed into an oil bath and heated at 115 °C for 24 h. Afterwards the reaction solution was cooled to ambient temperature, the toluene was removed under reduced pressure and the residual crude product was isolated *via* flash chromatography (isocratic DCM:MeOH 98:2 *v/v*). **5g** was obtained as colorless solid (62 mg, 92 % yield).

^{1}H NMR (600 MHz, Chloroform-*d*) δ 7.79 (d, *J* = 2.7 Hz, 2H), 4.12 (dd, *J* = 13.2, 2.9 Hz, 2H), 4.00 – 3.92 (m, 1H), 3.86 (s, 6H), 2.29 (s, 3H).
^{13}C NMR (151 MHz, Chloroform-*d*) δ 170.72, 164.27, 161.05, 144.92-144.54, 143.43–142.81 (m), 141.69–141.45 (m), 139.05-138.86 (m), 137.50–137.15, 133.62, 127.48, 125.74, 125.14, 121.93, 107.37–107.09 (m), 62.51, 43.58, 37.29, 9.69.
^{19}F NMR (565 MHz, Chloroform-*d*) δ -141.44 (dt, *J* = 22.7, 6.8 Hz), -142.42 (dt, *J* = 22.5, 5.9 Hz), -150.79 (t, *J* = 21.4 Hz), -160.46 (td, *J* = 22.0, 6.7 Hz), -160.77 (td, *J* = 22.0, 6.8 Hz).
LC-HRMS: refer to **Figure S114** and **Table S22**
FTIR (neat) cm^{-1}: 2966 (w), 2937 (w), 2880 (w), 1728 (w), 1518 (w), 1474 (m), 1406 (w), 1387 (w), 1356 (w), 1292 (w), 1242 (w), 1111 (w), 1036 (w), 991 (w), 930 (w), 879 (m), 827 (s), 739 (m), 642 (w).
Melting Point: Decomposition above 180 °C.

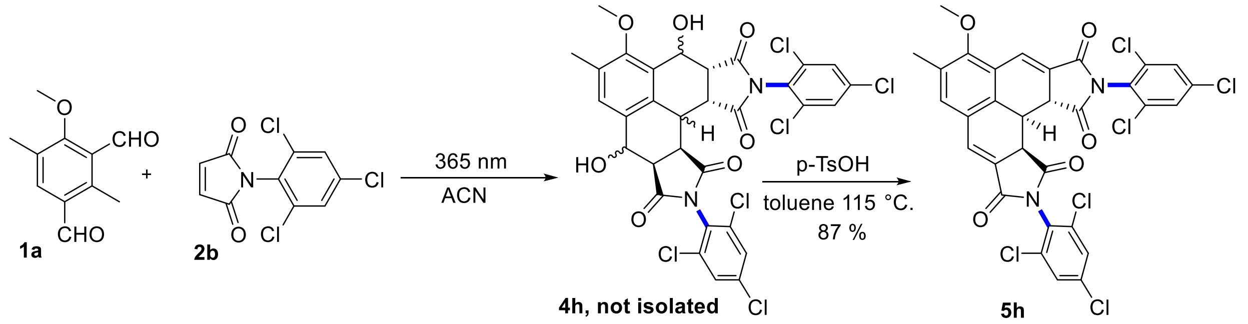

(11a*R*,11b*R*,11c*R*)-5-methoxy-6-methyl-2,10-bis(2,4,6-trichlorophenyl)-11b,11c-dihydro-1*H*-pyrrolo[3',4':2,3]naphtho[1,8*ef*]-isoindole-1,3,9,11(2*H*,10*H*,11a*H*)-tetraone (5h): 4-Methoxy-2,5-dimethylisophthalaldehyde (**1a**, 19.2 mg, 0.10 mmol, 1.00 eq) and 1-(2,4,6-trichlorophenyl)-1*H*-pyrrole-2,5-dione (**2b**, 59.2 mg, 0.205 mmol 2.25 eq) were dissolved in 10 mL dry acetonitrile (20 mL crimp vial, 10 mmol L^{-1}). The solution was degassed by passing through N_2 for 15 min and irradiated for 70 min (385 nm, 0.5 A, 2 cm distance) Afterwards, the solvent was removed under reduced pressure. The crude product mixture 4h contained all possible endo/exo combinations. The crude product and *p*-TsOH monohydrate (1.90 mg, 10 µmol, 10 mol %) were dissolved in 12 mL toluene and the solution was degassed by passing through N_2 for 20 min in a crimp vial. Then the vial was immersed into an oil bath and heated at 115 °C for 24 h. Afterwards the reaction solution was cooled to ambient temperature, the toluene was removed under reduced pressure and the residual crude product was isolated *via* flash chromatography (isocratic DCM:MeOH 98:2 *v/v*). **5h** was obtained as colorless waxy solid (62 mg, 92 % yield).
* Four additional peaks caused by the rotational barriers of the two C_{Ar}-N bonds, caused by the *ortho*-Cl atoms (highlighted in blue).

(4h)
LC-HRMS: refer to **Figure S108**, **Figure S109** and **Table S17**

(5h)
^{1}H NMR (600 MHz, Chloroform-*d*) δ: 7.81 (d, *J* = 2.8 Hz, 1H), 7.45 (d, *J* = 5.1 Hz, 4H), 7.44 (d, *J* = 2.8 Hz, 1H), 7.26 (s, 1H), 4.14 (dd, *J* = 12.9, 2.8 Hz, 1H), 4.09 (dd, *J* = 13.2, 2.8 Hz, 1H), 3.98 (td, *J* = 13.0, 1.1 Hz, 1H), 3.85 (s, 3H), 2.35 (s, 3H).
^{13}C NMR (151 MHz, $CDCl_3$) δ 170.96, 170.87, 164.85, 164.82, 159.29, 136.69, 136.65*, 135.79, 135.37, 135.33*, 134.88, 132.83, 132.04*, 130.49, 128.94, 128.90*, 128.74, 127.41, 127.33, 127.27, 126.41, 126.09, 125.94, 62.22, 43.68, 43.54, 36.36, 15.88.
LC-HRMS: refer to **Figure S115**, **Figure S116** and **Table S23**
FTIR (neat) cm^{-1}: 3067 (w), 3020 (w), 2922 (s), 2853 (m), 1794 (w), 1724 (s), 1666 (w), 1603 (w), 1580 (w), 1556 (m), 1512 (w), 1460 (s), 1375 (m), 1356 (m), 1323 (w), 1261 (m), 1238 (m), 1204 (m), 1173 (m), 1153 (m), 1109 (m), 1076 (m), 1028 (w), 1013 (m), 972 (m), 935 (w), 912 (w), 897 (w), 876 (w), 856 (w), 843 (w), 818 (m), 804 (m), 746 (s), 700 (s), 660 (w), 631 (w), 608 (w).

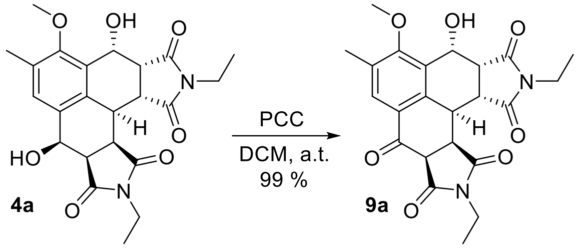

(3a*R*,8*R*,8a*R*,11a*R*,11b*S*,11c*R*)-2,10-diethyl-8-hydroxy-7-methoxy-6-methyl-3a,8,8a,11a, 11b,11c-hexahydro-1*H*-pyrrolo[3',4':2,3]naphtho[1,8-*ef*]isoindole-1,3,4,9,11(2*H*,10*H*)-pentaone (9a): (3a*R*,4*R*,8*R*,8a*R*,11a*R*,11b*R*,11c*R*)-2,10-Diethyl-4,8-dihydroxy-5-methoxy-6-methyl-3a,8,8a,11a, 11b,11c-hexahydro-1*H*-pyrrolo[3',4':2,3]naphtho[1,8-*ef*]iso-indole-1,3,9,11-(2*H*,4*H*,10*H*)-tetraone (**4a,** 30 mg, 0.068 mmol, 1.00 eq) was dissolved in 7 mL dry DCM and pyridinium chlorochromate (PCC, 22 mg, 0.102 mmol, 1.50 eq) was added. The reaction mixture was stirred at ambient temperature for 6 h. Afterwards, the 1 mL of isopropyl alcohol was added, the mixture stirred for 30 min and the solution filtered through a short plug of silica gel (washing with DCM/MeOH 99:1 *v/v*). The product **9a** was obtained as colorless solid without further purification (29.5 mg, 99% yield).

^{1}H NMR (600 MHz, DMSO-d_6) δ: 7.63 (d, J = 0.9 Hz, 1H), 5.63 (dd, J = 3.9, 1.2 Hz, 1H), 5.37 (t, J = 3.6 Hz, 1H), 4.57 (dd, J = 9.3, 6.5 Hz, 1H), 4.20 (d, J = 7.4 Hz, 1H), 4.11 (dd, J = 7.4, 5.8 Hz, 1H), 3.89 (td, J = 6.2, 1.2 Hz, 1H), 3.80 (s, 3H), 3.53 – 3.41 (m, J = 7.2 Hz, 2H), 3.33 – 3.26 (m, 2H), 3.15 (ddd, J = 9.3, 3.4, 1.1 Hz, 1H), 2.26 (s, 3H), 1.09 (t, J = 7.2 Hz, 3H), 0.92 (t, J = 7.2 Hz, 3H).
^{13}C NMR (151 MHz, DMSO-d_6) δ: 186.59, 179.89, 177.64, 176.77, 169.68, 160.12, 139.02, 131.35, 130.65, 130.25, 126.82, 61.77, 59.78, 54.02, 45.47, 43.09, 37.10, 33.38, 32.85, 27.89, 15.63, 12.73, 12.55.
LC-HRMS: refer to **Figure S119**, **Table S26** and **Scheme S16**
FTIR (neat) cm^{-1}: 3096 (w, br), 2986 (w), 2941 (w), 2850 (w, br), 1782 (w), 1767 (w), 1707 (s), 1684 (s), 1668 (s), 1595 (m), 1570 (m), 1441 (m), 1398 (m), 1373 (w), 1346 (s), 1310 (m), 1294 (m), 1254 (m), 1225 (s), 1163 (m), 1119 (m), 1076 (m), 1047 (m), 1024 (m), 1014 (m), 1003 (s), 964 (m), 937 (m), 922 (m), 899 (w), 889 (w), 847 (m), 824 (m), 810 (w), 787 (m), 764 (m), 739 (m), 723 (m), 696 (w), 685 (m), 667 (w), 648 (m), 622 (w), 613 (w).
Melting Point: Decomposition above 220 °C.
Elemental Analysis: calculated for $C_{23}H_{24}N_2O_7$: C: 62.72, H: 5.49, N: 6.36 − found: C: 61.91, H: 5.33, N: 5.74.

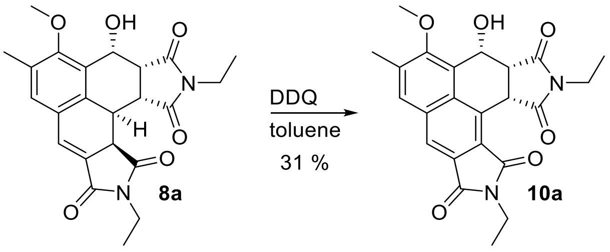

(3a*R*,4*R*,11c*S*)-2,10-diethyl-4-hydroxy-5-methoxy-6-methyl-3a,11c-dihydro-1*H*-pyrrolo-[3',4':2,3]naphtho[1,8-*ef*]isoindole-1,3,9,11(2*H*,4*H*,10*H*)-tetraone (10a): A solution of (3a*R*, 4*R*,11a*R*,11b*R*,11c*R*)-2,10-diethyl-4-hydroxy-7-methoxy-6-methyl-3a,11a,11b,11c-tetra-hydro-1*H*-pyrrolo[3',4':2,3]naphtho[1,8-*ef*]isoindole-1,3,9,11(2*H*,4*H*,10*H*)-tetraone (**8a,** 15 mg, 0.035 mmol, 1.00 eq) and 2,3-dichlor-5,6-dicyano-1,4-benzochinon (DDQ, 24.1 mg, 0.106 mmol, 3.00 eq) in 3.5 ml toluene was degassed by passing through a stream of nitrogen and heated to 95 °C for 16 hours. The resulting solution was cooled to room temperature and washed with 5 % aq. NaOH solution, followed by brine. The organic layer was dried over Na_2SO_4 and evaporated to dryness in vacuo. The residual crude product was isolated *via* flash chromatography (gradient *n*-hexane: ethyl acetate 80:20-0:100 *v/v*). **10a** was obtained as yellowish solid (4.3 mg, 31 % yield).

^{1}H NMR (600 MHz, Chloroform-*d*) δ: 7.90 (d, *J* = 1.1 Hz, 1H), 7.66 (s, 1H), 5.88 (d, *J* = 4.1 Hz, 1H), 5.05 (d, *J* = 7.7 Hz, 1H), 3.93 (s, 3H), 3.75 (dddd, *J* = 28.1, 20.7, 12.5, 6.8 Hz, 4H), 3.41 (dd, *J* = 7.7, 4.0 Hz, 1H), 3.07 (s, 1H), 2.50 (d, *J* = 1.0 Hz, 3H), 1.31 (t, *J* = 7.2 Hz, 3H), 1.23 (t, *J* = 7.2 Hz, 3H).
^{13}C NMR (151 MHz, Chloroform-*d*) δ: 176.42, 173.96, 168.28, 167.72, 157.17, 135.24, 133.50, 132.09, 129.98, 126.77, 126.54, 125.83, 125.43, 123.60, 62.70, 61.63, 45.11, 39.57, 34.59, 33.34, 17.03, 13.96, 12.85.
LC-HRMS: refer to **Figure S120 Table S27** and **Scheme S17**

FTIR (neat) cm^{-1}: 3441 (w, br), 2989 (w), 2924 (w), 2876 (w), 2849 (w), 2735 (w), 1757 (m), 1691 (s), 1668 (s), 1597 (w), 1555 (m), 1472 (m), 1456 (m), 1441 (m), 1393 (s), 1373 (m), 1344 (m), 1325 (m), 1302 (m), 1277 (m), 1244 (m), 1227 (m), 1211 (m), 1184 (m), 1165 (m), 1086 (m), 1067 (s), 1051 (s), 1018 (m), 982 (m), 959 (w), 895 (m), 878 (m), 800 (w), 788 (w), 762 (m), 754 (m), 737 (s), 687 (w), 665 (w).
Melting Point: Decomposition above 220 °C.
Elemental Analysis: calculated for $C_{23}H_{22}N_2O_6$: C: 65.40, H: 5.25, N: 6.63 − found: C: 65.31, H: 5.22, N: 6.26.

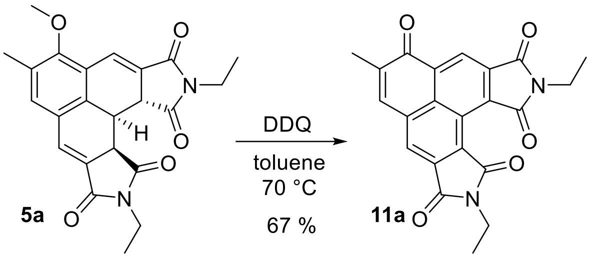

2,10-diethyl-6-methyl-1H-pyrrolo[3',4':2,3]naphtho[1,8-*ef*]isoindole-1,3,5,9,11(2*H*,10*H*)-pentaone (11a): A solution of (11a*R*,11b*R*,11c*R*)-2,10-diethyl-5-methoxy-6-methyl-11b,11c-dihydro-1*H*-pyrrolo[3',4':2,3]-naphtho[1,8-*ef*]isoindole-1,3,9,11(2*H*,10*H*,11a*H*)-tetraone (**5a,** 15 mg, 0.035 mmol, 1.00 eq) and 2,3-dichloro-5,6-dicyano-1,4-benzochinon (DDQ, 17.61 mg, 0.078 mmol, 2.11 eq) in 4 ml toluene was degassed by passing through a stream of nitrogen and heated to 70 °C for 30 hours. The resulting solution was cooled to room temperature and washed with 5 % aq. NaOH solution, followed by brine. The organic layer was dried over Na_2SO_4 and evaporated to dryness in vacuo. The residual crude product was isolated *via* flash chromatography (gradient *n*-hexane: ethyl acetate 80:20-40:60 *v/v*). **11a** was obtained as yellow crystalline solid (9.6 mg, 67 % yield).

^{1}H NMR (600 MHz, Chloroform-*d*) δ: 9.05 (s, 1H), 8.10 (s, 1H), 7.74 – 7.63 (m, 1H), 3.87 (dq, *J* = 14.4, 7.2 Hz, 4H), 2.29 (d, *J* = 1.4 Hz, 3H), 1.36 (td, *J* = 7.2, 4.2 Hz, 6H).
^{13}C NMR (151 MHz, Chloroform-*d*) δ 184.02, 166.86, 166.51, 165.90, 165.66, 139.76, 138.19, 136.42, 135.44, 135.27, 134.14, 133.58, 133.55, 129.94, 125.12, 123.71, 120.16, 33.99, 33.87, 16.48, 14.04, 13.99.
LC-HRMS: refer to **Figure S121**, **Table S28**
FTIR (neat) cm^{-1}: 3049 (w), 2953 (w), 2929 (w), 2851 (w), 1780 (w), 1765 (m), 1703 (s), 1641 (s), 1568 (w), 1529 (w), 1464 (w), 1445 (m), 1404 (m), 1393 (s), 1352 (m), 1327 (m), 1302 (m), 1204 (m), 1180 (w), 1163 (w), 1140 (w), 1088 (m), 1068 (m), 1030 (m), 991 (w), 959 (w), 928 (m), 914 (m), 885 (w), 876 (w), 843 (w), 829 (w), 808 (m), 777 (s), 748 (m), 717 (m). 662 (m).
Melting Point: Decomposition above 200 °C.
Elemental Analysis: calculated for $C_{22}H_{16}N_2O_5$: C: 68.04, H: 4.15, N: 7.21 − found: C: 67.82, H: 4.07, N: 7.14.

VI. Procedures for the Generation of PLYD Neutral Radicals

In the following section, the procedures for generation of PLYDs **6a**, **6d**, **6g** and **6h** are described. Importantly, the temperature for the DDQ-oxidation of the substrates **5a**, **5d**, **5g** and **5h** need to be judiciously elected to match the redox-potential of the substrate and concomitantly avoid overoxidation and subsequent side-reactions. For instance, the first oxidation attempt of **5a** led to the formation of **11a** instead of the desired neutral radical **6a**. For **6d**, the assumed intermediates involved in the oxidation and subsequent hydrolysis could be directly observed in the LC-HRMS (refer to **Figure S112** and **Scheme S15**). Substrate **5f** did not afford the corresponding neutral radical, possibly due to the high oxidation potential of the substrate and the required high temperatures for the DDQ oxidation that led to competing side-reactions.

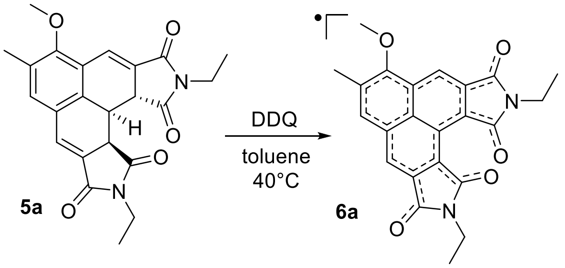

Neutral radical **6a**: A solution of (11a*R*,11b*R*,11c*R*)-2,10-diethyl-5-methoxy-6-methyl-11b,11c-dihydro-1*H*-pyrrolo[3',4':2,3]-naphtho[1,8-*ef*]isoindole-1,3,9,11(2*H*,10*H*,11a*H*)-tetraone (**5a**, 12.2 mg, 0.03 mmol, 1.00 eq) and 2,3-dichlor-5,6-dicyano-1,4-benzochinon (DDQ, 13.6 mg, 0.06 mmol, 2.00 eq) in 2 mL toluene was degassed by passing through a stream of nitrogen and heated to 40 °C for 24 hours. The resulting solution was cooled on an ice-bath and washed with 5% aq. NaOH solution, followed by brine. The organic layer was dried over Na_2SO_4 and the volatiles removed at ambient temperature under high vacuum to provide **6a**.

EPR: refer to **Figure S6** and **Table S2**.

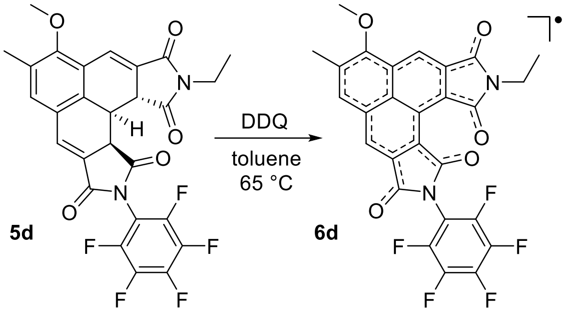

Neutral radical **6d**: A solution of (11a*R*,11b*S*,11c*R*)-2-ethyl-5-methoxy-6-methyl-10-(perfluorophenyl)-11b,11c-dihydro-1*H*-pyrrolo[3',4':2,3]naphtho[1,8-*ef*]isoindole-1,3,9,11(2*H*, 10*H*,11a*H*)-tetraone (**5d**, 15 mg, 0.022 mmol, 1.00 eq) and 2,3-dichlor-5,6-dicyano-1,4-benzochinon (DDQ, 10.0 mg, 0.044 mmol, 2.00 eq) in 2 mL toluene was degassed by passing through a stream of nitrogen and heated to 65 °C for 48 hours. The resulting solution was cooled on an ice-bath and washed with 5% aq. NaOH solution, followed by brine. The organic layer was dried over Na_2SO_4 and the volatiles removed at room temperature under high vacuum to provide **6d**.

EPR: refer to **Figure S7** and **Table S2**.

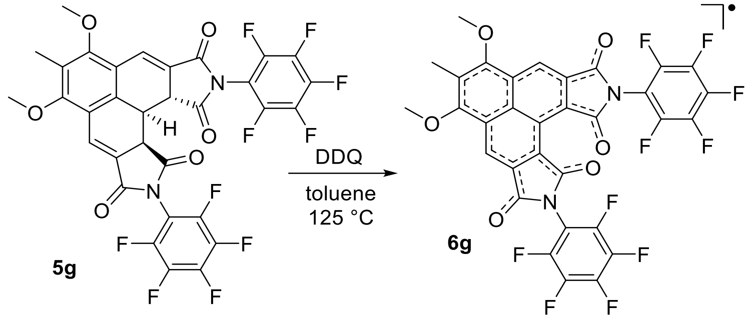

Neutral radical **6g**: A solution of (11a*R*,11c*R*)-5,7-dimethoxy-6-methyl-2,10-bis(perfluorophenyl)-11b,11c-dihydro-1*H*-pyrrolo[3',4':2,3]naphtho[1,8-*ef*]isoindole-1,3,9,11-(2*H*,10*H*,11a*H*)-tetraone (**5g,** 30 mg, 0.042 mmol, 1.00 eq) and 2,3-dichlor-5,6-dicyano-1,4-benzochinon (DDQ, 19.2 mg, 0.084 mmol, 2.00 eq) in 4 mL toluene was degassed by passing through a stream of nitrogen and heated to 125 °C for 15 hours. The resulting solution was cooled on an ice-bath and washed with 5% aq. NaOH solution, followed by brine. The organic layer was dried over Na_2SO_4 and the volatiles removed at room temperature under high vacuum to provide **6g**.

EPR: refer to **Figure S8** and **Table S2**.
LC-HRMS: refer to **Figure S122** and **Table S29**

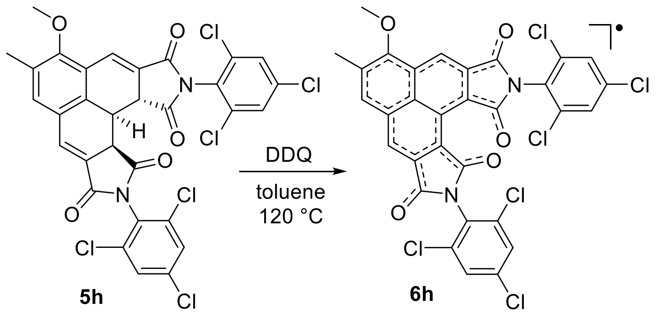

Neutral radical **6h**: A solution of (11a*R*,11b*R*,11c*R*)-5-methoxy-6-methyl-2,10-bis(2,4,6-trichlorophenyl)-11b,11c-dihydro-1*H*-pyrrolo[3',4':2,3]naphtho[1,8-*ef*]isoindole-1,3,9,11-(2*H*,10*H*,11a*H*)-tetraone (**5h,** 28.50 mg, 0.04 mmol, 1.00 eq) and 2,3-dichlor-5,6-dicyano-1,4-benzochinon (DDQ, 18.3 mg, 0.081 mmol, 2.00 eq) in 4 mL toluene was degassed by passing through a stream of nitrogen and heated to 40 °C for 24 hours. The resulting solution was cooled on an ice-bath and washed with 5% aq. NaOH solution, followed by brine. The organic layer was dried over Na_2SO_4 and the volatiles removed at room temperature under high vacuum to provide **6h**.

EPR: refer to **Figure S9** and **Table S2**.

VII. Results and Discussion

Stereo- and regiochemistry of the consecutive Diels-Alder reactions: The stereochemistry of the two consecutive Diels-Alder reactions of the *o*-QDMs formed from MIAs and FBIs with non-cyclic dienophiles can potentially lead to $2^7 = 128$ stereoisomers (or 64 diastereomers). Furthermore another 2^7 regioisomeric stereoisomers can be formed if the regioselectivity is reduced or inverted, since the two non-identical dienophiles are exchanged as a consequence. The key considerations explaining the finding that only two (a pair of enantiomers) of 128 stereoisomers are formed as major products are summarized in

Scheme S1. The first reason for the reduction of the number of possible stereoisomers is that maleimides e.g. achiral, cyclic dienophiles are used. Therefore, the two maleimide bridgehead stereocenters (β = γ; ε = ζ) are dependent of one another and the number of stereoisomers is reduced to $2^5 = 32$.

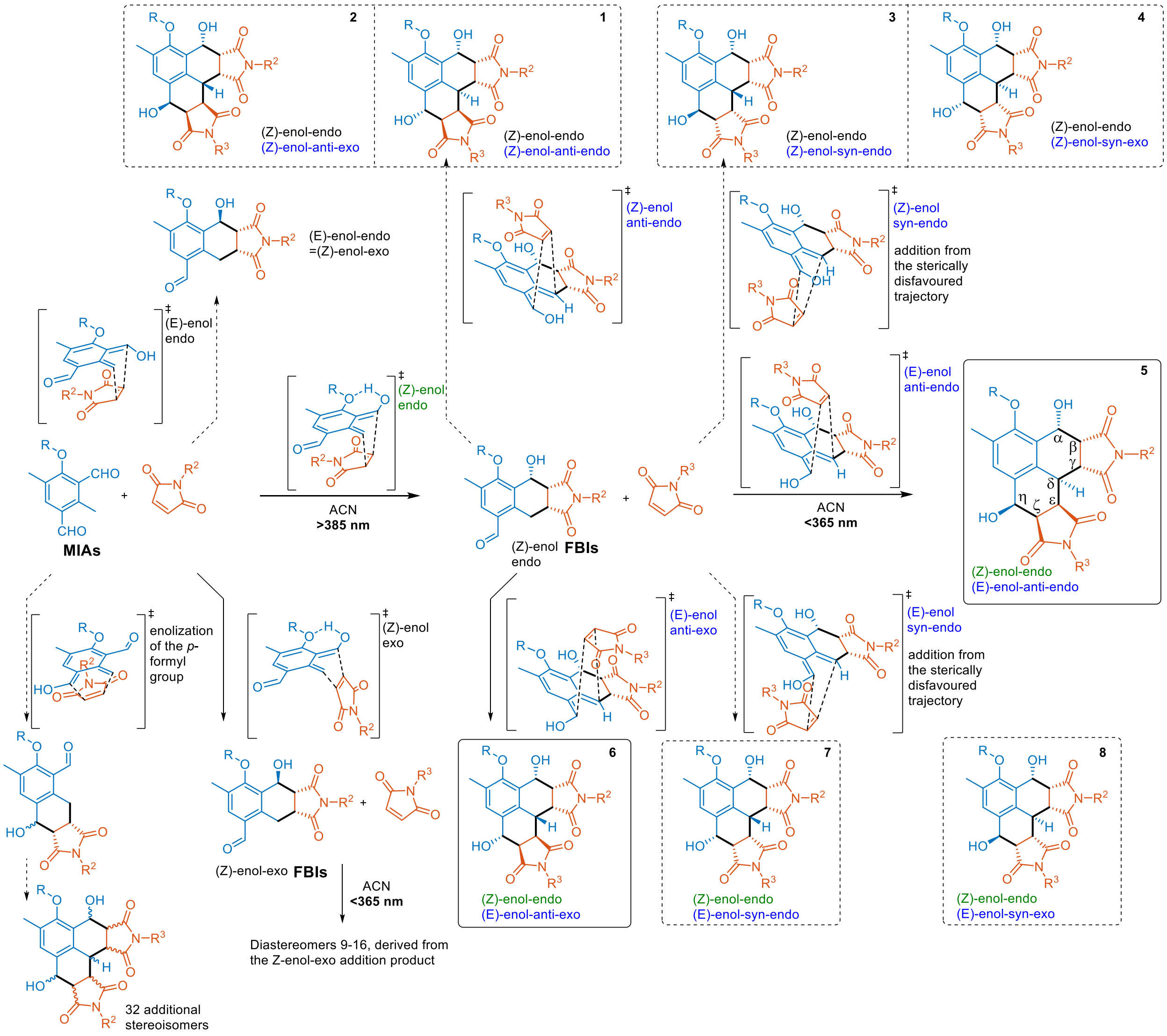

Scheme S1 Illustration of the key steps that determine the stereochemistry of the HPDDs obtained in this work, including the relevant transition state geometries. The dotted boxes and arrows indicate reaction pathways that can be ruled out by the observed stereochemistry of the isolated products. The sold lines represent products that are formed. The transition states and considerations towards diastereomers **9-16** are similar to diastereomers **1-8**, with the difference of the α-stereocenter being inverted. Therefore, these transition states are not being displayed again.

The assumed formation of the (Z)-enol as reactive intermediate in the first addition does not reduce the number of stereoisomers, since the product of the *endo*-addition to the (E)-enol is identic to the *exo*-addition to the (Z)-enol. In contrast,

for the addition to the enol derived from the FBIs the configuration of the enol-intermediate is important for the stereochemical reaction outcome. If an (E)-enol is formed, the relative positions of the hydrogen (δ) and hydroxy-moieties (η) must be anti-periplanar for both addition trajectories (syn and anti) and for either *endo* or *exo*-addition. In this reaction, we did not observe any product with syn-periplanar hydrogen (δ) and hydroxy-moieties (η), therefore the formation of the (Z)-enol can be ruled out (diastereomers **1-4** are not formed, as well as their analogues **13-16** derived from the exo-FBI), reducing the number of possible stereoisomers to 2^4 = 16. Finally, the addition from the sterically disfavoured *syn*-trajectory was not observed, reducing the number of stereoisomers to 8 e.g. 4 diastereomers (marked with the solid line box in **Scheme S2**. The major stereoisomer **5** is formed by two consecutive *endo*-additions to first (Z)-enol and second (E)-enol. *Exo*-addition to the (Z)-enol can be enforced by the use of a non-planar maleimide substrate.

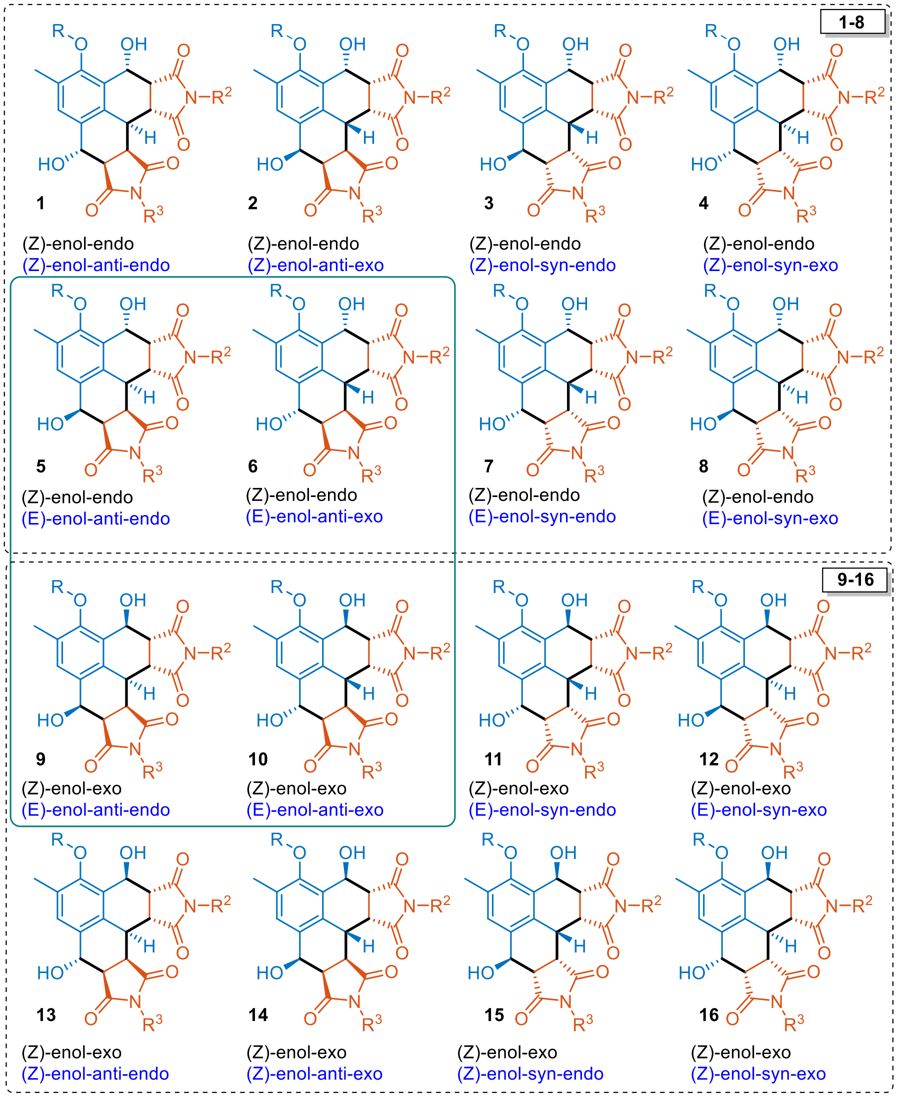

Scheme S2 Collation of the 16 diasteromers (32 stereoisomers, only one enantiomer is depicted) that could potentially be formed if no stereoselectivity was observed. The dotted lines group the products derived from endo-FBIs and exo-FBIs, the solid line indicates the 4 diastereomers that were experimentally observed in this work.

Transformations of 4a: As described in the main text, a feasible route from HDDP **4a** towards PLYD **6a** was explored. Along with finding the suitable reaction conditions for the desired transformation, several additional useful reactions were found (refer Scheme S3 for a summary and to section V for experimental details). Initially, the acid catalyzed E1-elimination of **4a** at 105 °C afforded a regioisomeric mixture of hydroxy-tetrahydro-phenalene diimides **7a** and **8a**. Notably, the OH-group in *peri*-position to the OMe group is removed at a lower proportion. The removal of two equivalents of water from **4a** at increased temperature

(115 °C) afforded dihydro-phenalene diimide **5a** in excellent yield. Compared to **4a** (Φ_F = 15 ± 2 %, $\lambda_{F,max}$ = 450 nm), **5a** exhibits a bathochromic shift of both absorption and fluorescence wavelength and a minor increase in fluorescence quantum yield (Φ_F = 18 ± 1 %, $\lambda_{F,max}$ = 470 nm). First attempts of the DDQ oxidation of **5a** in toluene at 70 °C and higher temperatures provided primarily phenalene-1-on diimide **11a**, instead of the desired neutral radical. Importantly, phenalenones are found in plants and microbes and possess significant biological activity,[10] but phenalenon diimides have not yet been described to the best of our knowledge. DDQ oxidation in toluene at 35 °C, however, led to the phenalenyl neutral radical **6a**. As described in the main text, the decomposition of **6a** under ambient conditions in solution leads to the formation of **11a**. We assume, that the radical cation of **6a** is instable and rearranges to **11a**. Furthermore, attempts to oxidize **7a** and **4a** to the respective oxophenalenyl diimide or oxophenalenyloxy diimide neutral radicals were unsuccessful. Selective oxidation of a single hydroxy-group (non-*peri* to the OMe group) with a pyridinium chlorochromate led to hydroxy-hexahydro phenalenon diimide **9a** in nearly quantitative yield. Lastly, oxidation of **7a** with DDQ afforded tetrahydrophenalenol diimide **10a**, however in low yield.

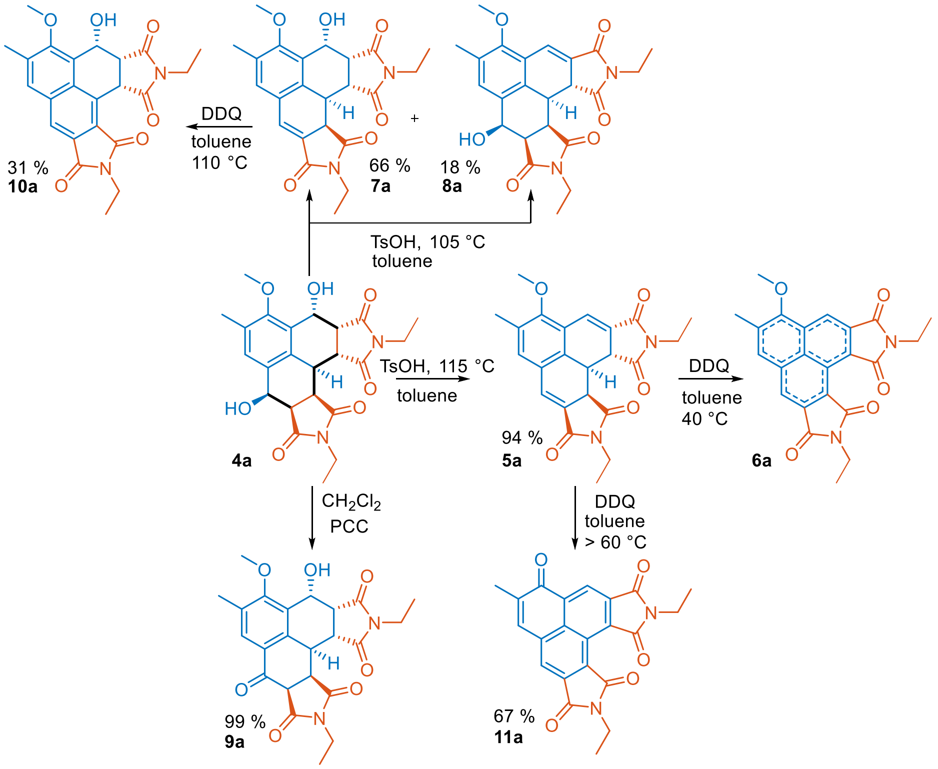

Scheme S3 Transformations of hexahydro-phenalene-1,6-diol diimide (HPDD) **4a** to various products **5a-11a** *via* acid catalyzed elimination and/or oxidation reactions.

Synthesis of additional neutral radicals 6d, 6g and 6h: To increase the stability of radical **6a** and study the electronic influence of the substituents, dihydrophenalenes **5d**, **5f**, **5g** and **5h** neutral radicals **6d, 6g** and **6h** were synthesized. They feature electron withdrawing substituents on the maleimide group (refer to Scheme S4). Increasing temperatures of 65 °C for **5d** to 125 °C for **5g** were required for the DDQ oxidation, indicating both an increasing oxidation potential and a better radical stability compared to PLYD **6a**. Attempts to convert **5f** to a neutral radical failed surprisingly. **6g**, featuring not only two electron withdrawing pentafluorophenyl maleimide substituents but also an additional OMe-group on the α_2-position was observed to be stable for several weeks under ambient conditions. We hypothesize that the additional push-pull effect may play a role for the stabilization of this radical. LC-HRMS of **6g** (refer to Figure S122) was performed without apparent decomposition, showing a single peak in the UV-channel and a single product signal in the negative ionization mode and confirming the purity of the obtained molecule.

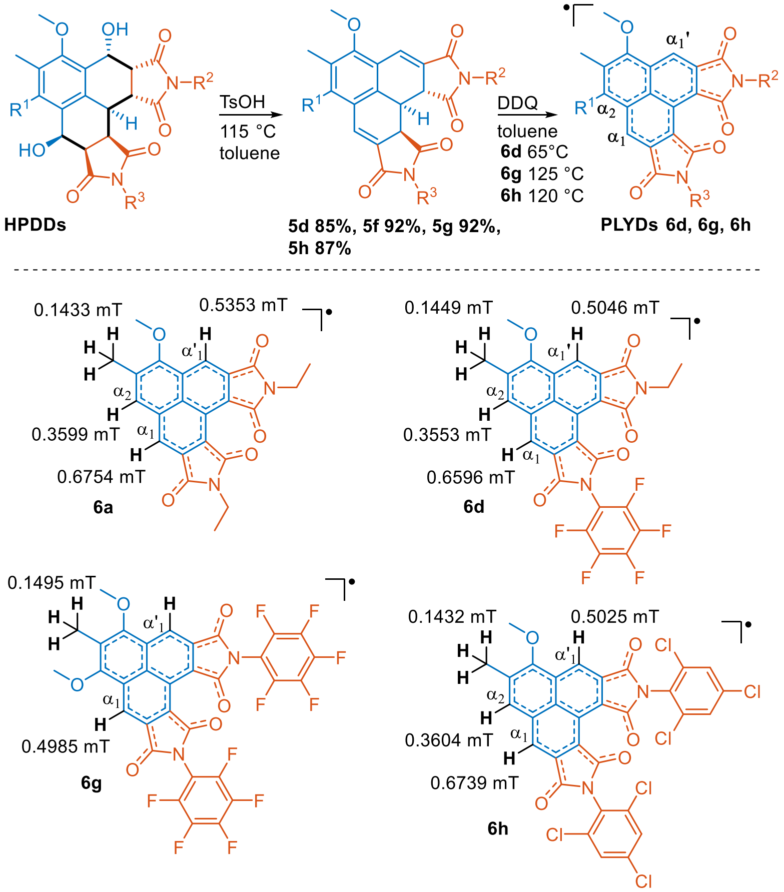

Scheme S4 (Top) Synthesis of dihydro-phenalenes **5d**, **5g** and **5h** *via* elimination and subsequent oxidation to PLYD radicals **6d**, **6g** and **6h**. (Bottom) PLYD Neutral radicals presented in this work including hyperfine coupling constants.

Determination of the hyperfine coupling constants (hfccs): The simulations/fitting of the EPR spectra was performed using the EASYSPIN toolbox for MATLAB®[6] using the fast movement isotropic model 'garlic'. The hyperfine coupling of the OMe-group(s), the substituents of the maleimide groups, for instance ethyl or trichlorophenyl (^{37}Cl and ^{39}Cl) were experimentally not observed and therefore not taken into account for the optimization. Furthermore, the coupling of the ^{13}C nuclei was not taken into account, due to the number of non-equivalent carbon atoms in the PLYD radicals presented herein. Initially, the C_{2v}-symmetry of **6g,** allowed for a reduction of spin system to the 3 methyl protons and two equivalent α-protons. A least square fit using the Nelder/Mead downhill simplex method was used. The optimization parameters were the two hfccs and the linewidth resulting in a fitting with a small root mean square deviation (RMSD). The obtained hfccs and line width were subsequently used as starting points for the optimization of the more complex (more independent optimization parameters) PLYD radicals **6a**, **6d** and **6h**, having two additional coupling protons. The results are summarized in **Table S2**. The corresponding EPR spectra are depicted in **Figure S7**, **Figure S8** and **Figure S9**. Apparently, the exchange of the maleimide substituents influence the hfccs, for instance the $α_1$ hfcc of **6a** compared to **6d**. The additional α-OMe group in **6g** reduces this hfcc further, however increases the hfcc of the ß-CH_3 group. With the herein presented synthesis method the fine tuning of the phenalenyl diimide system is easily possible without the necessity to perform a multitude of multi-step syntheses.

Table S2 Collation of the hyperfine coupling constants (hfccs) obtained from the EPR-simulation for free radicals **6a**, **6d**, **6g** and **6h**.

**hfccs**	**6a: MHz (mT)**	**6d: MHz (mT)**	**6g: MHz (mT)**	**6h: MHz (mT)**
$α_1$	18.9299 (0.6754)	18.4899 (0.6596)	13.9748 (0.4985)	18.9034 (0.6739)
'	15.0028 (0.5353)	14.1450 (0.5046)	$α_1$='	14.0938 (0.5025)
	10.0853 (0.3599)	9.9593 (0.3553)	--	10.1102 (0.3604)
CH_3	4.0152 (0.1433)	4.0629 (0.1449)	4.1910 (0.1495)	4.1521 (0.1432)

Cyclic voltammetry: For the most stable radical **6g** and its precursor **5g**, CV measurements were performed, refer to **Figure S10** and **Figure S11**. The cyclic voltammogram of **6a** indicates that the oxidation to the phenalenyl cation at 0.45 V is not reversible, and the reversible reduction to the phenalenyl anion is followed by a two-electron reduction, presumably involving the imide groups. We speculate, that the formation of a phenalenone similar to the observed **11a** occurs after oxidation to the cation by a carbocation rearrangement, resulting in the non-reversibility of the oxidation wave. Two-electron reduction is commonly observed for aromatic diimides,[11] however the low reduction potential of -0.40 V is unexpected. In the precursor **5g,** we found a non-reversible reduction potential of -1.47 V.

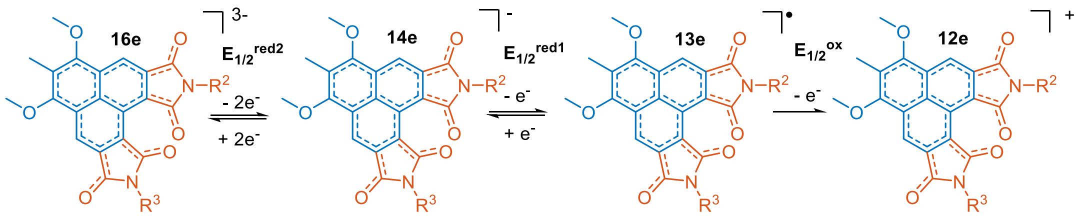

Scheme S5 Oxidation of PLYD **6g** to the respective cation (12e) and reduction to anions (14e and 16e). The cation was observed to be unstable in the CV measurement, probably due to the previously observed decay to the phenalenone.

VIII. EPR spectra and Simulations

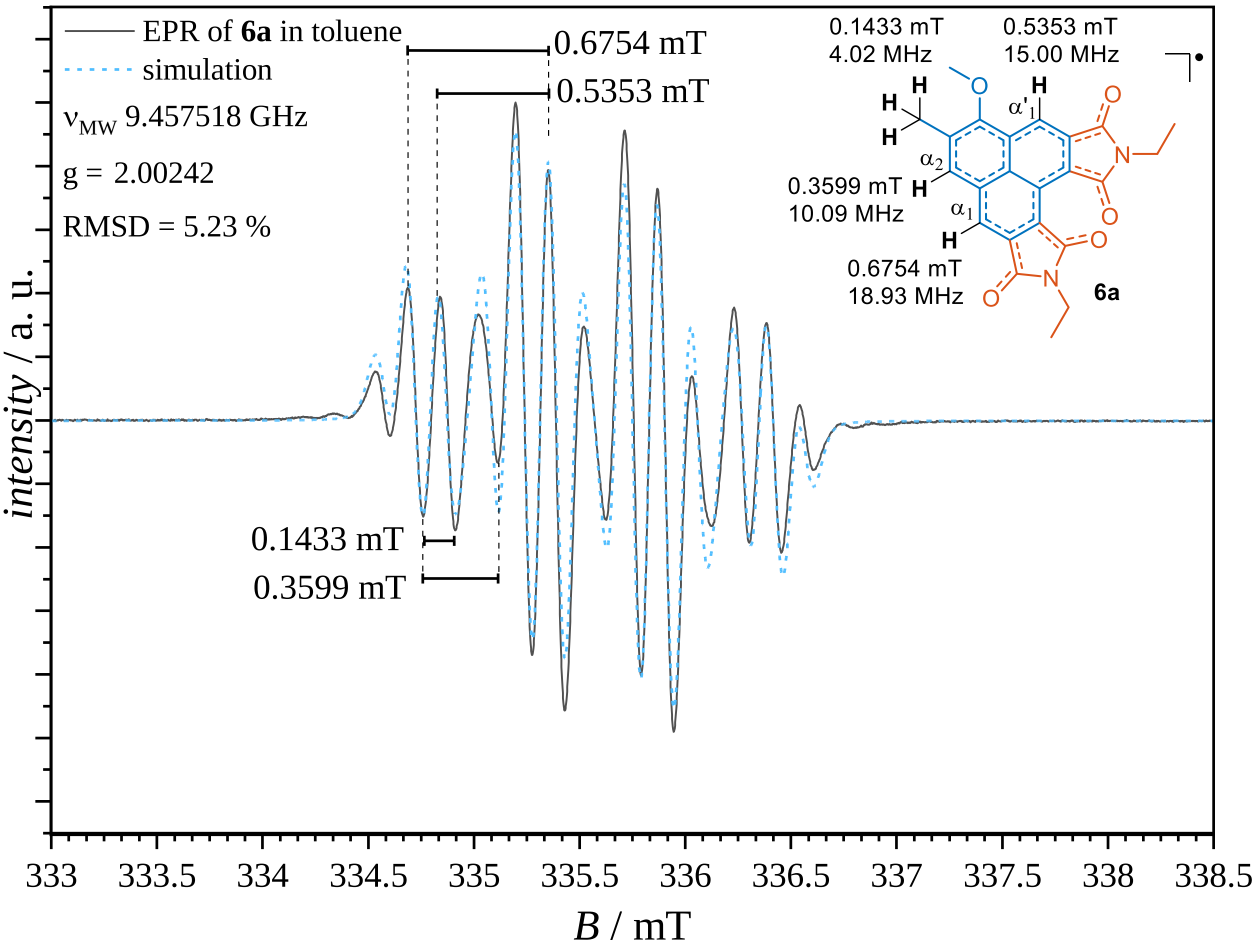

Figure S6 EPR-spectrum of free radical **6a** (black) recorded in toluene, microwave frequency and observed g-factor. Simulation (dotted, blue), resulting hfccs and root mean square deviation (RMSD).

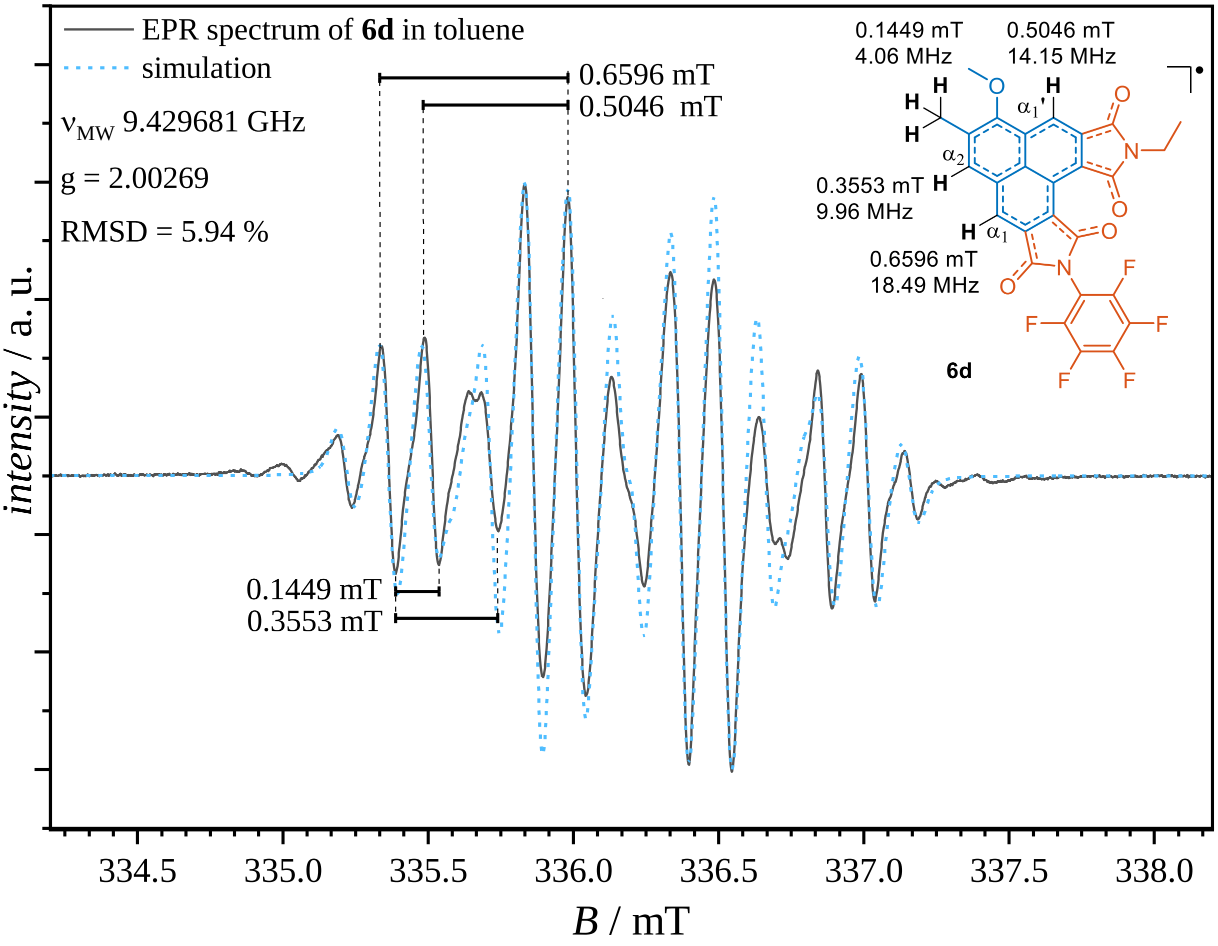

Figure S7 EPR-spectrum of free radical **6d** (black) recorded in toluene, microwave frequency and observed g-factor. Simulation (dotted, blue), resulting hfccs and root mean square deviation (RMSD).

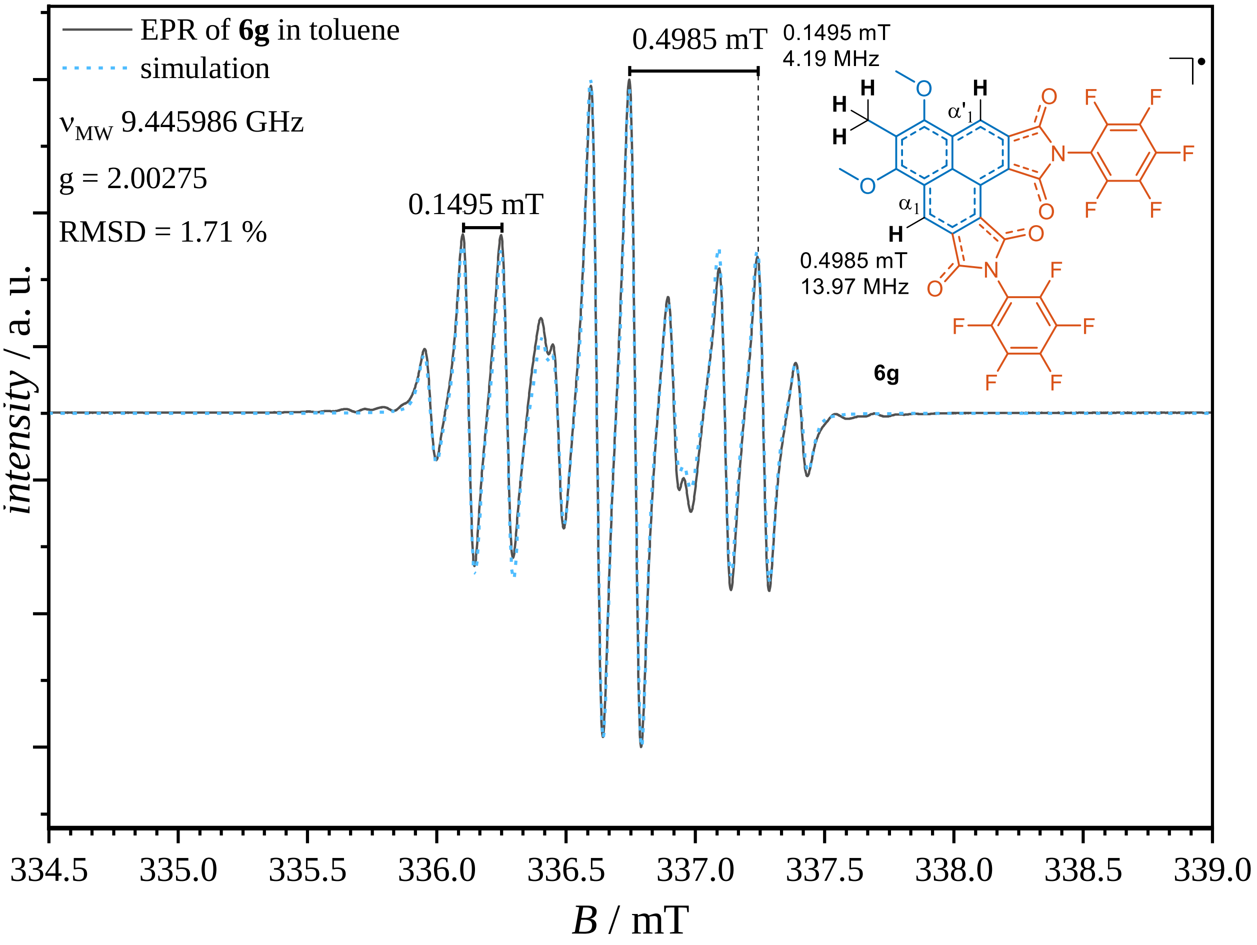

Figure S8 EPR-spectrum of free radical **6g** (black) recorded in toluene, microwave frequency and observed g-factor. Simulation (dotted, blue), resulting hfccs and root mean square deviation (RMSD).

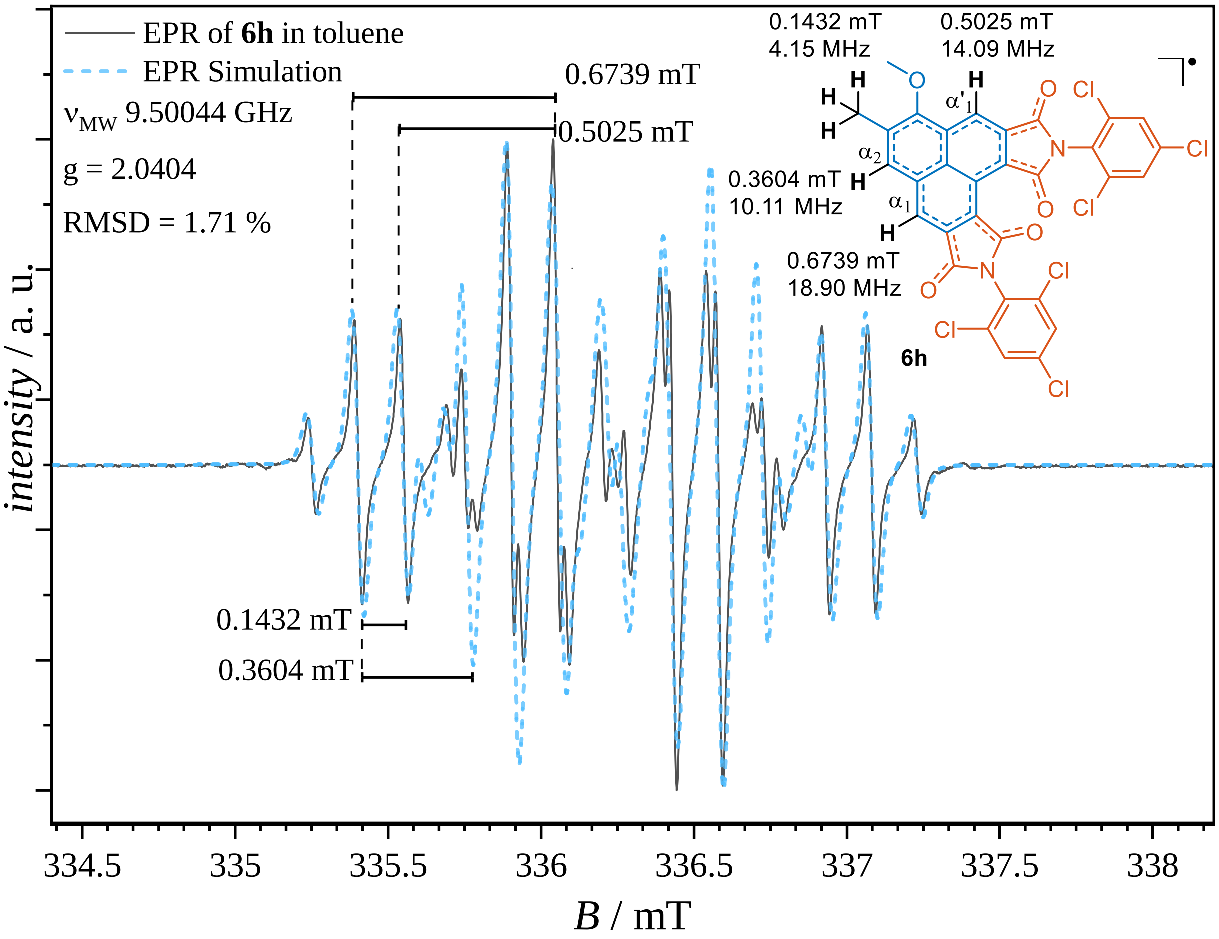

Figure S9 EPR-spectrum of free radical **6h** (black) recorded in toluene, microwave frequency and observed g-factor. Simulation (dotted, blue), resulting hfccs and root mean square deviation (RMSD).

IX. Cyclic Voltammetry

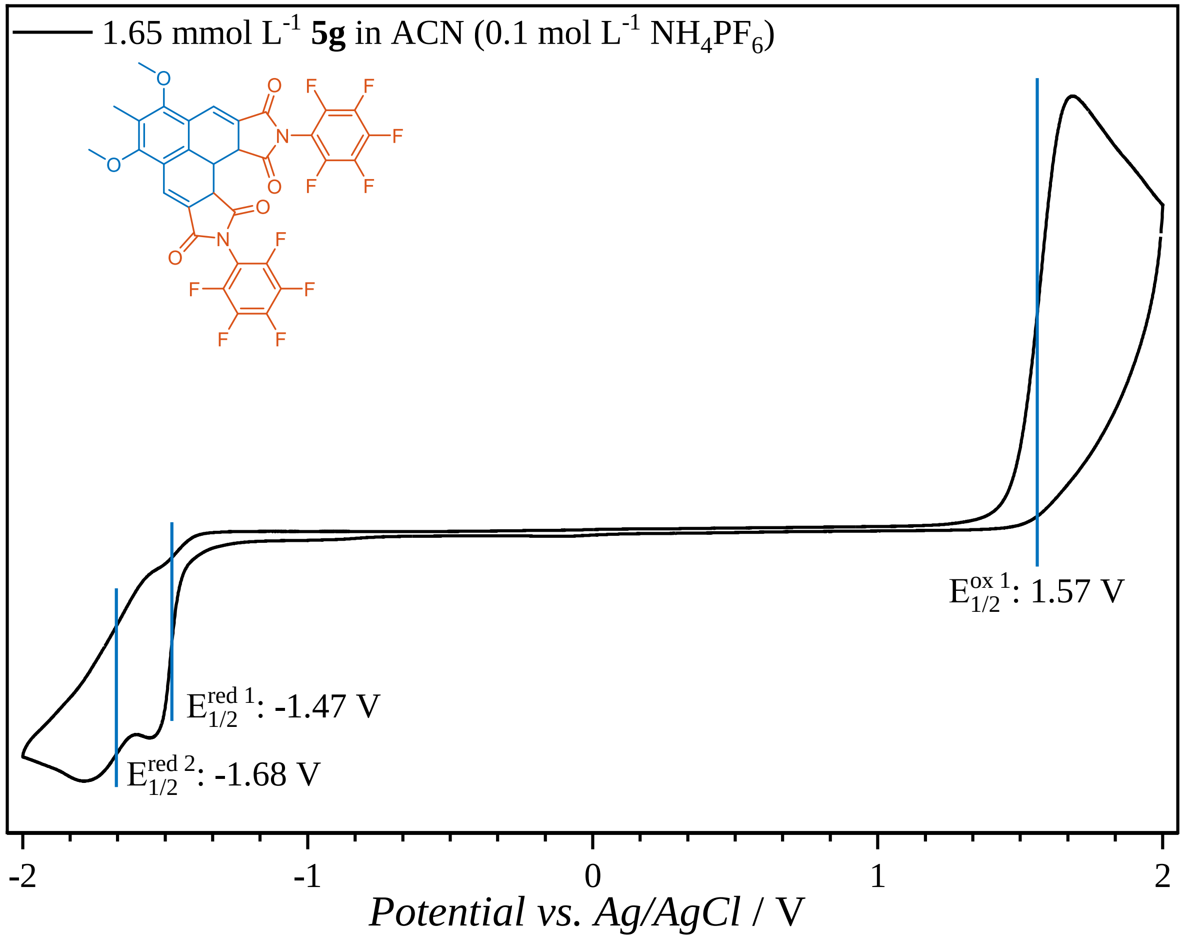

Figure S10 Cyclic voltammogram of **5g** 1.65 mmol L^{-1} in dry acetonitrile (Bu_4NPF_6 0.1 mol L^{-1}), scan rate 75 mVs^{-1}. Non-reversible oxidation wave at 1.57 V and non-reversible reduction waves at -1.47 and -1.68 V were observed.

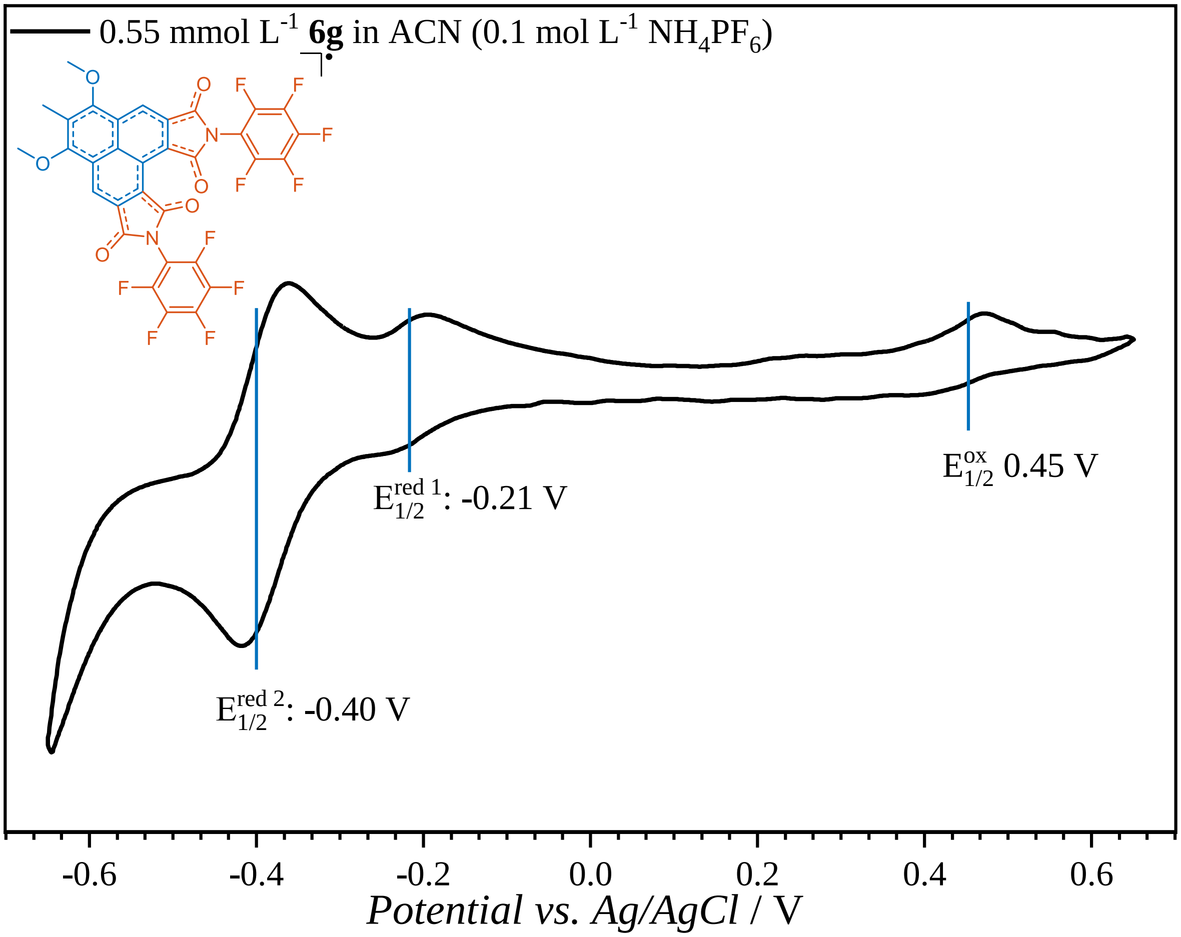

Figure S11 Cyclic voltammogram of **6g** 0.55 mmol L^{-1} in dry acetonitrile (Bu_4NPF_6 0.1 mol L^{-1}), scan rate 150 mVs^{-1}. Non-reversible oxidation wave at 0.45 V and reversible reduction waves at -0.21 and -0.40 V were observed.

X. Single Crystal XRD

Crystal Data for (3a*R*,4*R*,8*R*,8a*R*,11a*R*,11b*R*,11c*R*)-2,10-diethyl-4,8-dihydroxy-5-methoxy-6-methyl-3a,8,8a,11a,11b,11c-hexahydro-1*H*-pyrrolo[3',4':2,3]naphtho[1,8-*ef*]isoindole-1,3,9,11-(2*H*,4*H*,10*H*)-tetraone (**4a**), $C_{23.05}H_{26.075}N_{2.0125}O_7$ (M =443.31 g/mol): triclinic, space group P-1 (no. 2), a = 10.6979(2) Å, b = 12.4717(2) Å, c = 17.4011(2) Å, α = 90.7500(10)°, β = 97.1530(10)°, γ = 110.857(2)°, V = 2148.52(6) Å^3, Z = 4, T = 150.00(10) K, μ(MoK_α) = 0.102 mm^{-1}, D_{calc} = 1.370 g/cm^3, 57576 reflections measured (3.502° ≤ 2Θ ≤ 65.962°), 13836 unique (R_{int} = 0.0457, R_{sigma} = 0.0405) which were used in all calculations. The final R_1 was 0.0492 (I > 2σ(I)) and wR_2 was 0.1414 (all data). CCDC deposition number: CCDC 1988977.

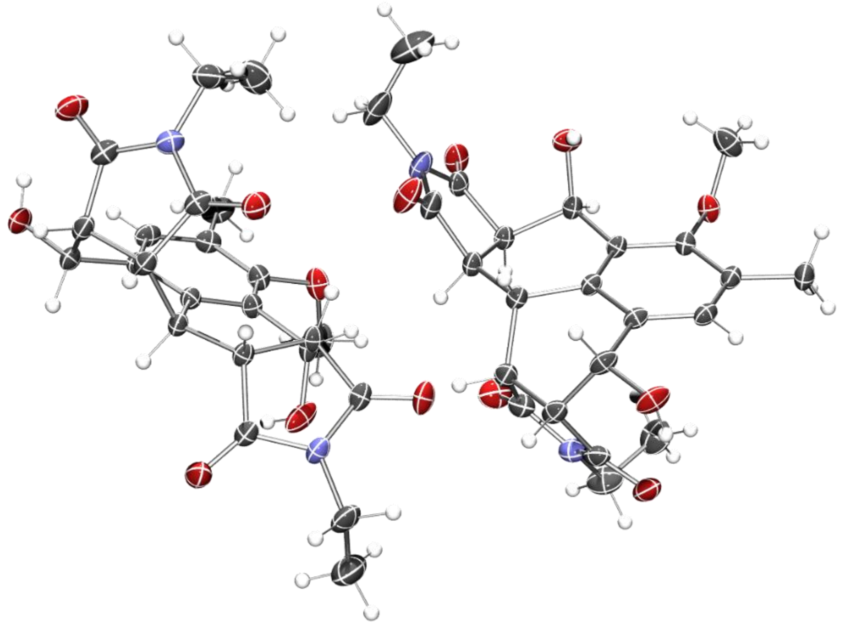

Figure S12 ORTEP diagram of crystal structure of (3a*R*,4*R*,8*R*,8a*R*,11a*R*,11b*R*,11c*R*)-2,10-diethyl-4,8-dihydroxy-5-methoxy-6-methyl-3a,8,8a,11a,11b,11c-hexahydro-1*H*-pyrrolo[3',4':2,3]-naphtho[1,8-*ef*]isoindole-1,3,9,11(2*H*,4*H*,10*H*)-tetraone (**4a**), displaying two independent molecules within the asymmetric unit. Ellipsoids are drawn at 50% probability. Disordered acetonitrile solvate not shown.

XI. UV-VIS Spectra

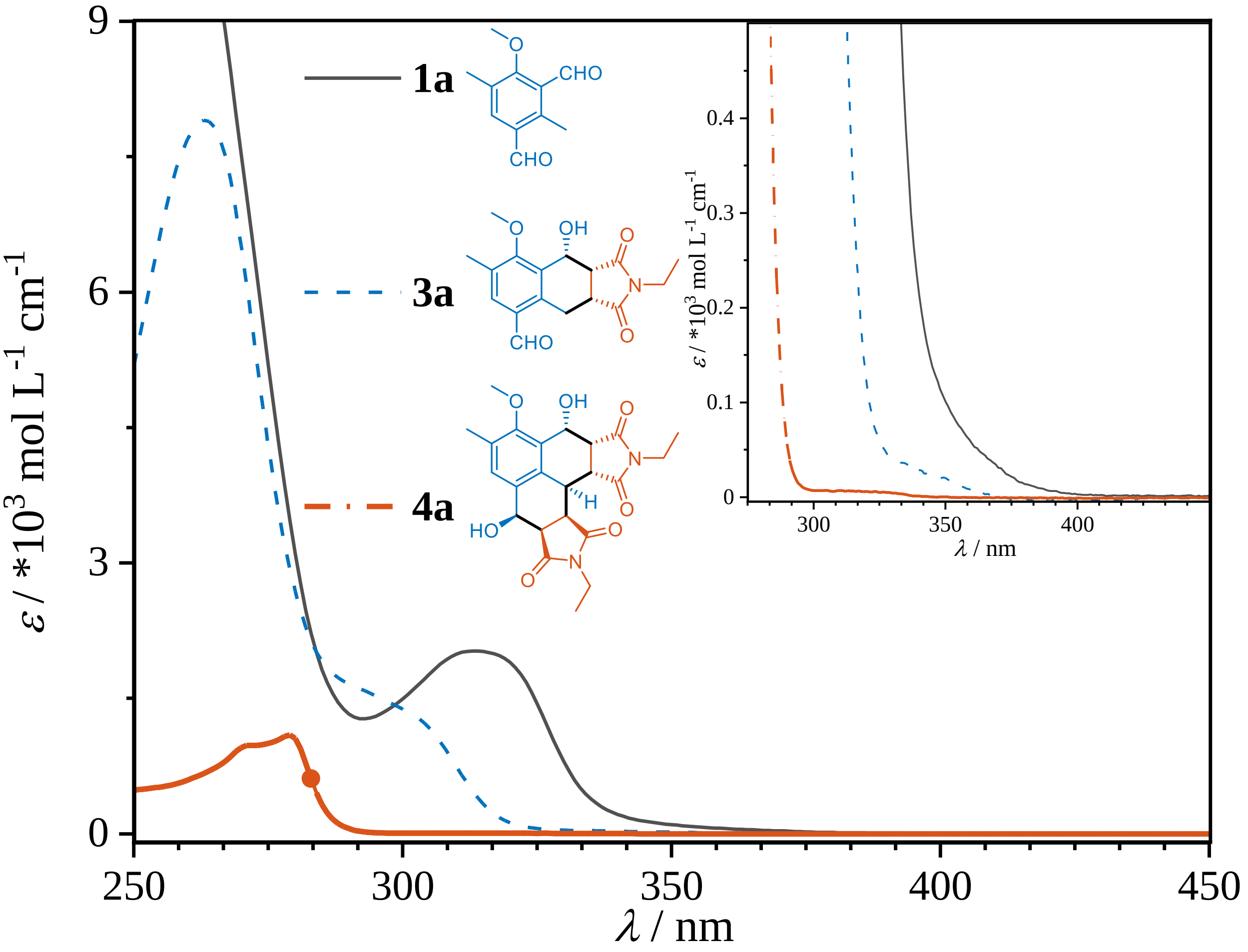

Figure S13 UV-VIS absorption spectra of **1a**, **3a** and **4a** in acetonitrile at 20 °C. Insert: Edge of the n-π* transition, relevant for the photoreactions and wavelength-selectivity.

XII. Fluorescence Spectra and Fluorescence Quantum Yields

Fluorescence quantum yield measurements: The fluorescence spectra and kinetics were measured using a Cary Eclipse Fluorescence Spectrophotometer from Agilent Technologies. The sample preparation and the experimental parameters (excitation wavelength, excitation and emission slit, scan rate and detector voltage are described below for each individual experiment). Samples were prepared in 10 mm quartz fluorescence cuvettes with septum caps and measured at ambient temperature. Baseline measurements were performed on each of the relevant solvents and subtracted from the absorbance and fluorescence intensities. Fluorescence quantum yield measurements were performed using anthracene (φ_f = 0.36, 356 nm, cyclohexane) as a reference. Samples were filtered prior to measurement and fluorescence intensities were measured at a minimum of 5 different concentrations with absorbance less than 0.1 at the fluorescence excitation wavelength. The linear relationship between absorbance (at the fluorescence excitation wavelength) and fluorescence intensity was used to determine the quantum yields.

The utilized settings for the fluorescence quantum yield measurements of the different samples was as follows:

- Anthracene fluorescence emission was measured in cyclohexane - start 366 nm, stop 600 nm, ex. wavelength 356 nm, ex. slit 2.5 nm, ex. slit 5 nm, scan rate 120 nm min^{-1}, data interval 1 nm, 600 V PMT voltage, excitation filter: Auto, emission filter open.
- 4a fluorescence emission was measured in acetonitrile - start 366 nm, stop 600 nm, ex. wavelength 356 nm, ex. slit 2.5 nm, ex. slit 5 nm, scan rate 120 nm min^{-1}, data interval 1 nm, 600 V PMT voltage, excitation filter: Auto, emission filter open.
- 4a fluorescence excitation was measured in acetonitrile - start 280 nm, stop 420 nm, emission wavelength 450 nm, ex. slit 2.5 nm, ex. slit 5 nm, scan rate 120 nm min^{-1}, data interval 1 nm, 600 V PMT voltage, excitation filter: Auto, emission filter open.

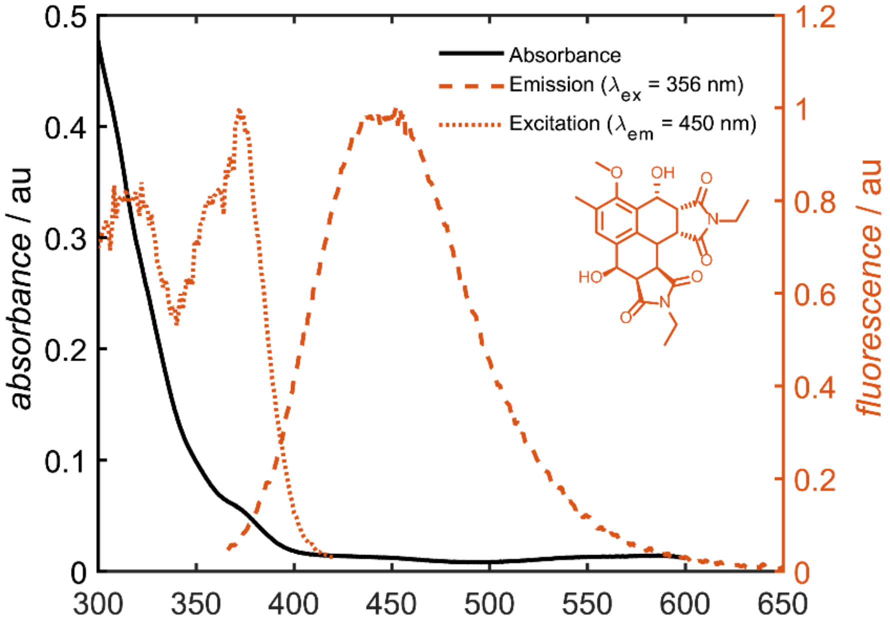

Figure S14 Absorbance, excitation and emission spectra of hexahydro-phenalene-1,6 diol diimide (**4a**) in acetonitrile.

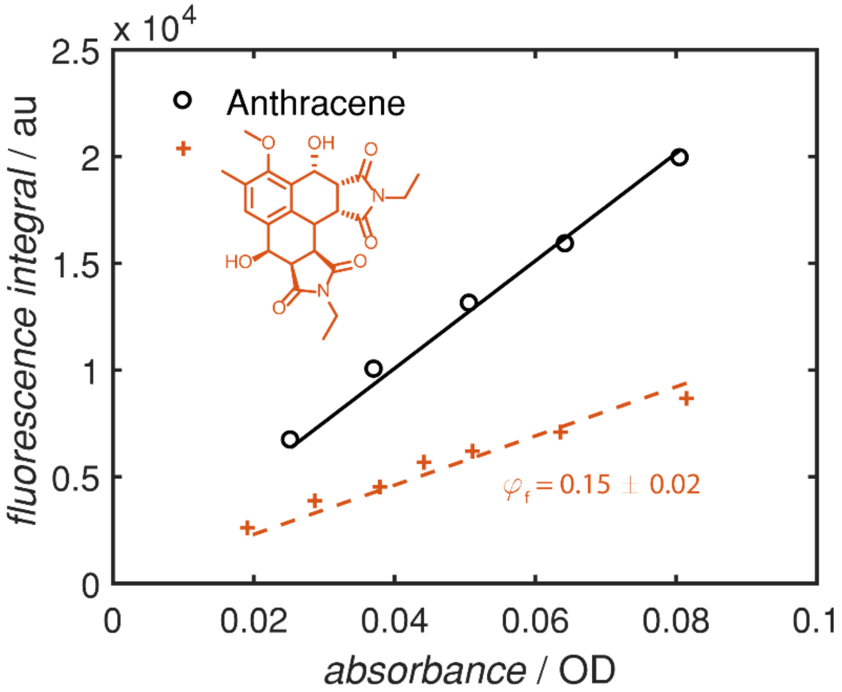

Figure S15 Fluorescence intensities measured at different concentrations indicated by the absorbance recorded at the fluorescence excitation wavelength. Gradients shown here were used to determine the fluorescence quantum yield of compound **4a** ($\varphi_f = 0.15 \pm 0.02$) relative to an anthracene reference standard ($\varphi_f = 0.36$, 356 nm, cyclohexane). Sample **4a** was dispersed in acetonitrile for measurement and anthracene was dispersed in cyclohexane.

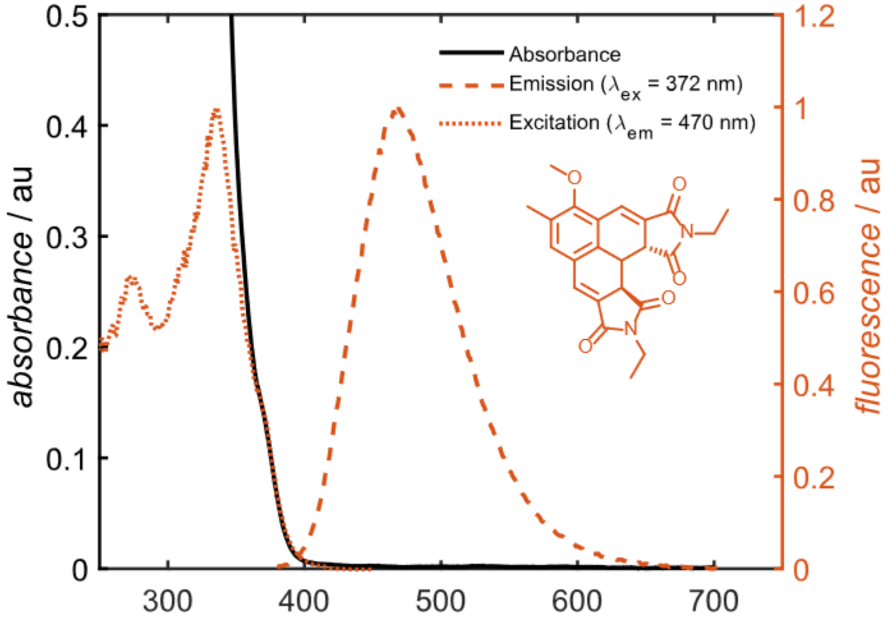

Figure S16 Absorbance, excitation and emission spectra of (11aR,11bR,11cR)-2,10-diethyl-5-methoxy-6-methyl-11b,11c-dihydro-1*H*-pyrrolo[3',4':2,3]naphtho[1,8-*ef*]isoindole-1,3,9,11-(2*H*,10*H*,11a*H*)-tetraone (**5a**) in ACN.

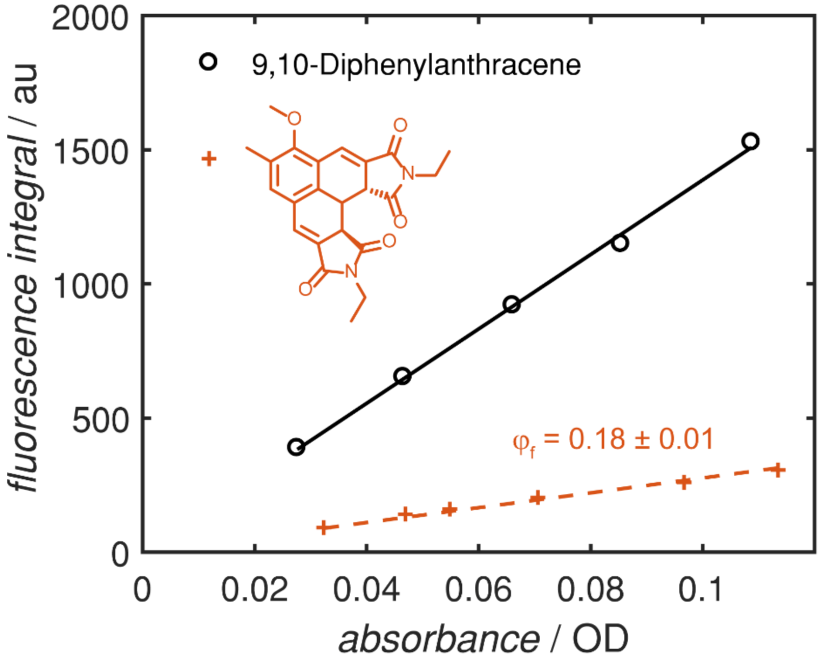

Figure S17 Fluorescence intensities measured at different concentrations indicated by the absorbance recorded at the fluorescence excitation wavelength. Gradients shown here were used to determine the fluorescence quantum yield of compound **5a** (φ_f = 0.18 ± 0.01) relative to an 9,10-diphenylanthracene reference standard (φ_f = 1.00, 372 nm, cyclohexane). Sample **5a** was dispersed in ACN for measurement and 9,10-diphenylanthracene was dispersed in cyclohexane.

XIII. NMR Spectra

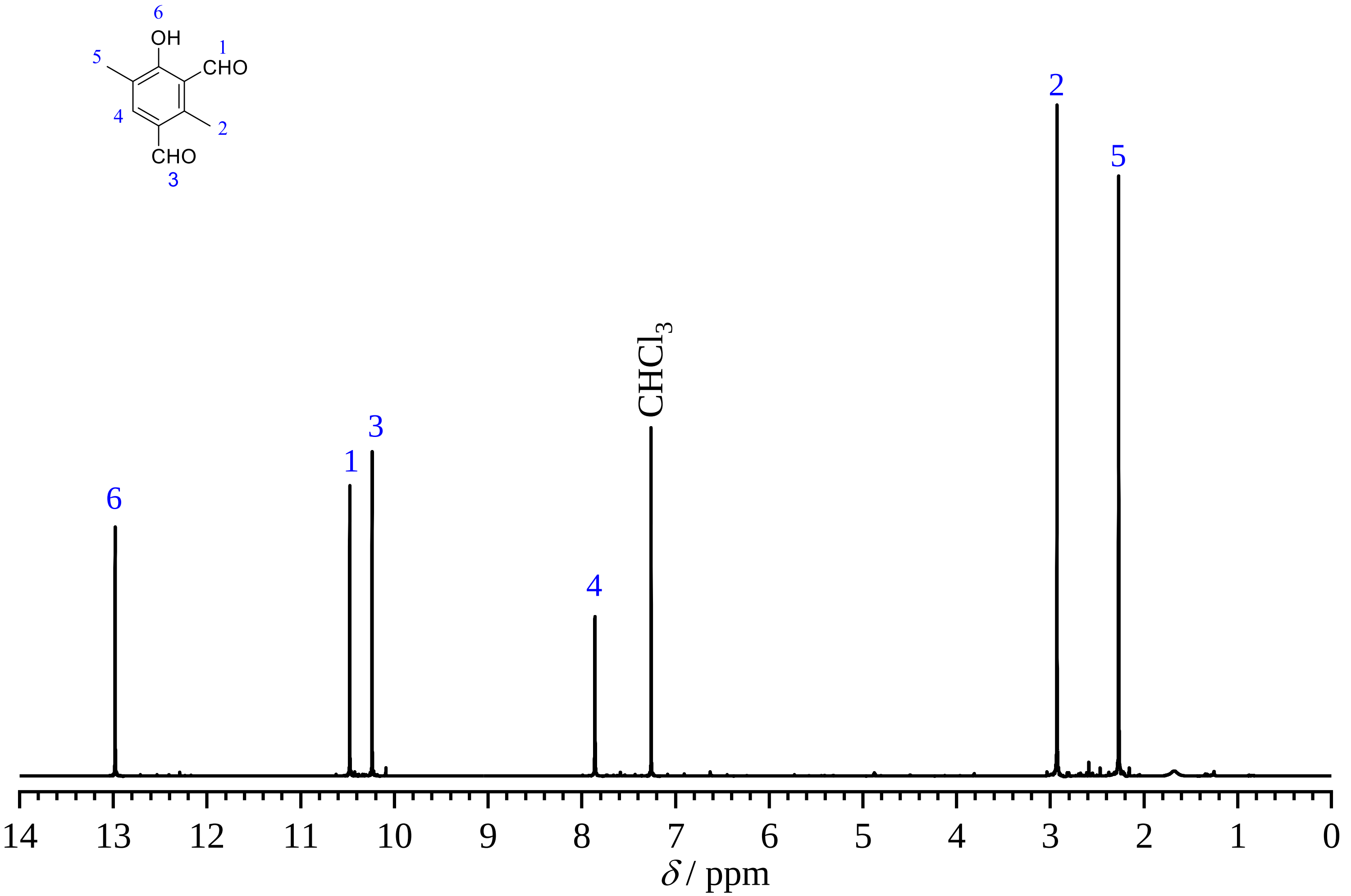

Figure S18 ^{1}H NMR spectrum of 4-hydroxy-2,5-dimethylisophthalaldehyde recorded in $CDCl_3$ and assigned resonances.

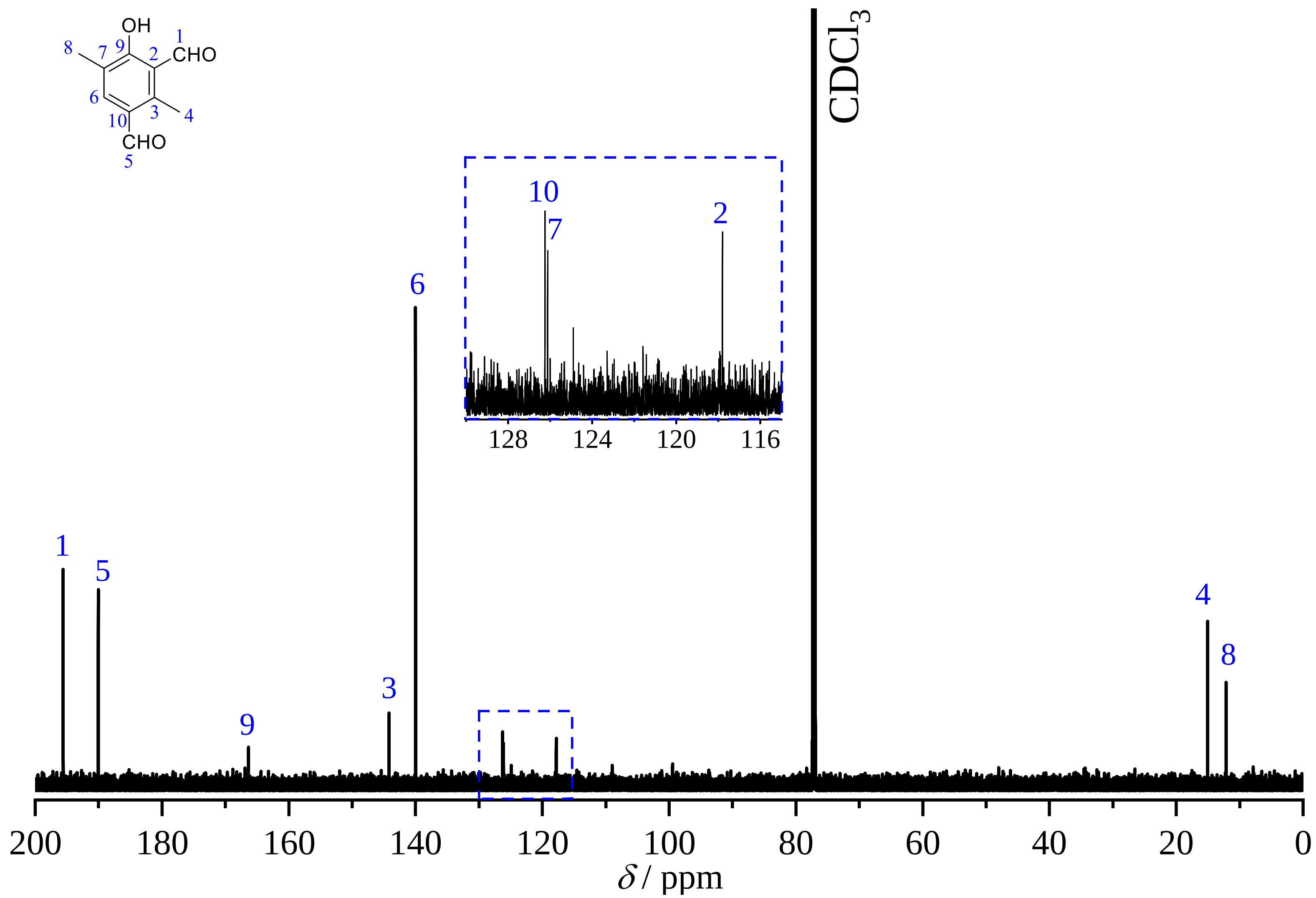

Figure S19 ^{13}C NMR spectrum of 4-hydroxy-2,5-dimethylisophthalaldehyde recorded in $CDCl_3$ and assigned resonances.

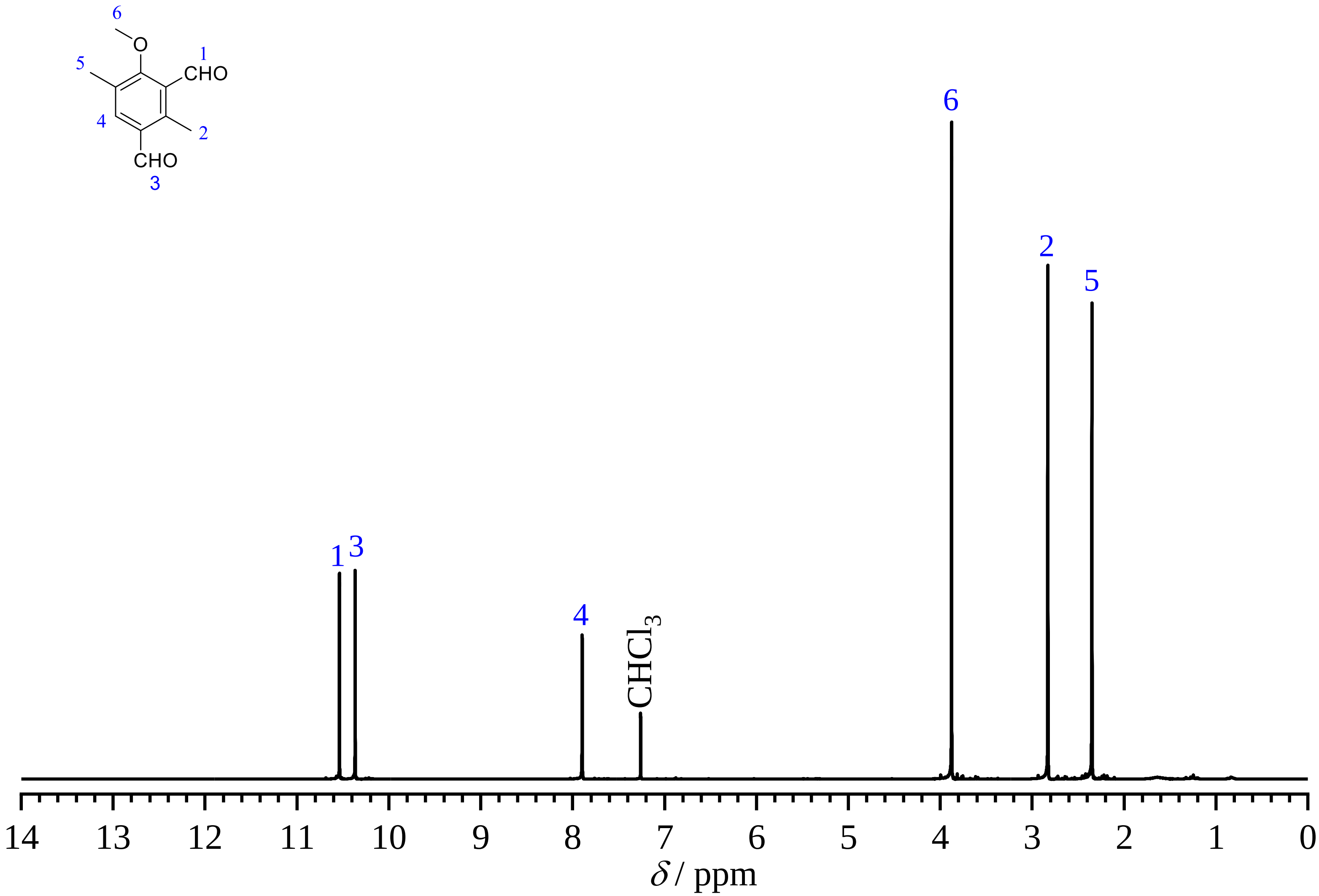

Figure S20 ^{1}H NMR spectrum of 4-methoxy-2,5-dimethylisophthalaldehyde (**1a**) recorded in $CDCl_3$ and assigned resonances.

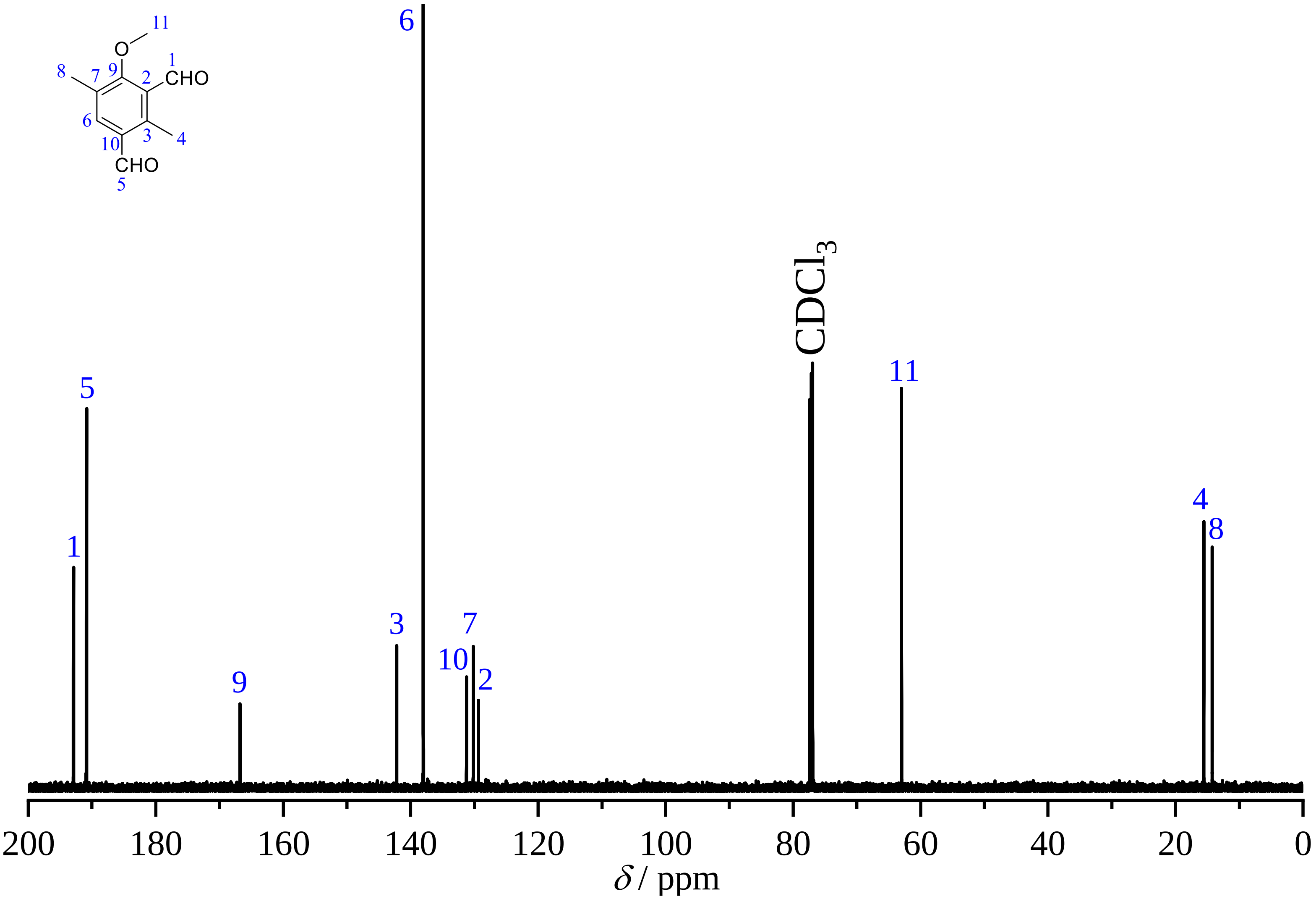

Figure S21 ^{13}C NMR spectrum of 4-methoxy-2,5-dimethylisophthalaldehyde (**1a**) recorded in $CDCl_3$ and assigned resonances.

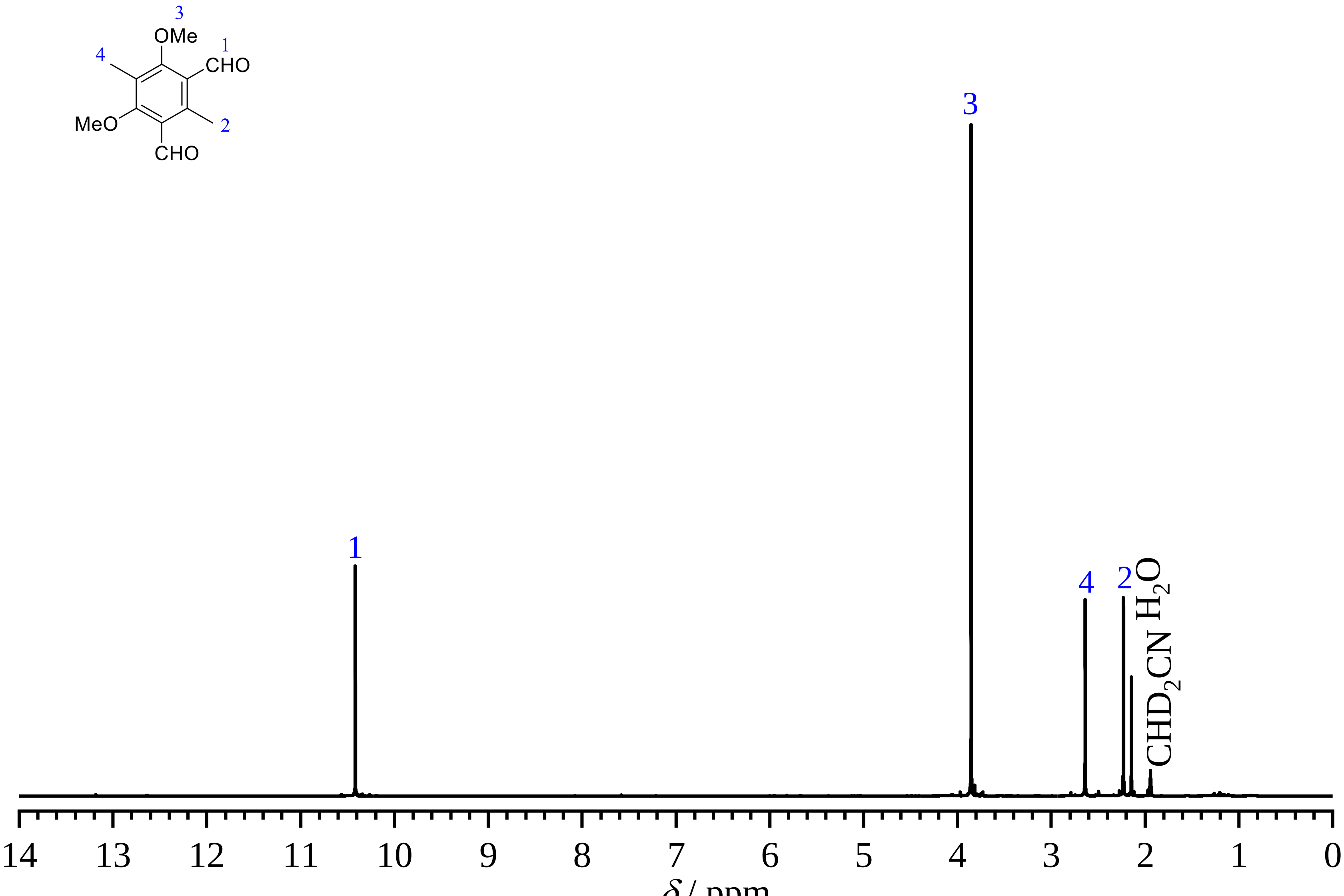

Figure S22 ^{1}H NMR spectrum of 4,6-dimethoxy-2,5-dimethylisophthalaldehyde (**1b**) recorded in $CDCl_3$ and assigned resonances.

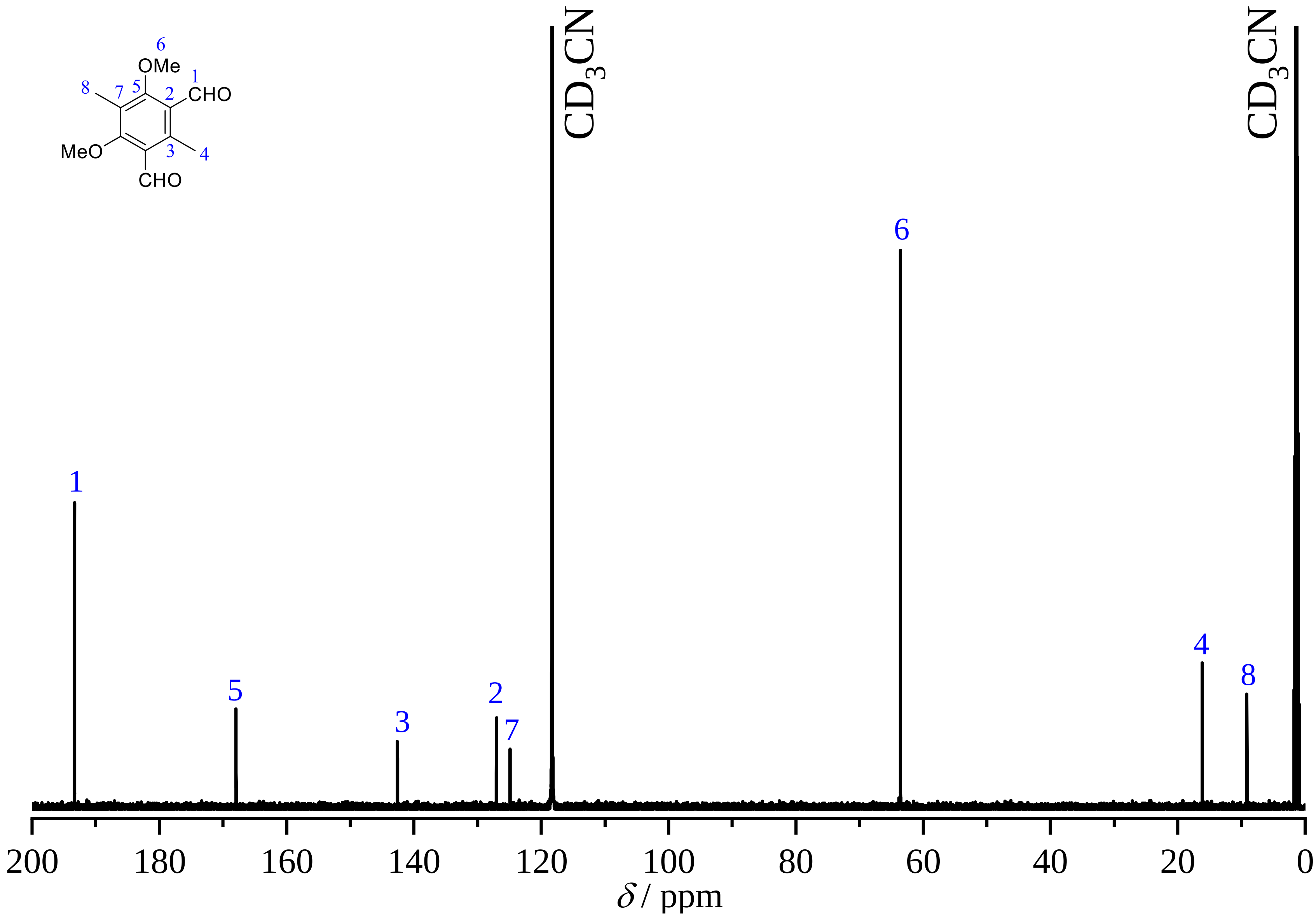

Figure S23 ^{13}C NMR spectrum of 4-methoxy-2,5-dimethylisophthalaldehyde (**1b**) recorded in $CDCl_3$ and assigned resonances.

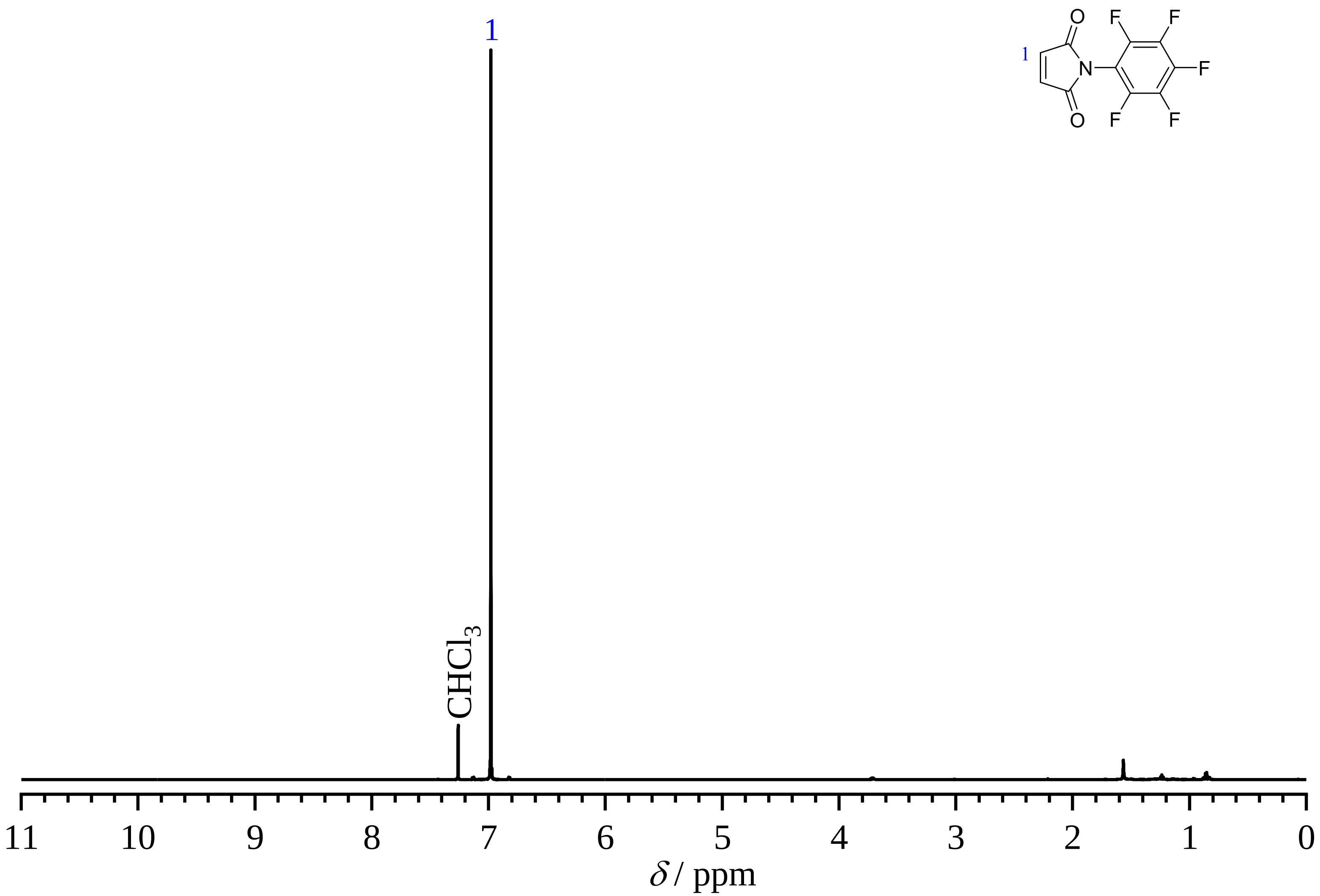

Figure S24 ^{1}H NMR spectrum and assigned resonances of 1-(perfluorophenyl)-1*H*-pyrrole-2,5-dione (**2c**) recorded in $CDCl_3$.

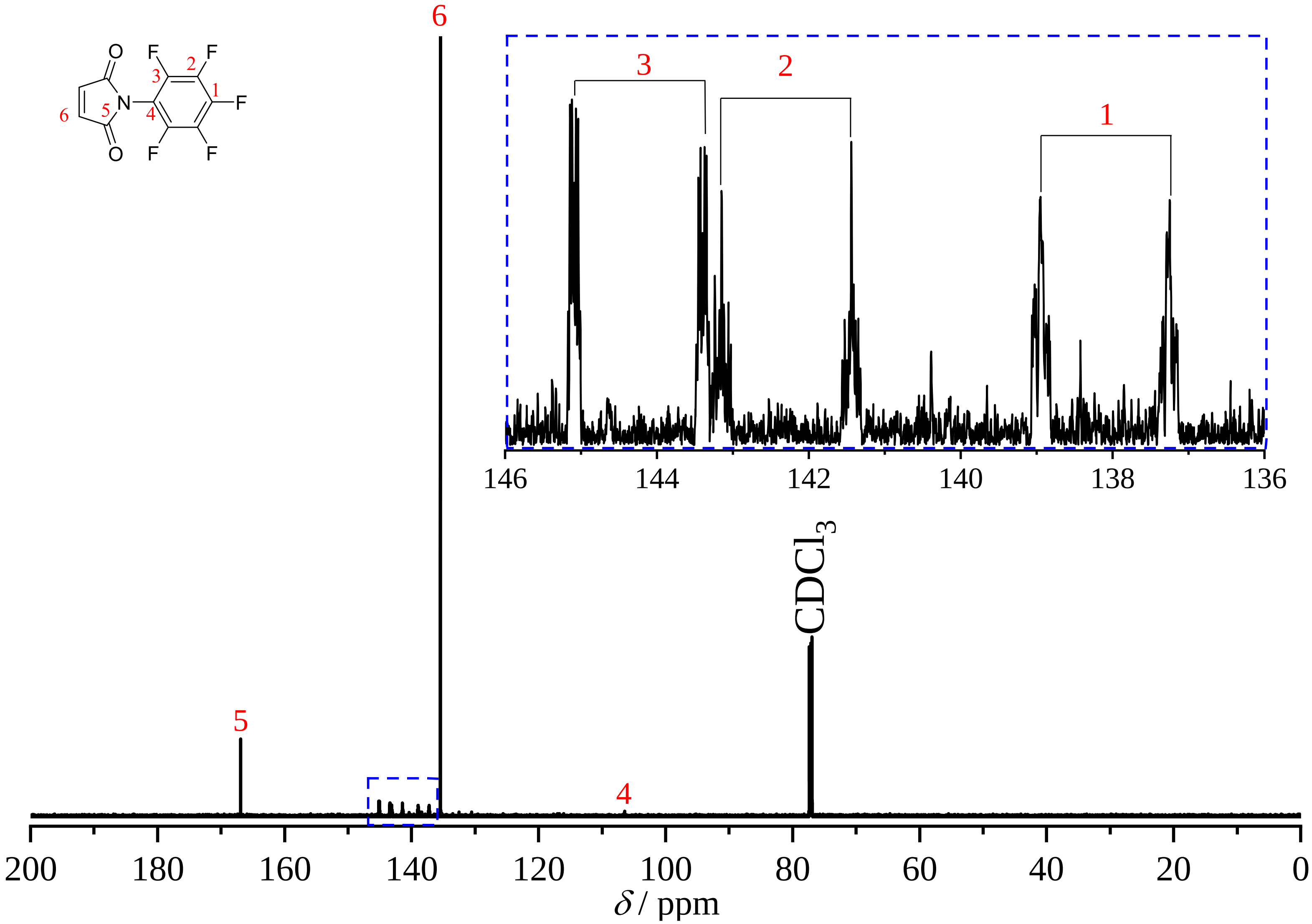

Figure S25 ^{13}C NMR spectrum and assigned resonances of 1-(perfluorophenyl)-1*H*-pyrrole-2,5-dione (**2c**) recorded in $CDCl_3$.

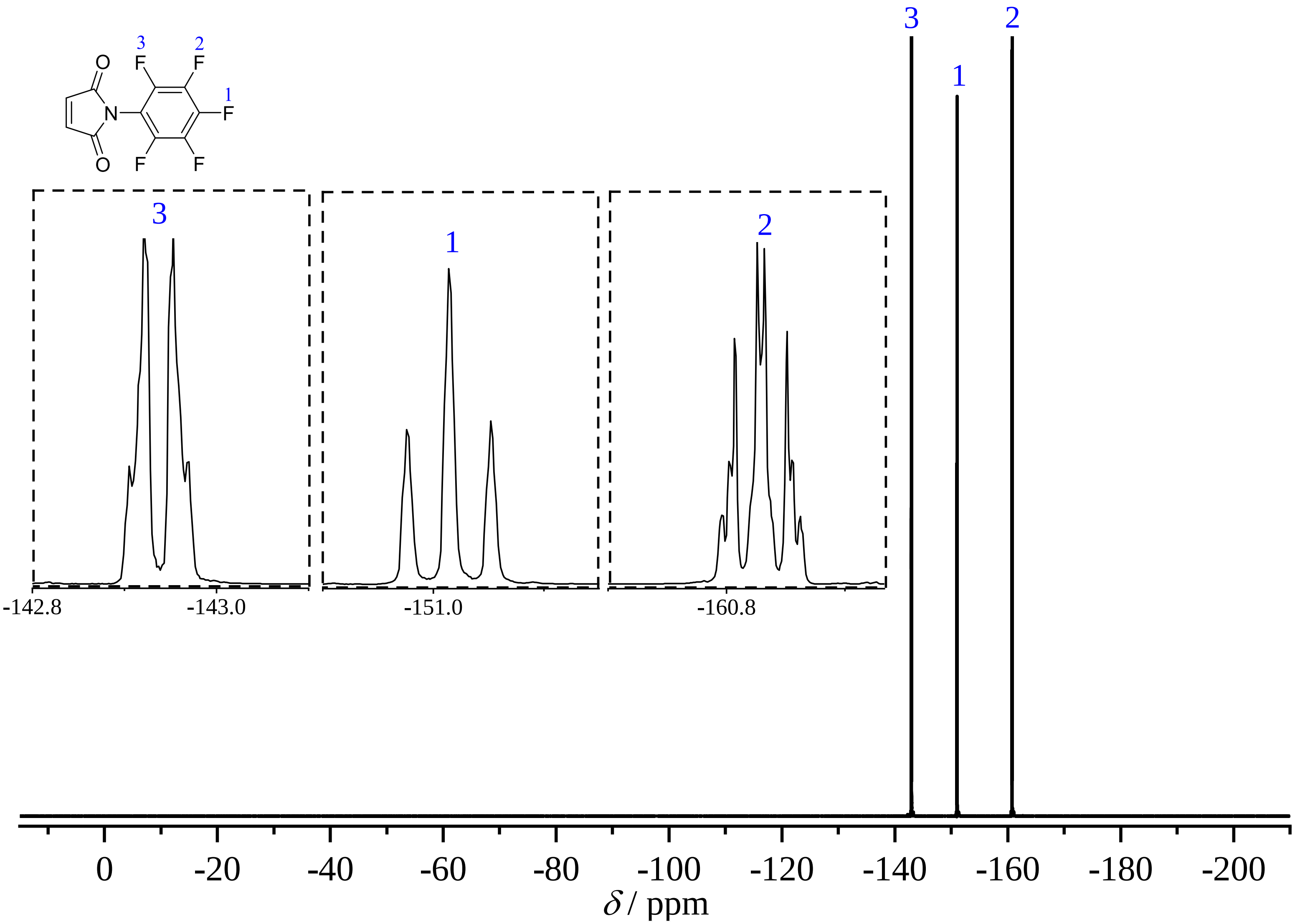

Figure S26 ^{19}F NMR spectrum and assigned resonances of 1-(perfluorophenyl)-1*H*-pyrrole-2,5-dione recorded (**2c**) in $CDCl_3$.

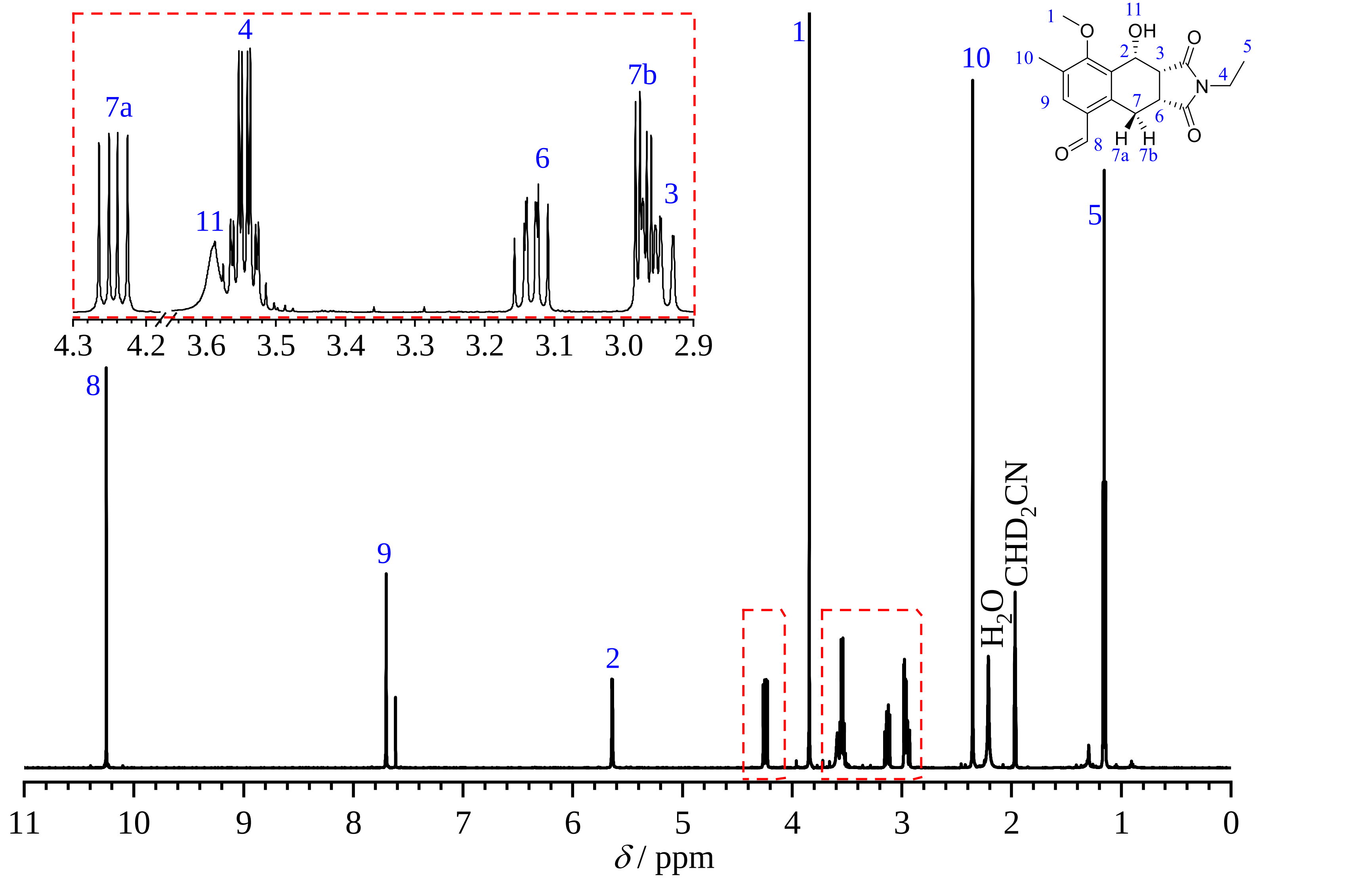

Figure S27 ^{1}H NMR spectrum of (3a*R*,9*R*,9a*R*)-2-ethyl-9-hydroxy-8-methoxy-7-methyl-1,3-dioxo-2,3,3a,4,9,9a-hexahydro-1*H*-benzo[*f*]isoindole-5-carbaldehyde (**3a**) recorded in CD_3CN and assigned resonances.

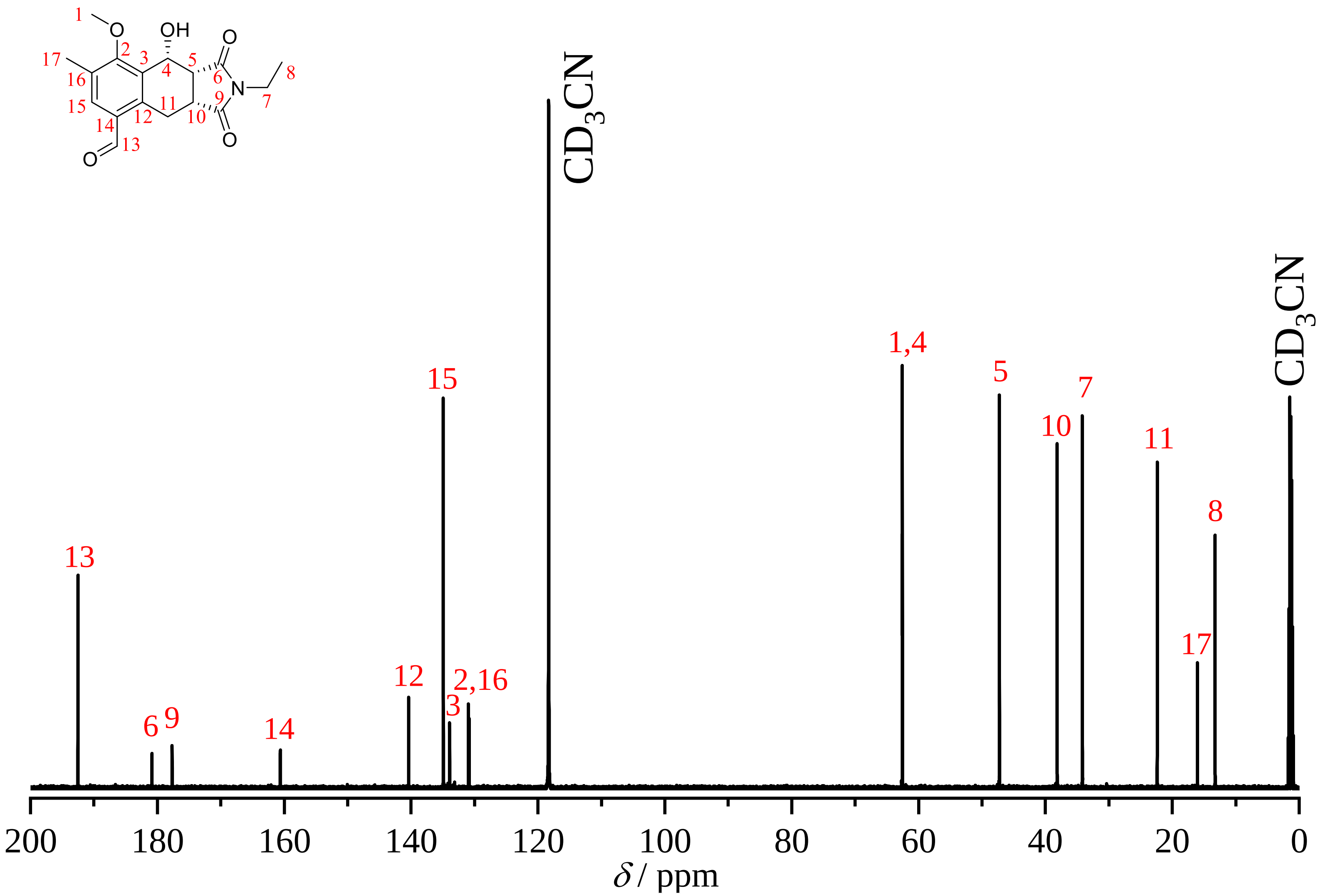

Figure S28 ^{13}C NMR spectrum of (3a*R*,9*R*,9a*R*)-2-ethyl-9-hydroxy-8-methoxy-7-methyl-1,3-dioxo-2,3,3a,4,9,9a-hexahydro-1*H*-benzo[*f*]isoindole-5-carbaldehyde (**3a**) recorded in CD_3CN and assigned resonances.

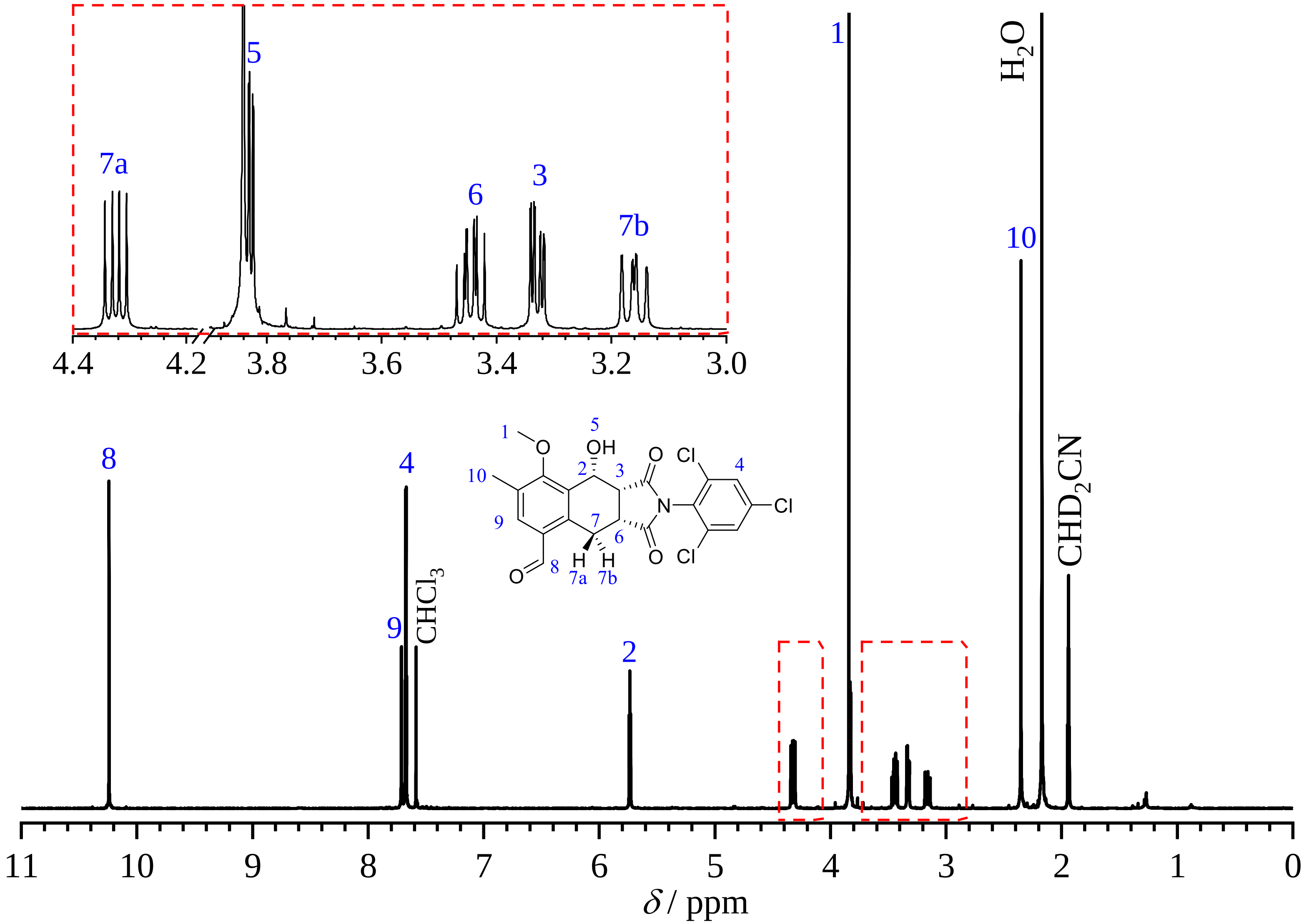

Figure S29 ^{1}H NMR spectrum of (3a*R*,9*R*,9a*R*)-9-hydroxy-8-methoxy-7-methyl-1,3-dioxo-2-(2,4,6-trichlorophenyl)-2,3,3a,4,9,9a-hexahydro-1*H*-benzo[*f*]isoindole-5-carbaldehyde **(*endo*-3b)** recorded in CD_3CN and assigned resonances.

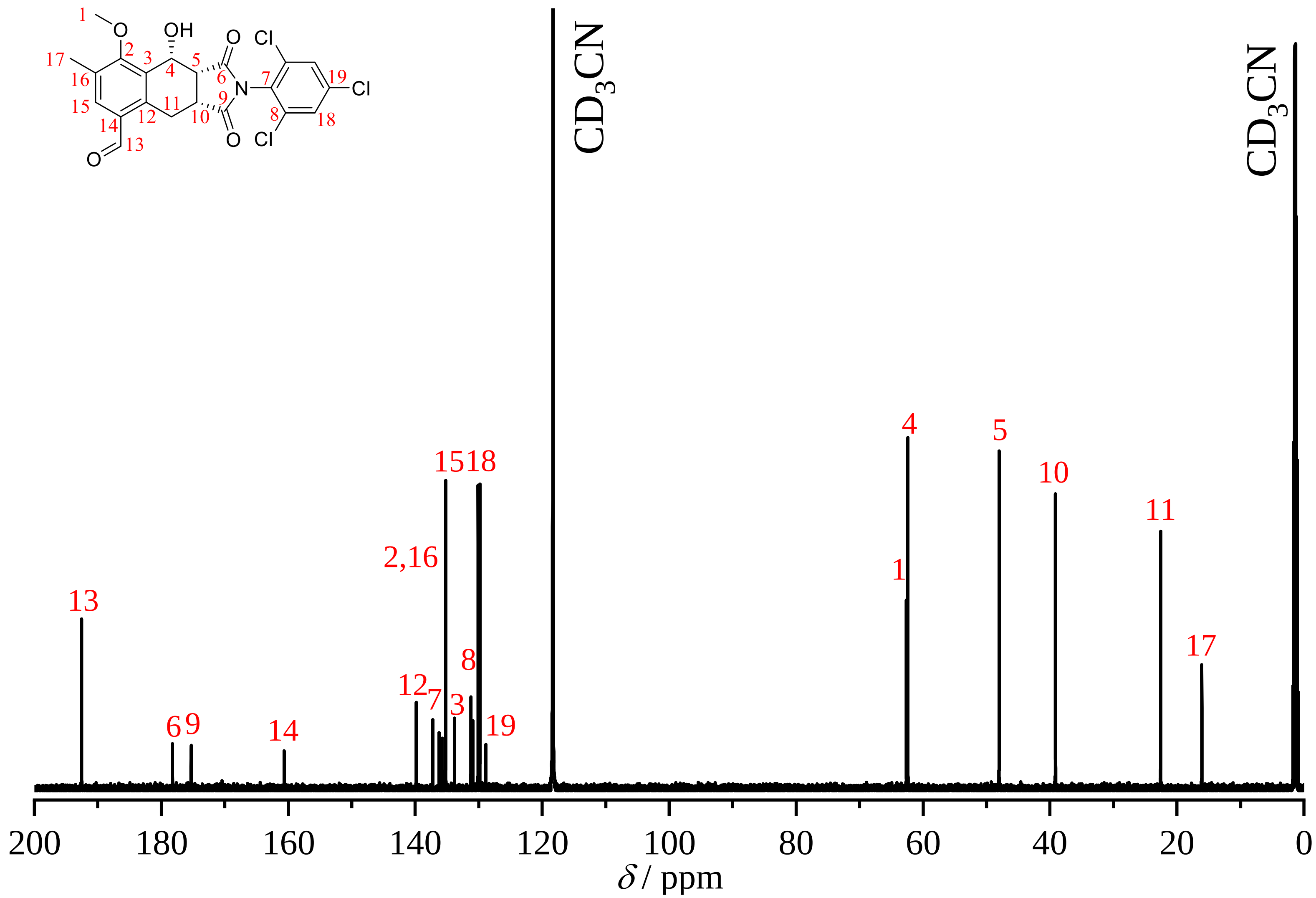

Figure S30 ^{13}C NMR spectrum of (3a*R*,9*R*,9a*R*)-9-hydroxy-8-methoxy-7-methyl-1,3-dioxo-2-(2,4,6-trichlorophenyl)-2,3,3a,4,9,9a-hexahydro-1*H*-benzo[*f*]isoindole-5-carbaldehyde (***endo***-3b) recorded in CD_3CN and assigned resonances.

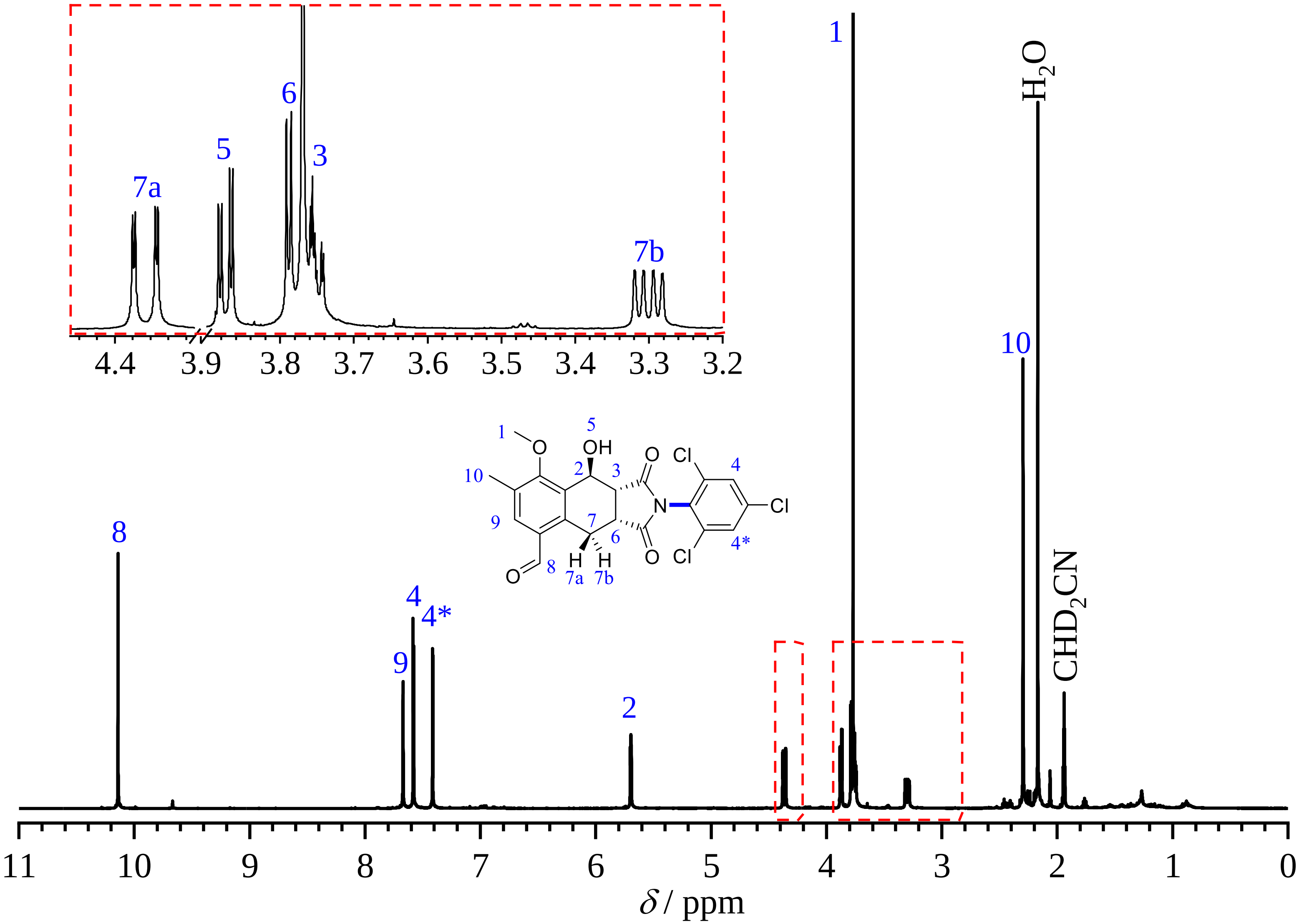

Figure S31 ^{1}H NMR spectrum of (3a*R*,9*S*,9a*R*)-9-hydroxy-8-methoxy-7-methyl-1,3-dioxo-2-(2,4,6-trichlorophenyl)-2,3,3a,4,9,9a-hexahydro-1*H*-benzo[*f*]isoindole-5-carbaldehyde **(*exo*-3b)** recorded in CD_3CN and assigned resonances.

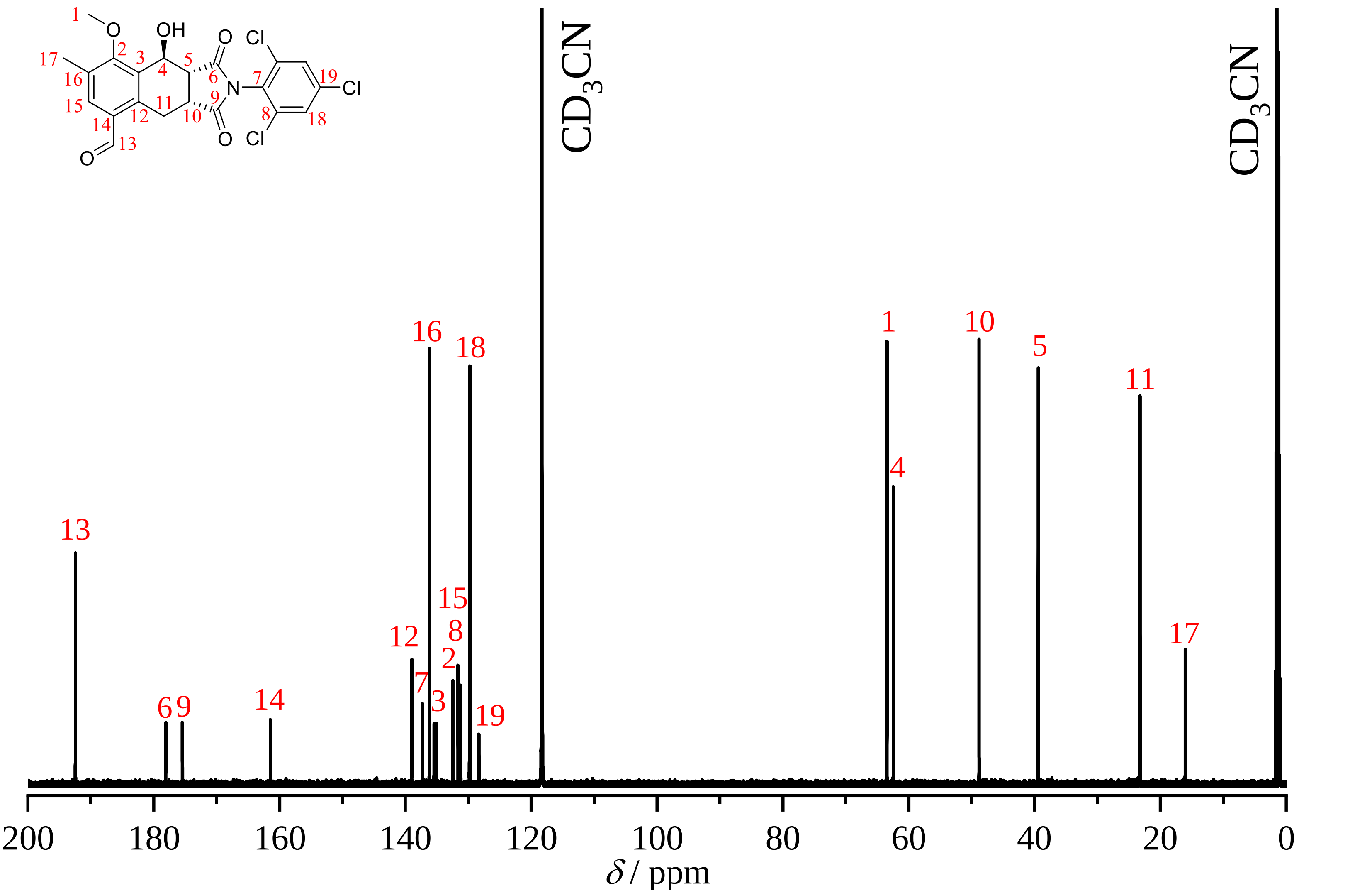

Figure S32 ^{13}C NMR spectrum of (3a*R*,9*S*,9a*R*)-9-hydroxy-8-methoxy-7-methyl-1,3-dioxo-2-(2,4,6-trichlorophenyl)-2,3,3a,4,9,9a-hexahydro-1*H*-benzo[*f*]isoindole-5-carbaldehyde **(*exo*-3b)** recorded in CD_3CN and assigned resonances.

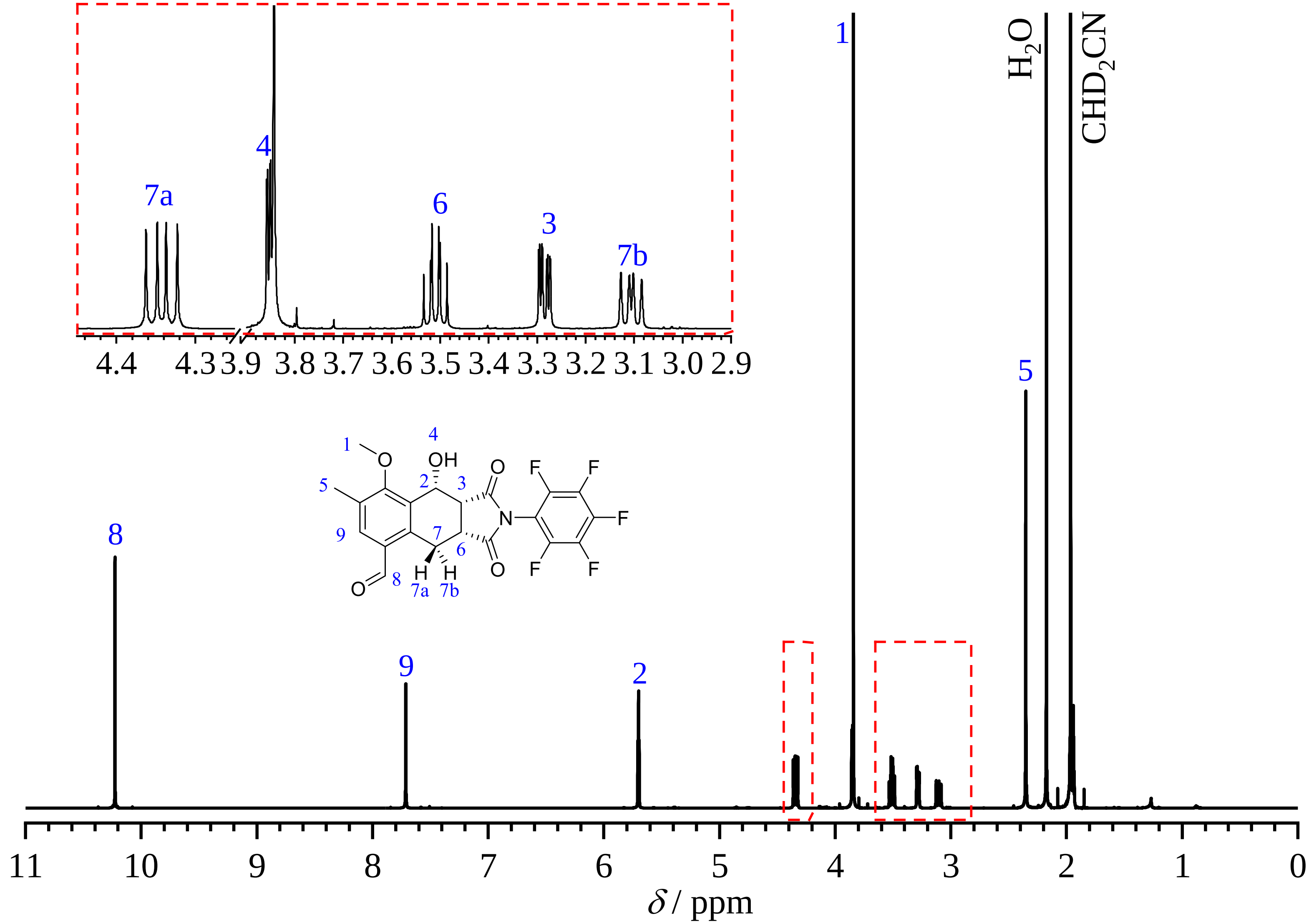

Figure S33 ^{1}H NMR spectrum of (3a*R*,9*R*,9a*R*)-9-hydroxy-8-methoxy-7-methyl-1,3-dioxo-2-(perfluorophenyl)-2,3,3a,4,9,9a-hexahydro-1*H*-benzo[*f*]isoindole-5-carbaldehyde (**3c**) recorded in CD_3CN and assigned resonances.

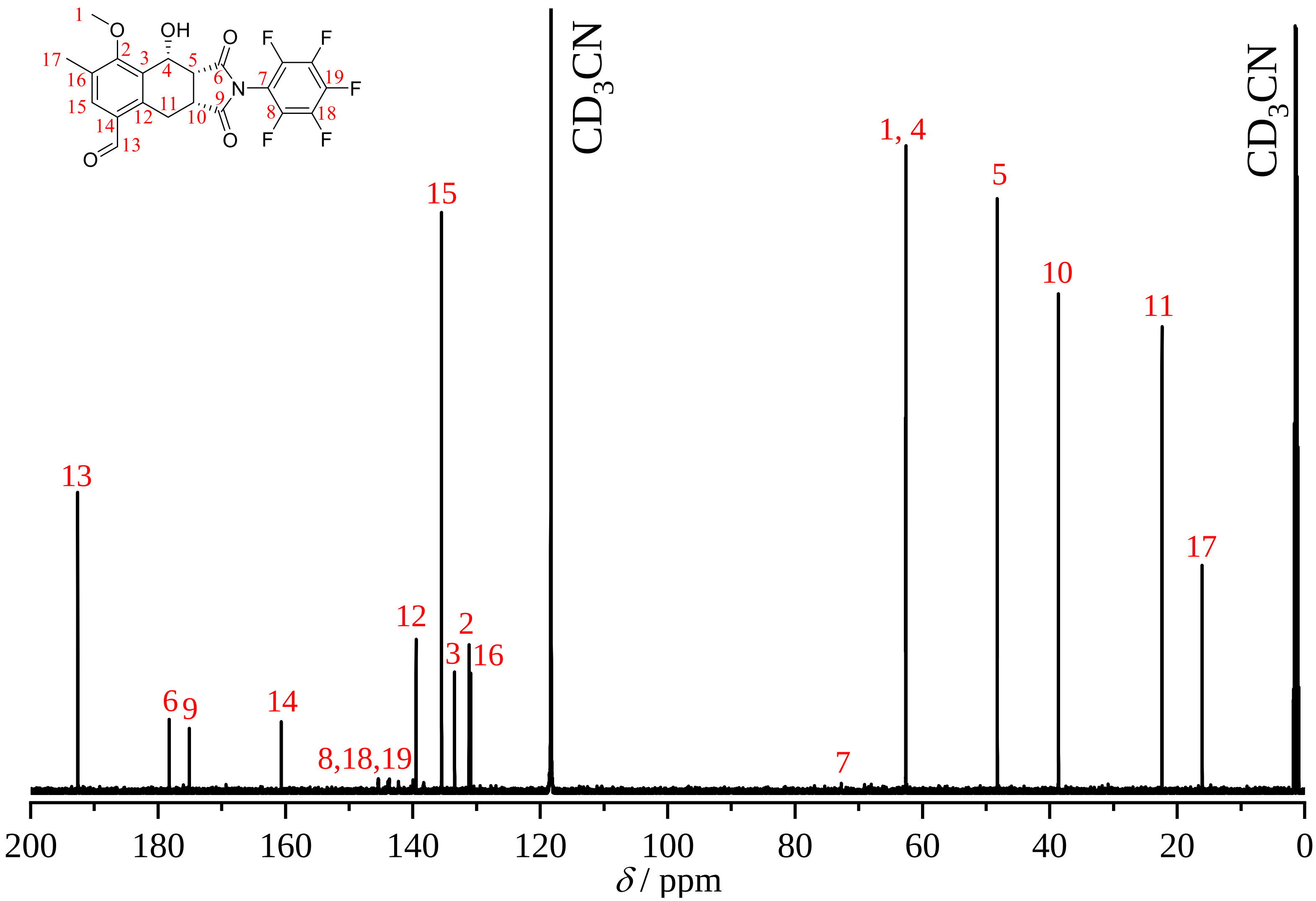

Figure S34 ^{13}C NMR spectrum of (3a*R*,9*R*,9a*R*)-9-hydroxy-8-methoxy-7-methyl-1,3-dioxo-2-(perfluorophenyl)-2,3,3a,4,9,9a-hexahydro-1*H*-benzo[*f*]isoindole-5-carbaldehyde (**3c**) recorded in CD_3CN and assigned resonances.

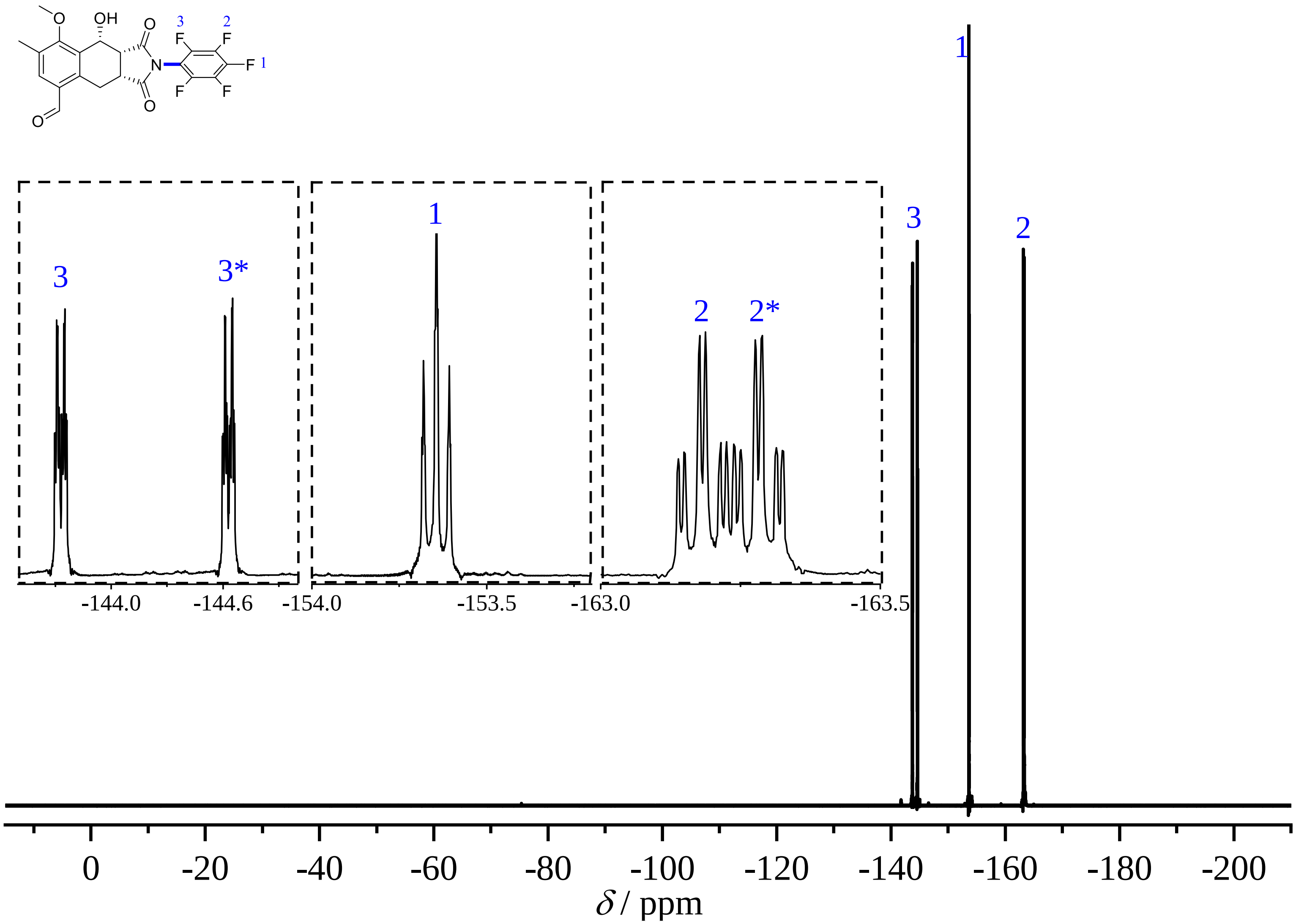

Figure S35 ^{19}F NMR spectrum of (3a*R*,9*R*,9a*R*)-9-hydroxy-8-methoxy-7-methyl-1,3-dioxo-2-(perfluorophenyl)-2,3,3a,4,9,9a-hexahydro-1*H*-benzo[*f*]isoindole-5-carbaldehyde (**3c**) recorded in CD_3CN and assigned resonances.

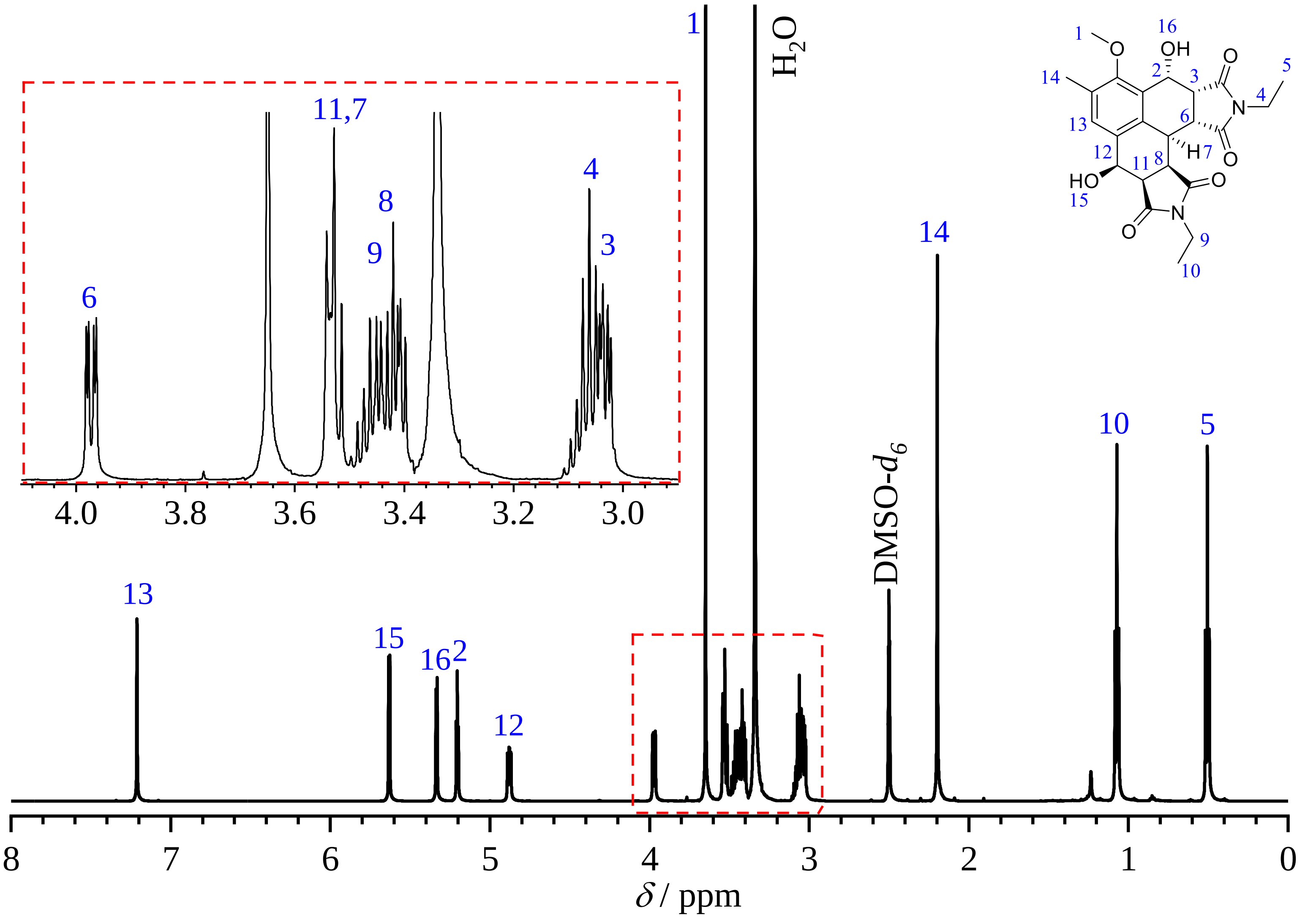

Figure S36 ^{1}H NMR spectrum of 3a*R*,4*R*,8*R*,8a*R*,11a*R*,11b*R*,11c*R*)-2,10-diethyl-4,8-dihydroxy-5-methoxy-6-methyl-3a,8,8a,11a,11b,11c-hexahydro-1*H*-pyrrolo[3',4':2,3]naphtho[1,8-*ef*]isoindole-1,3,9,11(2*H*,4*H*,10*H*)-tetraone (**4a**) in DMSO-d_6 and assigned resonances.

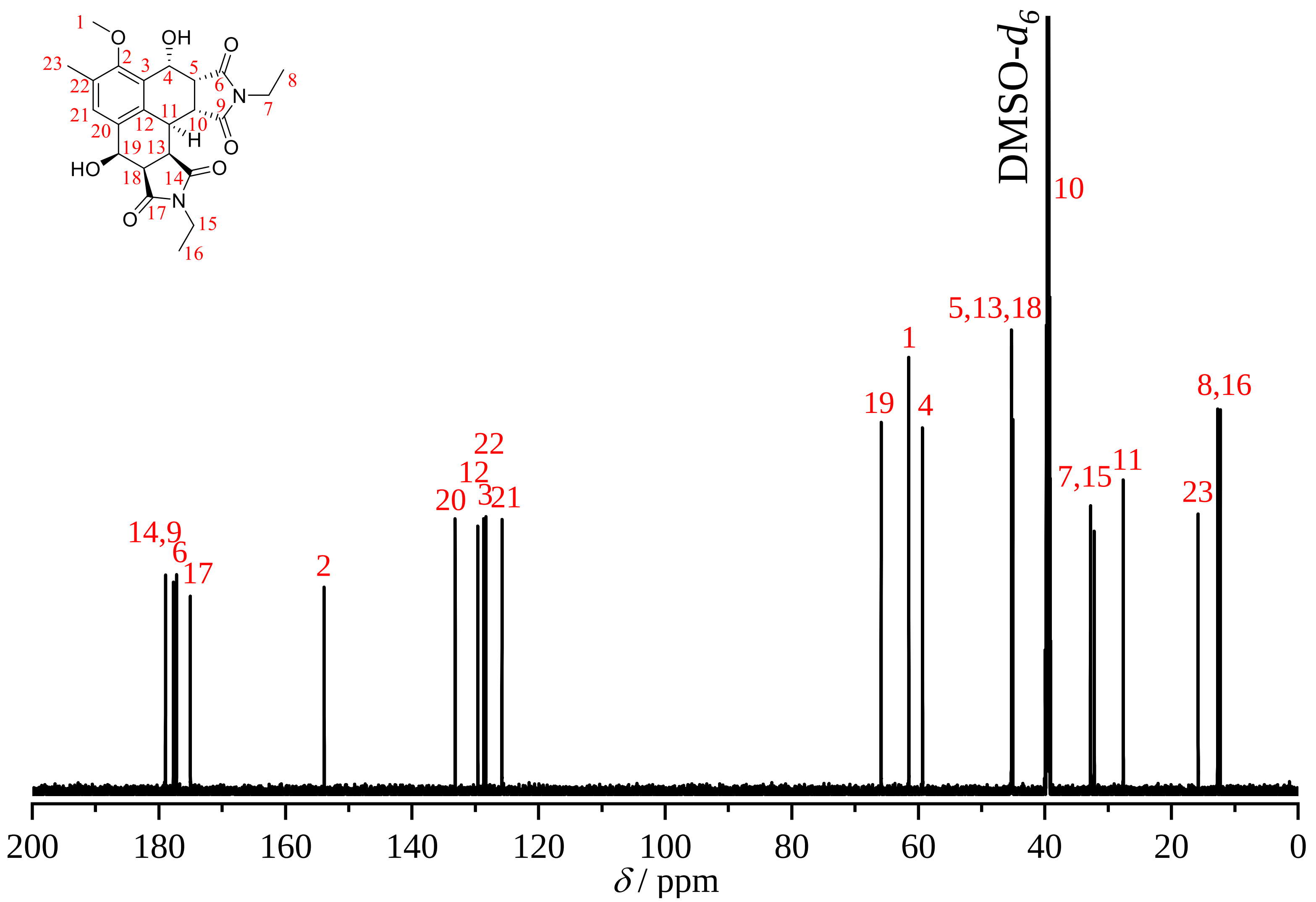

Figure S37 ^{13}C NMR spectrum of (3a*R*,4*R*,8*R*,8a*R*,11a*R*,11b*R*,11c*R*)-2,10-diethyl-4,8-dihydroxy-5-methoxy-6-methyl-3a,8,8a,11a,11b,11c-hexahydro-1*H*-pyrrolo[3',4':2,3]naphtho[1,8-*ef*]isoindole-1,3,9,11(2*H*,4*H*,10*H*)-tetraone (**4a**) recorded in DMSO-d_6 and assigned resonances.

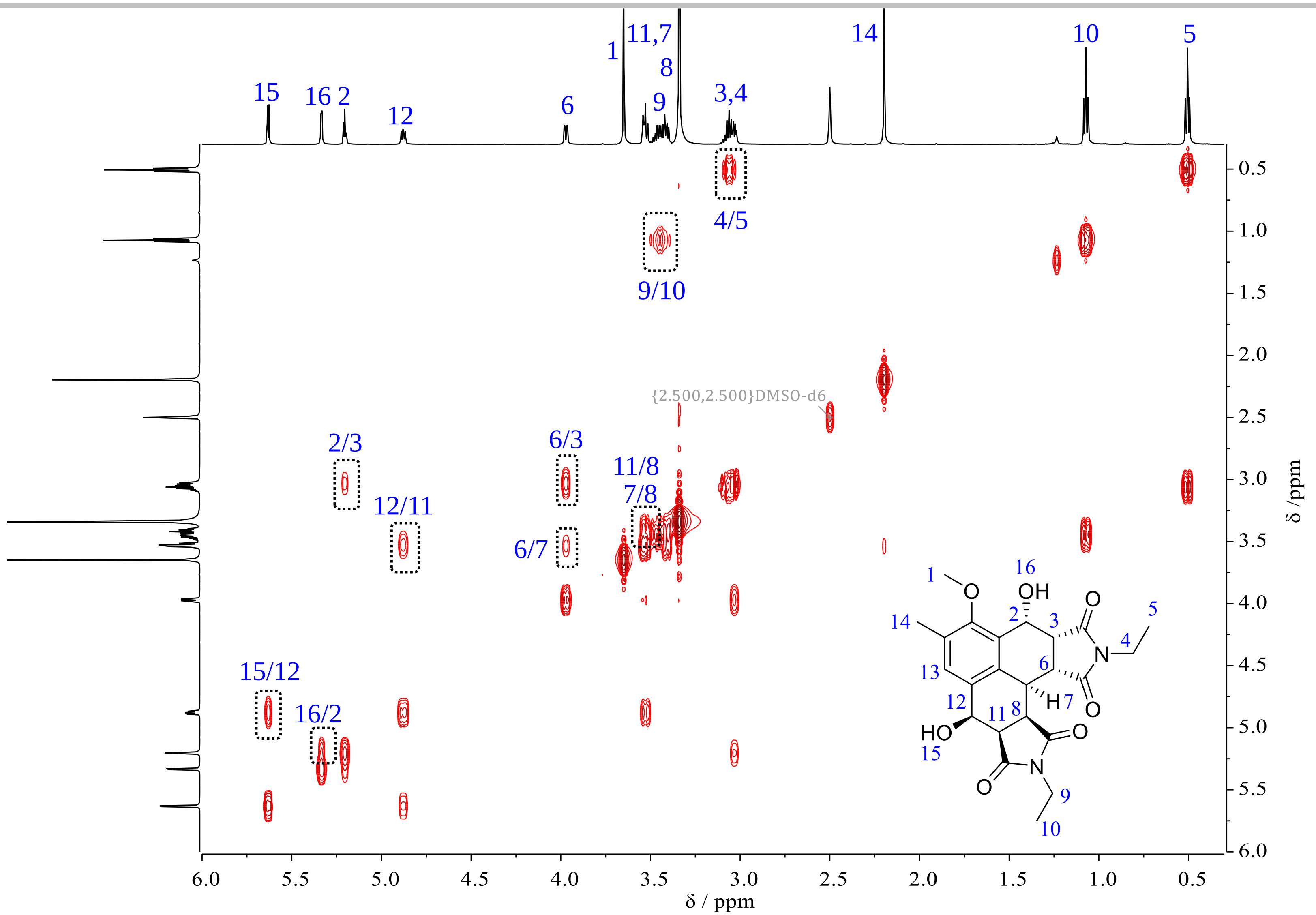

Figure S38 ^{1}H/^{1}H-COSY spectrum of (3a*R*,4*R*,8*R*,8a*R*,11a*R*,11b*R*,11c*R*)-2,10-diethyl-4,8-dihydroxy-5-methoxy-6-methyl-3a,8,8a,11a,11b,11c-hexahydro-1*H*-pyrrolo[3',4':2,3]naphtho[1,8-*ef*]isoindole-1,3,9,11(2*H*,4*H*,10*H*)-tetraone (**4a**) recorded in DMSO-d_6 and assigned resonances.

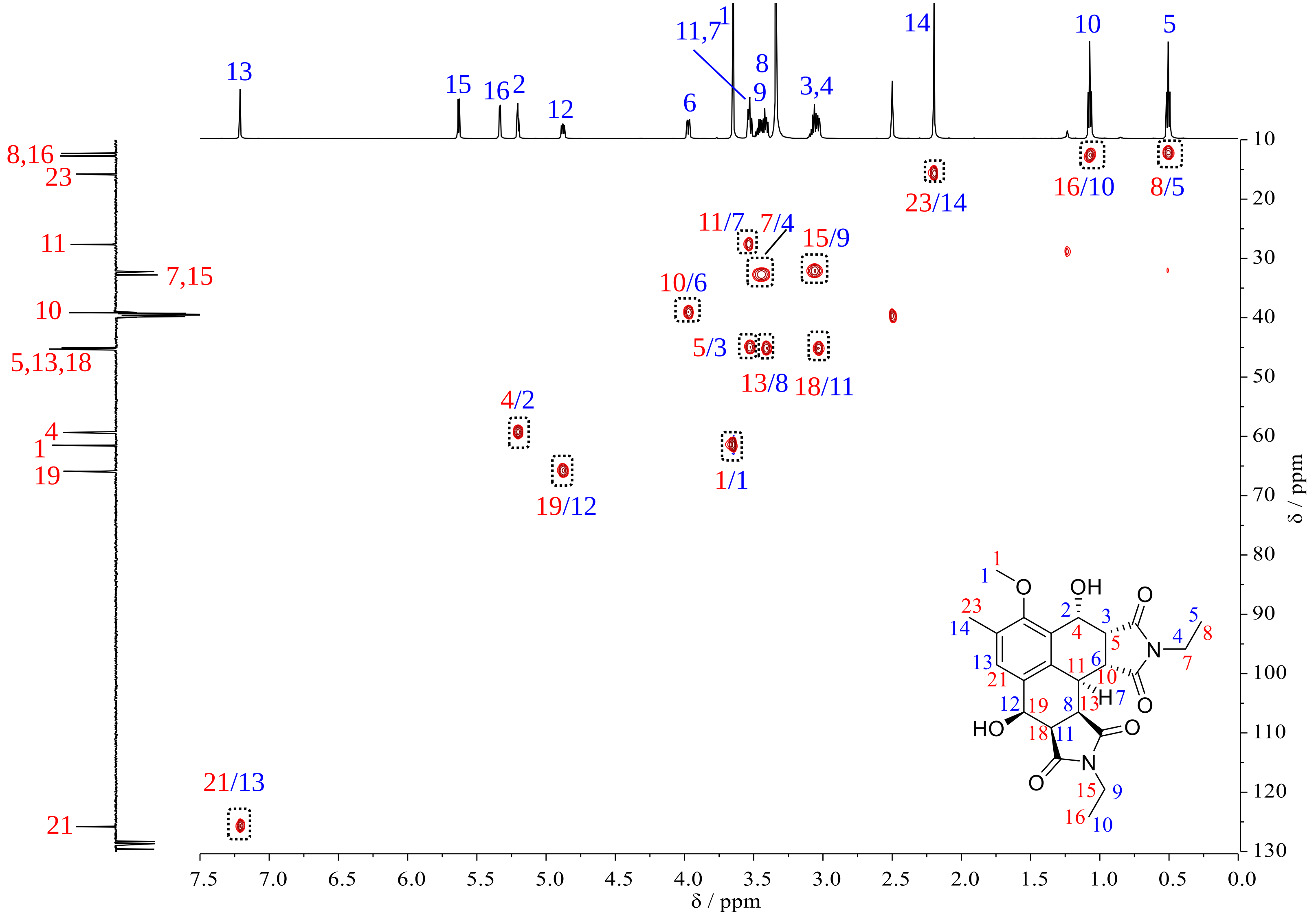

Figure S39 $^{1}H/^{13}H$-HSQC spectrum of (3a*R*,4*R*,8*R*,8a*R*,11a*R*,11b*R*,11c*R*)-2,10-diethyl-4,8-dihydroxy-5-methoxy-6-methyl-3a,8,8a,11a,11b,11c-hexahydro-1*H*-pyrrolo[3',4':2,3]naphtho[1,8-*ef*]isoindole-1,3,9,11(2*H*,4*H*,10*H*)-tetraone (**4a**) recorded in DMSO-d_6 and assigned ^{1}J couplings.

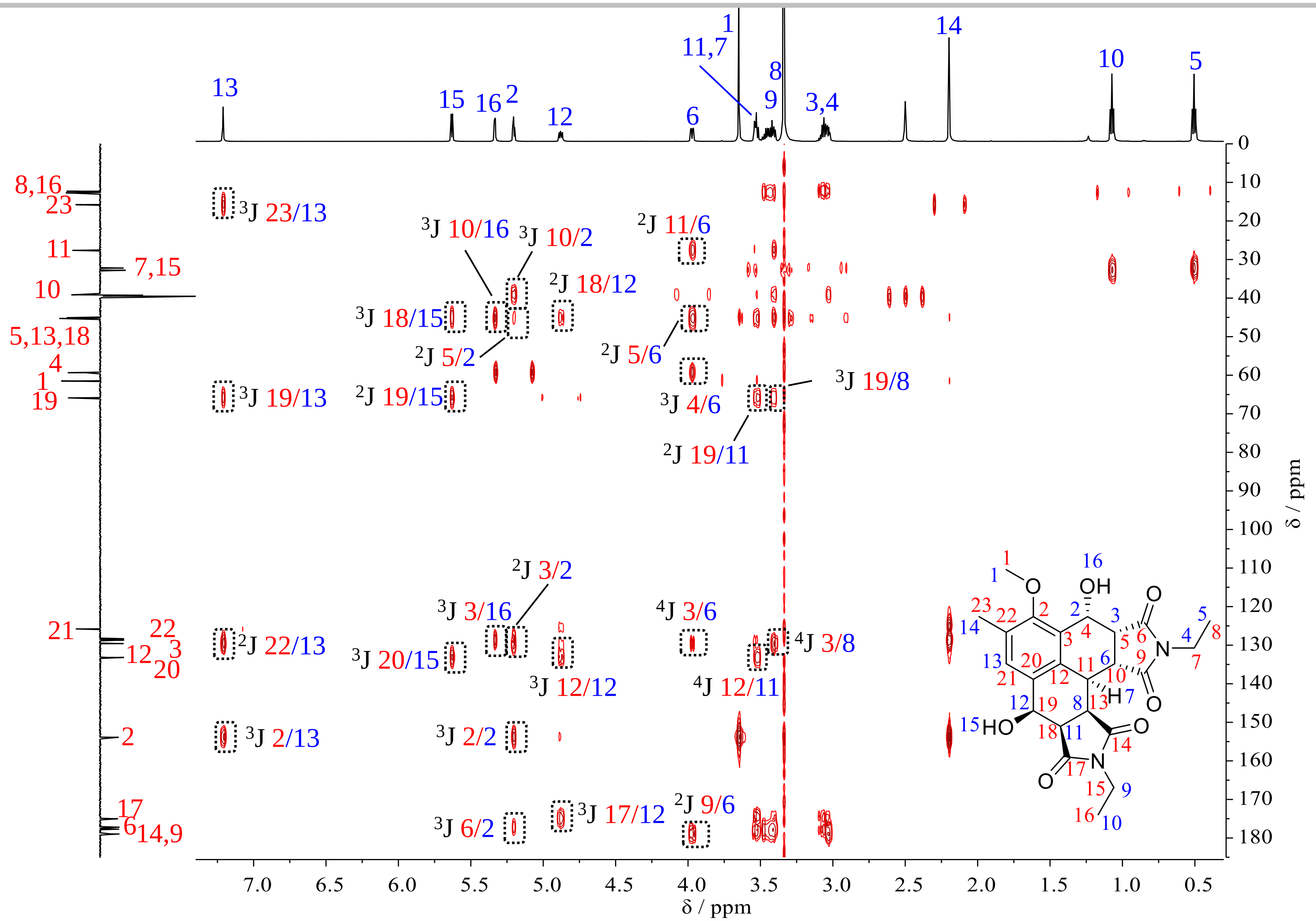

Figure S40 $^1H/^{13}C$-HMBC spectrum of (3a*R*,4*R*,8*R*,8a*R*,11a*R*,11b*R*,11c*R*)-2,10-diethyl-4,8-dihydroxy-5-methoxy-6-methyl-3a,8,8a,11a,11b,11c-hexahydro-1*H*-pyrrolo[3',4':2,3]naphtho[1,8-*ef*]isoindole-1,3,9,11(2*H*,4*H*,10*H*)-tetraone (**4a**) recorded in DMSO-d_6 and assigned ^2J, ^3J and ^4J couplings.

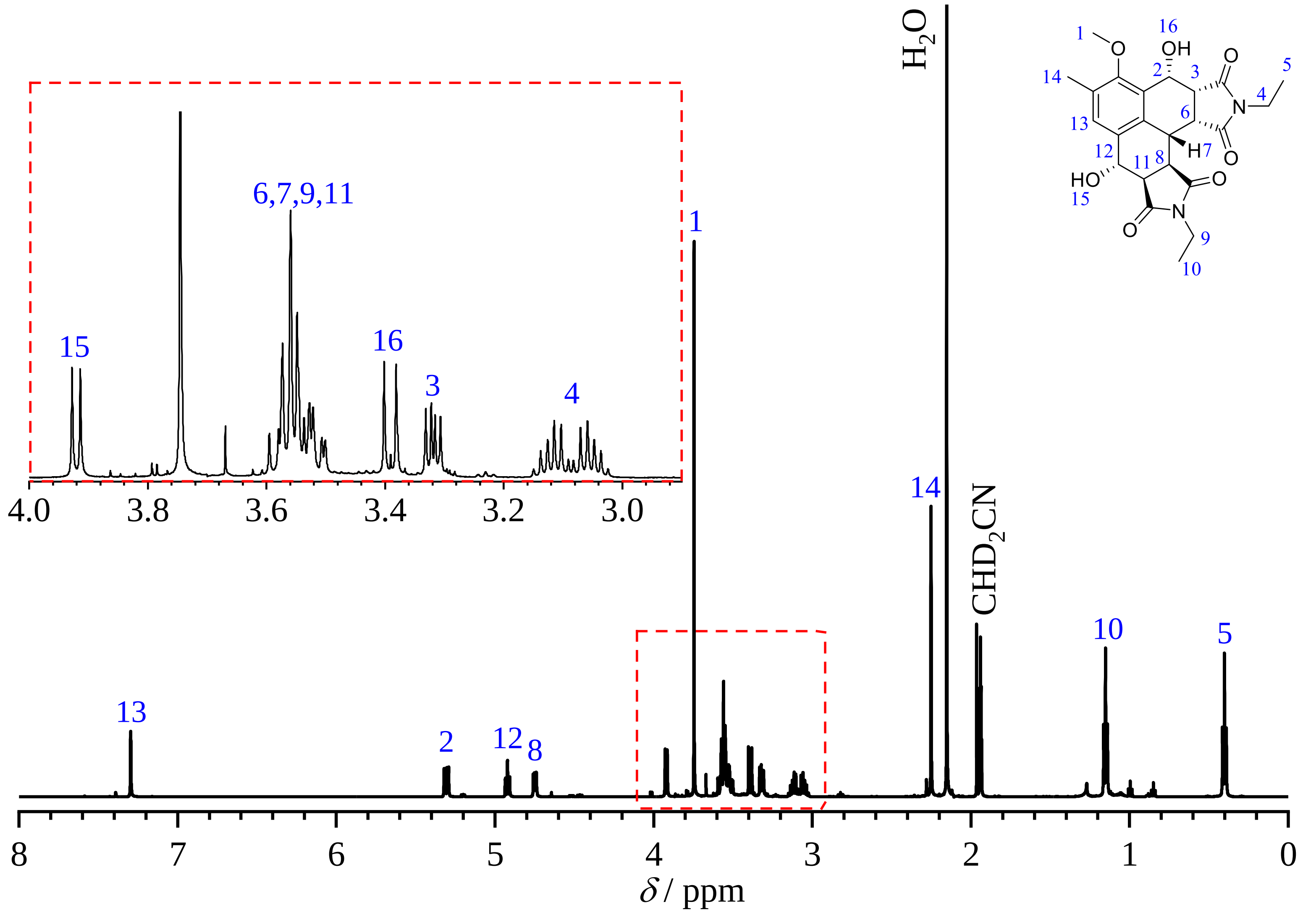

Figure S41 ^{1}H NMR spectrum of (3a*R*,4*R*,8S,8a*R*,11a*R*,11b*S*,11c*R*)-2,10-diethyl-4,8-dihydroxy-5-methoxy-6-methyl-3a,8,8a,11a,11b,11c-hexahydro-1*H*-pyrrolo[3',4':2,3]naphtho[1,8-*ef*]isoindole-1,3,9,11(2*H*,4*H*,10*H*)-tetraone (***exo*-4a**) in CD_3CN and assigned resonances.

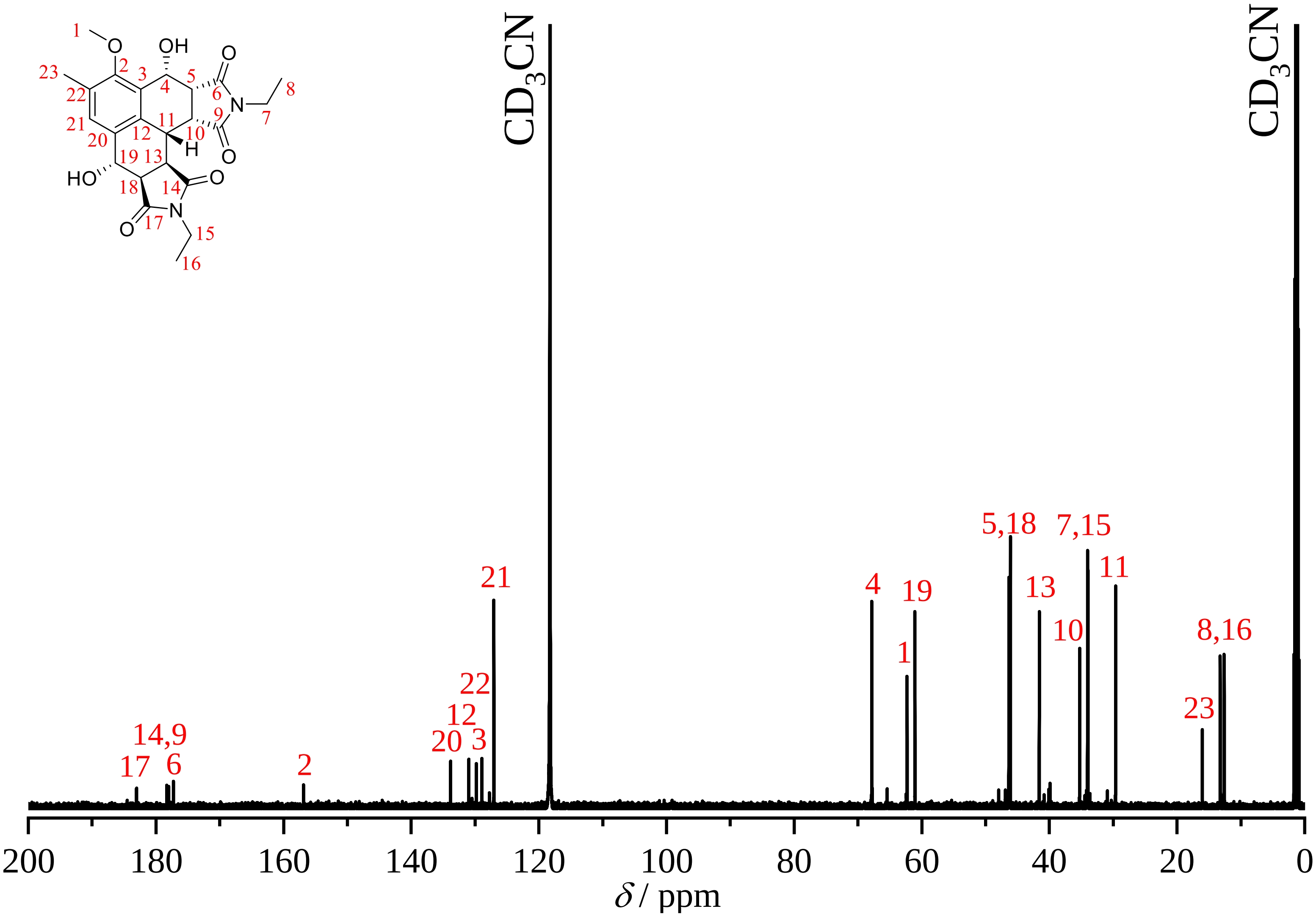

Figure S42 ^{13}C NMR spectrum of (3a*R*,4*R*,8S,8a*R*,11a*R*,11b*S*11c*R*)-2,10-diethyl-4,8-dihydroxy-5-methoxy-6-methyl-3a,8,8a,11a,11b,11c-hexahydro-1*H*-pyrrolo[3',4':2,3]naphtho[1,8-*ef*]isoindole-1,3,9,11(2*H*,4*H*,10*H*)-tetraone (***exo*-4a**) tetraone recorded in CD_3CN and assigned resonances.

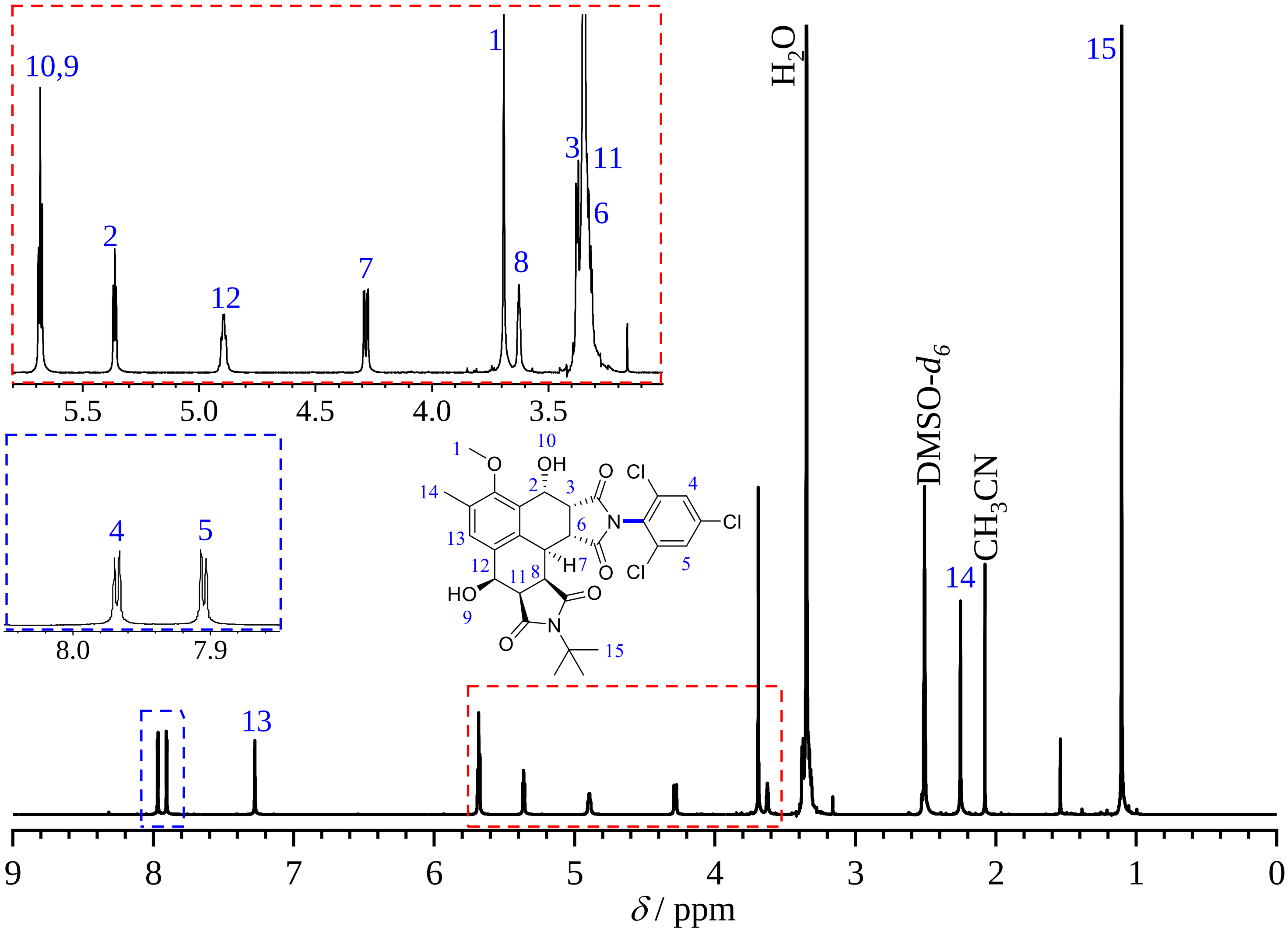

Figure S43 ^{1}H NMR spectrum and assigned resonances of (3a*R*,4*R*,8*R*,8a*R*,11a*R*,11b*R*,11c*R*)-10-(tert-butyl)-4,8-dihydroxy-5-methoxy-6-methyl-2-(2,4,6-trichlorophenyl)-3a,8,8a,11a,11b,11c-hexahydro-1*H*-pyrrolo[3',4':2,3]naphtho[1,8-*ef*]isoindole-1,3,9,11(2*H*,4*H*,10*H*)-tetraone recorded in CD_3CN (***endo*-4b**), recorded in DMSO-d_6.

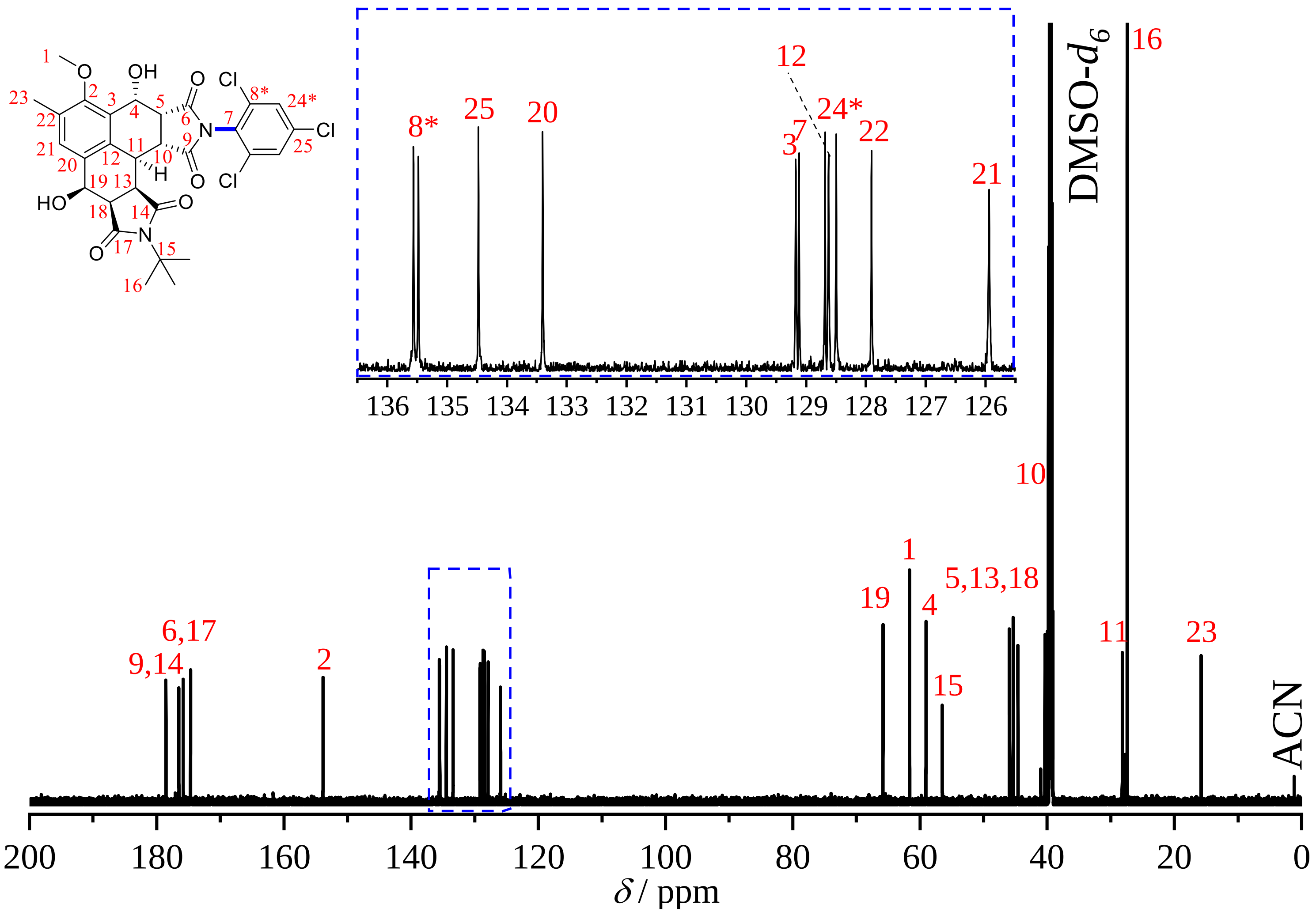

Figure S44 ^{13}H NMR spectrum and assigned resonances of ^{1}H NMR spectrum and assigned resonances of (3a*R*,4*R*,8*R*,8a*R*,11a*R*,11b*R*,11c*R*)-10-(tert-butyl)-4,8-dihydroxy-5-methoxy-6-methyl-2-(2,4,6-trichlorophenyl)-3a,8,8a,11a,11b,11c-hexahydro-1*H*-pyrrolo[3',4':2,3]naphtho[1,8-*ef*]isoindole-1,3,9,11(2*H*,4*H*,10*H*)-tetraone recorded in CD_3CN (***endo*-4b**), recorded in DMSO-d_6.

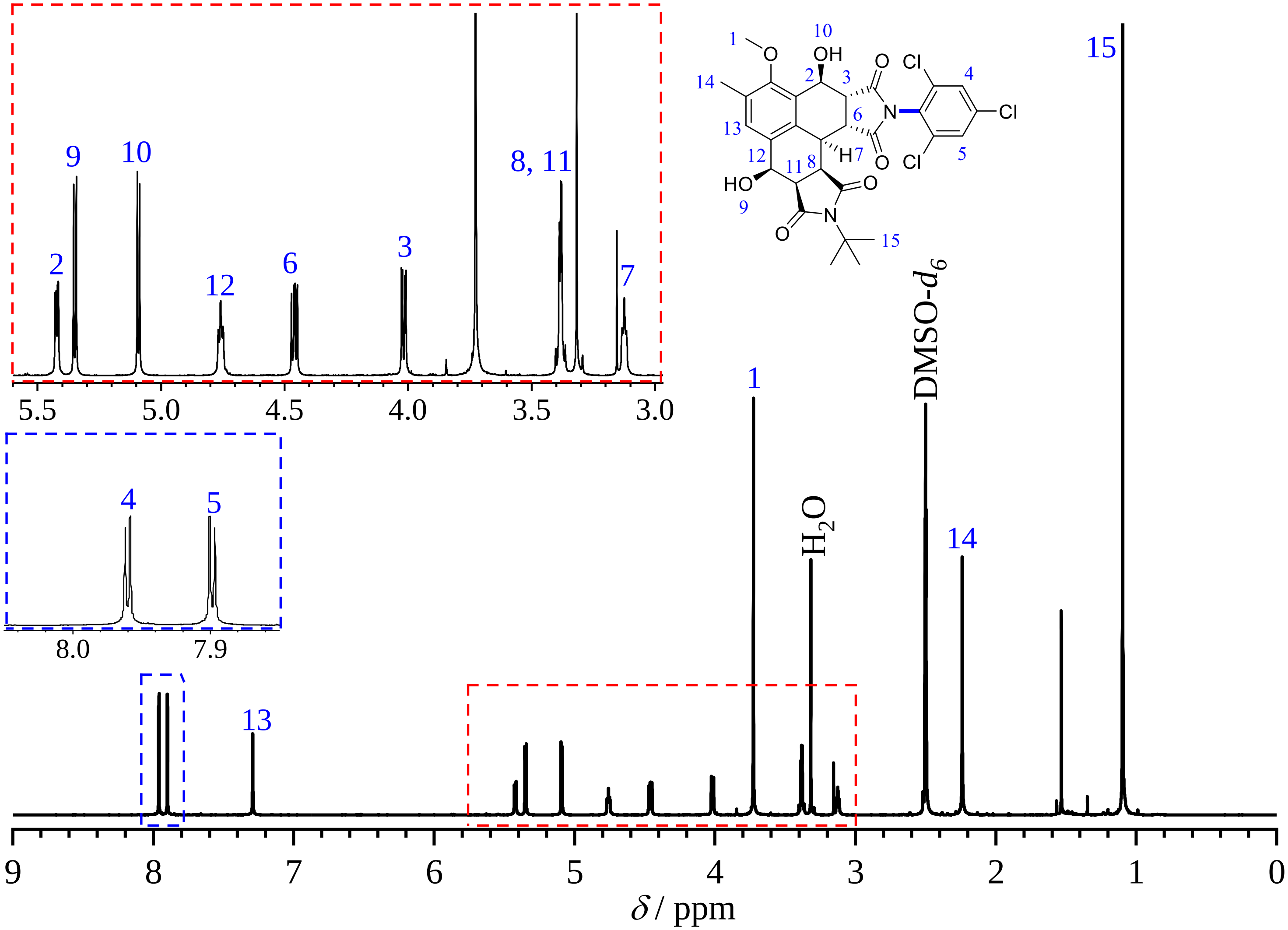

Figure S45 ^{1}H NMR spectrum and assigned resonances of ^{1}H NMR spectrum and assigned resonances of (3a*R*,4*R*,8*S*,8a*R*,11a*R*,11b*R*,11c*R*)-10-(tert-butyl)-4,8-dihydroxy-5-methoxy-6-methyl-2-(2,4,6-trichlorophenyl)-3a,8,8a,11a,11b,11c-hexahydro-1*H*-pyrrolo[3',4':2,3]naphtho[1,8-*ef*]isoindole-1,3,9,11(2*H*,4*H*,10*H*)-tetraone recorded in CD_3CN (***exo*-4b**), recorded in DMSO-d_6.

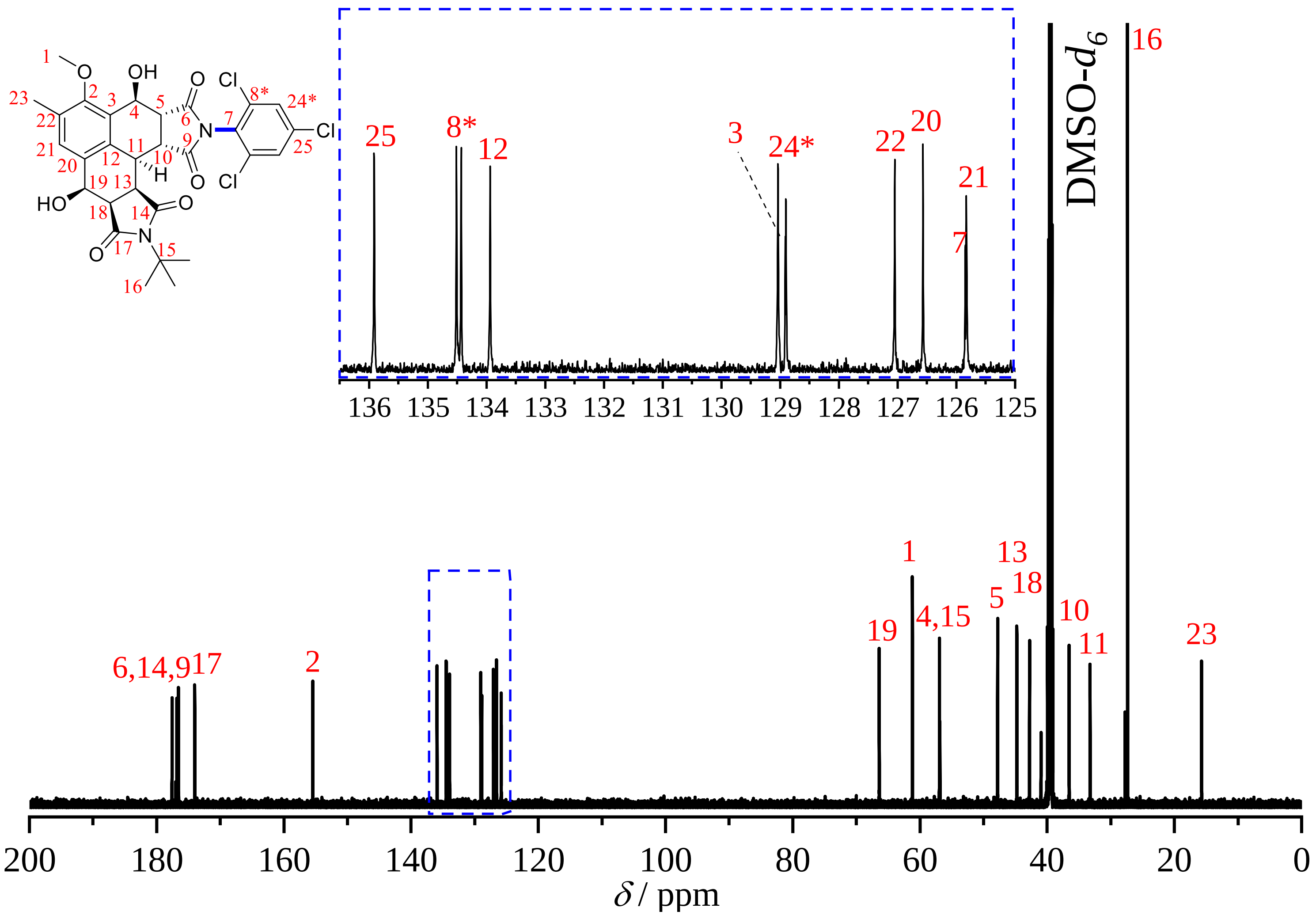

Figure S46 ^{13}C NMR spectrum and assigned resonances of ^{1}H NMR spectrum and assigned resonances of (3a*R*,4*R*,8*S*,8a*R*,11a*R*,11b*R*,11c*R*)-10-(tert-butyl)-4,8-dihydroxy-5-methoxy-6-methyl-2-(2,4,6-trichlorophenyl)-3a,8,8a,11a,11b,11c-hexahydro-1*H*-pyrrolo[3',4':2,3]naphtho[1,8-*ef*]isoindole-1,3,9,11(2*H*,4*H*,10*H*)-tetraone recorded in CD_3CN (***exo*-4b**), recorded in DMSO-d_6.

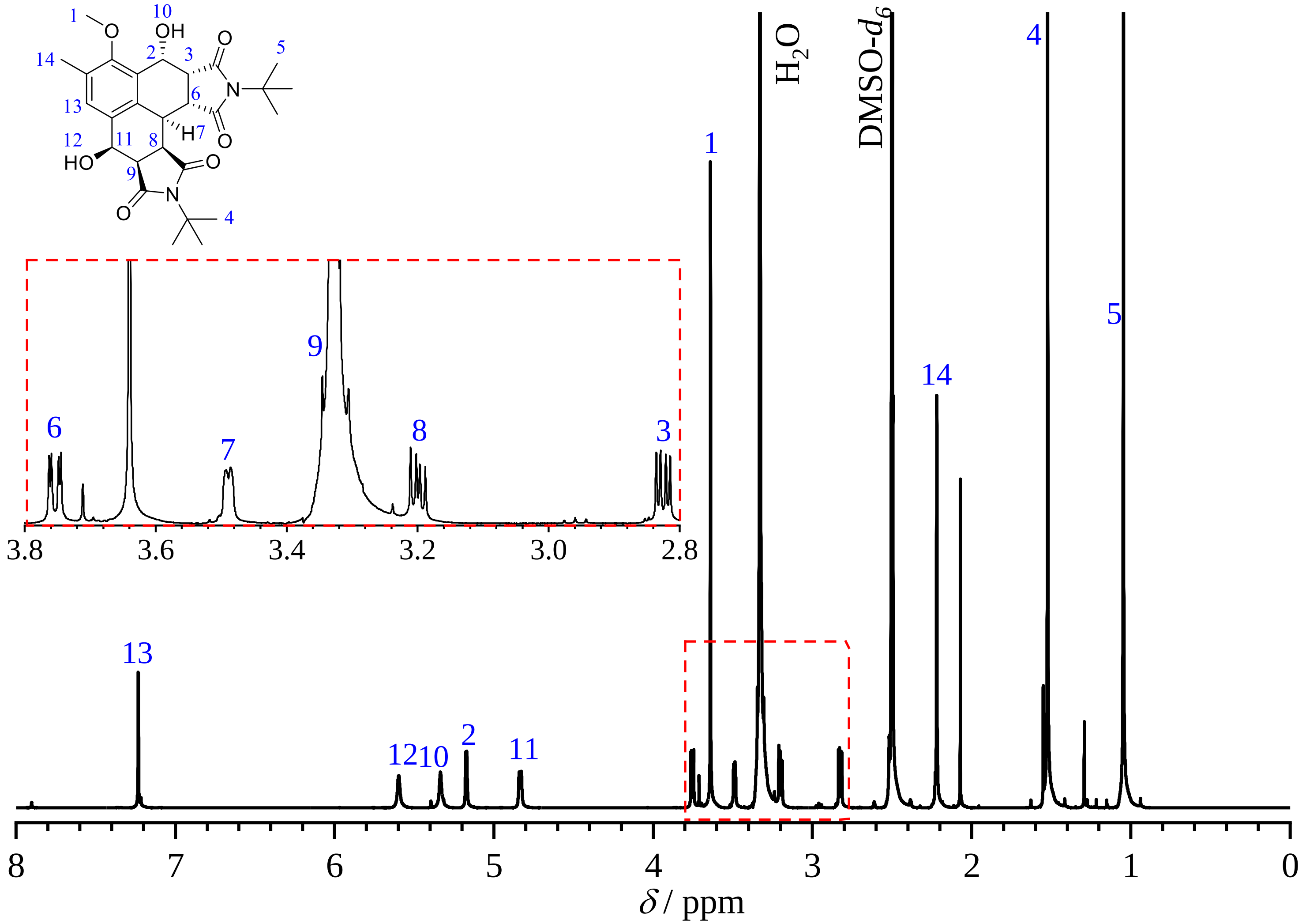

Figure S47 ^{1}H NMR spectrum and assigned resonances of (3a*R*,4*R*,8*R*,8a*R*,11a*R*,11b*R*,11c*R*)-2,10-di-tert-butyl-4,8-dihydroxy-5-methoxy-6-methyl-3a,8,8a,11a,11b,11c-hexahydro-1*H*-pyrrolo[3',4':2,3]naphtho[1,8-*ef*]isoindole-1,3,9,11(2*H*,4*H*,10*H*)-tetraone (**4c**) recorded in DMSO-d_6.

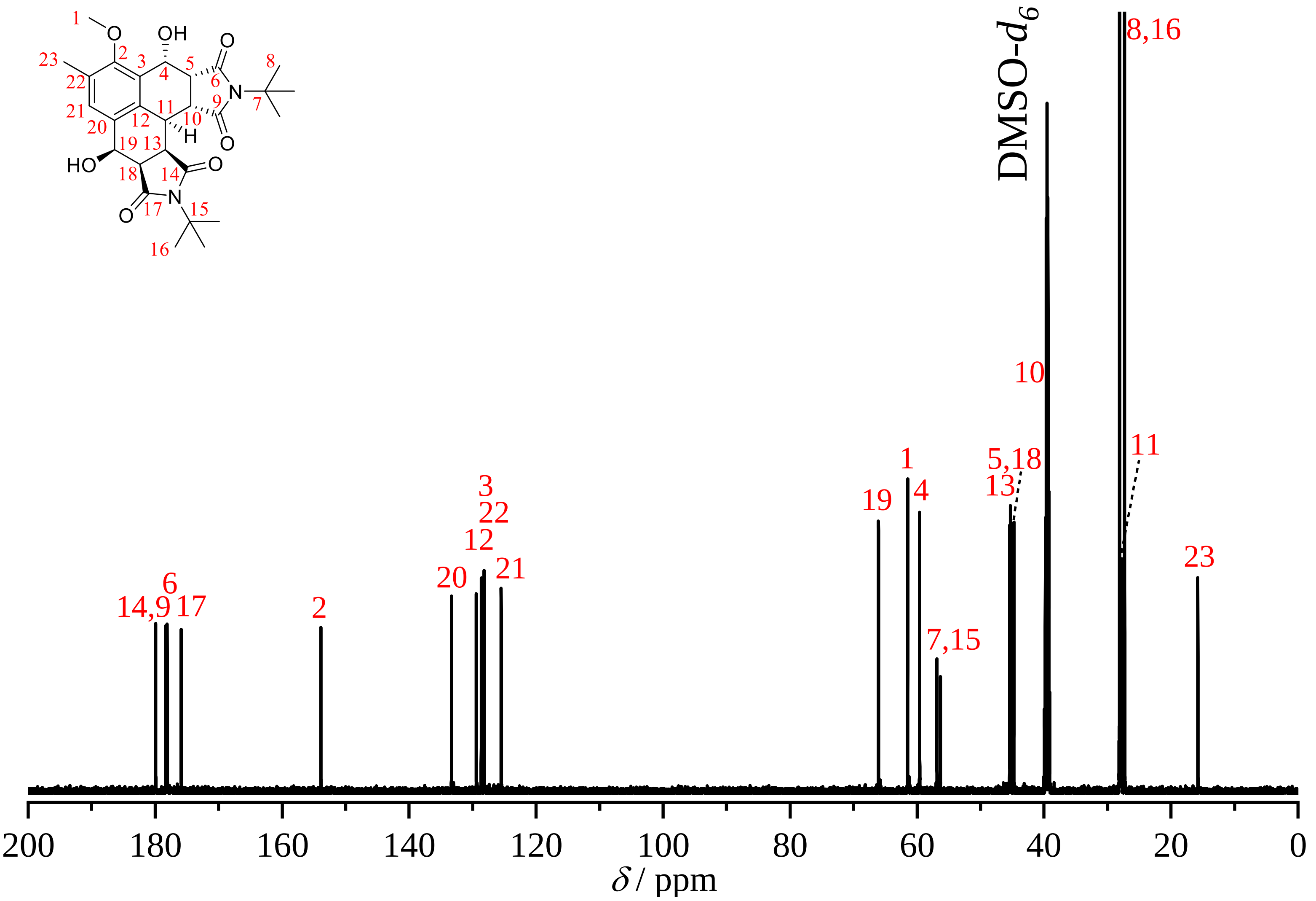

Figure S48 ^{13}C NMR spectrum and assigned resonances of (3a*R*,4*R*,8*R*,8a*R*,11a*R*,11b*R*,11c*R*)-2,10-di-tert-butyl-4,8-dihydroxy-5-methoxy-6-methyl-3a,8,8a,11a,11b,11c-hexahydro-1*H*-pyrrolo[3',4':2,3]naphtho[1,8-*ef*]isoindole-1,3,9,11(2*H*,4*H*,10*H*)-tetraone (**4c**) recorded in DMSO-d_6.

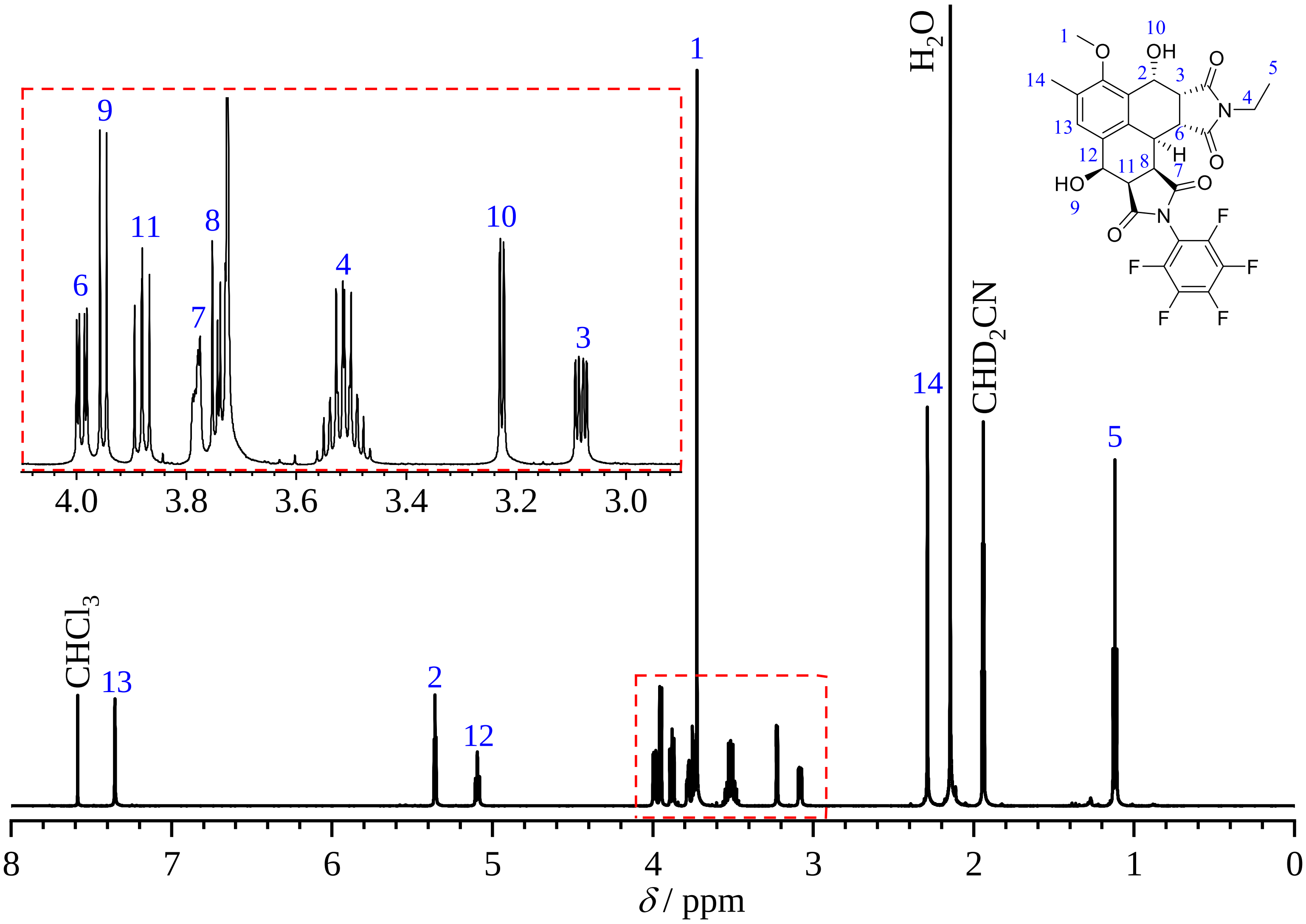

Figure S49 ^{1}H NMR spectrum and assigned resonances of (3a*R*,4*R*,8*R*,8a*R*,11a*R*,11b*S*,11c*R*)-2-ethyl-4,8-dihydroxy-5-methoxy-6-methyl-10-(perfluorophenyl)-3a,8,8a,11a,11b,11c-hexahydro-1*H*-pyrrolo[3',4':2,3]naphtho[1,8-*ef*]isoindole-1,3,9,11(2*H*,4*H*,10*H*)-tetraone (**4d**) recorded in CD_3CN.

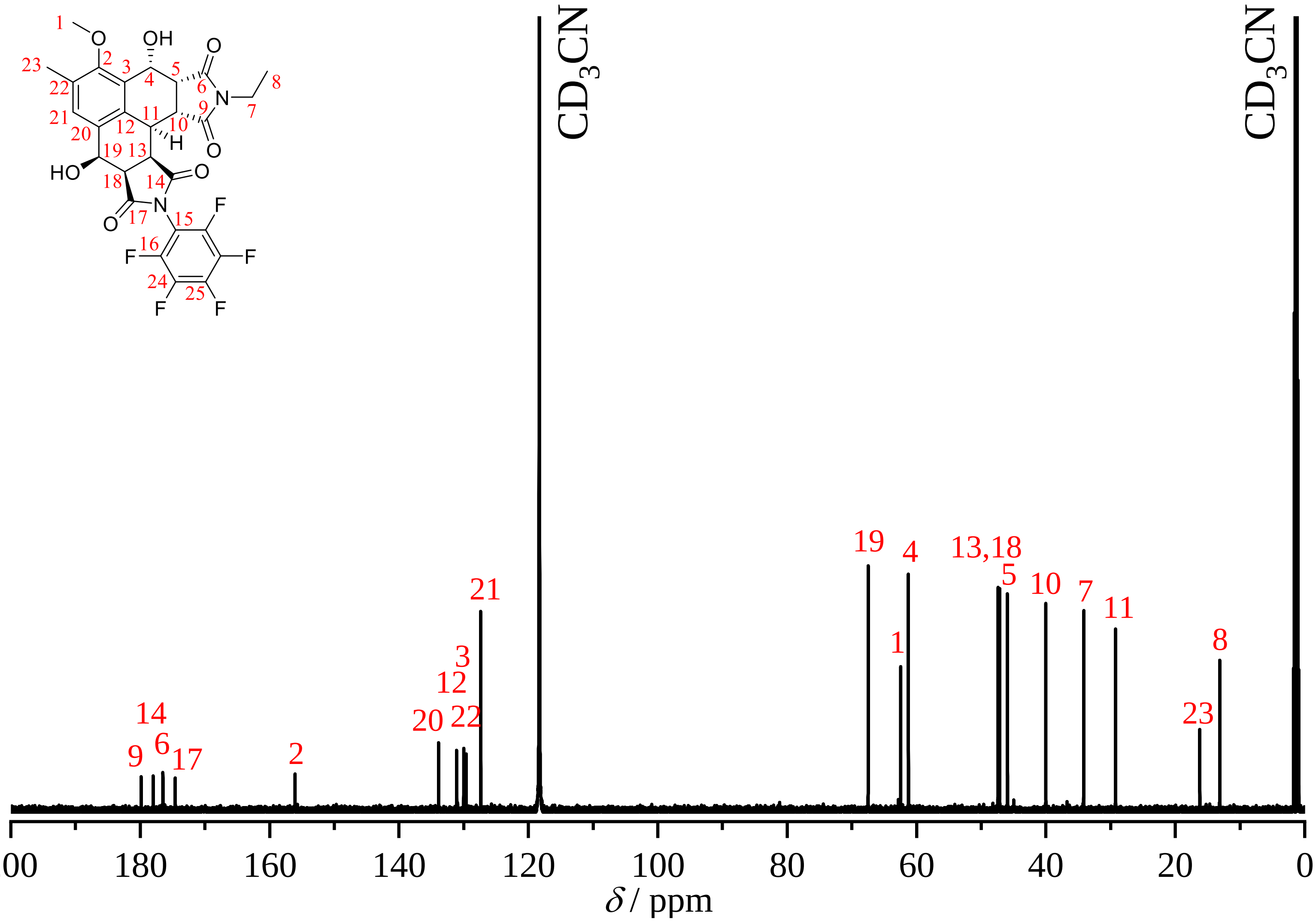

Figure S50 ^{13}C NMR spectrum of (3a*R*,4*R*,8*R*,8a*R*,11a*R*,11b*S*,11c*R*)-2-ethyl-4,8-dihydroxy-5-methoxy-6-methyl-10-(perfluorophenyl)-3a,8,8a,11a,11b,11c-hexahydro-1*H*-pyrrolo[3',4':2,3]naphtho[1,8-*ef*]isoindole-1,3,9,11(2*H*,4*H*,10*H*)-tetraone (**4d**) recorded in CD_3CN and assigned resonances. Signals of 15,16,24,25 are missing due to low signal intensity caused by ^{19}F-coupling.

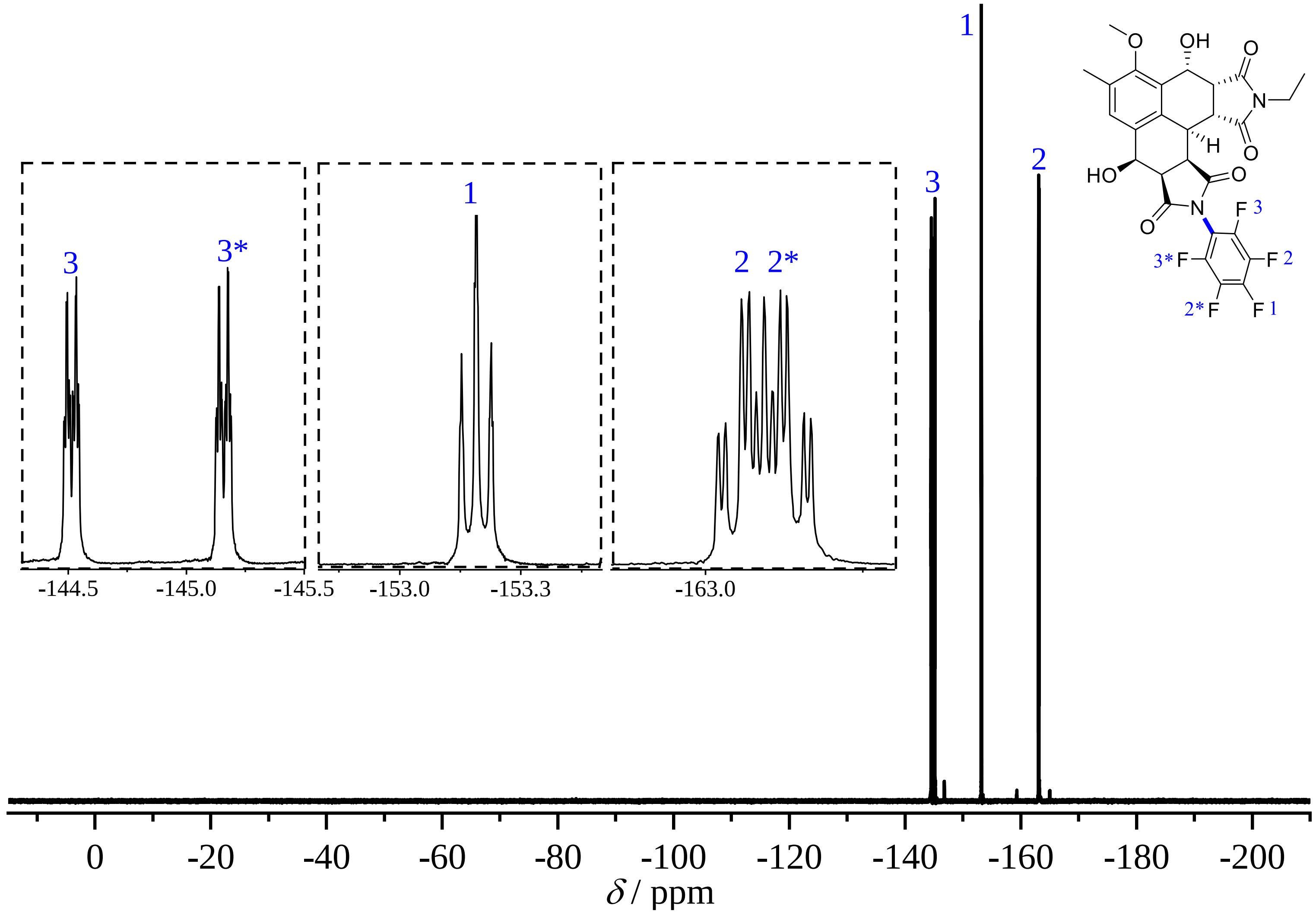

Figure S51 ^{19}F NMR spectrum of (3a*R*,4*R*,8*R*,8a*R*,11a*R*,11b*S*,11c*R*)-2-ethyl-4,8-dihydroxy-5-methoxy-6-methyl-10-(perfluorophenyl)-3a,8,8a,11a,11b,11c-hexahydro-1*H*-pyrrolo[3',4':2,3]naphtho[1,8-*ef*]isoindole-1,3,9,11(2*H*,4*H*,10*H*)-tetraone (**4d**) recorded in CD_3CN and assigned resonances.

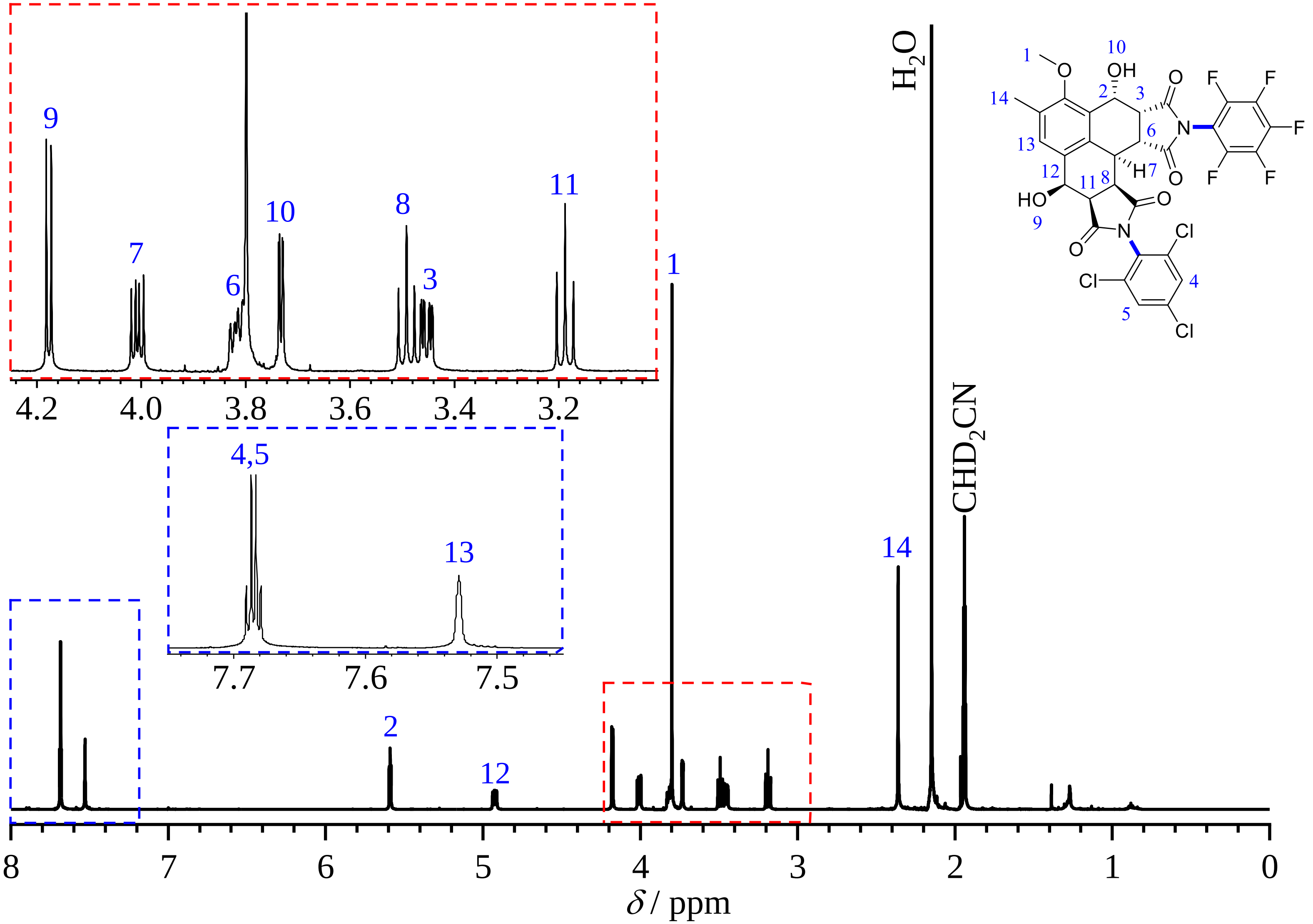

Figure S52 ^{1}H NMR spectrum and assigned resonances of (3a*R*,4*R*,8*R*,8a*R*,11a*R*,11b*S*,11c*R*)-4,8-dihydroxy-5-methoxy-6-methyl-2-(perfluorophenyl)-10-(2,4,6-trichlorophenyl)-3a,8,8a,11a,11b,11c-hexahydro-1H-pyrrolo[3',4':2,3]naphtho[1,8-*ef*]isoindole-1,3,9,11(2*H*,4*H*,10*H*)-tetraone (***endo*-4e**) recorded in CD_3CN.

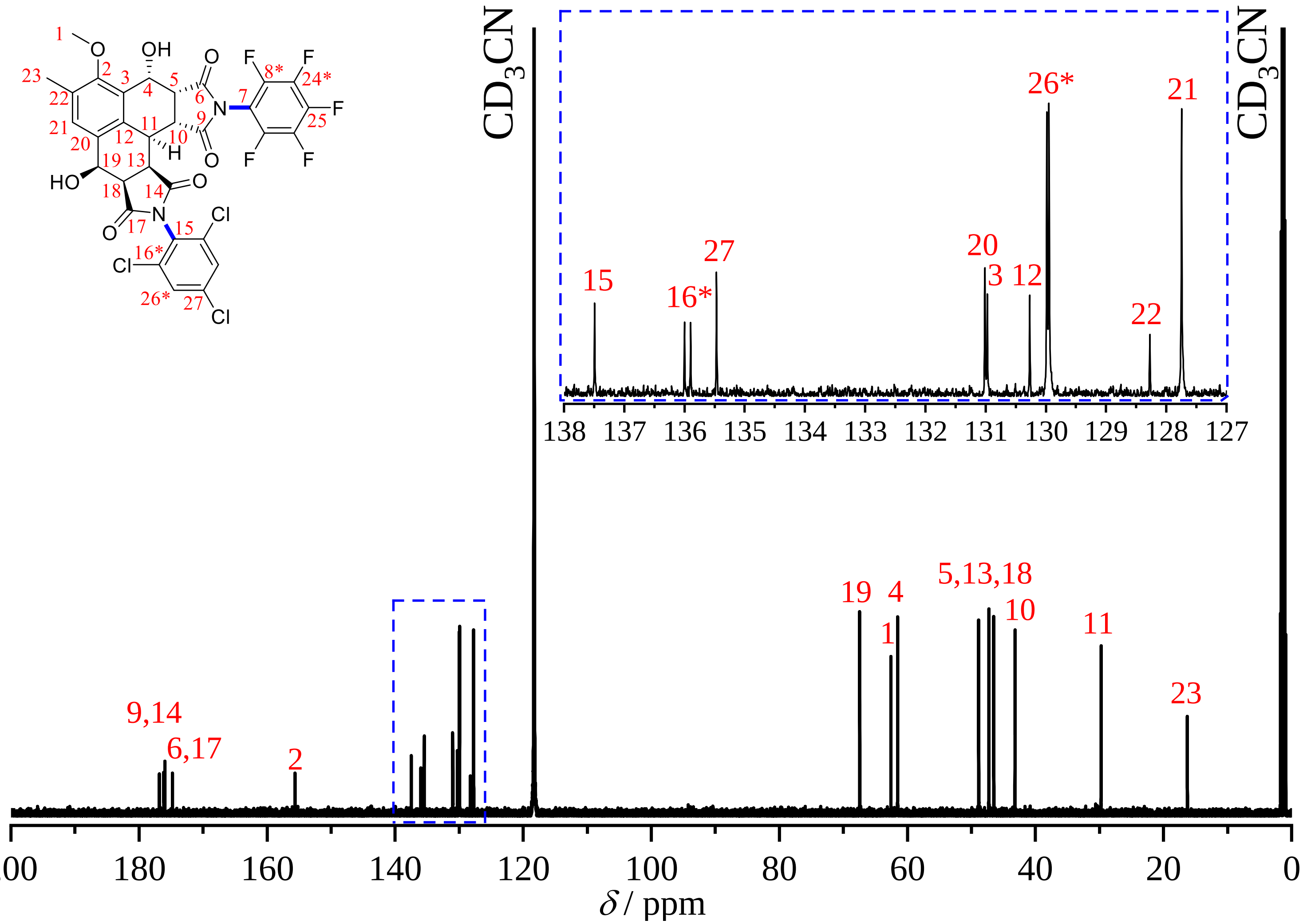

Figure S53 ^{13}C NMR spectrum and assigned resonances of (3a*R*,4*R*,8*R*,8a*R*,11a*R*,11b*S*,11c*R*)-4,8-dihydroxy-5-methoxy-6-methyl-2-(perfluorophenyl)-10-(2,4,6-trichlorophenyl)-3a,8,8a,11a,11b,11c-hexahydro-1H-pyrrolo[3',4':2,3]naphtho[1,8-*ef*]isoindole-1,3,9,11(2*H*,4*H*,10*H*)-tetraone (***endo*-4e**) recorded in CD_3CN. Signals of 7,8,24,25 are missing due to low signal intensity caused by ^{19}F-coupling.

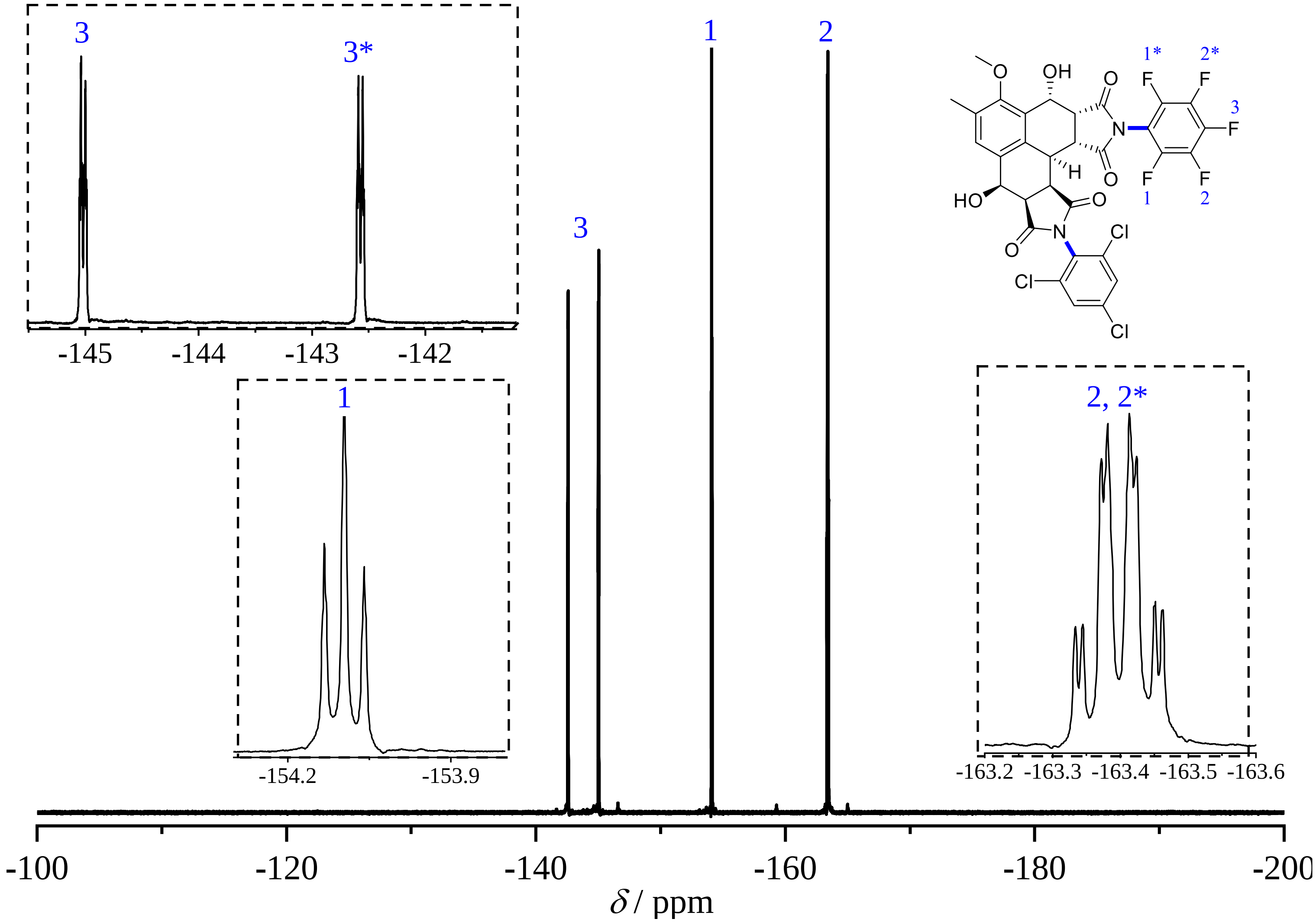

Figure S54 ^{19}F NMR spectrum and assigned resonances of (3a*R*,4*R*,8*R*,8a*R*,11a*R*,11b*S*,11c*R*)-4,8-dihydroxy-5-methoxy-6-methyl-2-(perfluorophenyl)-10-(2,4,6-trichlorophenyl)-3a,8,8a,11a,11b,11c-hexahydro-1H-pyrrolo[3',4':2,3]naphtho[1,8-*ef*]isoindole-1,3,9,11(2*H*,4*H*,10*H*)-tetraone (***endo*-4e**) recorded in CD_3CN.

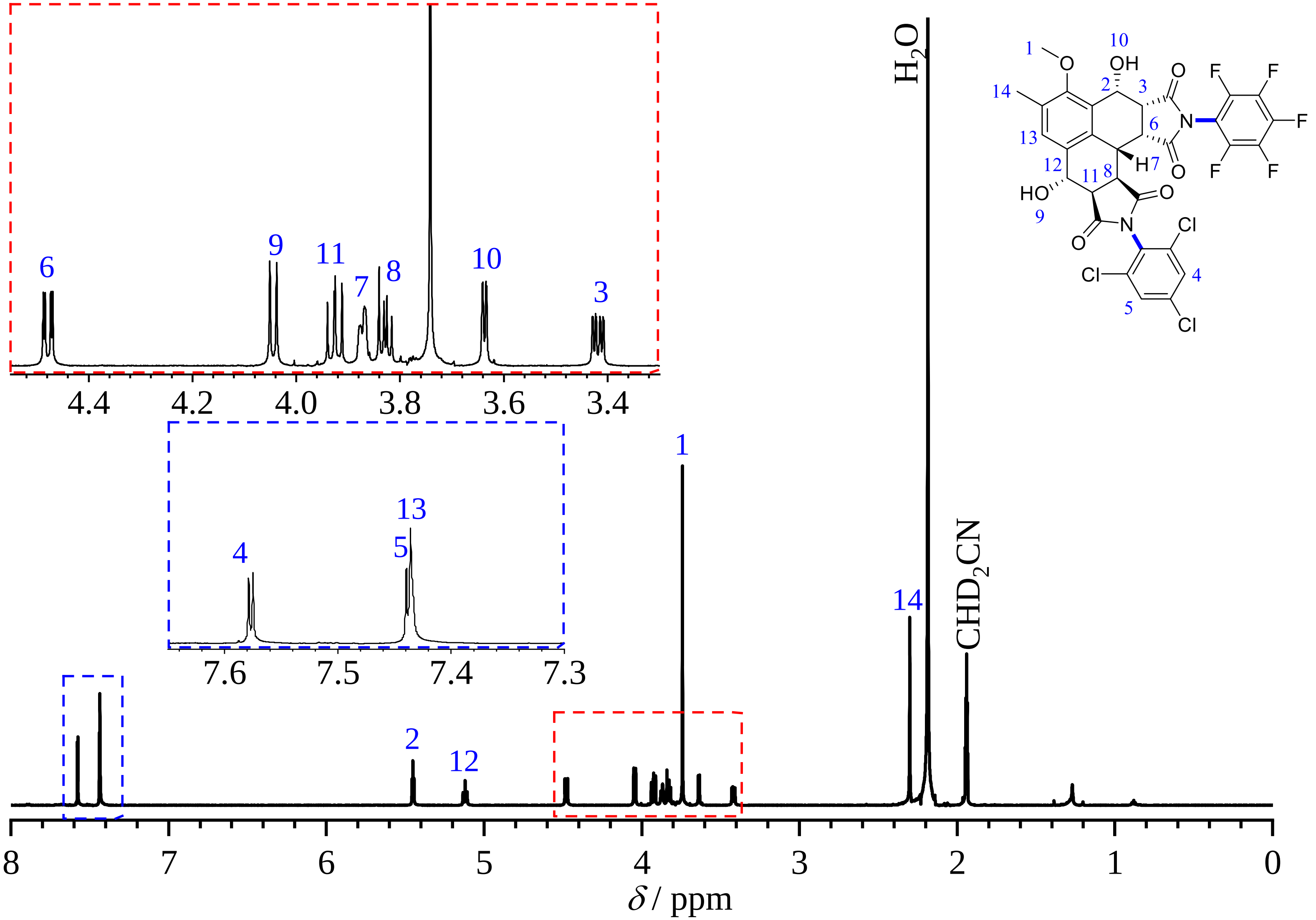

Figure S55 ^{1}H NMR spectrum and assigned resonances of (3a*R*,4*R*,8*R*,8a*R*,11a*R*,11b*R*,11c*R*)-4,8-dihydroxy-5-methoxy-6-methyl-2-(perfluorophenyl)-10-(2,4,6-trichlorophenyl)-3a,8,8a,11a,11b,11c-hexahydro-1H-pyrrolo[3',4':2,3]naphtho[1,8-*ef*]isoindole-1,3,9,11(2*H*,4*H*,10*H*)-tetraone (***exo*-4e**) recorded in CD_3CN.

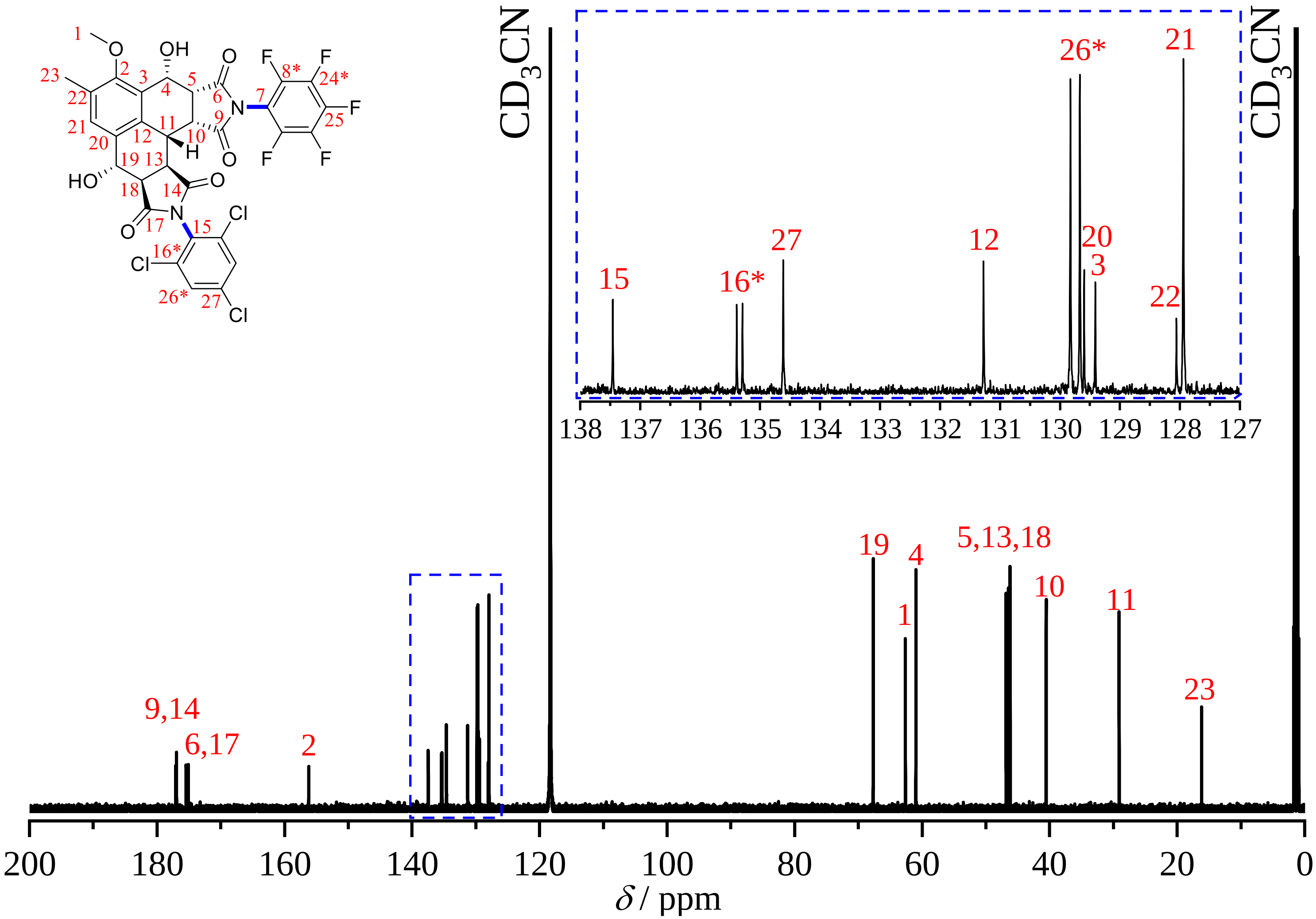

Figure S56 ^{13}C NMR spectrum and assigned resonances of (3a*R*,4*R*,8*R*,8a*R*,11a*R*,11b*R*,11c*R*)-4,8-dihydroxy-5-methoxy-6-methyl-2-(perfluorophenyl)-10-(2,4,6-trichlorophenyl)-3a,8,8a,11a,11b,11c-hexahydro-1H-pyrrolo[3',4':2,3]naphtho[1,8-*ef*]isoindole-1,3,9,11(2*H*,4*H*,10*H*)-tetraone (***exo*-4e**) recorded in CD_3CN. Signals of 7,8,24,25 are missing due to low signal intensity caused by ^{19}F-coupling.

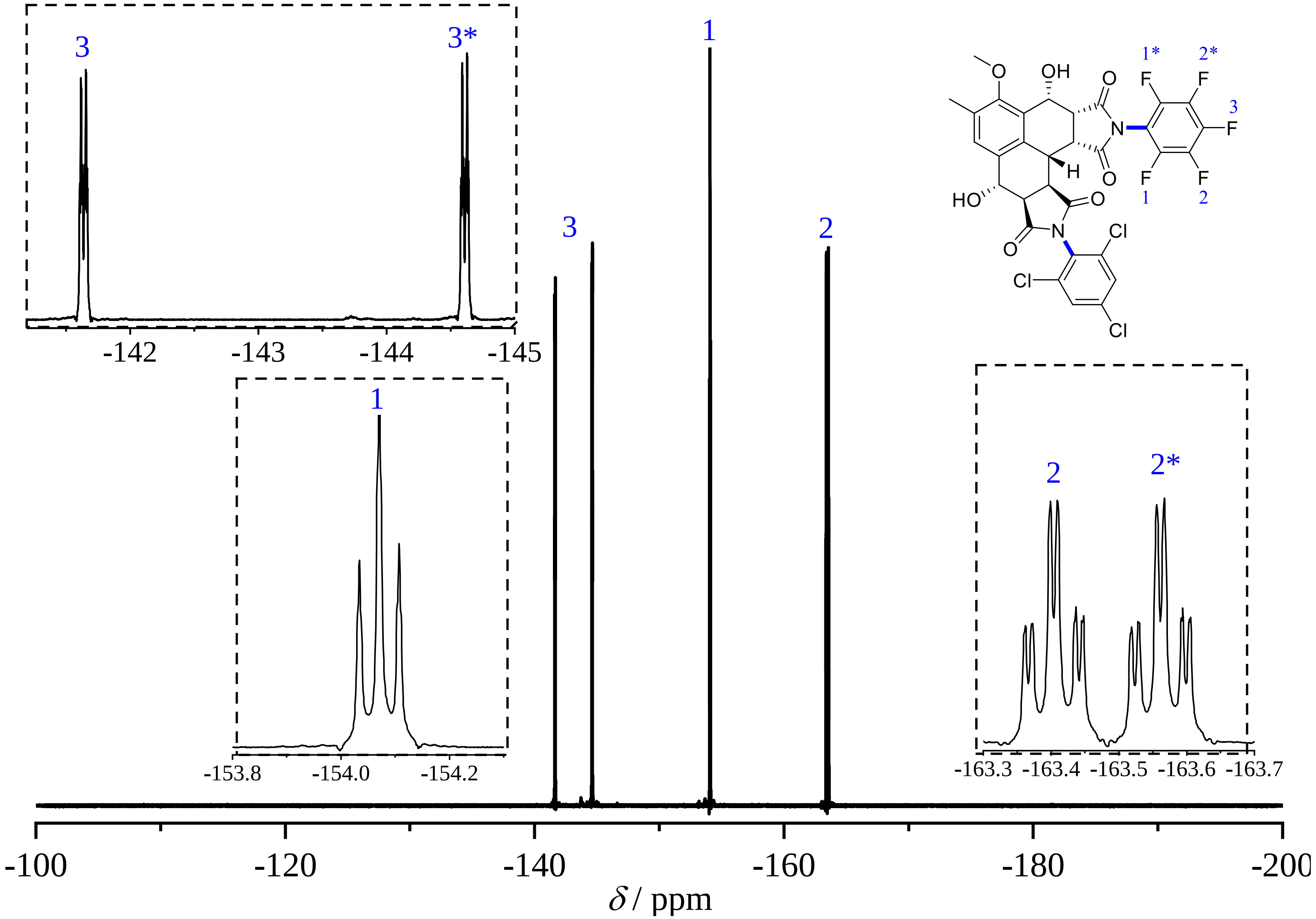

Figure S57 ^{19}F NMR spectrum and assigned resonances of (3a*R*,4*R*,8*R*,8a*R*,11a*R*,11b*R*,11c*R*)-4,8-dihydroxy-5-methoxy-6-methyl-2-(perfluorophenyl)-10-(2,4,6-trichlorophenyl)-3a,8,8a,11a,11b,11c-hexahydro-1*H*-pyrrolo[3',4':2,3]naphtho[1,8-*ef*]isoindole-1,3,9,11(2*H*,4*H*,10*H*)-tetraone (***exo*-4e**) recorded in CD_3CN.

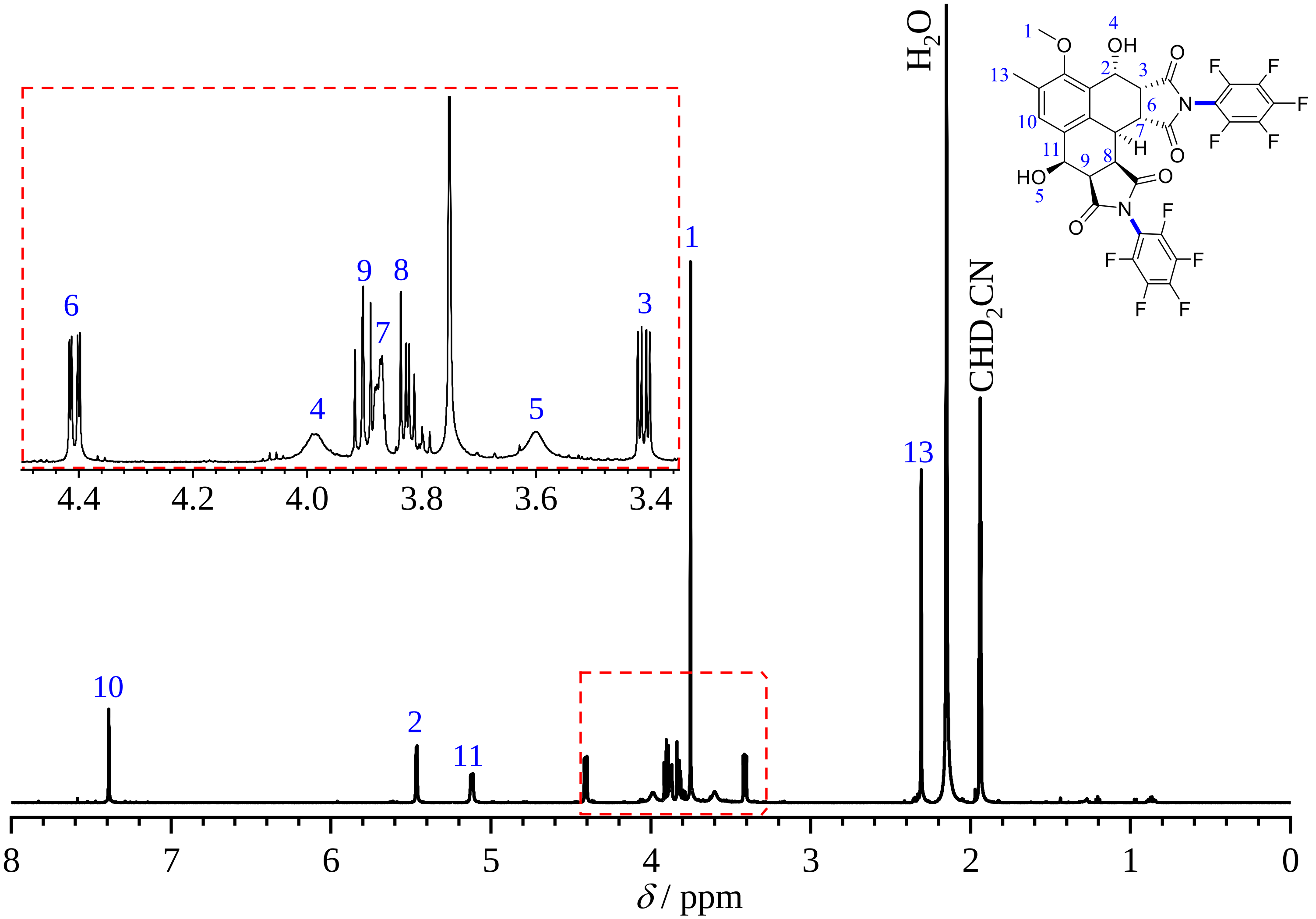

Figure S58 ^{1}H NMR spectrum and assigned resonances of (3a*R*,4*R*,8*R*,8a*R*,11a*R*,11b*R*,11c*R*)-4,8-dihydroxy-5-methoxy-6-methyl-2,10-bis(perfluorophenyl)-3a,8,8a,11a,11b,11c-hexahydro-1H-pyrrolo[3',4':2,3]naphtho[1,8-*ef*]isoindole-1,3,9,11(2*H*,4*H*,10*H*)-tetraone (**4f**) recorded in CD_3CN.

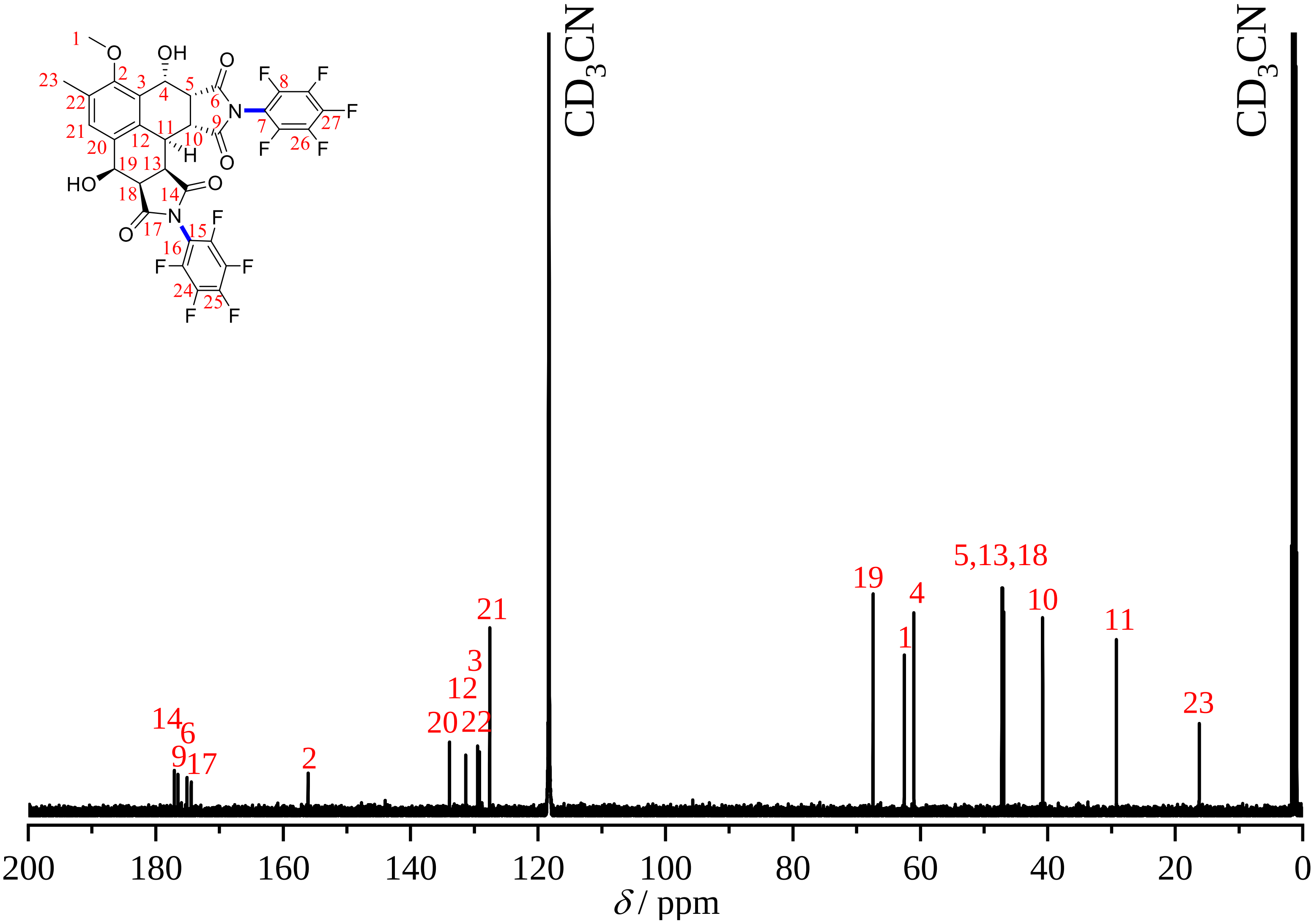

Figure S59 ^{13}C NMR spectrum and assigned resonances of (3a*R*,4*R*,8*R*,8a*R*,11a*R*,11b*R*,11c*R*)-4,8-dihydroxy-5-methoxy-6-methyl-2,10-bis(perfluorophenyl)-3a,8,8a,11a,11b,11c-hexahydro-1H-pyrrolo[3',4':2,3]naphtho[1,8-*ef*]isoindole-1,3,9,11(2*H*,4*H*,10*H*)-tetraone (**4f**) recorded in CD_3CN.

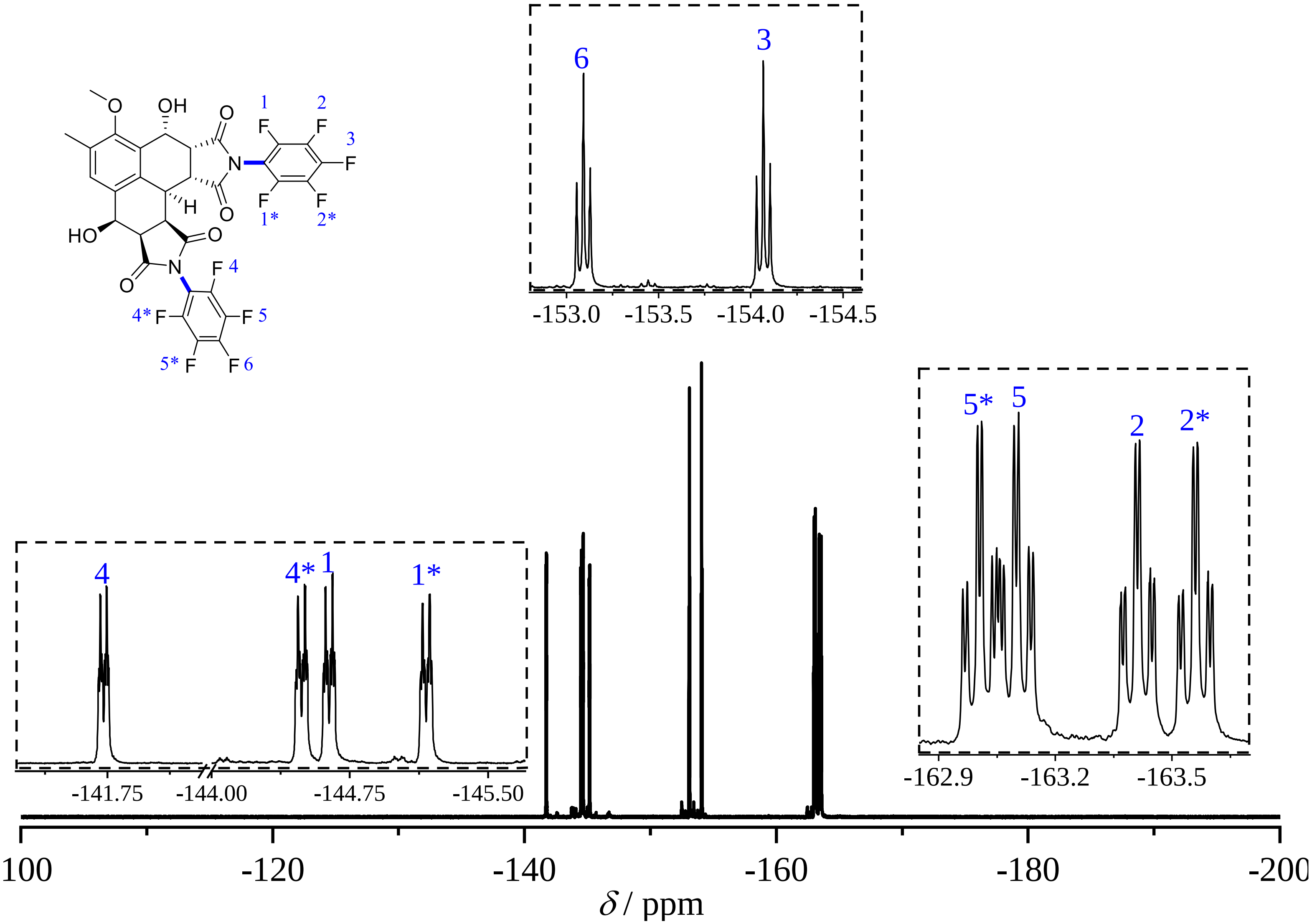

Figure S60 ^{19}F NMR spectrum and assigned resonances of (3a*R*,4*R*,8*R*,8a*R*,11a*R*,11b*R*,11c*R*)-4,8-dihydroxy-5-methoxy-6-methyl-2,10-bis(perfluorophenyl)-3a,8,8a,11a,11b,11c-hexahydro-1*H*-pyrrolo[3',4':2,3]naphtho[1,8-*ef*]isoindole-1,3,9,11(2*H*,4*H*,10*H*)-tetraone (**4f**) recorded in CD_3CN.

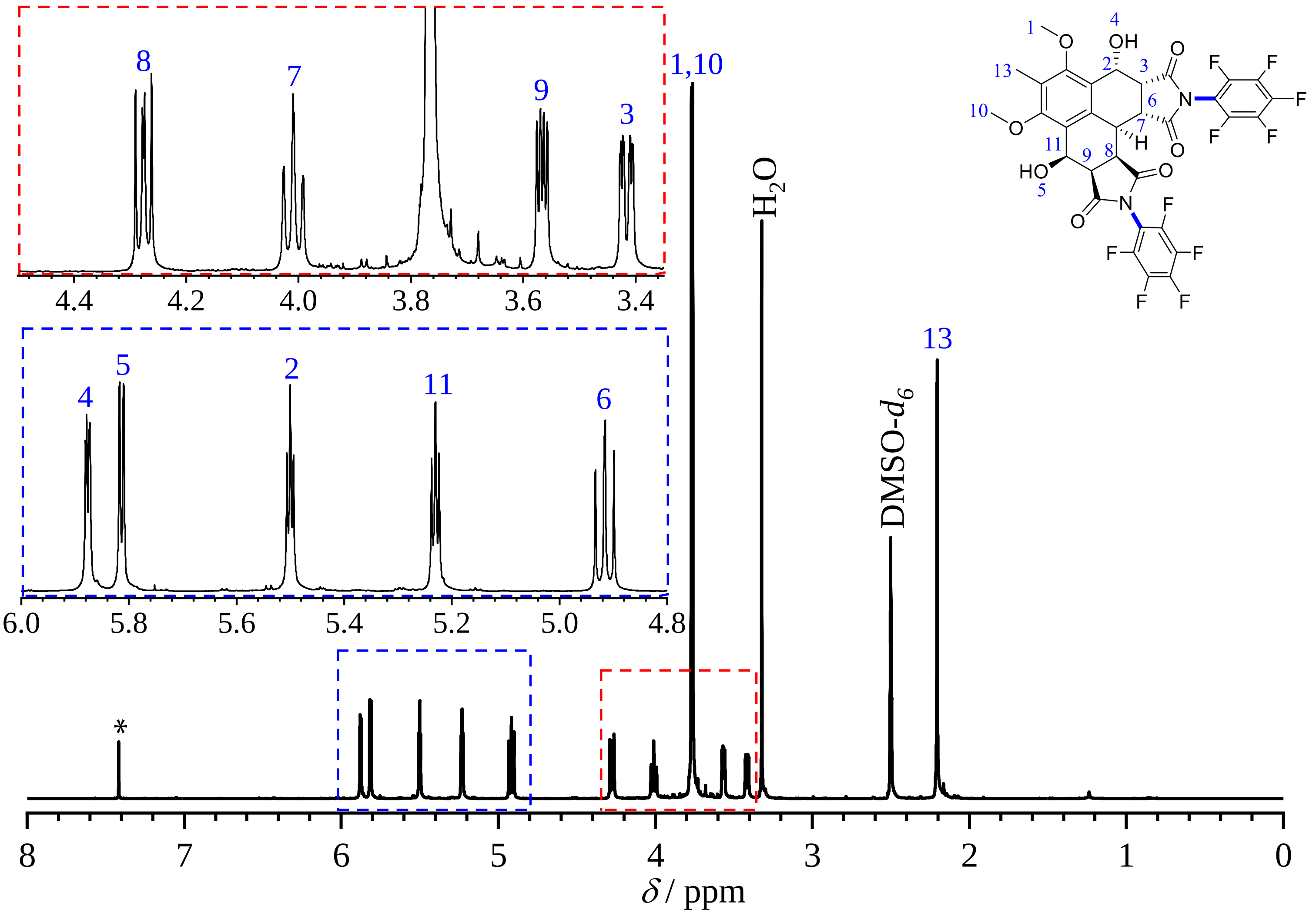

Figure S61 ^{1}H NMR spectrum and assigned resonances of (3a*R*,4*R*,8*R*,8a*R*,11a*R*,11c*R*)-4,8-dihydroxy-5,7-dimethoxy-6-methyl-2,10-bis(perfluorophenyl)-3a,8,8a,11a,11b,11c-hexahydro-1*H*-pyrrolo[3',4':2,3]naphtho[1,8-*ef*]isoindole-1,3,9,11(2*H*,4*H*,10*H*)-tetraone (**4g**) recorded in DMSO-d_6 *: impurity with **2c** (starting material).

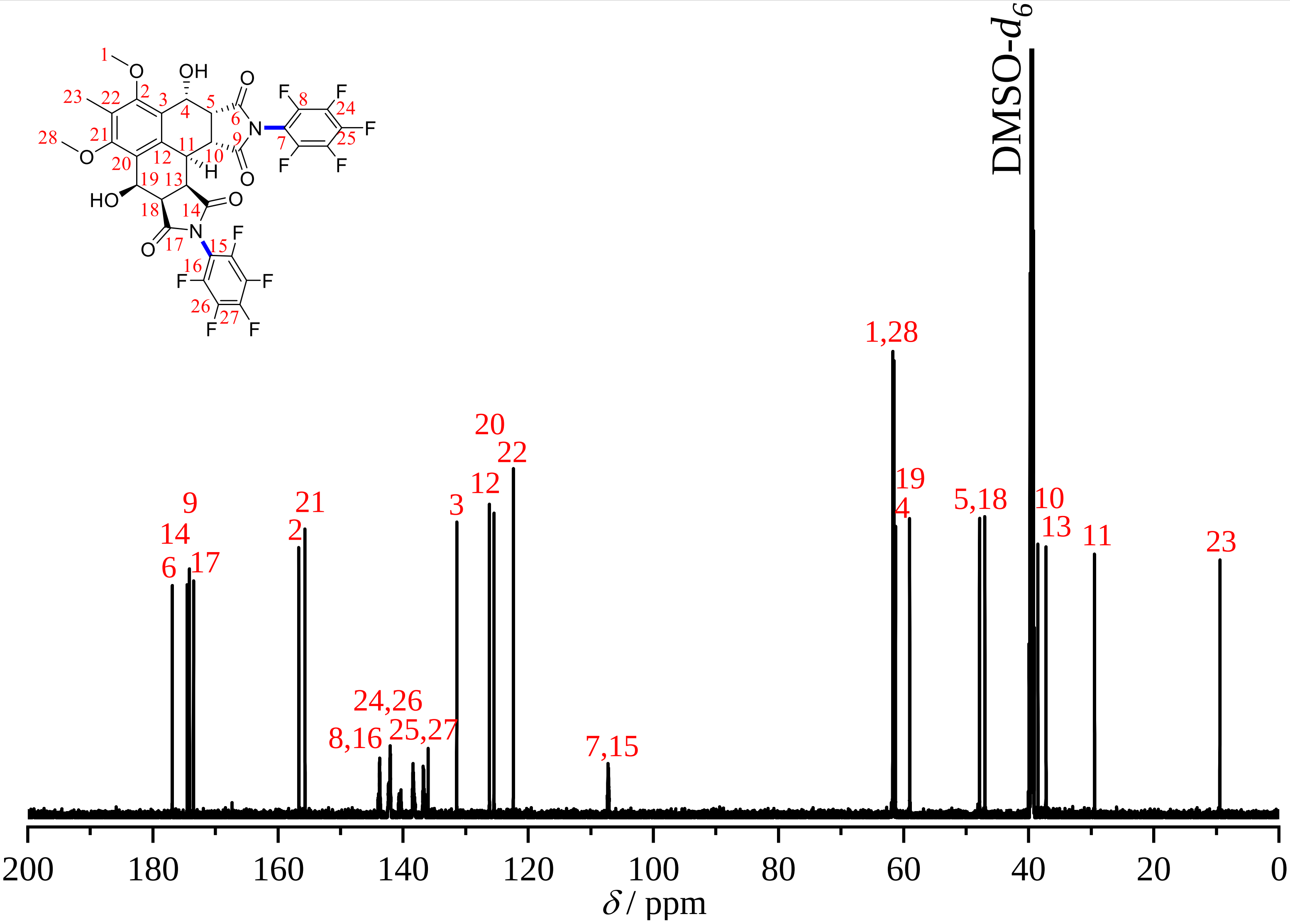

Figure S62 ^{13}C NMR spectrum and assigned resonances of (3a*R*,4*R*,8*R*,8a*R*,11a*R*,11c*R*)-4,8-dihydroxy-5,7-dimethoxy-6-methyl-2,10-bis(perfluorophenyl)-3a,8,8a,11a,11b,11c-hexahydro-1*H*-pyrrolo[3',4':2,3]naphtho[1,8-*ef*]isoindole-1,3,9,11(2*H*,4*H*,10*H*)-tetraone (**4g**) recorded in DMSO-d_6.

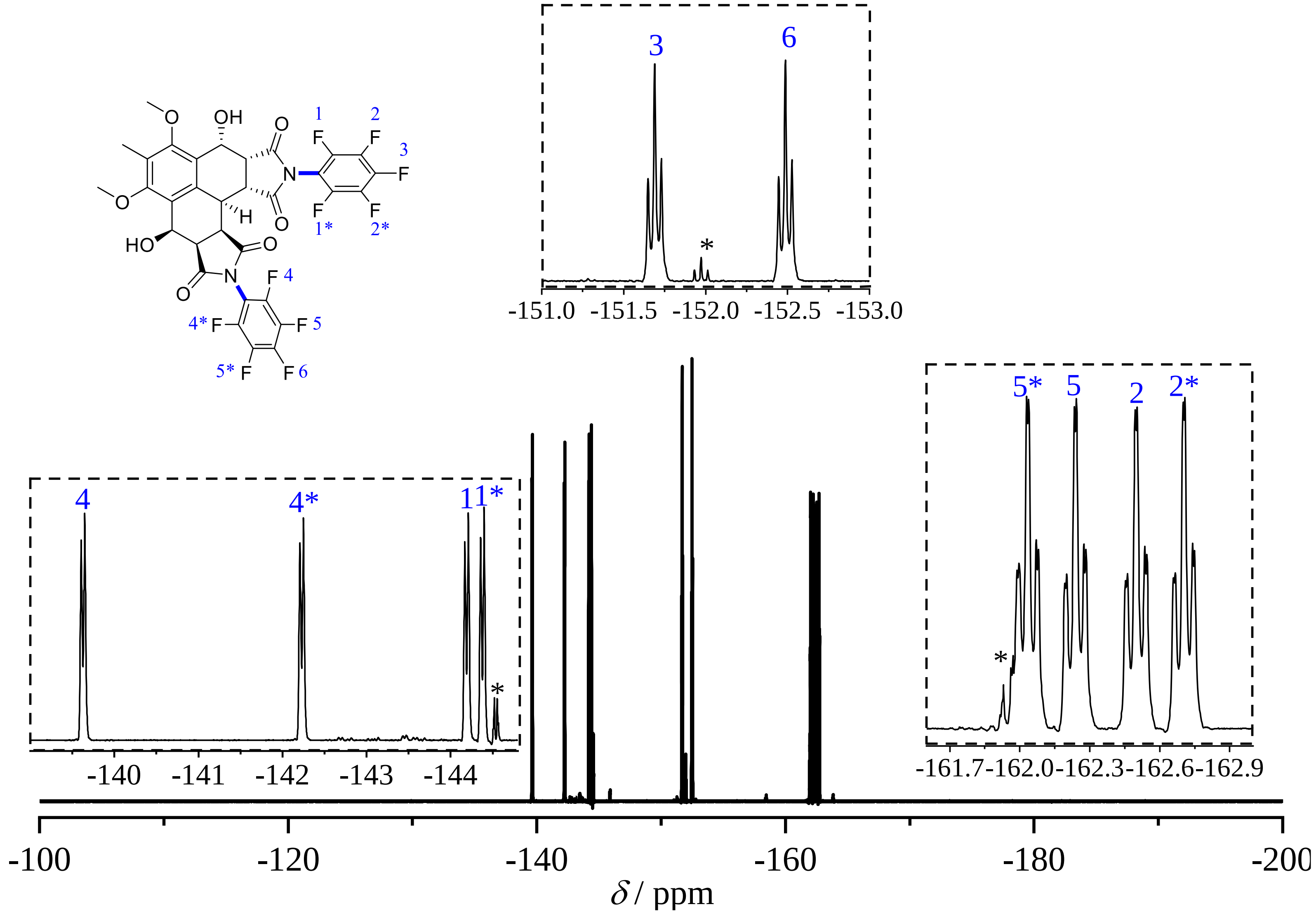

Figure S63 ^{13}C NMR spectrum and assigned resonances of (3a*R*,4*R*,8*R*,8a*R*,11a*R*,11c*R*)-4,8-dihydroxy-5,7-dimethoxy-6-methyl-2,10-bis(perfluorophenyl)-3a,8,8a,11a,11b,11c-hexahydro-1*H*-pyrrolo[3',4':2,3]naphtho[1,8-*ef*]isoindole-1,3,9,11(2*H*,4*H*,10*H*)-tetraone (**4g**) recorded in DMSO-d_6. *: impurity with **2c** (starting materials).

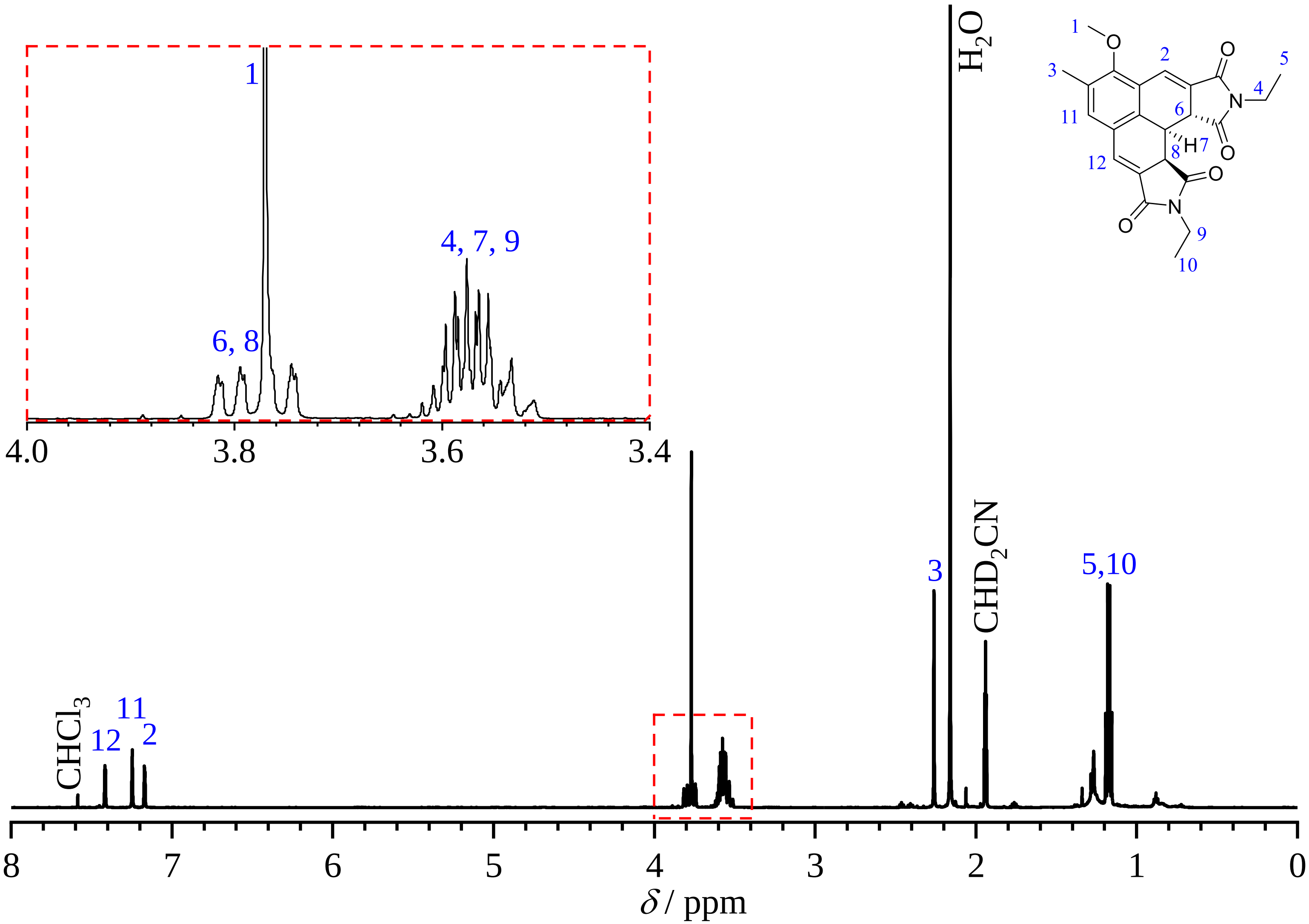

Figure S64 ^{1}H NMR spectrum and assigned resonances of (11a*R*,11b*R*,11c*R*)-2,10-diethyl-5-methoxy-6-methyl-11b,11c-dihydro-1*H*-pyrrolo[3',4':2,3]naphtho[1,8-*ef*]isoindole-1,3,9,11(2*H*,10*H*,11a*H*)-tetraone (**5a**) recorded in CD_3CN.

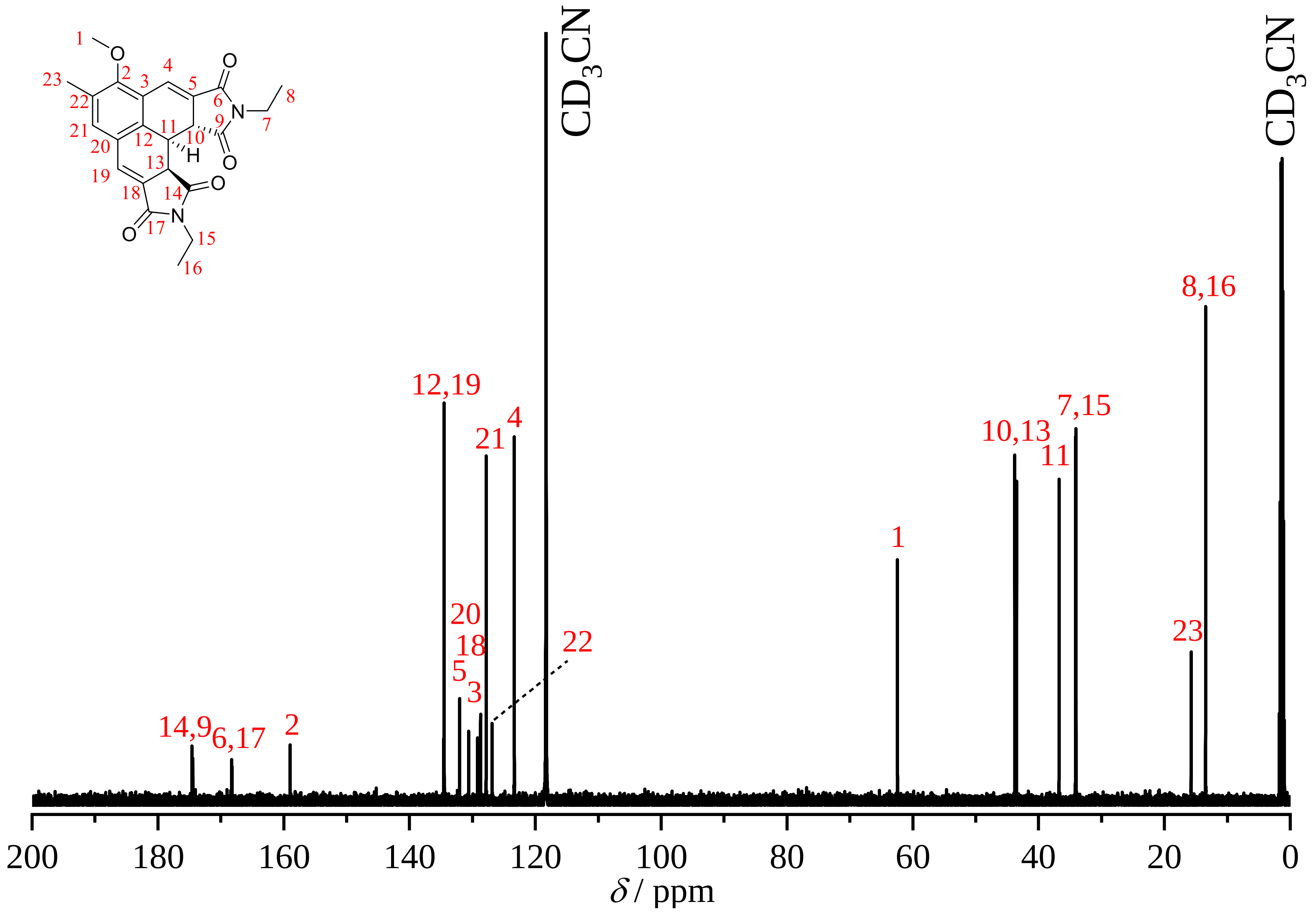

Figure S65 ^{13}C NMR spectrum and assigned resonances of (11a*R*,11b*R*,11c*R*)-2,10-diethyl-5-methoxy-6-methyl-11b,11c-dihydro-1*H*-pyrrolo[3',4':2,3]naphtho[1,8-*ef*]isoindole-1,3,9,11(2*H*,10*H*,11a*H*)-tetraone (**5a**) recorded in CD_3CN.

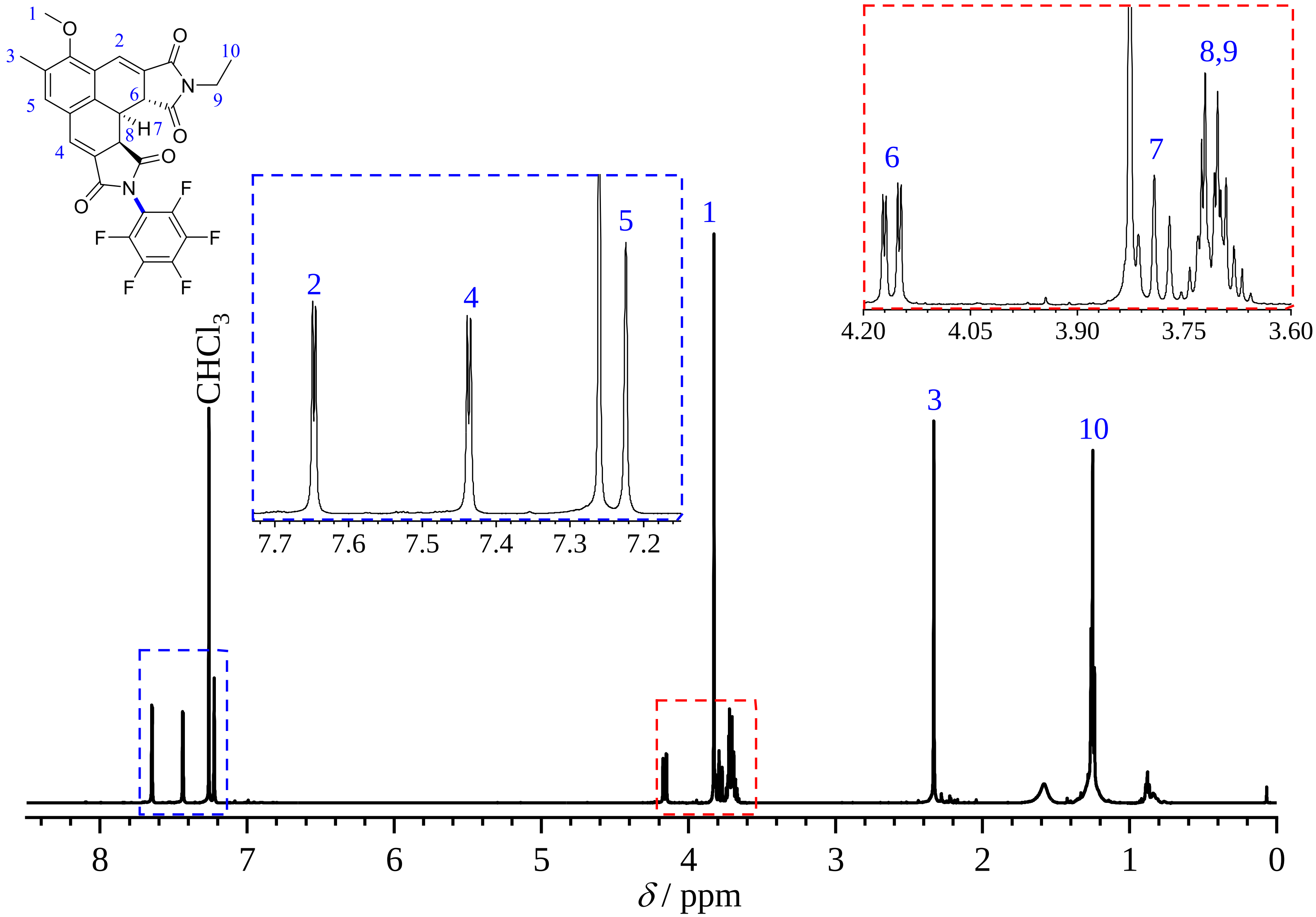

Figure S66 ^{1}H NMR spectrum and assigned resonances of (11a*R*,11b*S*,11c*R*)-2-ethyl-5-methoxy-6-methyl-10-(perfluorophenyl)-11b,11c-dihydro-1*H*-pyrrolo[3',4':2,3]naphtho[1,8-*ef*]isoindole-1,3,9,11(2*H*,10*H*,11a*H*)-tetraone (**5d**) recorded in CD_3CN.

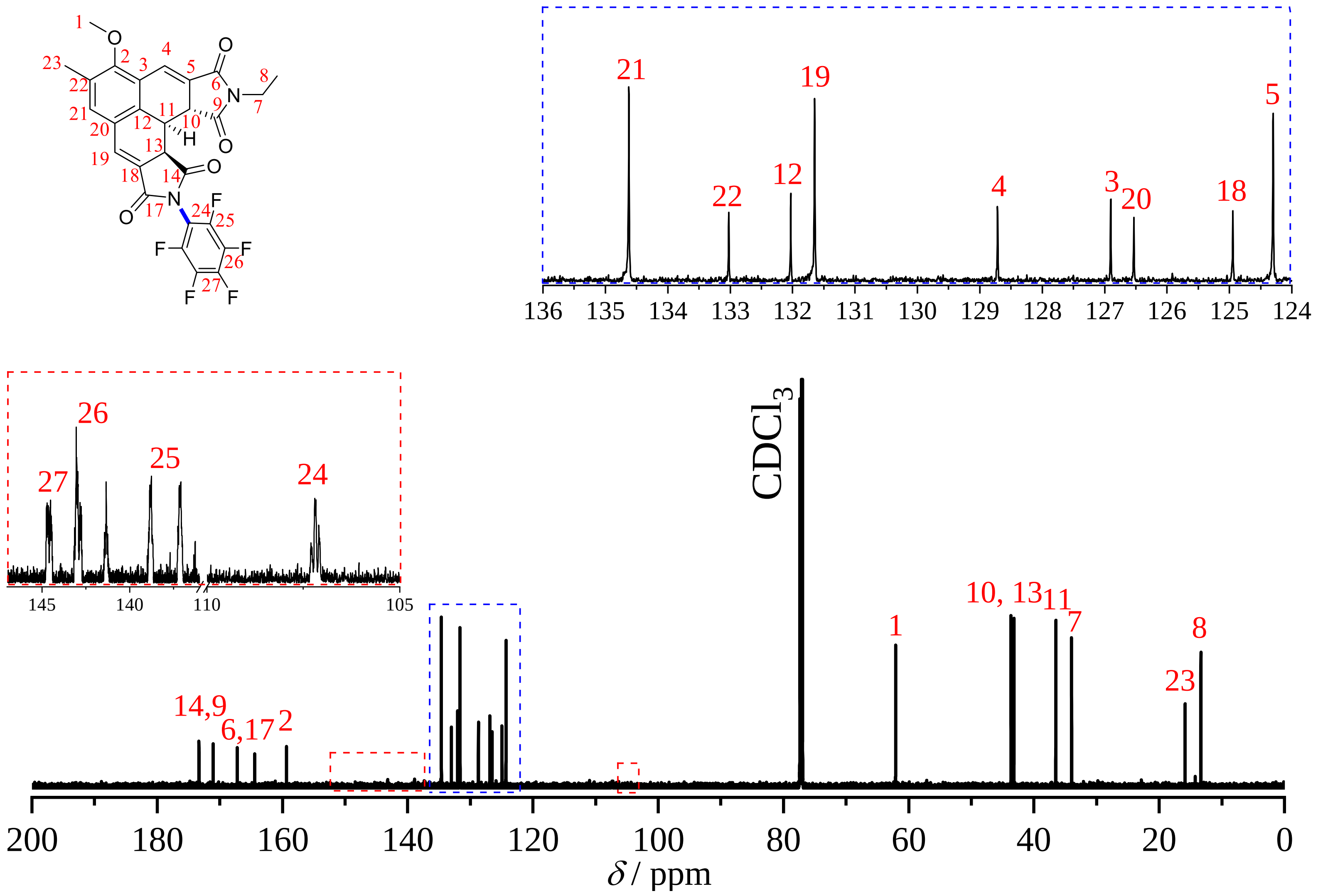

Figure S67 ^{13}C NMR spectrum and assigned resonances of (11a*R*,11b*S*,11c*R*)-2-ethyl-5-methoxy-6-methyl-10-(perfluorophenyl)-11b,11c-dihydro-1*H*-pyrrolo[3',4':2,3]naphtho[1,8-*ef*]isoindole-1,3,9,11(2*H*,10*H*,11a*H*)-tetraone (**5d**) recorded in CD_3CN.

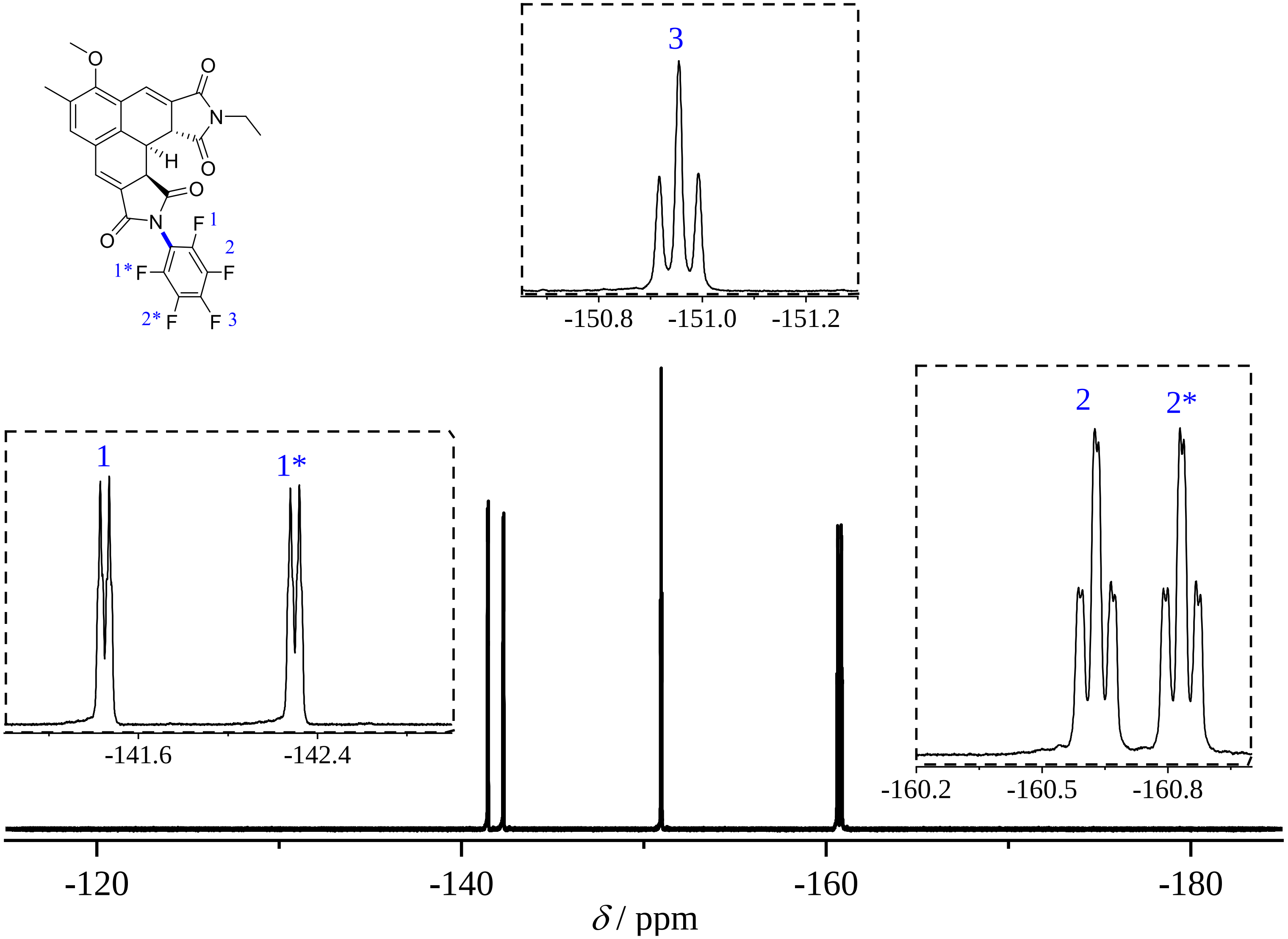

Figure S68 ^{19}F NMR spectrum and assigned resonances of (11a*R*,11b*S*,11c*R*)-2-ethyl-5-methoxy-6-methyl-10-(perfluorophenyl)-11b,11c-dihydro-1*H*-pyrrolo[3',4':2,3]naphtho[1,8-*ef*]isoindole-1,3,9,11(2*H*,10*H*,11a*H*)-tetraone (**5d**) recorded in CD_3CN.

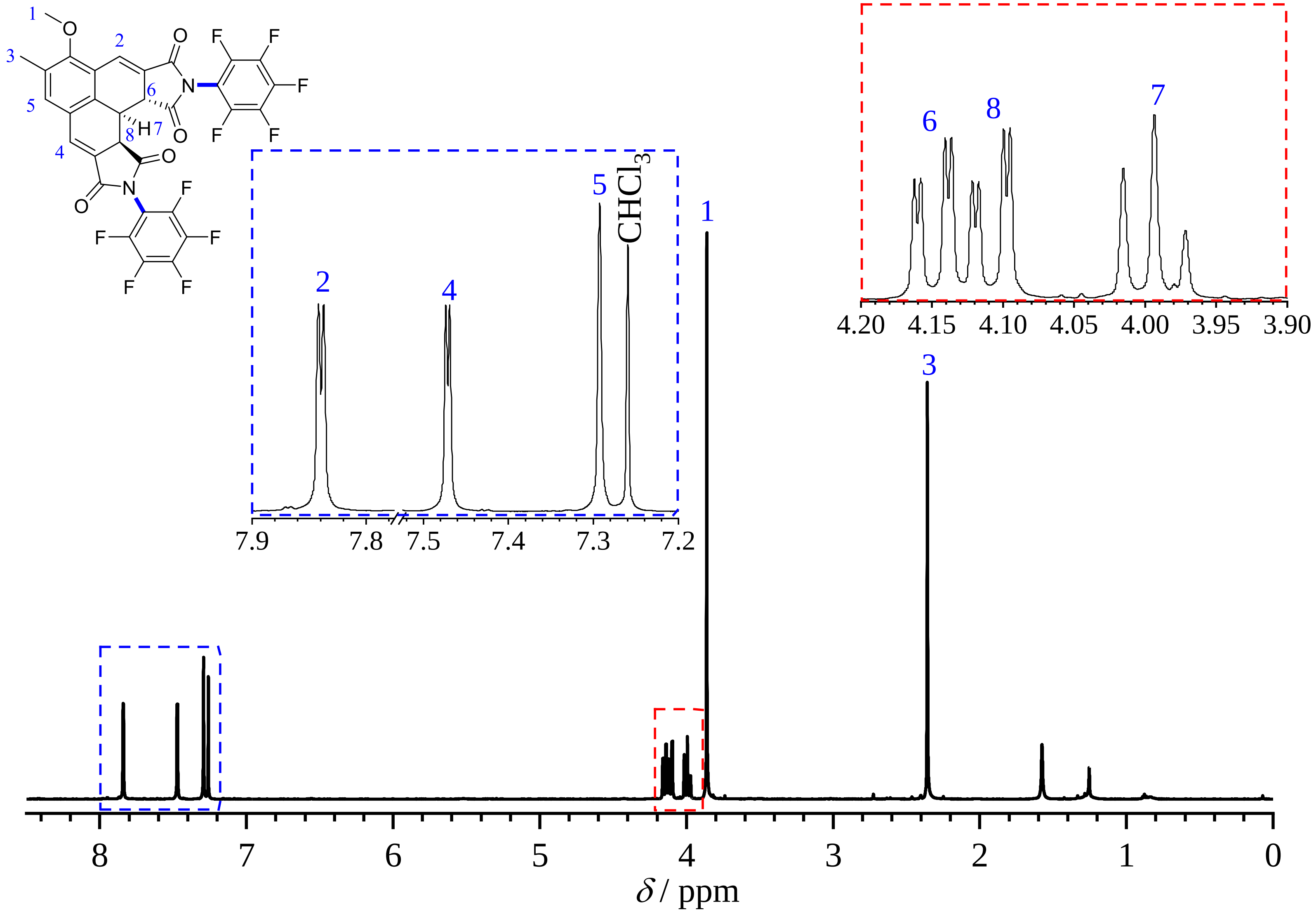

Figure S69 ^{1}C NMR spectrum and assigned resonances of (11a*R*,11b*R*,11c*R*)-5-methoxy-6-methyl-2,10-bis(perfluorophenyl)-11b,11c-dihydro-1*H*-pyrrolo[3',4':2,3]naphtho[1,8-*ef*]isoindole-1,3,9,11(2*H*,10*H*,11a*H*)-tetraone (**5f**) recorded in CD_3CN.

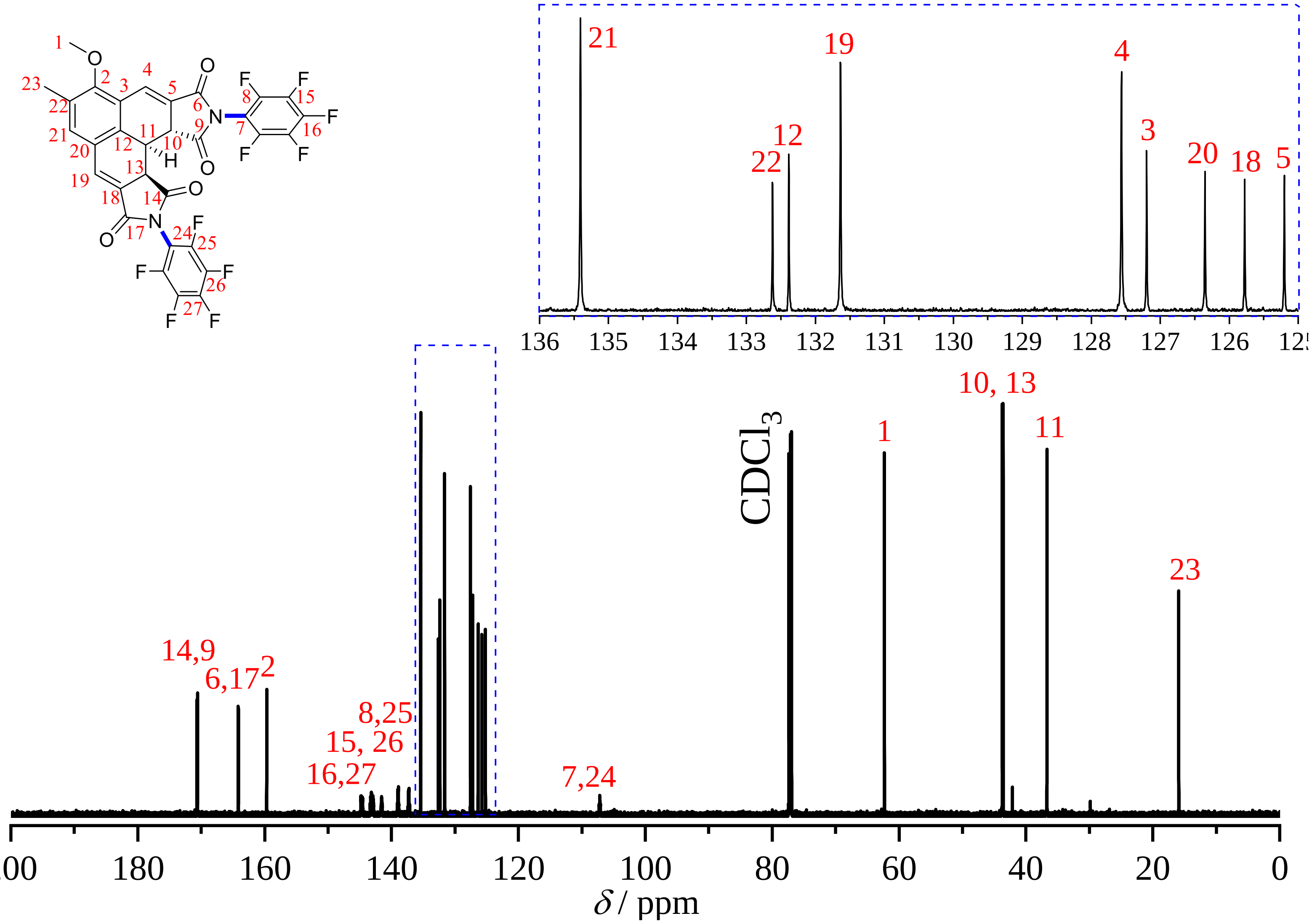

Figure S70 ^{13}C NMR spectrum and assigned resonances of (11a*R*,11b*R*,11c*R*)-5-methoxy-6-methyl-2,10-bis(perfluorophenyl)-11b,11c-dihydro-1*H*-pyrrolo[3',4':2,3]naphtho[1,8-*ef*]isoindole-1,3,9,11(2*H*,10*H*,11a*H*)-tetraone (**5f**) recorded in CD_3CN.

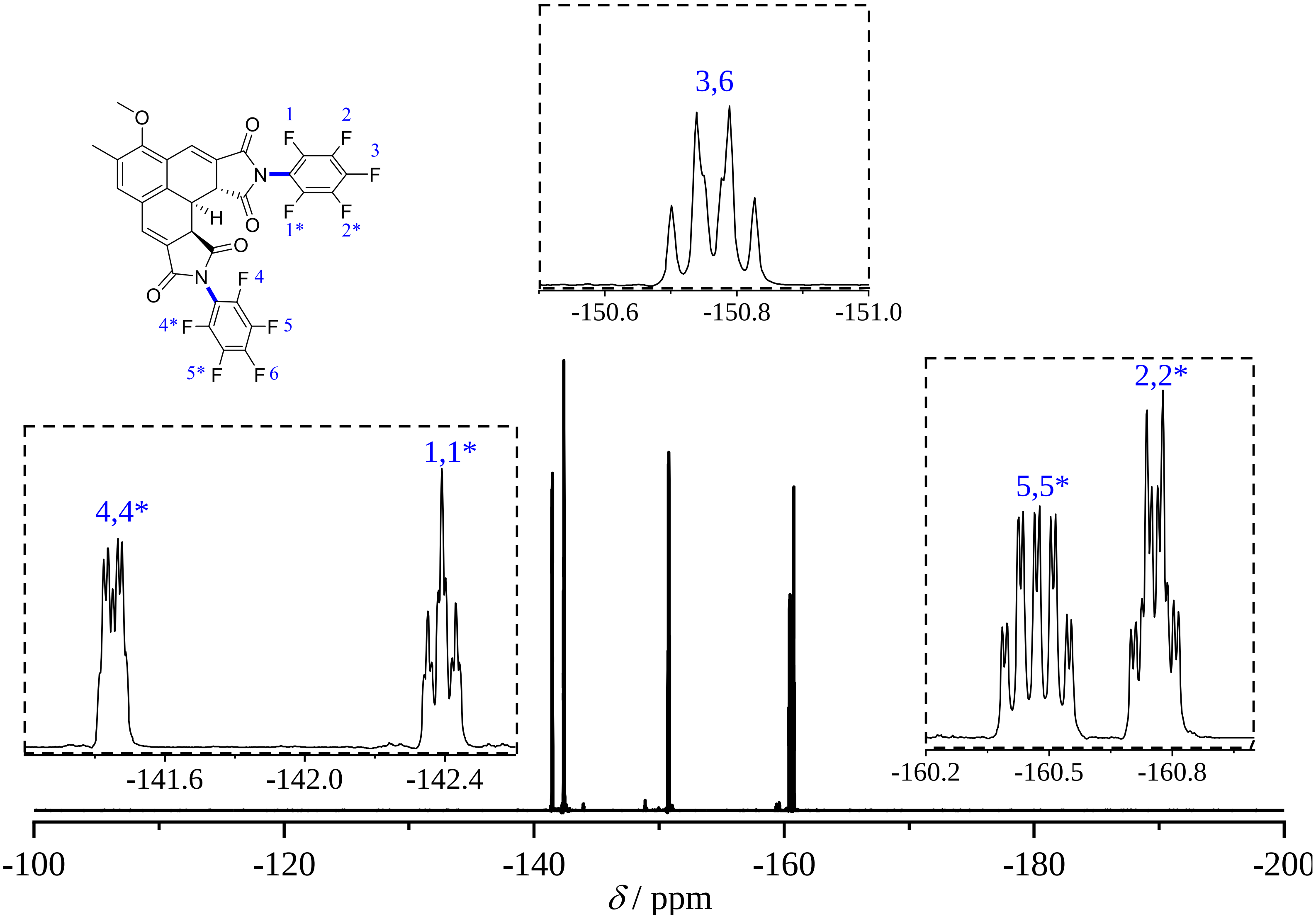

Figure S71 ^{19}F NMR spectrum and assigned resonances of (11a*R*,11b*R*,11c*R*)-5-methoxy-6-methyl-2,10-bis(perfluorophenyl)-11b,11c-dihydro-1*H*-pyrrolo[3',4':2,3]naphtho[1,8-*ef*]isoindole-1,3,9,11(2*H*,10*H*,11a*H*)-tetraone (**5f**) recorded in CD_3CN.

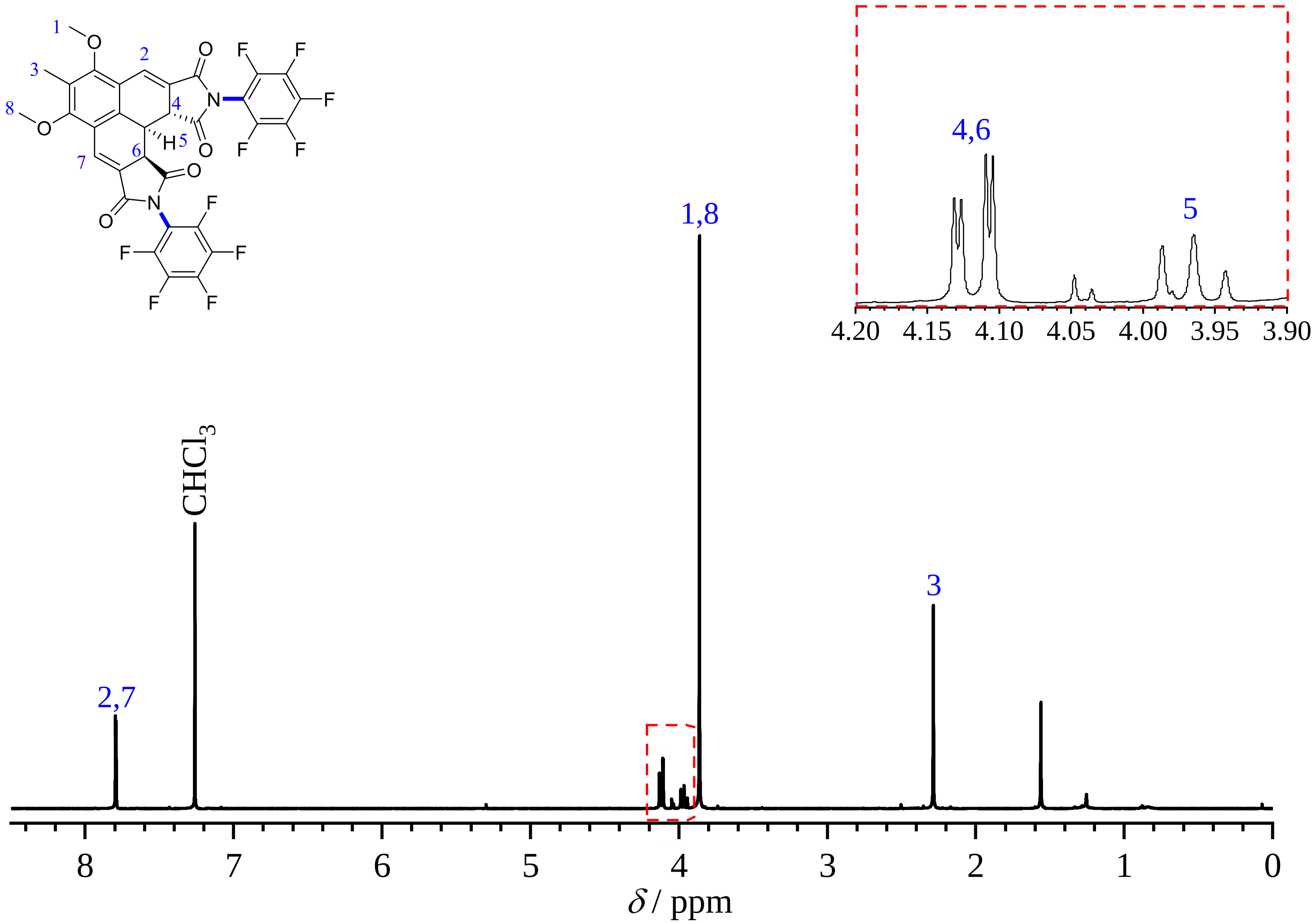

Figure S72 ^{1}H NMR spectrum and assigned resonances of (11a*R*,11c*R*)-5,7-dimethoxy-6-methyl-2,10-bis(perfluorophenyl)-11b,11c-dihydro-1*H*-pyrrolo[3',4':2,3]naphtho[1,8-*ef*]isoindole-1,3,9,11(2*H*,10*H*,11a*H*)-tetraone (**5g**) recorded in CD_3CN.

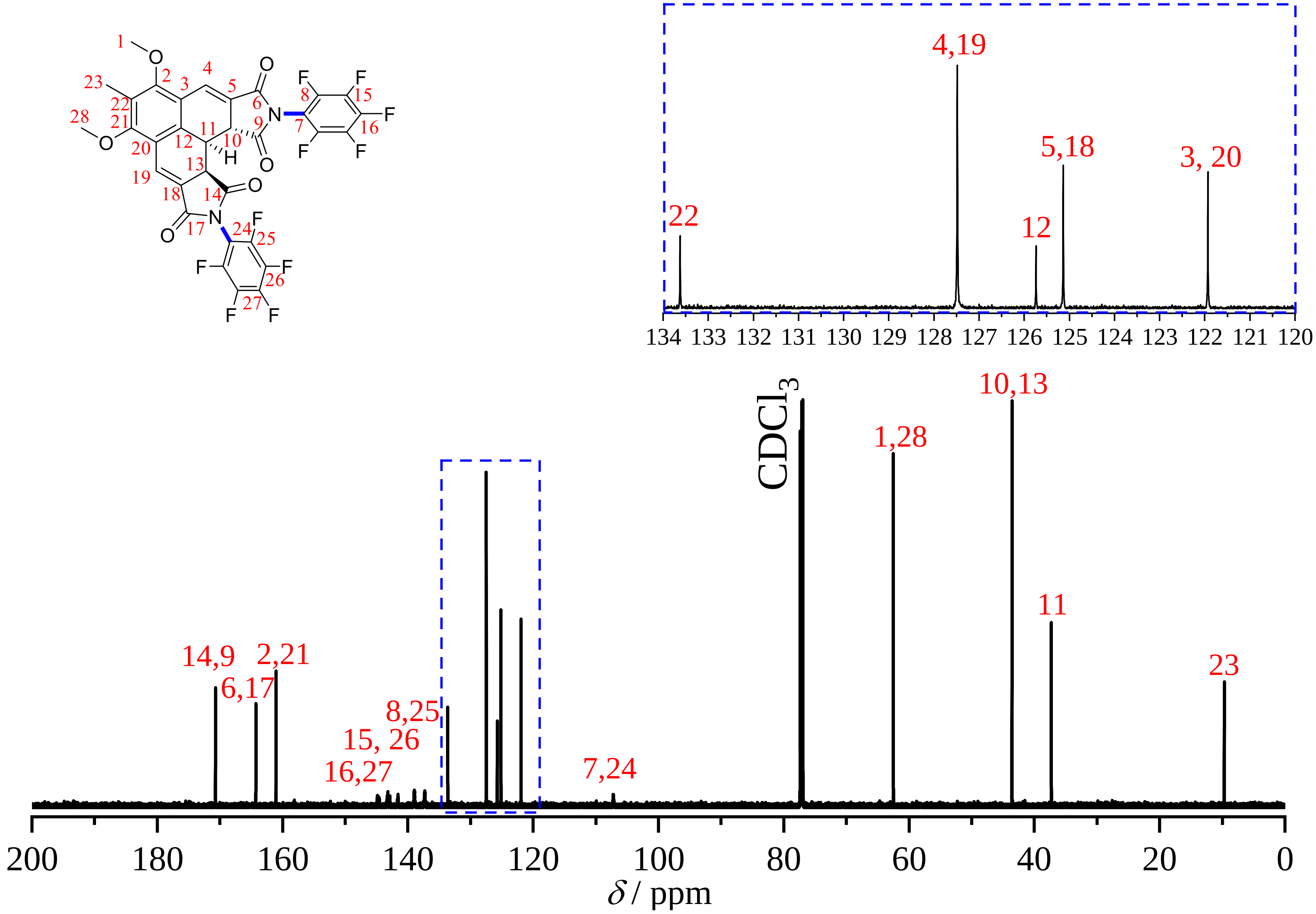

Figure S73 ^{13}C NMR spectrum and assigned resonances of (11a*R*,11c*R*)-5,7-dimethoxy-6-methyl-2,10-bis(perfluorophenyl)-11b,11c-dihydro-1*H*-pyrrolo[3',4':2,3]naphtho[1,8-*ef*]isoindole-1,3,9,11(2*H*,10*H*,11a*H*)-tetraone (**5g**) recorded in CD_3CN.

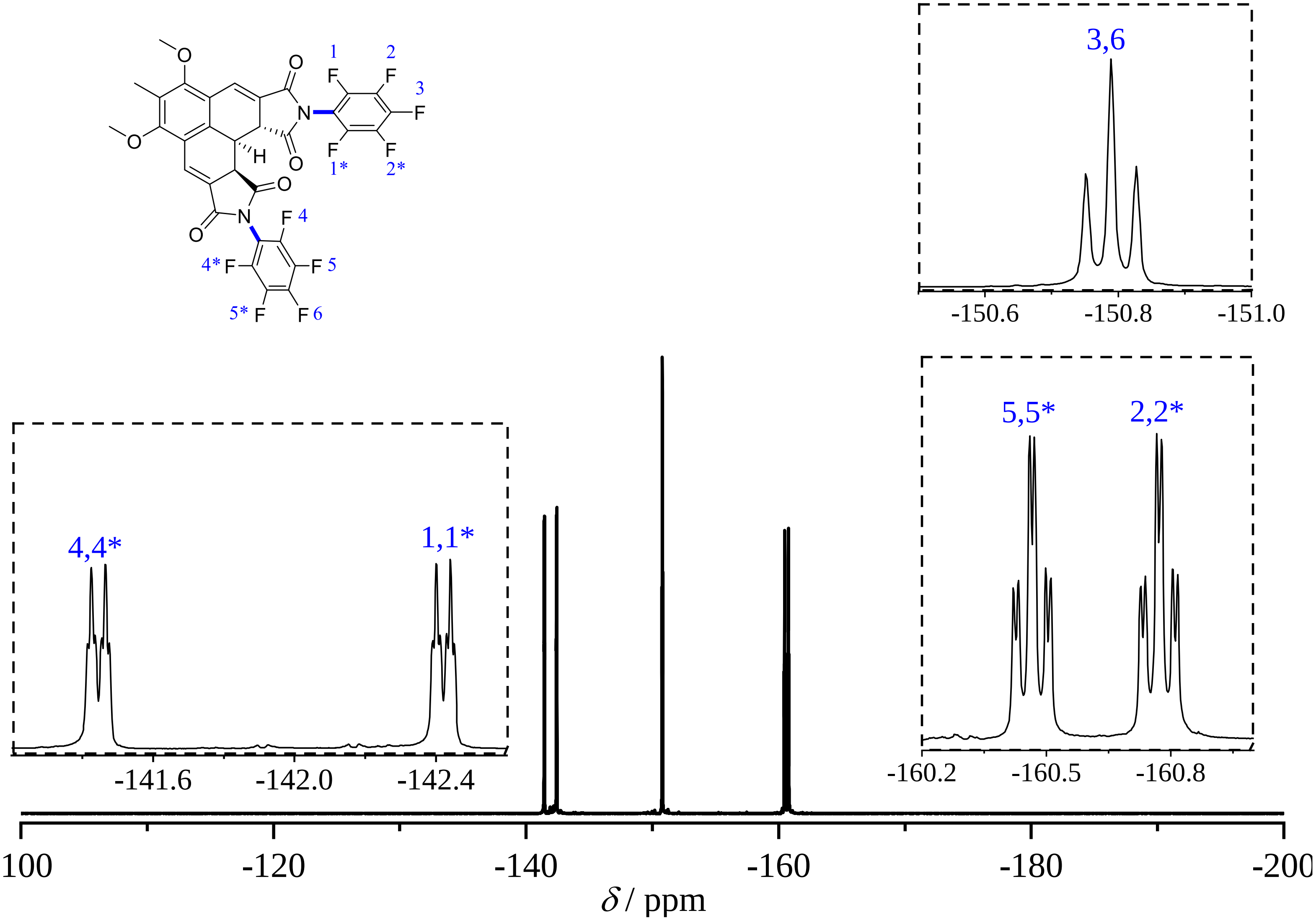

Figure S74 ^{19}F NMR spectrum and assigned resonances of (11a*R*,11c*R*)-5,7-dimethoxy-6-methyl-2,10-bis(perfluorophenyl)-11b,11c-dihydro-1*H*-pyrrolo[3',4':2,3]naphtho[1,8-*ef*]isoindole-1,3,9,11(2*H*,10*H*,11a*H*)-tetraone (**5g**) recorded in CD_3CN.

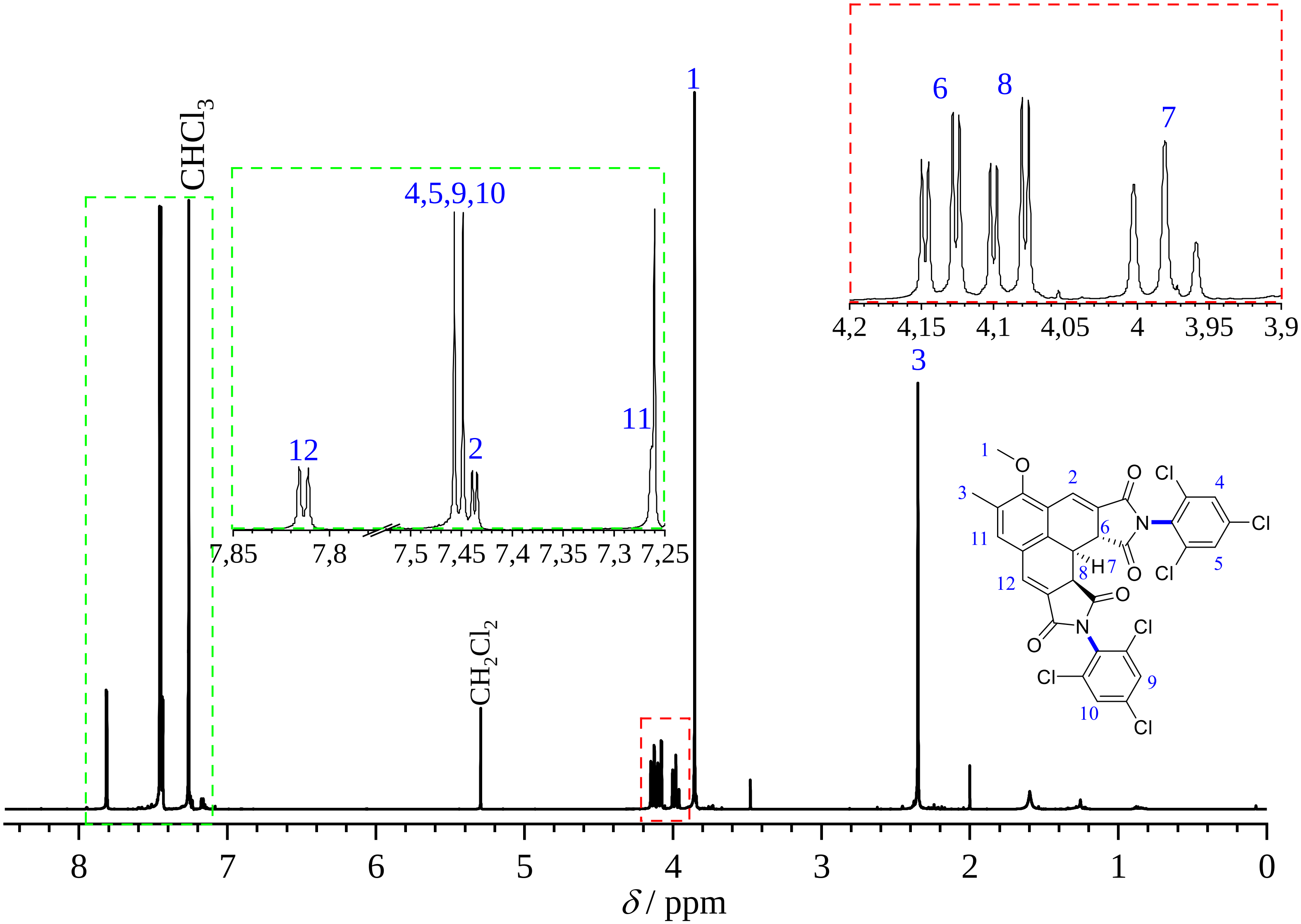

Figure S75 ^{1}H NMR spectrum and assigned resonances of (11a*R*,11b*R*,11c*R*)-5-methoxy-6-methyl-2,10-bis(2,4,6-trichlorophenyl)-11b,11c-dihydro-1*H*-pyrrolo[3',4':2,3]naphtho[1,8-*ef*]isoindole-1,3,9,11(2*H*,10*H*,11a*H*)-tetraone (**5h**) recorded in CD_3CN.

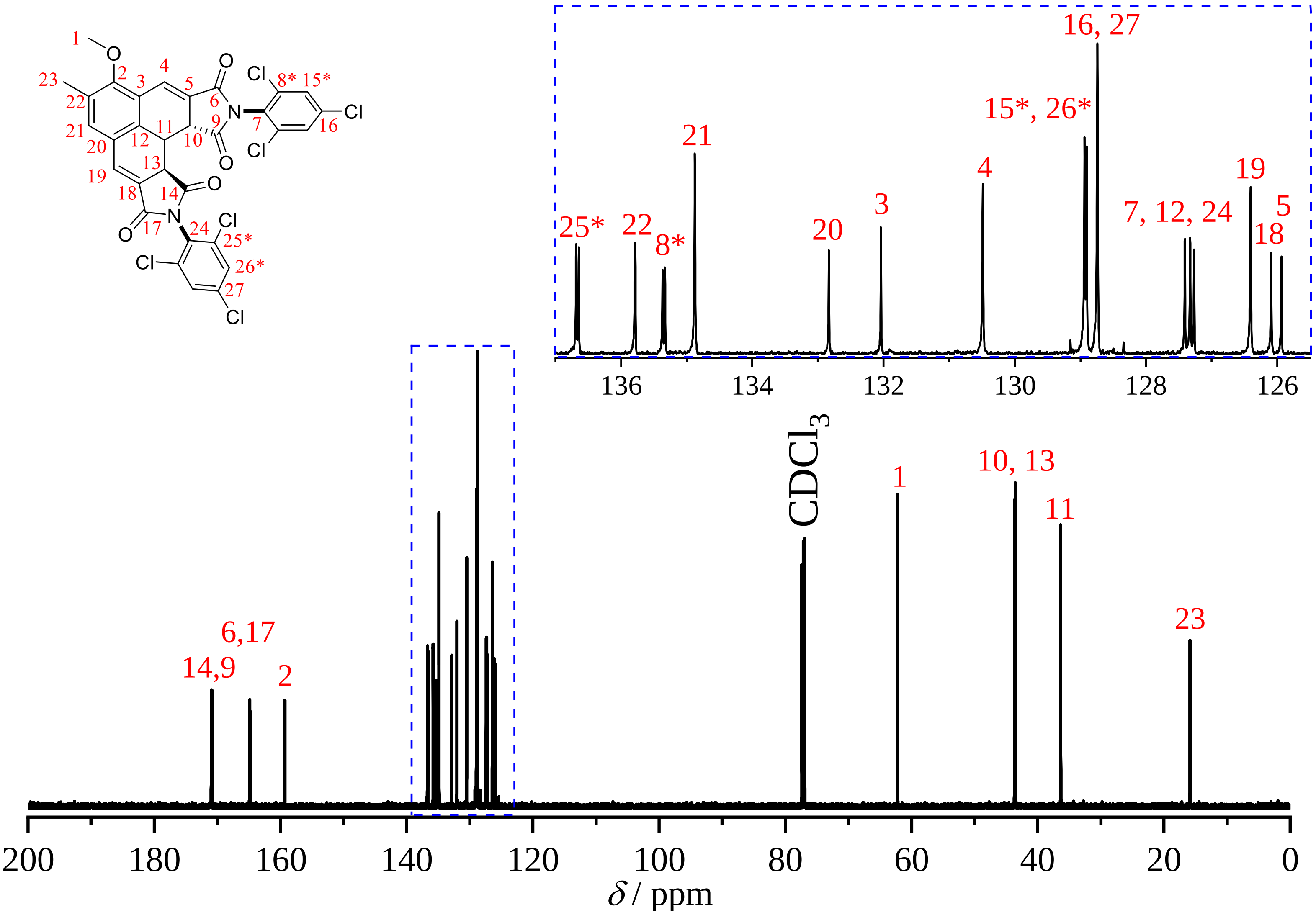

Figure S76 ^{13}C NMR spectrum and assigned resonances of (11a*R*,11b*R*,11c*R*)-5-methoxy-6-methyl-2,10-bis(2,4,6-trichlorophenyl)-11b,11c-dihydro-1*H*-pyrrolo[3',4':2,3]naphtho[1,8-*ef*]isoindole-1,3,9,11(2*H*,10*H*,11a*H*)-tetraone (**5h**) recorded in CD_3CN.

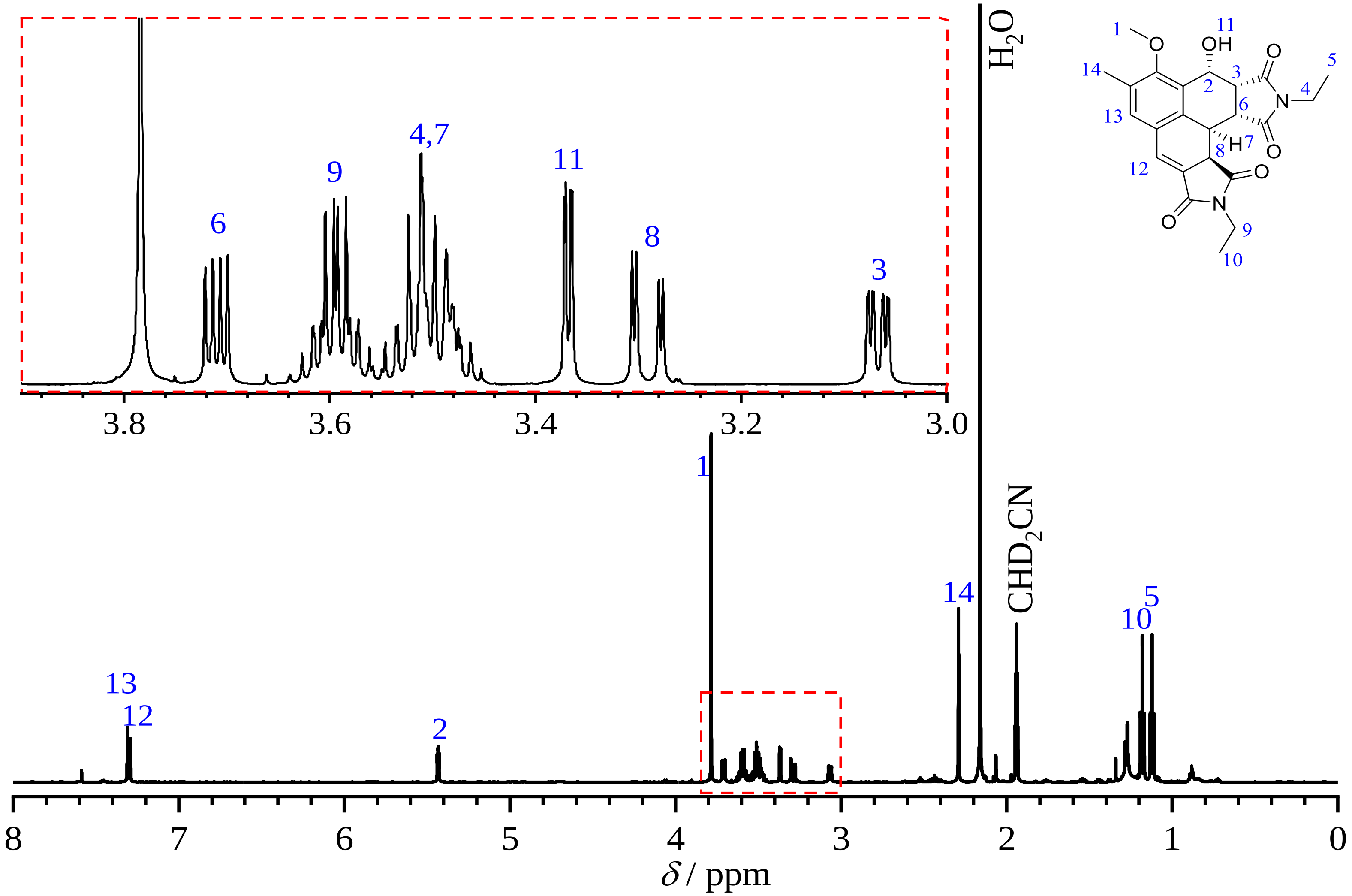

Figure S77 ^{1}H NMR spectrum and assigned resonances of (3a*R*,4*R*,11a*R*,11b*R*,11c*R*)-2,10-diethyl-4-hydroxy-7-methoxy-6-methyl-3a,11a,11b,11c-tetrahydro-1*H*-pyrrolo[3',4':2,3]naphtho[1,8-*ef*]isoindole-1,3,9,11(2*H*,4*H*,10*H*)-tetraone (**7a**) in CD_3CN.

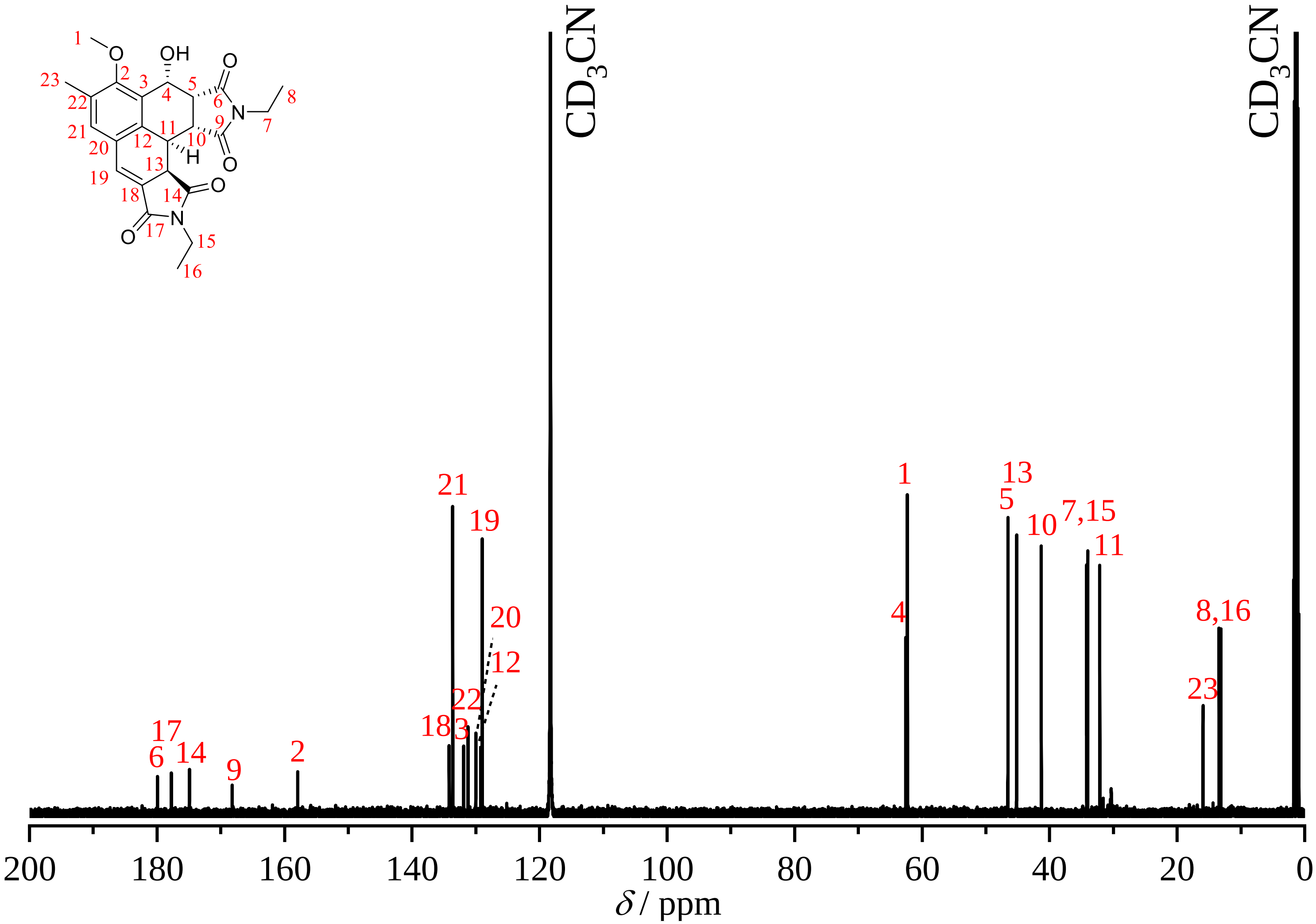

Figure S78 ^{13}C NMR spectrum and assigned resonances of (3a*R*,4*R*,11a*R*,11b*R*,11c*R*)-2,10-diethyl-4-hydroxy-7-methoxy-6-methyl-3a,11a,11b,11c-tetrahydro-1*H*-pyrrolo[3',4':2,3]naphtho[1,8-*ef*]isoindole-1,3,9,11(2*H*,4*H*,10*H*)-tetraone (**7a**) in CD_3CN.

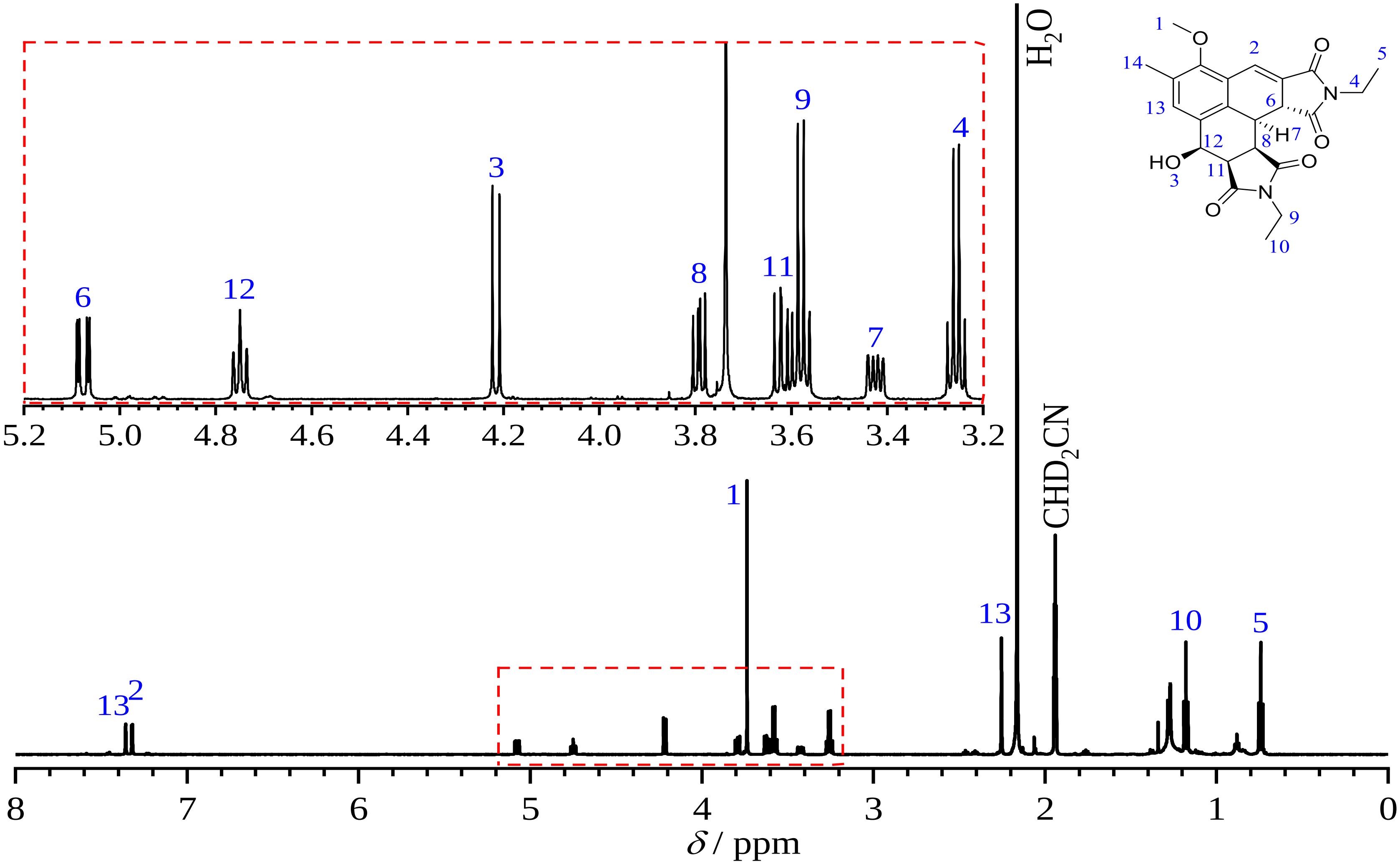

Figure S79 ^{1}H NMR spectrum and assigned resonances of (3a*R*,4*R*,11a*R*,11b*S*,11c*R*)-2,10-diethyl-4-hydroxy-5-methoxy-6-methyl-3a,11a,11b,11c-tetrahydro-1H-pyrrolo[3',4':2,3]naphtho[1,8-*ef*]isoindole-1,3,9,11(2*H*,4*H*,10*H*)-tetraone (**8a**), recorded in CD_3CN.

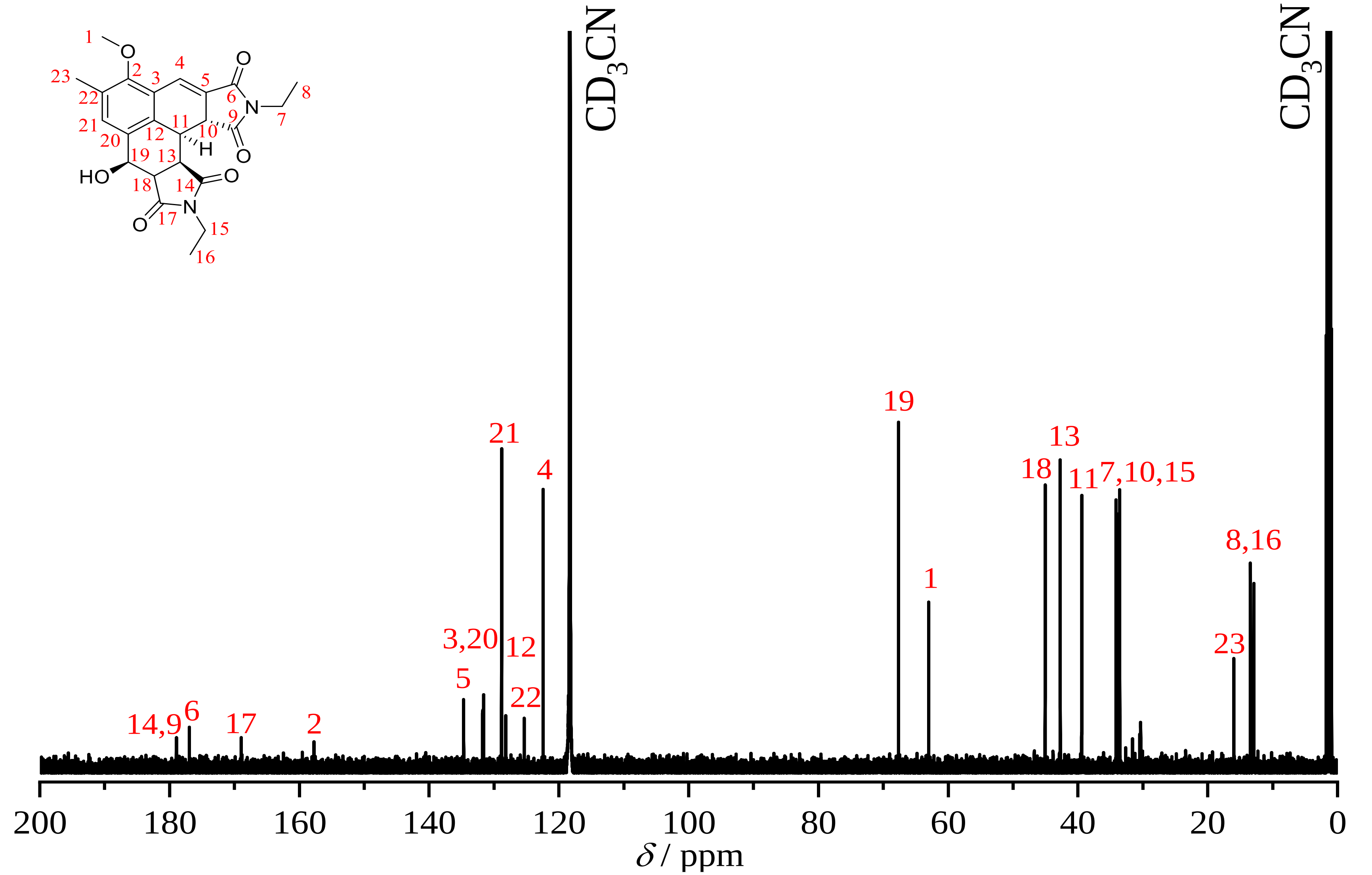

Figure S80 ^{13}C NMR spectrum and assigned resonances of (3a*R*,4*R*,11a*R*,11b*S*,11c*R*)-2,10-diethyl-4-hydroxy-5-methoxy-6-methyl-3a,11a,11b,11c-tetrahydro-1H-pyrrolo[3',4':2,3]naphtho[1,8-*ef*]isoindole-1,3,9,11(2*H*,4*H*,10*H*)-tetraone (**8a**), recorded in CD_3CN.

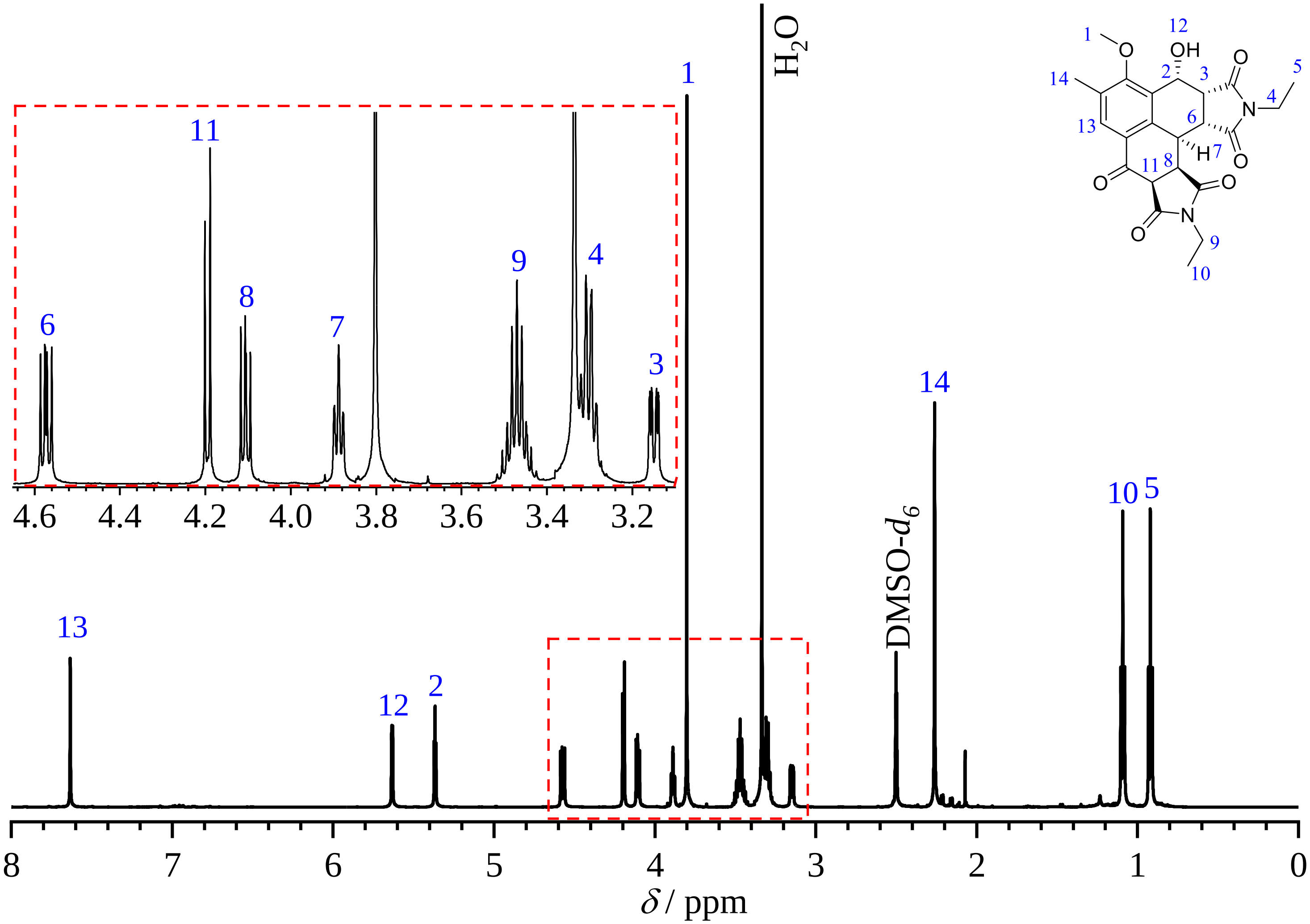

Figure S81 ^{1}H NMR spectrum and assigned resonances of (3a*R*,8*R*,8a*R*,11a*R*,11b*S*,11c*R*)-2,10-diethyl-8-hydroxy-7-methoxy-6-methyl-3a,8,8a,11a,11b,11c-hexahydro-1*H*-pyrrolo[3',4':2,3]naphtho[1,8-*ef*]isoindole-1,3,4,9,11(2*H*,10*H*)-pentaone (**9a**), recorded in DMSO-d_6.

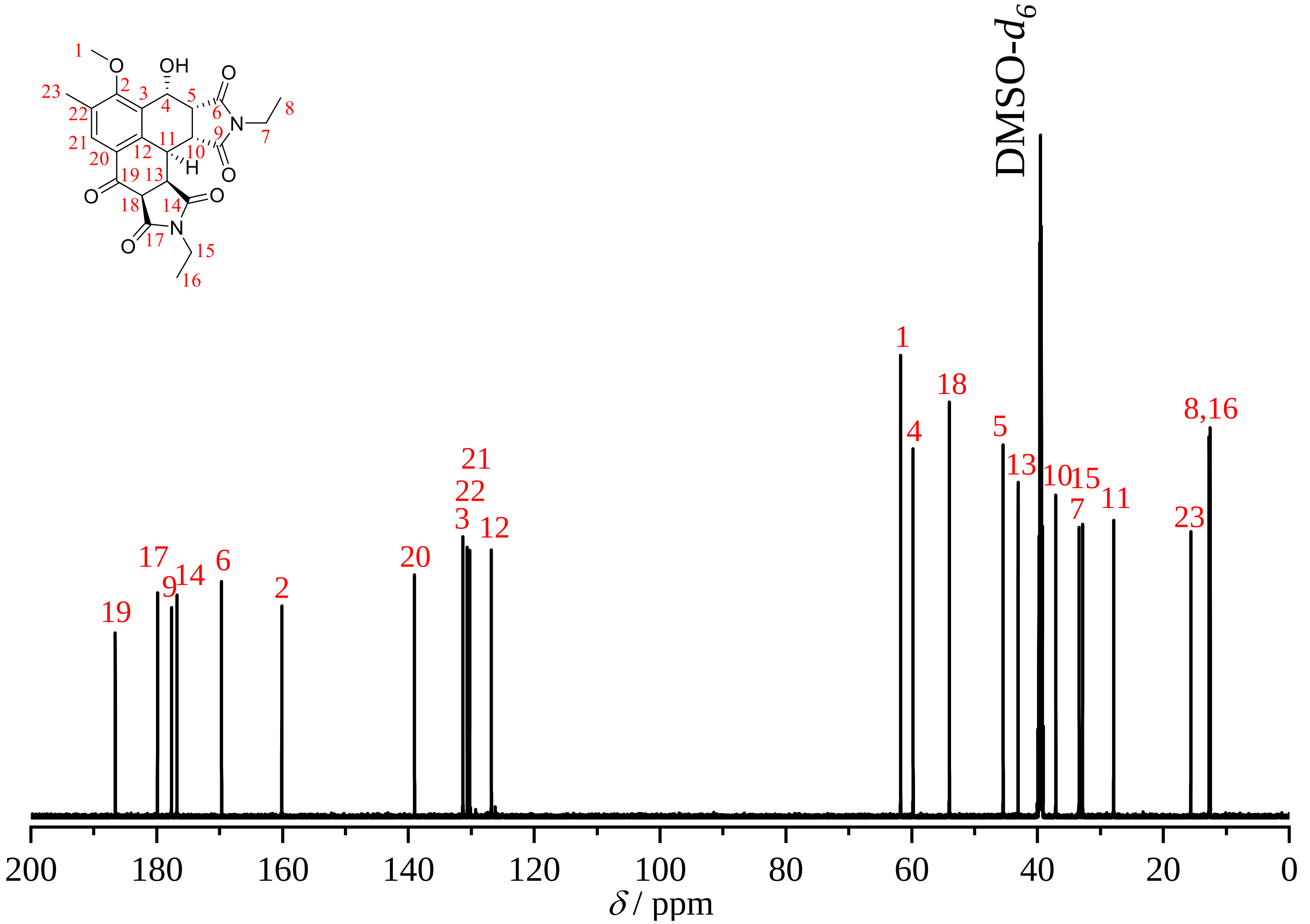

Figure S82 ^{13}C NMR spectrum and assigned resonances of (3a*R*,8*R*,8a*R*,11a*R*,11b*S*,11c*R*)-2,10-diethyl-8-hydroxy-7-methoxy-6-methyl-3a,8,8a,11a,11b,11c-hexahydro-1*H*-pyrrolo[3',4':2,3]naphtho[1,8-*ef*]isoindole-1,3,4,9,11(2*H*,10*H*)-pentaone (**9a**), recorded in DMSO-d_6.

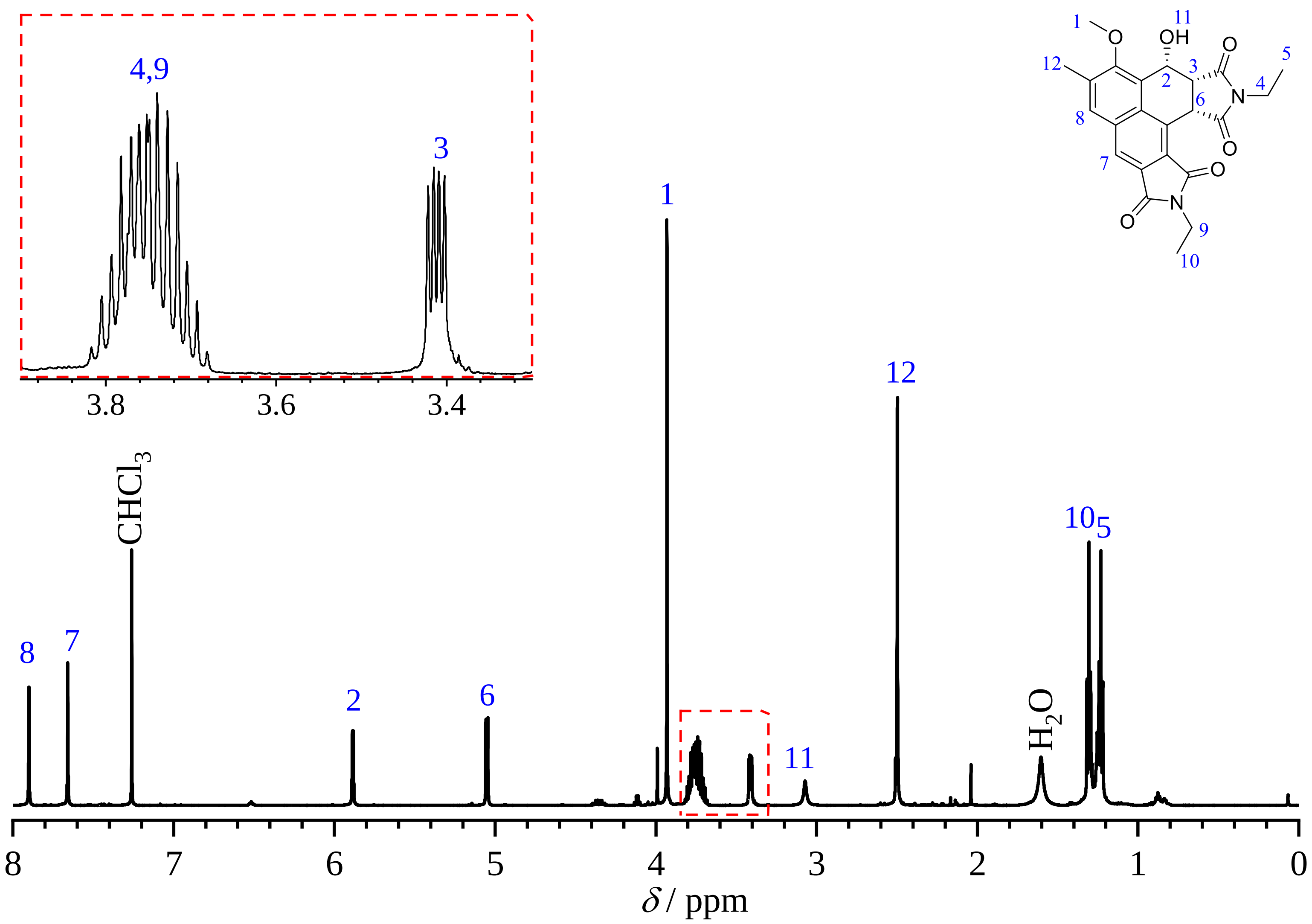

Figure S83 ^{1}H NMR spectrum and assigned resonances of (3a*R*,4*R*,11c*S*)-2,10-diethyl-4-hydroxy-5-methoxy-6-methyl-3a,11c-dihydro-1*H*-pyrrolo[3',4':2,3]naphtho[1,8-*ef*]isoindole-1,3,9,11(2*H*,4*H*,10*H*)-tetraone (**10a**), recorded in $CDCl_3$.

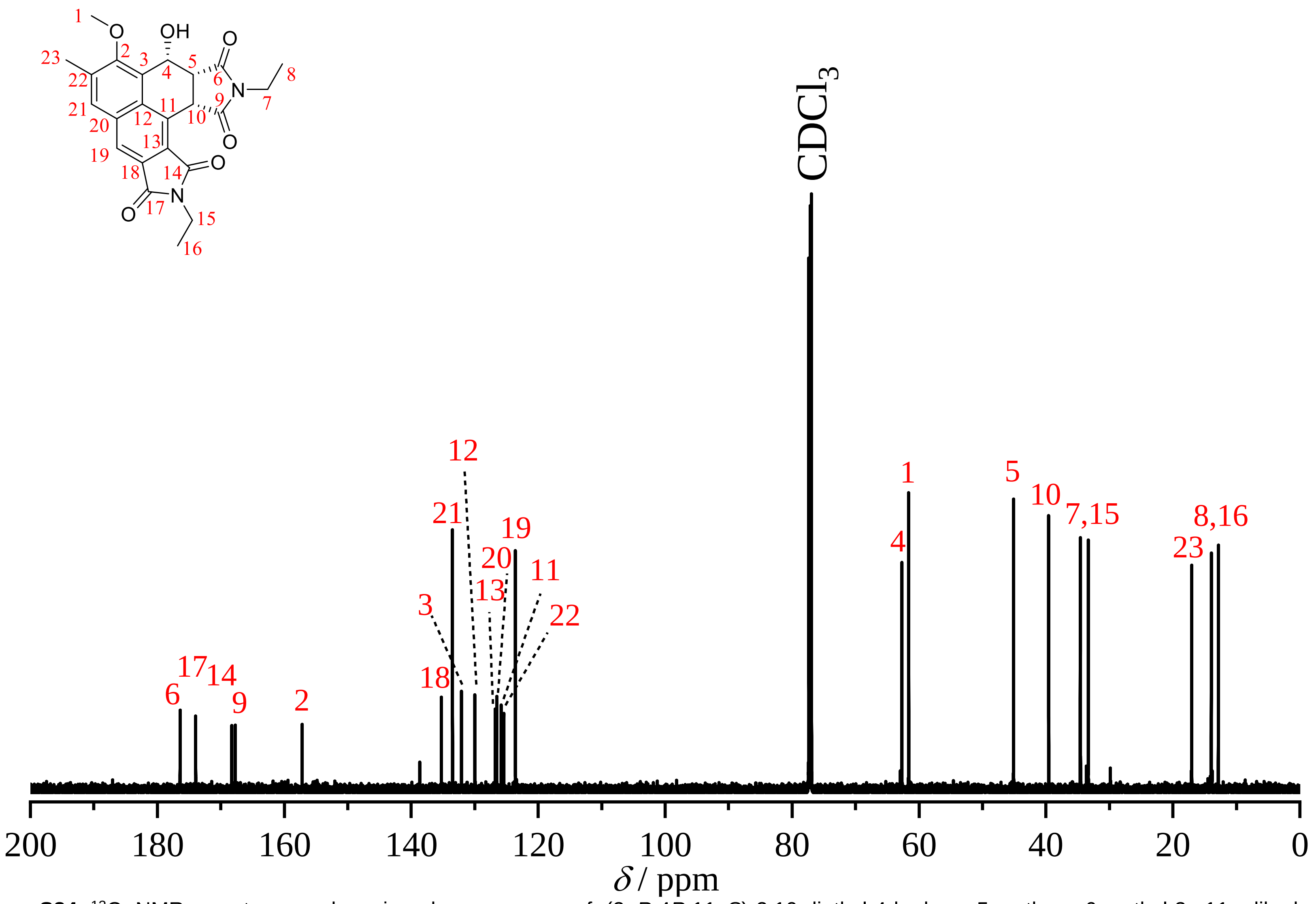

Figure S84 ^{13}C NMR spectrum and assigned resonances of (3a*R*,4*R*,11c*S*)-2,10-diethyl-4-hydroxy-5-methoxy-6-methyl-3a,11c-dihydro-1*H*-pyrrolo[3',4':2,3]naphtho[1,8-*ef*]isoindole-1,3,9,11(2*H*,4*H*,10*H*)-tetraone (**10a**), recorded in $CDCl_3$.

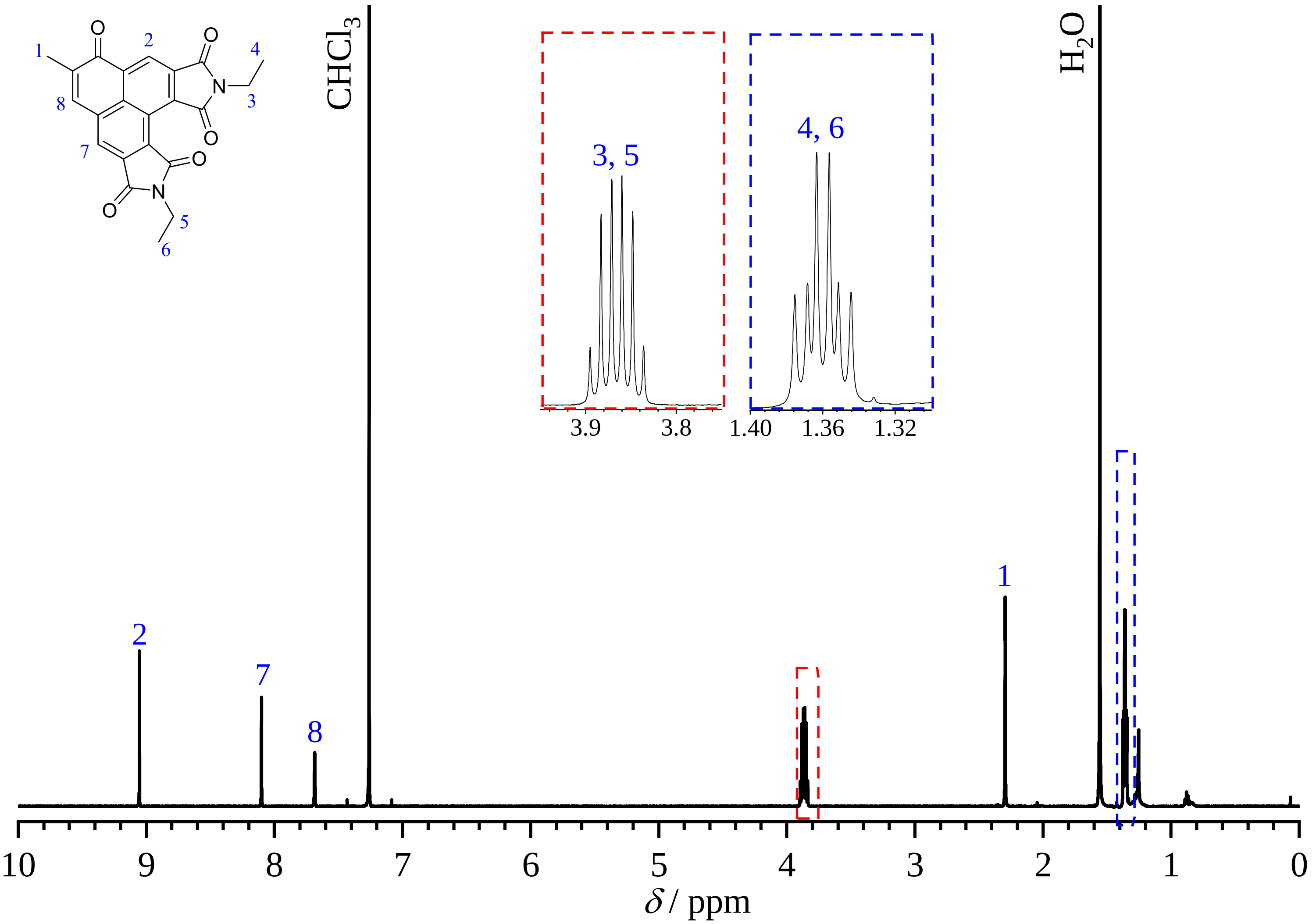

Figure S85 ^{1}H NMR spectrum and assigned resonances of 2,10-diethyl-6-methyl-1H-pyrrolo[3',4':2,3]naphtho[1,8-*ef*]isoindole-1,3,5,9,11(2*H*,10*H*)-pentaone (**11a**) recorded in $CDCl_3$.

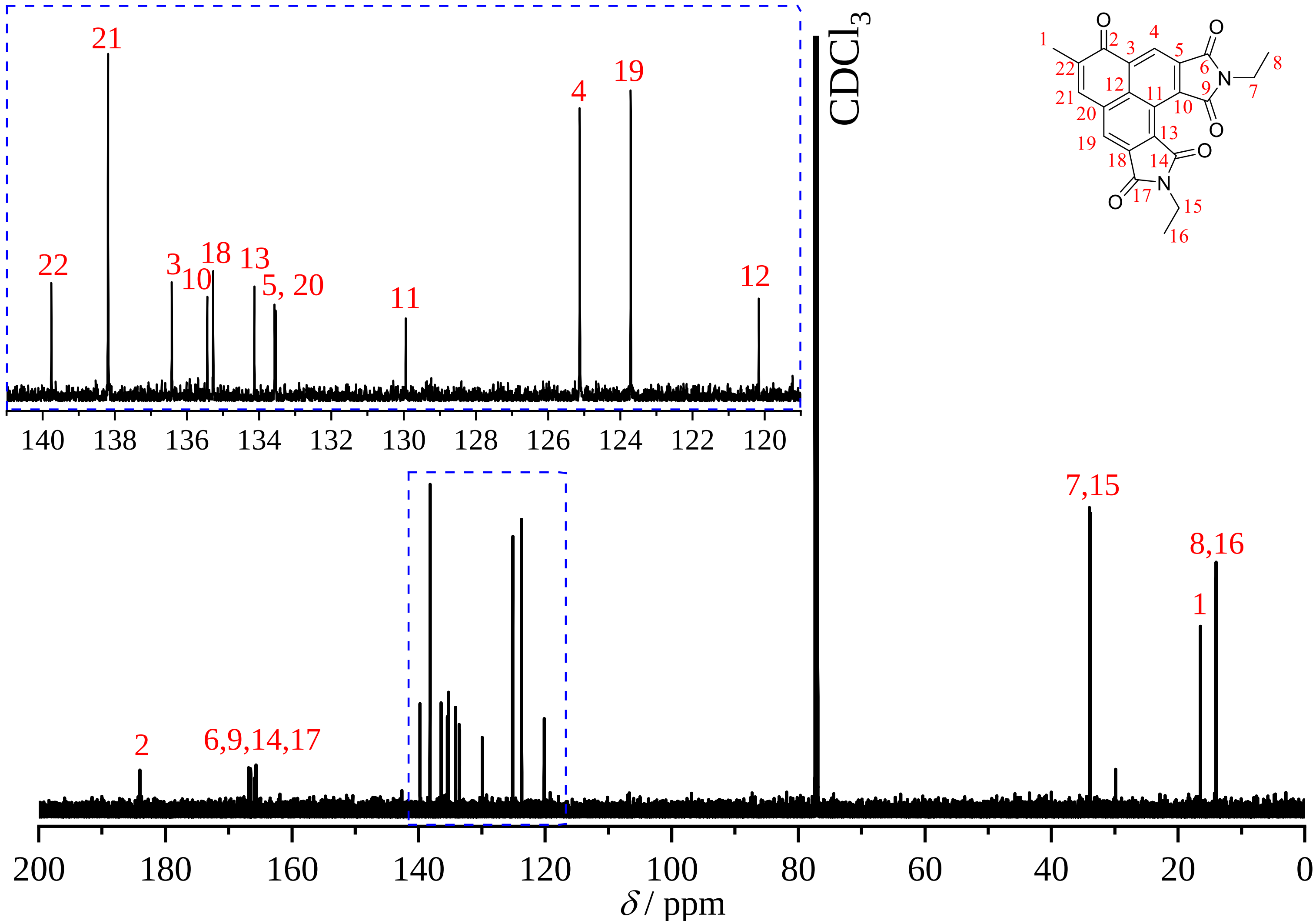

Figure S86 ^{13}C NMR spectrum and assigned resonances of 2,10-diethyl-6-methyl-1*H*-pyrrolo[3',4':2,3]naphtho[1,8-*ef*]isoindole-1,3,5,9,11(2*H*,10*H*)-pentaone (**11a**) recorded in CD_3CN.

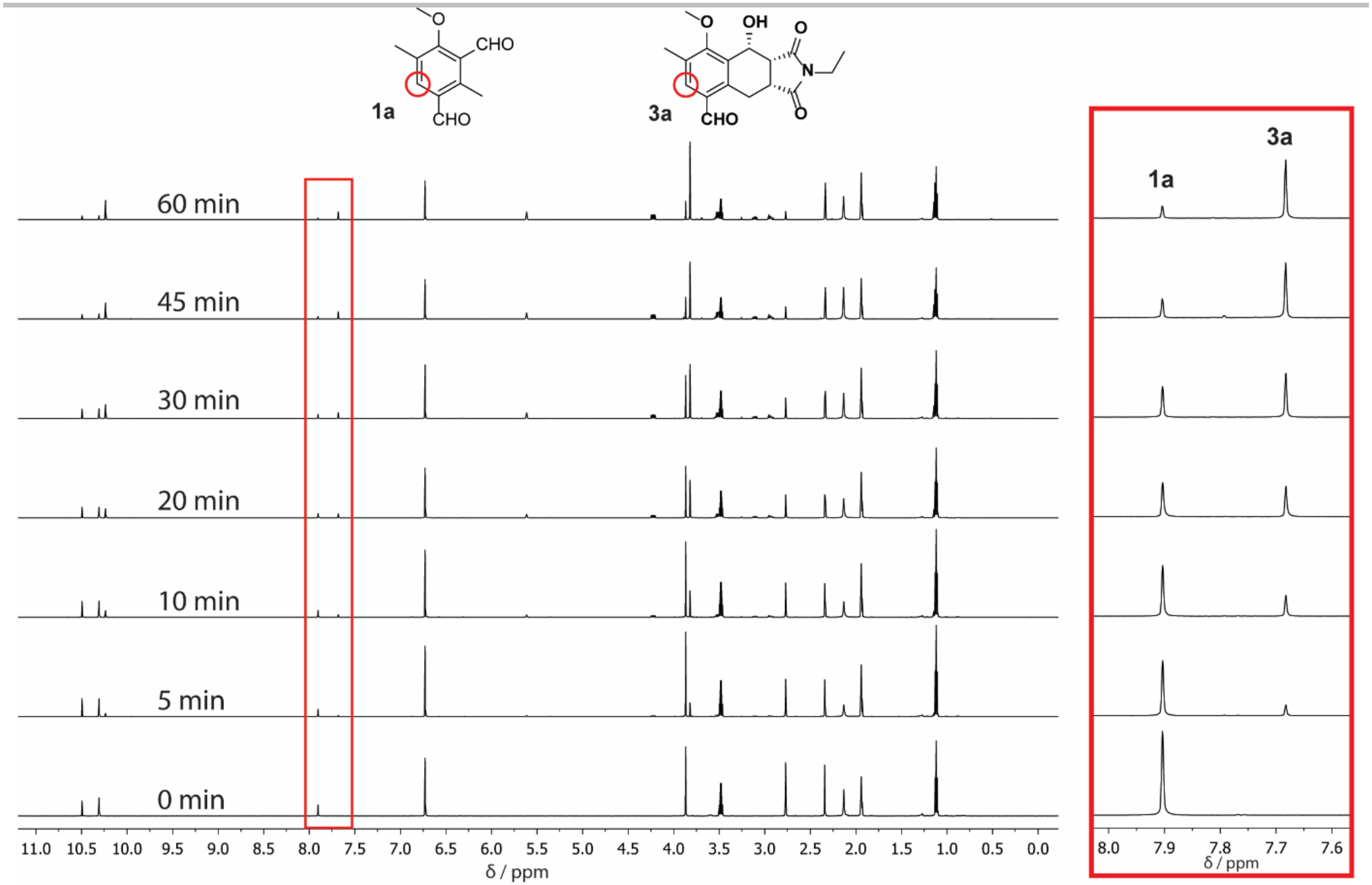

Figure S87 ^{1}H-NMR spectra of reaction solutions irradiated with for various times to determine the quantum yield of the reaction of **1a** and **2a** to form **3a** in CD_3CN. Conversions were determined using the circled proton signals in the highlighted region (7.6-8.0 ppm).

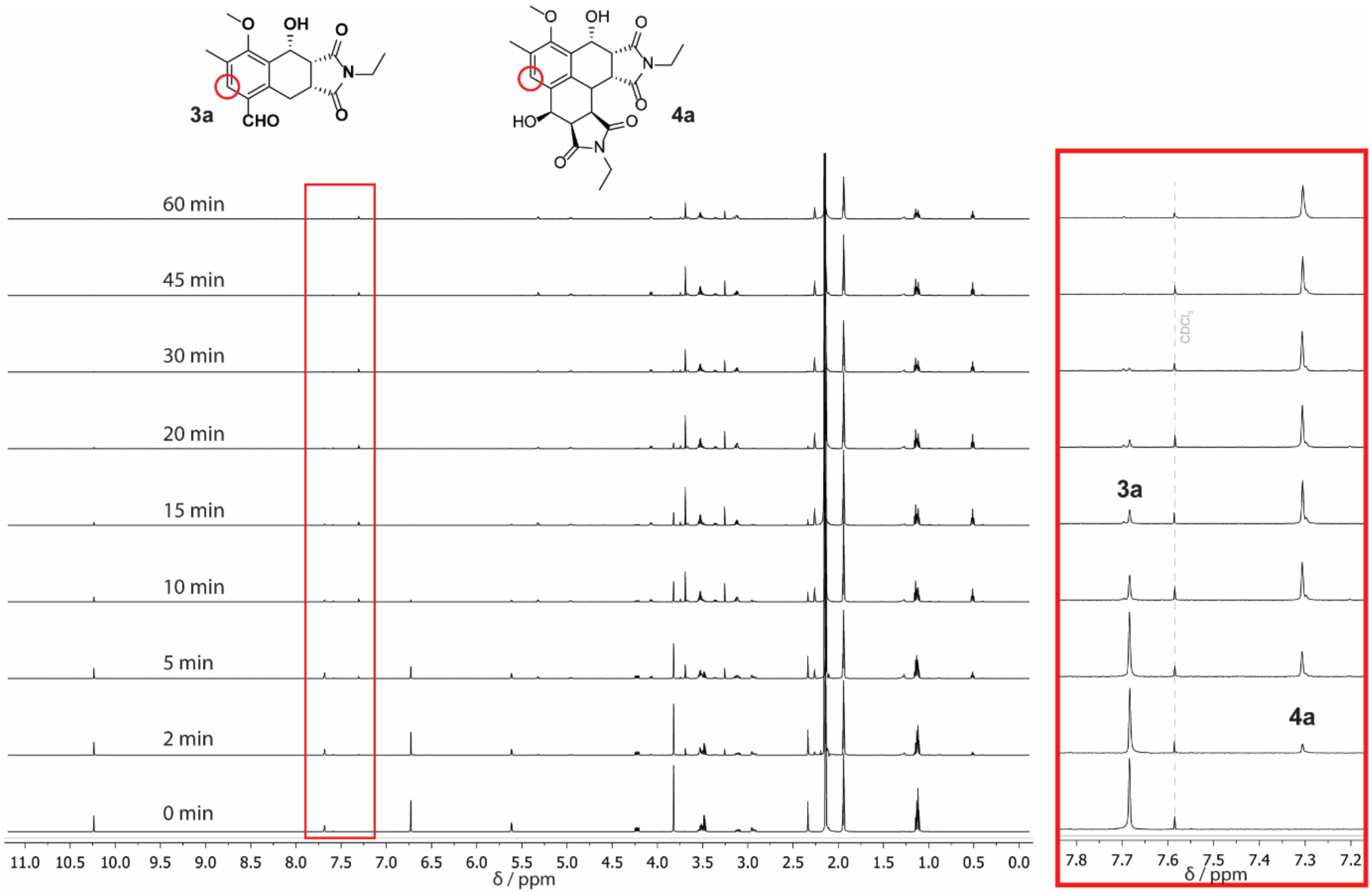

Figure S88 ^{1}H-NMR spectra of reaction solutions irradiated with for various times to determine the quantum yield of the reaction of **3a** and **2a** to form **4a** in CD_3CN. Conversions were determined using the circled proton signals in the highlighted region (7.2-7.8 ppm).

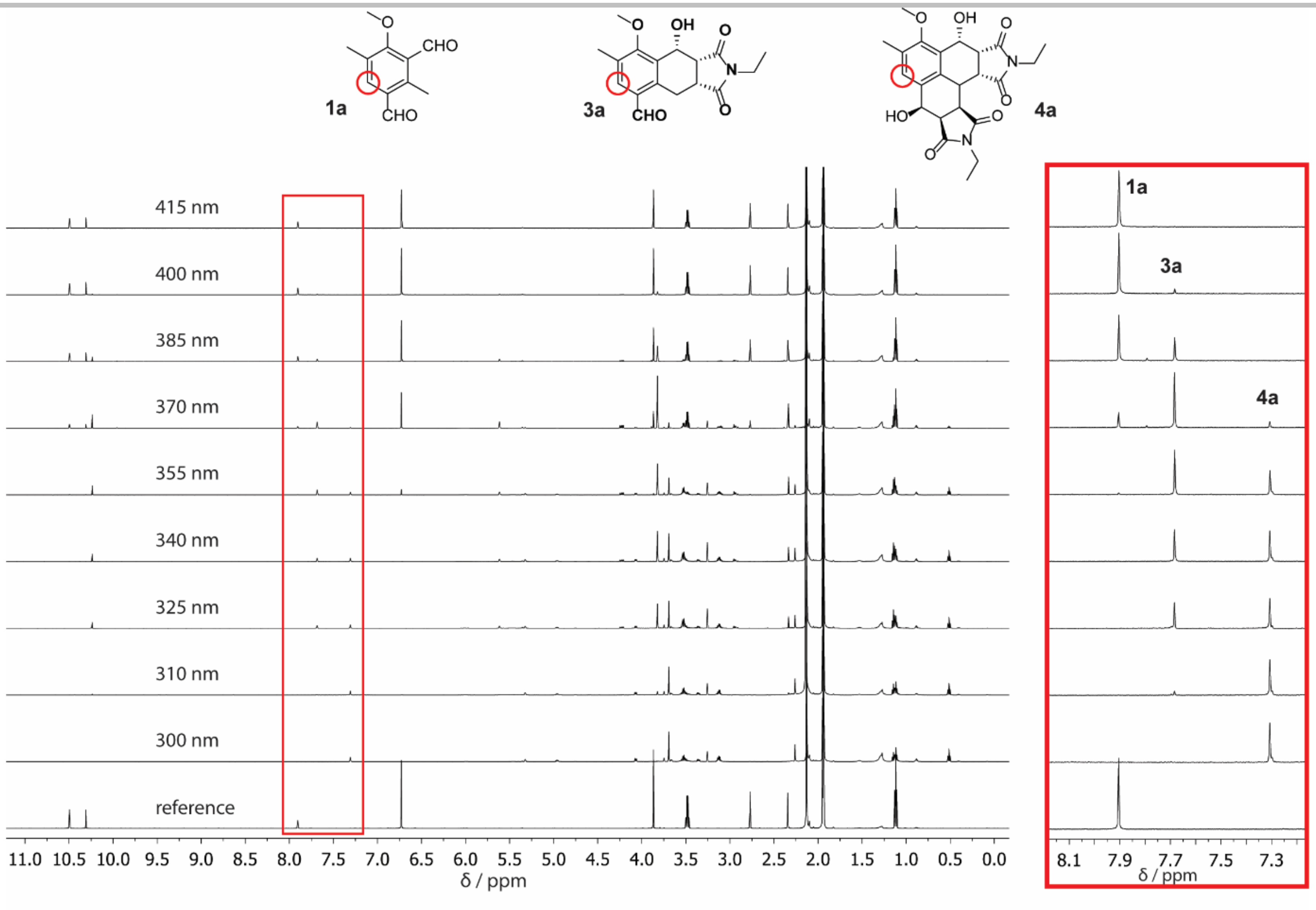

Figure S89 ^{1}H-NMR spectra of reaction solutions irradiated with various wavelengths to determine the wavelength dependent conversion of **1a** and **2a** to form **3a** and **4a** in CD_3CN. Conversions were determined using the circled proton signals in the highlighted region (7.2-8.1 ppm).

XIV. LC-HRMS Traces, Spectra and Fragmentation Patterns

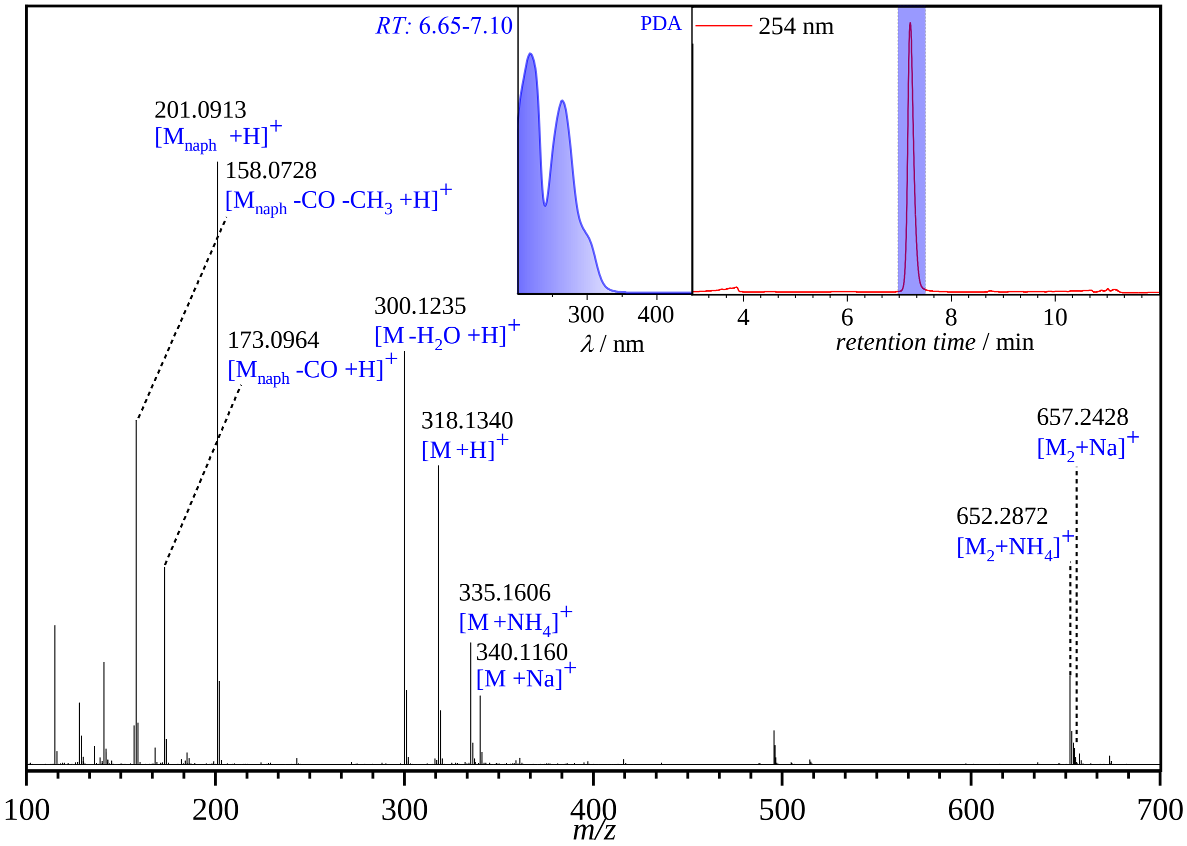

Figure S90 LC-trace (254 nm detector wavelength), accumulated mass-spectra and accumulated PDA spectra of (3a*R*,9*R*,9a*R*)-2-ethyl-9-hydroxy-8-methoxy-7-methyl-1,3-dioxo-2,3,3a,4,9,9a-hexahydro-1*H*-benzo[*f*]isoindole-5-carbaldehyde (**3a**).

Table S3 Collation of observed signals in the mass spectrum **Figure S90** and comparison with theoretically expected *m/z* values for the assigned signals. The abbreviations and fragmentation pattern are defined in **Scheme S6.**

Symbol	m/z^{exp}	m/z^{theor}	Δ_{ppm}	composition
[M$_2$+Na]$^+$	657.2428	657.2419	1.37	$C_{34}H_{38}N_2NaO_{10}^+$
[M$_2$+NH$_4$]$^+$	652.2872	652.2865	1.07	$C_{34}H_{42}N_3O_{10}^+$
[M +Na]$^+$	340.1160	340.1155	1.47	$C_{17}H_{19}NNaO_5^+$
[M +NH$_4$]$^+$	335.1606	335.1601	1.49	$C_{17}H_{23}N_2O_5^+$
[M +H]$^+$	318.134	318.1336	1.26	$C_{17}H_{20}NO_5^+$
[M -H$_2$O +H]$^+$	300.1235	300.123	1.67	$C_{17}H_{18}NO_4^+$
[M$_{naph}$ +H]$^+$	201.0913	201.091	1.49	$C_{13}H_{13}O_2^+$
[M$_{naph}$ -CO +H]$^+$	173.0964	173.0961	1.73	$C_{12}H_{13}O^+$
[M$_{naph}$ -CO -CH$_3$ +H]$^+$	158.0728	158.0726	1.27	$C_{11}H_{10}O^{\bullet+}$

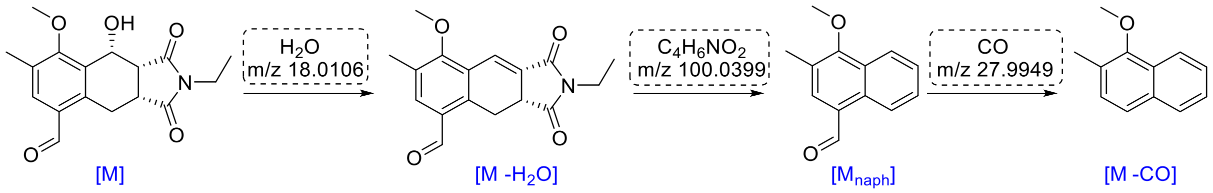

Scheme S6 Fragments identified in the ESI-MS spectrum **Figure S90** and corresponding abbreviations.

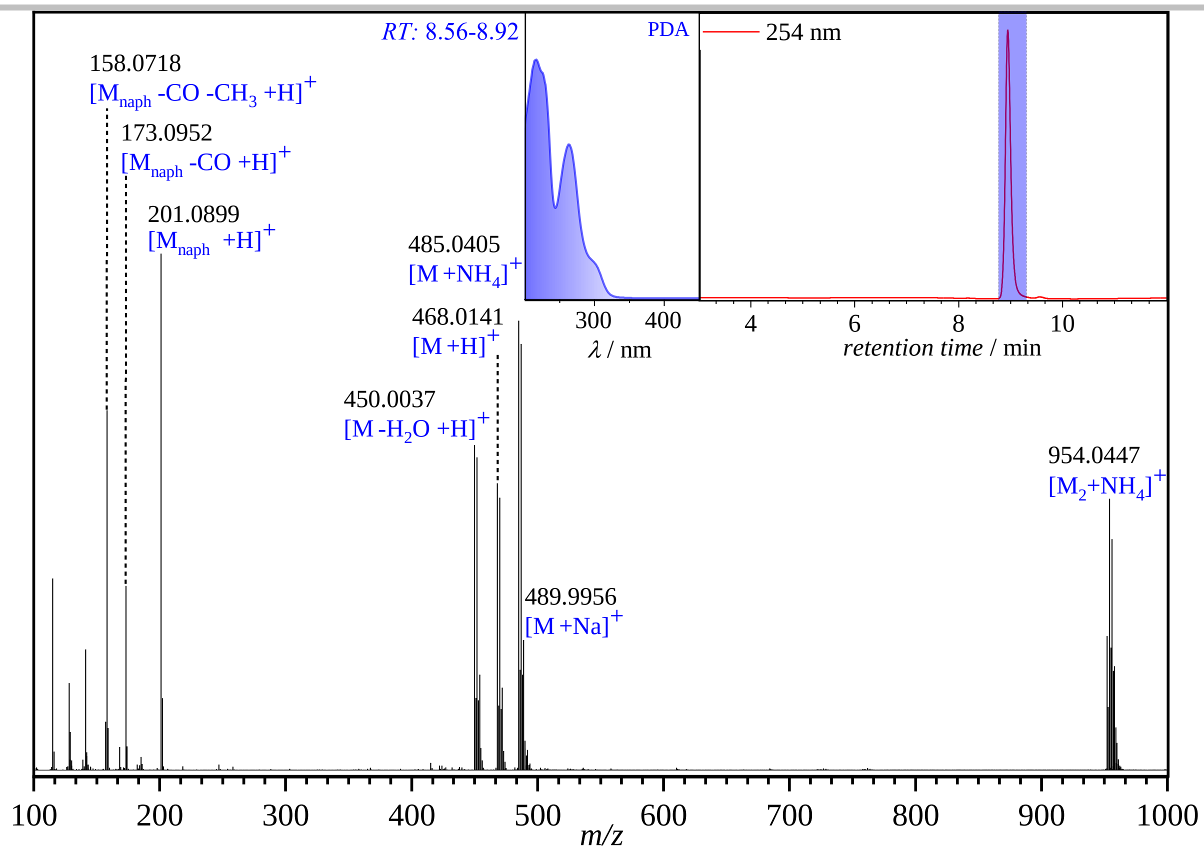

Figure S91 LC-trace (254 nm detector wavelength), accumulated mass-spectra and accumulated PDA spectra of (3a*R*,9*R*,9a*R*)-9-hydroxy-8-methoxy-7-methyl-1,3-dioxo-2-(2,4,6-trichloro-phenyl)-2,3,3a,4,9,9a-hexahydro-1*H*-benzo[*f*]isoindole-5-carbaldehyde (***endo*-3b**).

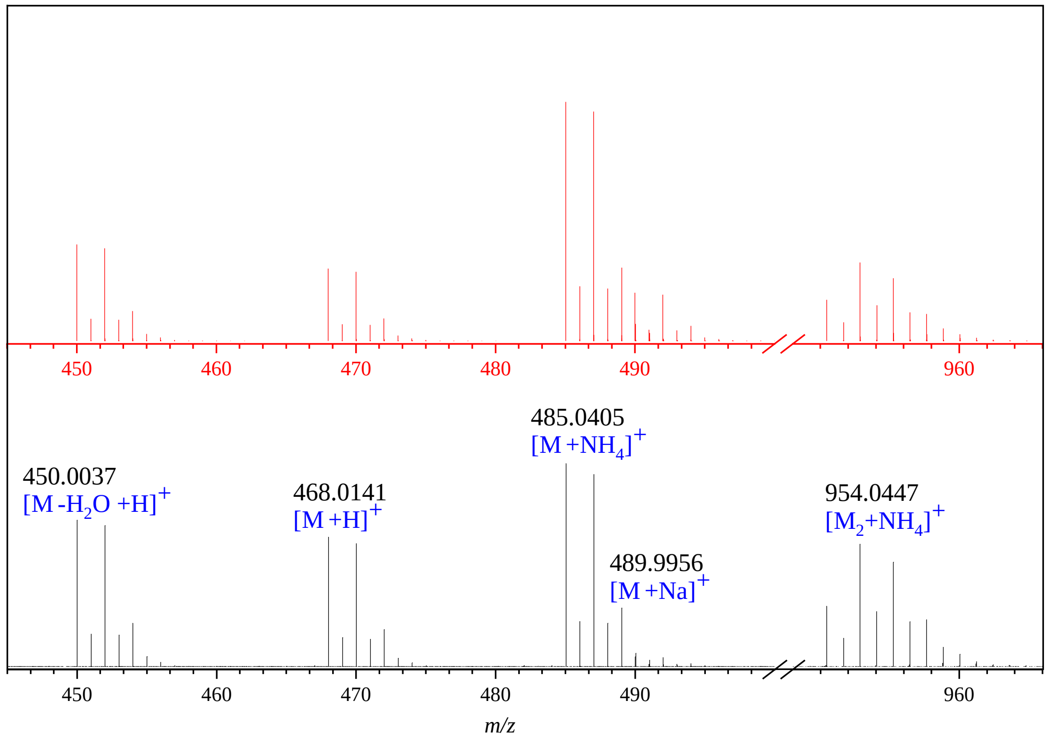

Figure S92 Simulated (red) and experimental spectra of (3a*R*,9*R*,9a*R*)-9-hydroxy-8-methoxy-7-methyl-1,3-dioxo-2-(2,4,6-trichloro-phenyl)-2,3,3a,4,9,9a-hexahydro-1*H*-benzo[*f*]isoindole-5-carbaldehyde (***endo*-3b**) (black) showing the isotopic patters of the major signals in the mass spectrum (refer to **Figure S91**).

Table S4 Collation of observed signals in the mass spectrum **Figure S91** and comparison with theoretically expected *m/z* values for the assigned signals. The abbreviations and fragmentation pattern are similar to **3a** (refer to **Scheme S6**).

Symbol	m/z^{exp}	m/z^{theor}	Δ_{ppm}	composition
$[M_2+NH_4]^+$	954.0447	954.0497	5.24	$C_{42}H_{36}Cl_6N_3O_{10}^+$
$[M+Na]^+$	489.9956	489.9986	6.12	$C_{21}H_{16}Cl_3NNaO_5^+$
$[M+NH_4]^+$	485.0405	485.0432	5.57	$C_{21}H_{20}Cl_3N_2O_5^+$
$[M+H]^+$	468.0141	468.0167	5.56	$C_{21}H_{17}Cl_3NO_5^+$
$[M-H_2O+H]^+$	450.0037	450.0061	5.33	$C_{21}H_{15}Cl_3NO_4^+$
$[M_{naph}+H]^+$	201.0899	201.091	5.47	$C_{13}H_{13}O2^+$
$[M_{naph}-CO+H]^+$	173.0952	173.0961	5.20	$C_{12}H_{13}O^+$
$[M_{naph}-CO-CH_3+H]^+$	158.0718	158.0726	5.06	$C_{11}H_{10}O^{\bullet+}$

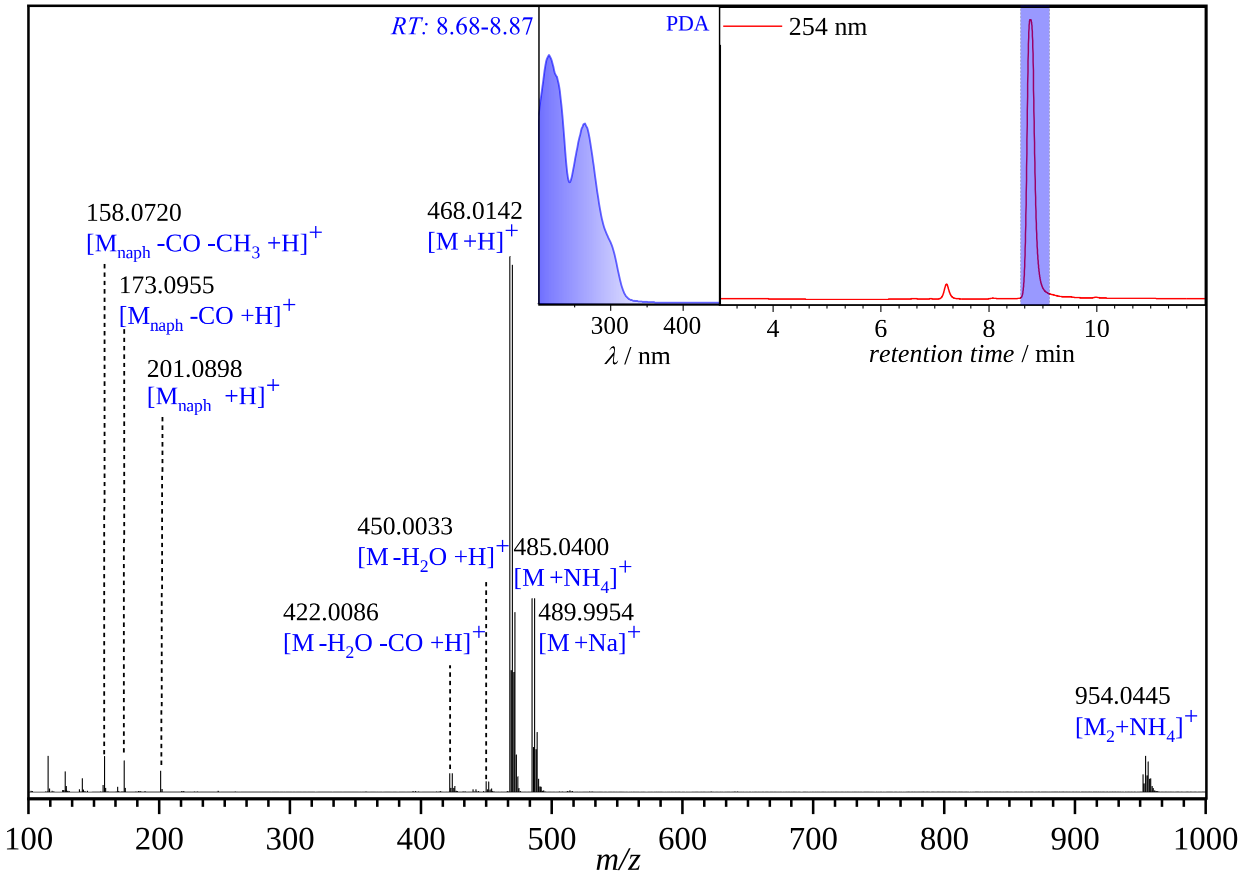

Figure S93 LC-trace (254 nm detector wavelength), accumulated mass-spectra and accumulated PDA spectra of (3a*R*,9*S*,9a*R*)-9-hydroxy-8-methoxy-7-methyl-1,3-dioxo-2-(2,4,6-trichloro-phenyl)-2,3,3a,4,9,9a-hexahydro-1*H*-benzo[*f*]isoindole-5-carbaldehyde (***exo*-3b**).

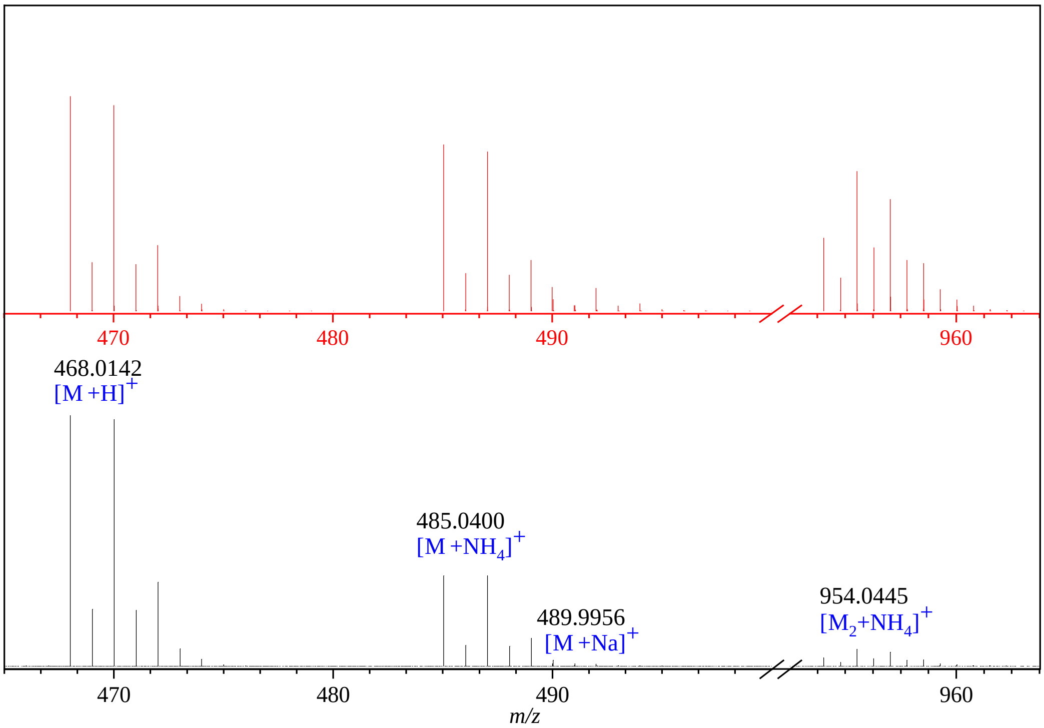

Figure S94 Simulated (red) and experimental spectra of (3a*R*,9*S*,9a*R*)-9-hydroxy-8-methoxy-7-methyl-1,3-dioxo-2-(2,4,6-trichlorophenyl)-2,3,3a,4,9,9a-hexahydro-1*H*-benzo[*f*]isoindole-5-carbaldehyde (**exo-3b**). (black) showing the isotopic patters of the major signals in the mass spectrum (refer to **Figure S93**).

Table S5 Collation of observed signals in the mass spectrum **Figure S93** and comparison with theoretically expected *m/z* values for the assigned signals. The abbreviations and fragmentation pattern are similar to **3a** (refer to **Scheme S6**).

Symbol	m/z^{exp}	m/z^{theor}	Δ_{ppm}	composition
$[M_2+NH_4]^+$	954.0445	954.0497	5.45	$C_{42}H_{36}Cl_6N_3O_{10}^+$
$[M+Na]^+$	489.9954	489.9986	6.53	$C_{21}H_{16}Cl_3NNaO_5^+$
$[M+NH_4]^+$	485.04	485.0432	6.60	$C_{21}H_{20}Cl_3N_2O_5^+$
$[M+H]^+$	468.0142	468.0167	5.34	$C_{21}H_{17}Cl_3NO_5^+$
$[M-H_2O+H]^+$	450.0033	450.0061	6.22	$C_{21}H_{15}Cl_3NO_4^+$
$[M-H_2O-CO+H]^+$	422.0086	422.0112	6.16	$C_{20}H_{15}Cl_3NO_3$+
$[M_{naph}+H]^+$	201.0898	201.091	5.97	$C_{13}H_{13}$O2$^+$
$[M_{naph}-CO+H]^+$	173.095	173.0961	6.35	$C_{12}H_{13}O^+$
$[M_{naph}-CO-CH_3+H]^+$	158.072	158.0726	3.80	$C_{11}H_{10}O^{\bullet+}$

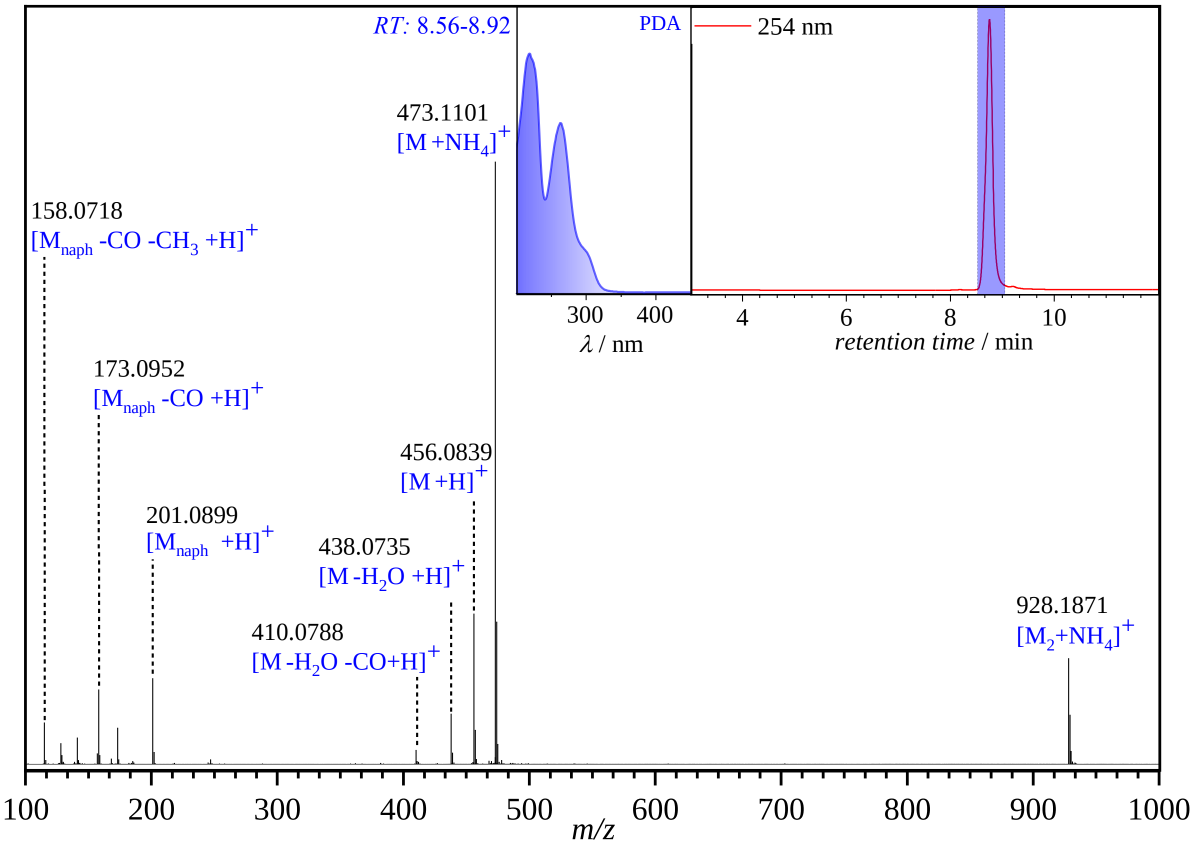

Figure S95 LC-trace (254 nm detector wavelength), accumulated mass-spectra and accumulated PDA spectra of (3a*R*,9*R*,9a*R*)-9-hydroxy-8-methoxy-7-methyl-1,3-dioxo-2-(perfluorophenyl)-2,3,3a,4,9,9a-hexahydro-1*H*-benzo[*f*]isoindole-5-carbaldehyde (**3c**).

Table S6 Collation of observed signals in the mass spectrum **Figure S95** and comparison with theoretically expected *m/z* values for the assigned signals. The abbreviations and fragmentation pattern are defined in **Scheme S7**.

Symbol	m/z^{exp}	m/z^{theor}	Δ_{ppm}	composition
$[M_2+NH_4]^+$	928.1871	928.1923	5.60	$C_{42}H_{32}F_{10}N_3O_{10}^+$
$[M+NH_4]^+$	473.1101	473.1130	6.13	$C_{21}H_{18}F_5N_2O_5^+$
$[M+H]^+$	456.0839	456.0865	5.70	$C_{21}H_{15}F_5NO_5^+$
$[M-H_2O+H]^+$	438.0735	438.0759	5.48	$C_{21}H_{13}F_5NO_4^+$
$[M-H_2O-CO+H]^+$	410.0788	410.0810	5.36	$C_{20}H_{13}F_5NO_3^+$
$[M_{naph}+H]+$	201.0899	201.091	5.47	$C_{13}H_{13}O_2^+$
$[M_{naph}-CO+H]^+$	173.0952	173.0961	5.20	$C_{12}H_{13}O^+$
$[M_{naph}-CO-CH_3+H]^+$	158.0718	158.0726	5.06	$C_{11}H_{10}O^{\bullet+}$

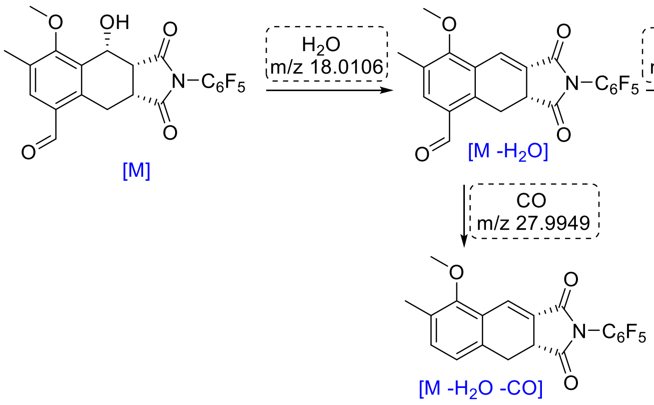

Scheme S7 Fragments identified in the ESI-MS spectrum **Figure S95** and corresponding abbreviations.

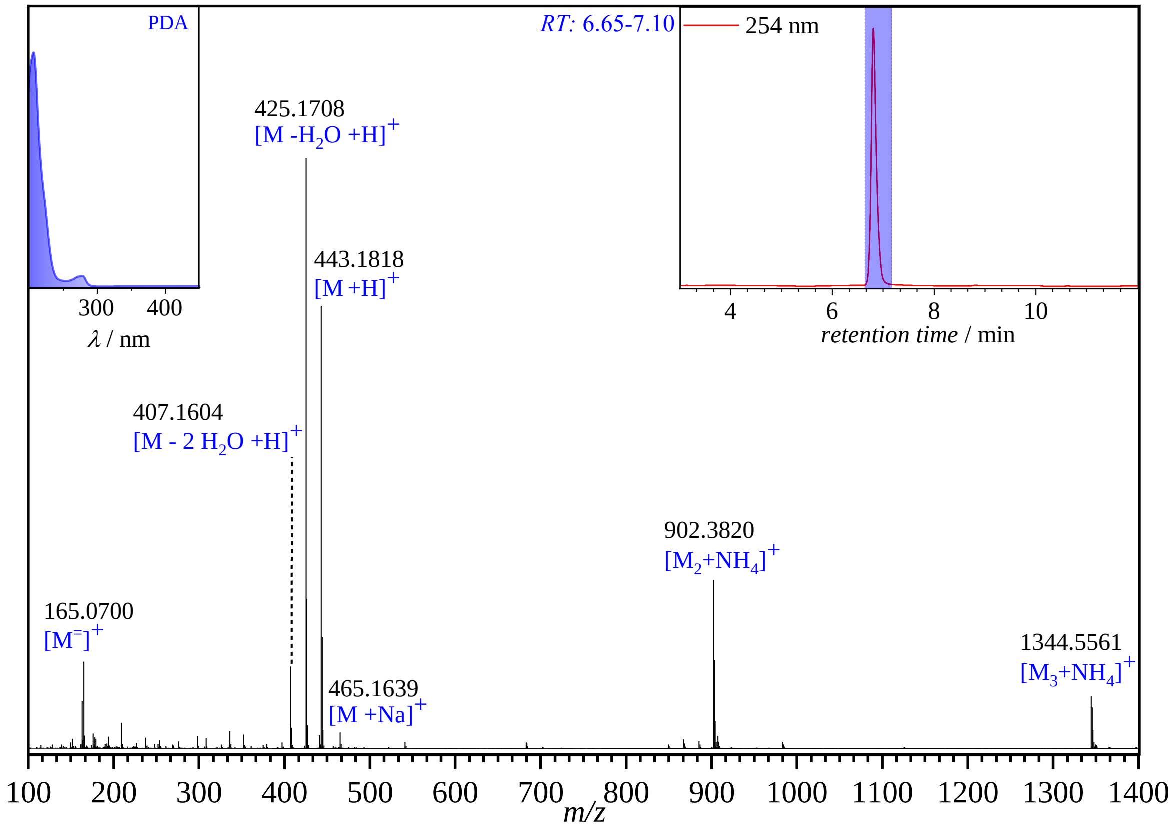

Figure S96 LC-trace (254 nm detector wavelength), accumulated mass-spectra and accumulated PDA spectra of 3a*R*,4*R*,8*R*,8a*R*,11a*R*,11b*R*,11c*R*)-2,10-diethyl-4,8-dihydroxy-5-methoxy-6-methyl-3a,8,8a,11a,11b,11c-hexahydro-1*H*-pyrrolo[3',4':2,3]naphtho[1,8-*ef*]isoindole-1,3,9,11(2*H*,4*H*,10*H*)-tetraone (**4a**).

Table S7 Collation of observed signals in the mass spectrum **Figure S96** and comparison with theoretically expected *m/z* values for the assigned signals. The abbreviations and fragmentation pattern are defined in **Scheme S8**.

Symbol	m/z^{exp}	m/z^{theor}	Δ_{ppm}	composition
$[M_3+NH_4]^+$	1344.5561	1344.5558	0.22	$C_{69}H_{82}N_7O_{21}^+$
$[M_2+NH_4]^+$	902.3820	902.3818	0.22	$C_{46}H_{56}N_5O_{14}^+$
$[M +Na]^+$	465.1639	465.1632	1.50	$C_{23}H_{26}N_2NaO_7^+$
$[M +H]^+$	443.1818	443.1813	1.13	$C_{23}H_{27}N_2O_7^+$
[M -H_2O +H]+	425.1708	425.1707	0.24	$C_{23}H_{25}N_2O_6^+$
[M - 2 H_2O +H]$^+$	407.1604	407.1601	0.74	$C_{23}H_{23}N_2O_5^+$

$[M^{=}]^+$	165.0700	165.0699	0.61	$C_{13}H_9^+$

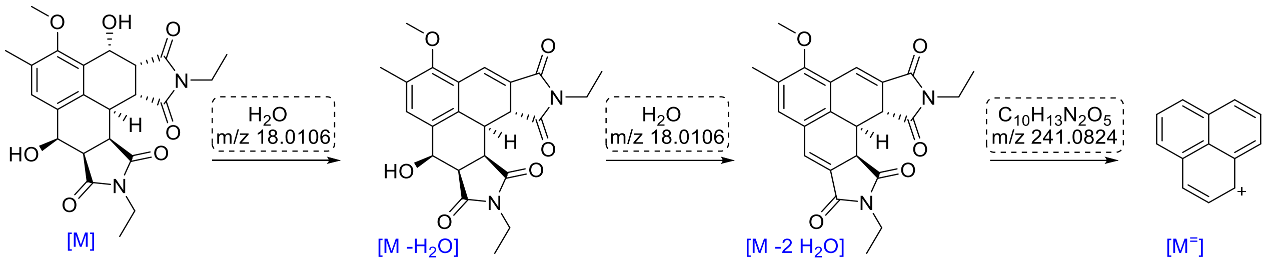

Scheme S8 Fragments identified in the ESI-MS spectrum **Figure S96** and corresponding abbreviations.

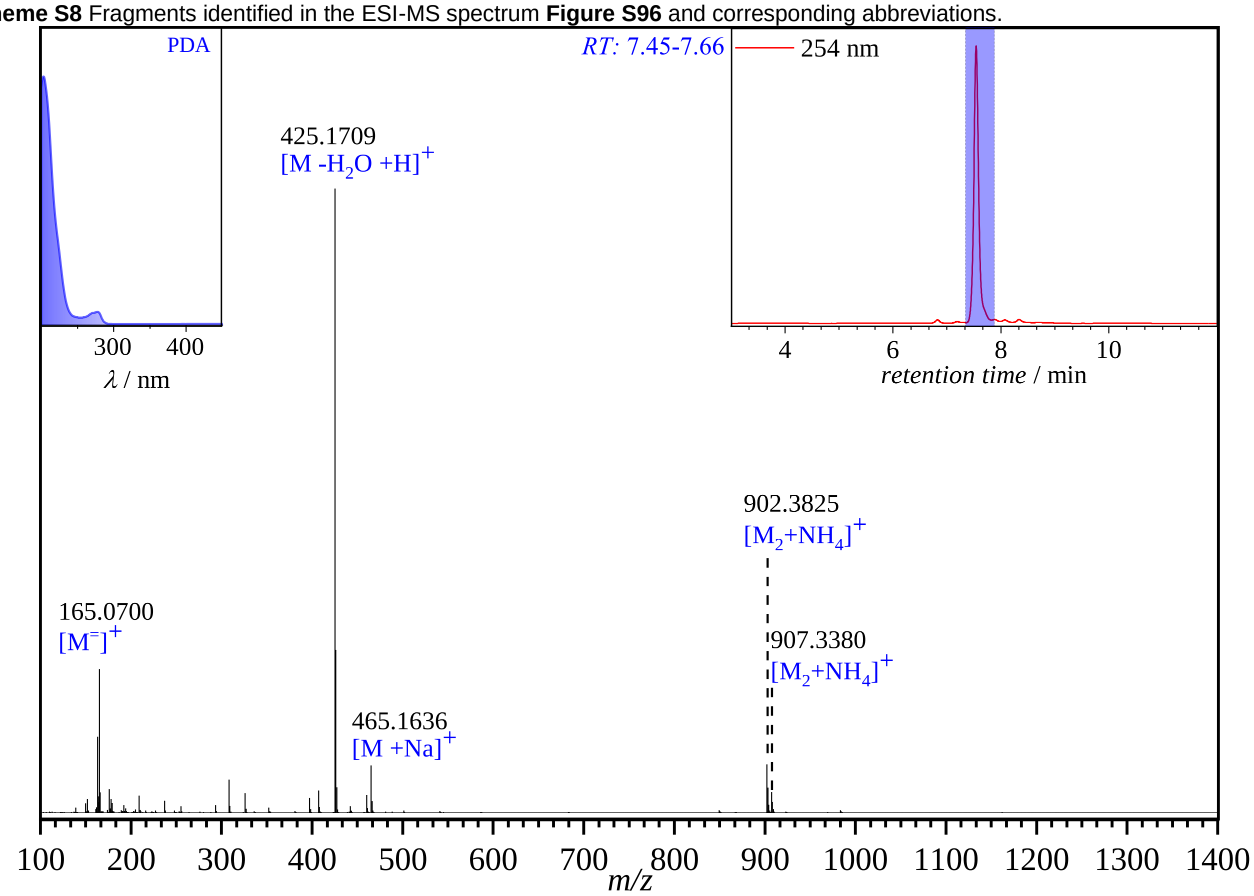

Figure S97 LC-trace (254 nm detector wavelength), accumulated mass-spectra and accumulated PDA spectra of (3a*R*,4*R*,8S,8a*R*,11a*R*,11b*S*,11c*R*)-2,10-diethyl-4,8-dihydroxy-5-methoxy-6-methyl-3a,8,8a,11a,11b,11c-hexahydro-1*H*-pyrrolo[3',4':2,3]naphtho[1,8-*ef*]isoindole-1,3,9,11(2*H*,4*H*,10*H*)-tetraone (***exo*-4a**).

Table S8 Collation of observed signals in the mass spectrum **Figure S97** and comparison with theoretically expected *m/z* values for the assigned signals. The abbreviations and fragmentation pattern are similar to **4a** (refer to **Scheme S8**).

Symbol	m/z^{exp}	m/z^{theor}	Δ_{ppm}	composition
$[M_2+Na]$	907.338	907.3372	0.88	$C_{46}H_{52}N_4NaO_{14}^+$
$[M_2+NH_4]^+$	902.3820	902.3818	0.22	$C_{46}H_{56}N_5O_{14}^+$
$[M+Na]^+$	465.1636	465.1632	0.86	$C_{23}H_{26}N_2NaO_7^+$
$[M-H_2O+H]^+$	425.1709	425.1707	0.47	$C_{23}H_{25}N_2O_6^+$
$[M^{=}+H]^+$	165.0700	165.0699	0.61	$C_{13}H_9^+$

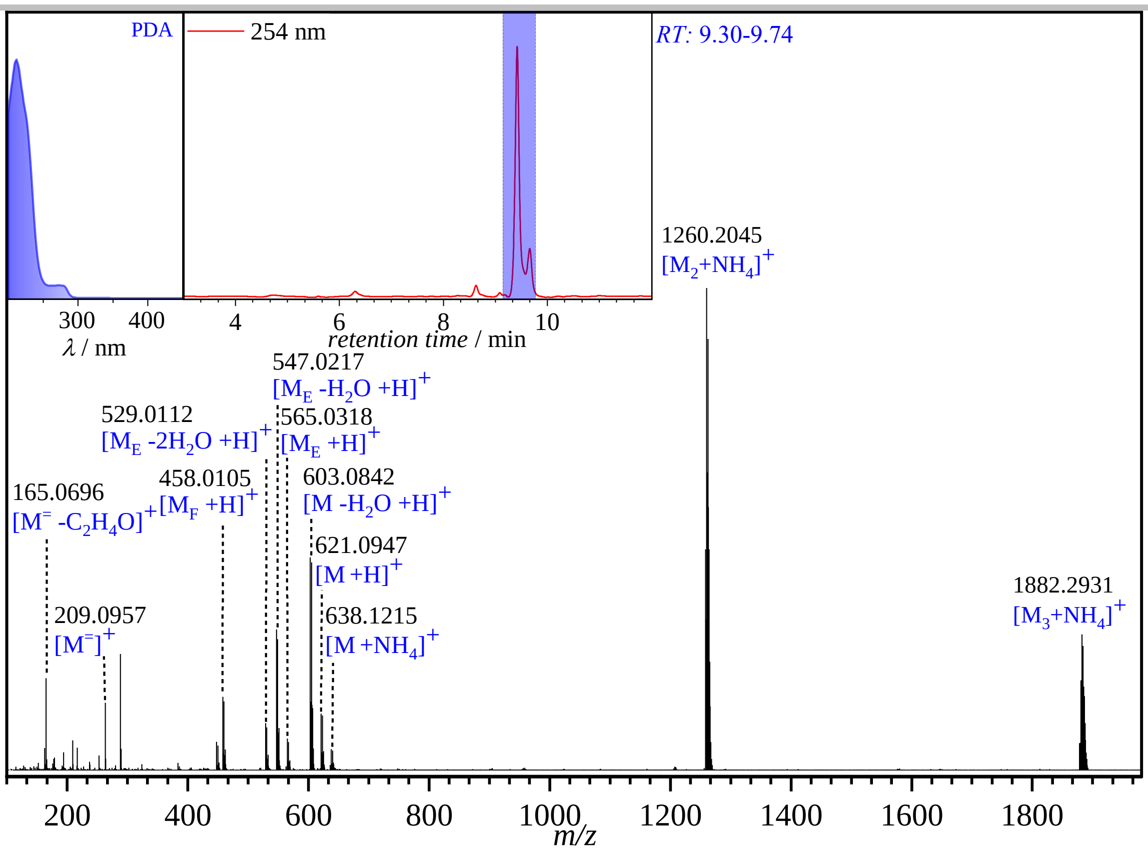

Figure S98 LC-trace (254 nm detector wavelength), accumulated mass-spectra and accumulated PDA spectra of (3a*R*,4*R*,8*R*,8a*R*,11a*R*,11b*R*,11c*R*)-10-(tert-butyl)-4,8-dihydroxy-5-methoxy-6-methyl-2-(2,4,6-trichlorophenyl)-3a,8,8a,11a,11b,11c-hexahydro-1*H*-pyrrolo[3',4':2,3]naphtho-[1,8-*ef*]isoindole-1,3,9,11(2*H*,4*H*,10*H*)-tetraone (***endo*-4b**).

Table S9 Collation of observed signals in the mass spectrum **Figure S98** and comparison with theoretically expected *m/z* values for the assigned signals. The abbreviations and fragmentation pattern are defined in **Scheme S9**.

Symbol	m/z^{exp}	m/z^{theor}	Δ_{ppm}	composition
$[M_3+NH_4]^+$	1882.2915	1882.2931	0.85	$C_{87}H_{85}Cl_9N_7O_{21}^+$
$[M_2+NH_4]^+$	1260.2045	1260.2076	2.46	$C_{58}H_{58}Cl_6N_5O_{14}^+$
$[M+NH_4]^+$	638.1215	638.1222	1.10	$C_{29}H_{31}Cl_3N_3O_7^+$
$[M +H]^+$	621.0947	621.0957	1.61	$C_{29}H_{28}Cl_3N_2O_7^+$
$[M -H_2O +H]^+$	603.0842	603.0851	1.49	$C_{29}H_{26}Cl_3N_2O_6^+$
$[M_E +H]^+$	565.0318	565.0331	2.30	$C_{25}H_{20}Cl_3N_2O_7^+$
$[M_E -H_2O +H]^+$	547.0217	547.0225	1.46	$C_{25}H_{18}Cl_3N_2O_6^+$
$[M_E -2H_2O +H]^+$	529.0112	529.0119	1.32	$C_{25}H_{16}Cl_3N_2O_5^+$
$[M_F +H]^+$	458.0105	458.0112	1.53	$C_{23}H_{15}Cl_3NO_3^+$
$[M^=]^+$	209.0957	209.0961	1.91	$C_{15}H_{13}O^+$
$[M^= -C_2H_4O]^+$	165.0696	165.0699	1.82	$C_{13}H_9^+$

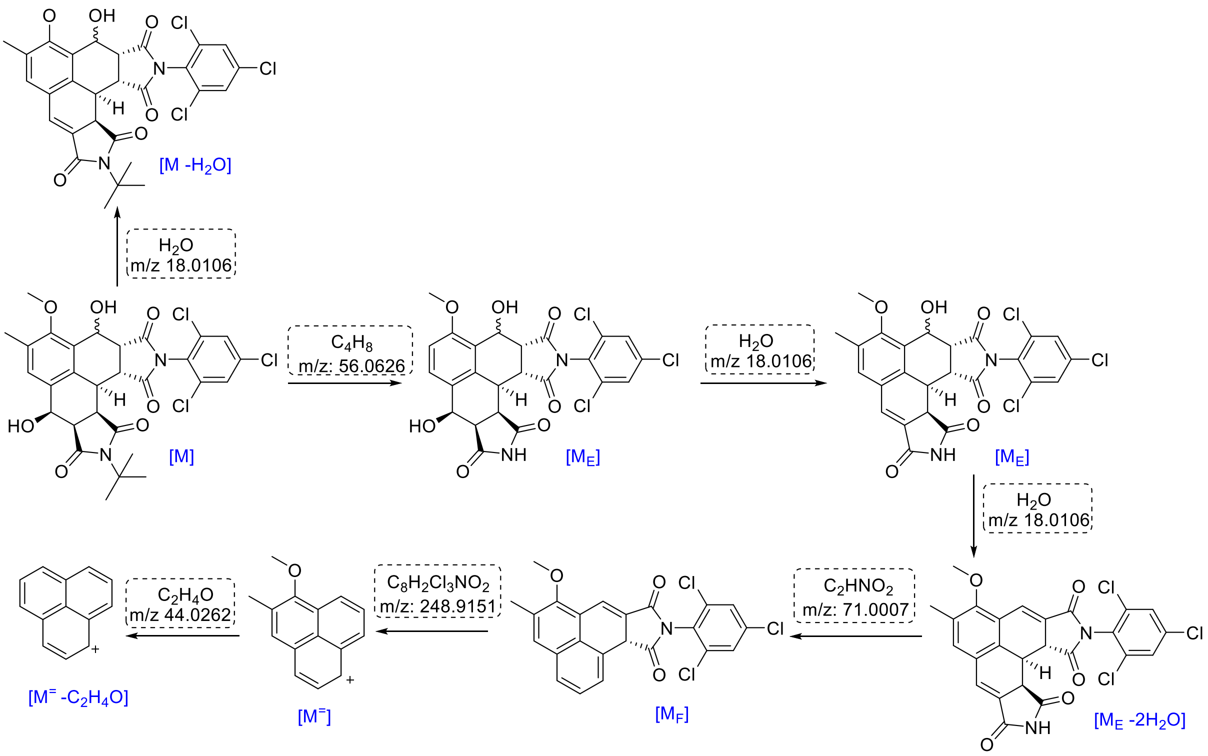

Scheme S9 Fragments identified in the ESI-MS spectrum **Figure S98** and corresponding abbreviations.

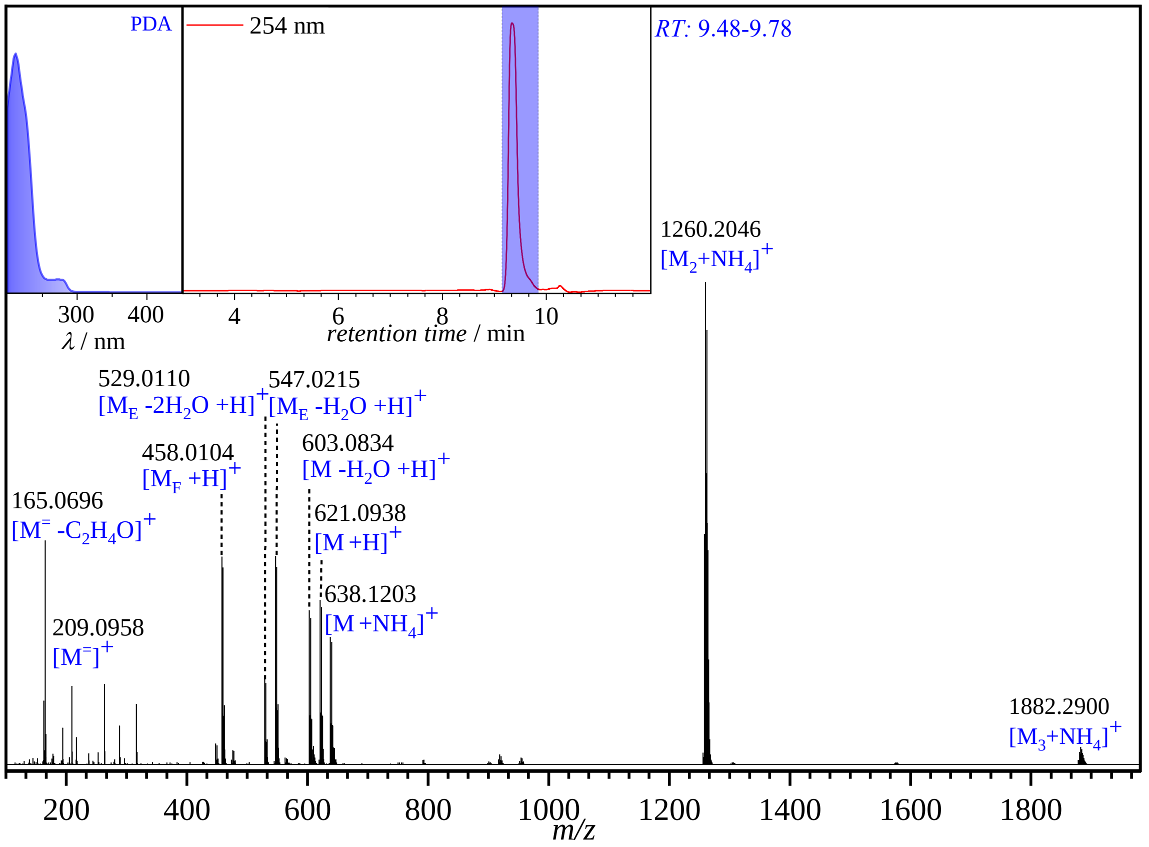

Figure S99 LC-trace (254 nm detector wavelength), accumulated mass-spectra and accumulated PDA spectra of (3a*R*,4*R*,8*S*,8a*R*,11a*R*,11b*R*,11c*R*)-10-(tert-butyl)-4,8-dihydroxy-5-methoxy-6-methyl-2-(2,4,6-trichlorophenyl)-3a,8,8a,11a,11b,11c-hexahydro-1*H*-pyrrolo[3',4':2,3]naphtho-[1,8-*ef*]isoindole-1,3,9,11(2*H*,4*H*,10*H*)-tetraone (***exo*-4b**).

Table S10 Collation of observed signals in the mass spectrum **Figure S99** and comparison with theoretically expected *m/z* values for the assigned signals. The abbreviations and fragmentation pattern are similar to ***endo*-4b** (refer to **Scheme S9**).

Symbol	m/z^{exp}	m/z^{theor}	Δ_{ppm}	composition
$[M_3+NH_4]^+$	1882.2900	1882.2931	1.65	$C_{87}H_{85}Cl_9N_7O_{21}^+$
$[M_2+NH_4]^+$	1260.2046	1260.2076	2.38	$C_{58}H_{58}Cl_6N_5O_{14}^+$
$[M+NH_4]^+$	638.1203	638.1222	2.98	$C_{29}H_{31}Cl_3N_3O_7^+$
$[M +H]^+$	621.0938	621.0957	3.06	$C_{29}H_{28}Cl_3N_2O_7^+$
$[M -H_2O +H]^+$	603.0834	603.0851	2.82	$C_{29}H_{26}Cl_3N_2O_6^+$
$[M_E -H_2O +H]^+$	547.0215	547.0225	1.83	$C_{25}H_{18}Cl_3N_2O_6^+$
$[M_E -2H_2O +H]^+$	529.0110	529.0119	1.70	$C_{25}H_{16}Cl_3N_2O_5^+$
$[M_F +H]^+$	458.0104	458.0112	1.75	$C_{23}H_{15}Cl_3NO_3^+$
$[M^=]^+$	209.0956	209.0961	2.39	$C_{15}H_{13}O^+$
$[M^= -C_2H_4O]^+$	165.0698	165.0699	0.61	$C_{13}H_9^+$

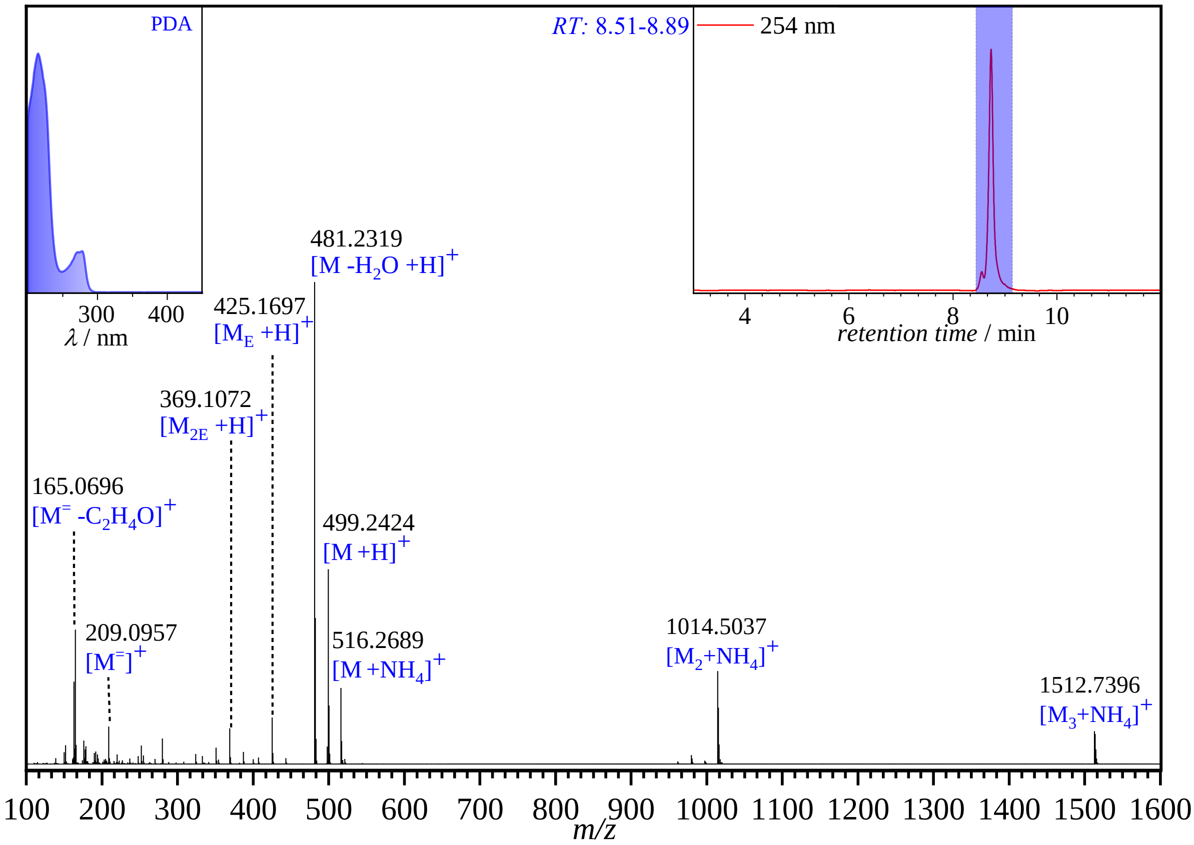

Figure S100 LC-trace (254 nm detector wavelength), accumulated mass-spectra and accumulated PDA spectra of (3a*R*,4*R*,8*R*,8a*R*,11a*R*,11b*R*,11c*R*)-2,10-di-tert-butyl-4,8-dihydroxy-5-methoxy-6-methyl-3a,8,8a,11a,11b,11c-hexahydro-1*H*-pyrrolo[3',4':2,3]naphtho[1,8-*ef*]isoindole-1,3,9,11-(2*H*,4*H*,10*H*)-tetraone (**4c**).

Table S11 Collation of observed signals in the mass spectrum **Figure S100** and comparison with theoretically expected *m/z* values for the assigned signals. The abbreviations and fragmentation pattern are defined in **Scheme S10**.

Symbol	m/z^{exp}	m/z^{theor}	Δ_{ppm}	composition
$[M_3+NH_4]^+$	1512.7396	1512.7436	2.64	$C_{81}H_{106}N_7O_{21}^+$
$[M_2+NH_4]^+$	1014.5037	1014.5070	3.25	$C_{54}H_{72}N_5O_{14}^+$
$[M+NH_4]^+$	516.2689	516.2704	2.91	$C_{27}H_{38}N_3O_7^+$
$[M+H]^+$	499.2424	499.2439	3.00	$C_{27}H_{35}N_2O_7^+$
$[M-H_2O+H]^+$	481.2319	481.2333	2.91	$C_{27}H_{33}N_2O_6^+$
$[M_E+H]^+$	425.1697	425.1707	2.35	$C_{23}H_{25}N_2O_6^+$
$[M_{2E}+H]^+$	369.1072	369.1081	2.44	$C_{19}H_{17}N_2O_6^+$
$[M_=]^+$	209.0957	209.0961	1.91	$C_{15}H_{13}O^+$
$[M_=-C_2H_4O]^+$	165.0696	165.0699	1.82	$C_{13}H_9^+$

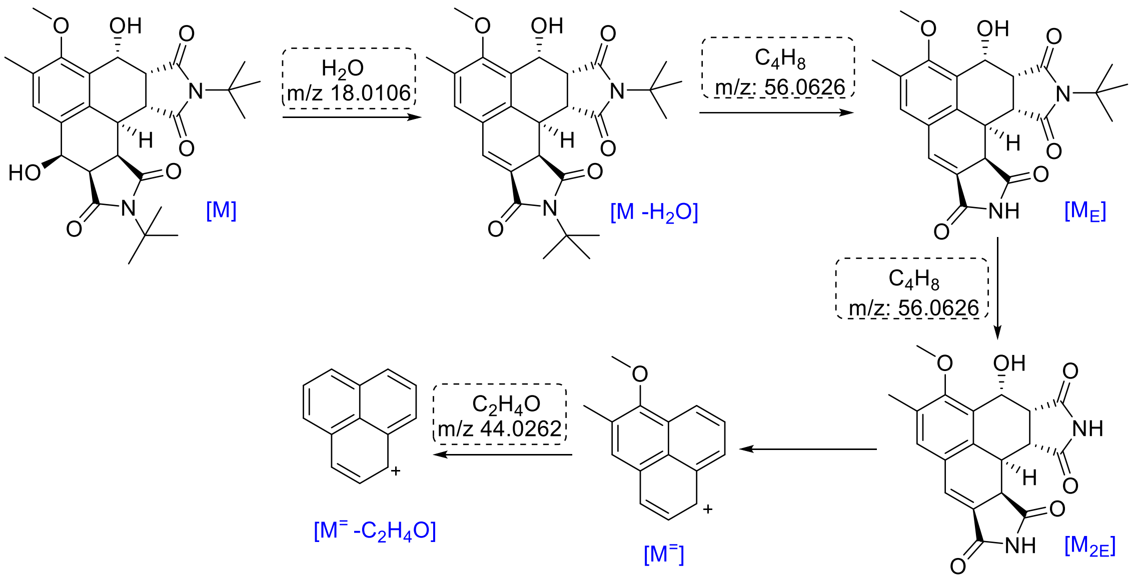

Scheme S10 Fragments identified in the ESI-MS spectrum **Figure S100** and corresponding abbreviations.

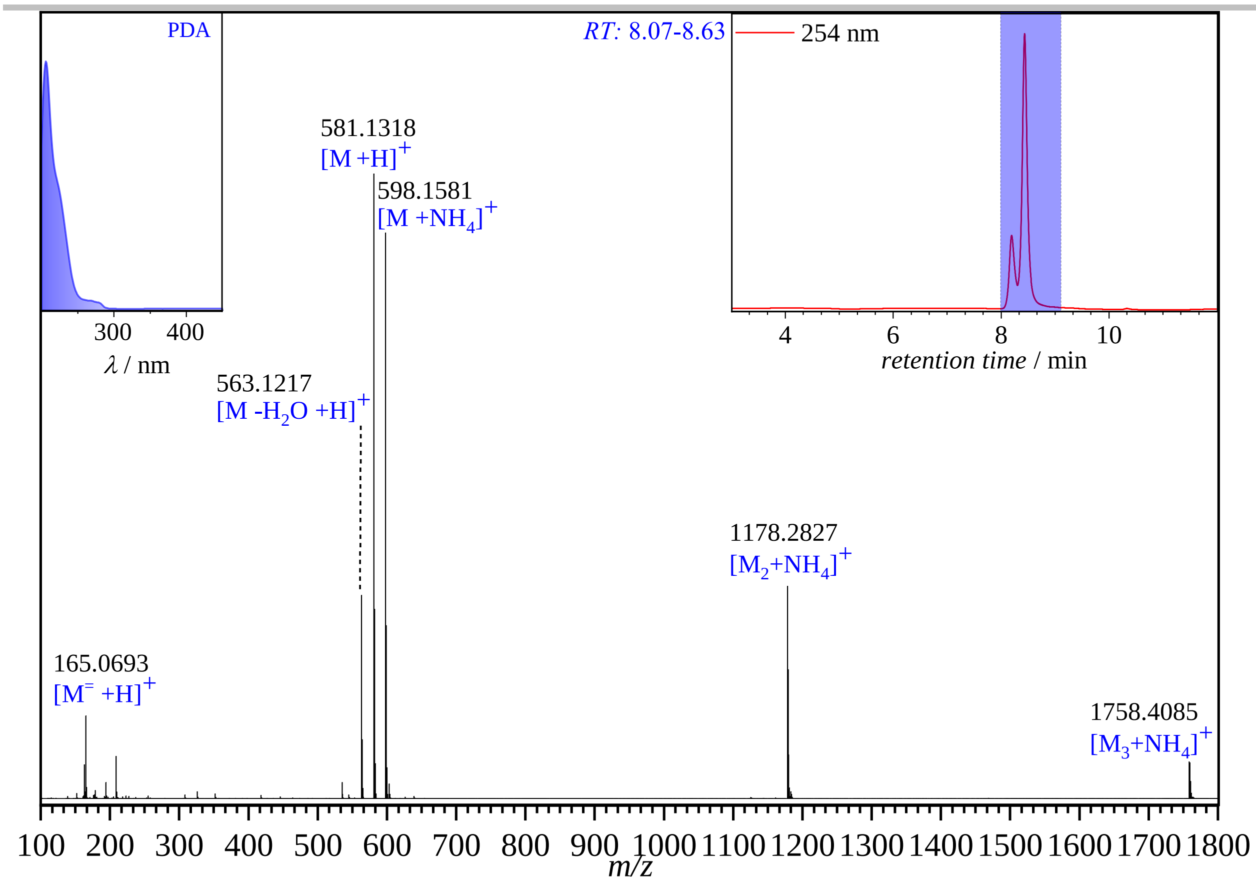

Figure S101 LC-trace (254 nm detector wavelength), accumulated mass-spectra and accumulated PDA spectra of (3a*R*,4*R*,8*R*,8a*R*,11a*R*,11b*S*,11c*R*)-2-ethyl-4,8-dihydroxy-5-methoxy-6-methyl-10-(perfluorophenyl)-3a,8,8a,11a,11b,11c-hexahydro-1*H*-pyrrolo[3',4':2,3]naphtho[1,8-*ef*]isoindole-1,3,9,11(2*H*,4*H*,10*H*)-tetraone (**4d**).

Table S12 Collation of observed signals in the mass spectrum **Figure S101** and comparison with theoretically expected *m/z* values for the assigned signals. The abbreviations and fragmentation pattern are similar to **4a** (refer to **Scheme S9**).

Symbol	m/z^{exp}	m/z^{theor}	Δ_{ppm}	composition
$[M_3+NH_4]^+$	1758.4085	1758.4145	3.41	$C_{81}H_{67}F_{15}N_7O_{21}^+$
$[M_2+NH_4]^+$	1178.2827	1178.2876	4.16	$C_{54}H_{46}F_{10}N_5O_{14}^+$
$[M +NH_4]^+$	598.1581	598.1607	4.35	$C_{27}H_{25}F_5N_3O_7^+$
$[M +H]^+$	581.1318	581.1342	4.13	$C_{27}H_{22}F_5N_2O_7^+$
$[M -H_2O +H]^+$	563.1217	563.1236	3.37	$C_{27}H_{20}F_5N_2O_6^+$
$[M^= +H]^+$	165.0693	165.0699	3.63	$C_{13}H_9^+$

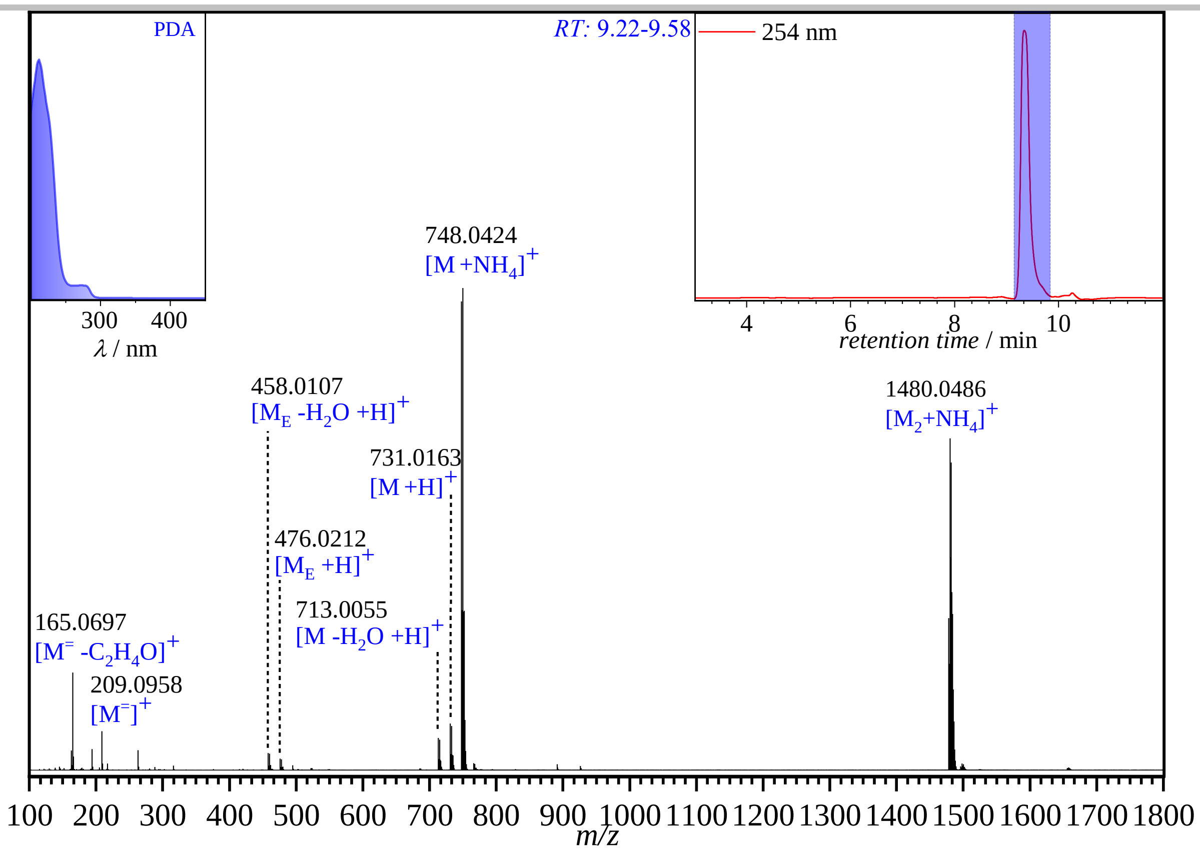

Figure S102 LC-trace (254 nm detector wavelength), accumulated mass-spectra and accumulated PDA spectra of (3a*R*,4*R*,8*R*,8a*R*,11a*R*,11b*S*,11c*R*)-4,8-dihydroxy-5-methoxy-6-methyl-2-(perfluorophenyl)-10-(2,4,6-trichlorophenyl)-3a,8,8a,11a,11b,11c-hexahydro-1H-pyrrolo-[3',4':2,3]naphtho[1,8-*ef*]isoindole-1,3,9,11(2*H*,4*H*,10*H*)-tetraone (***endo*-4e**).

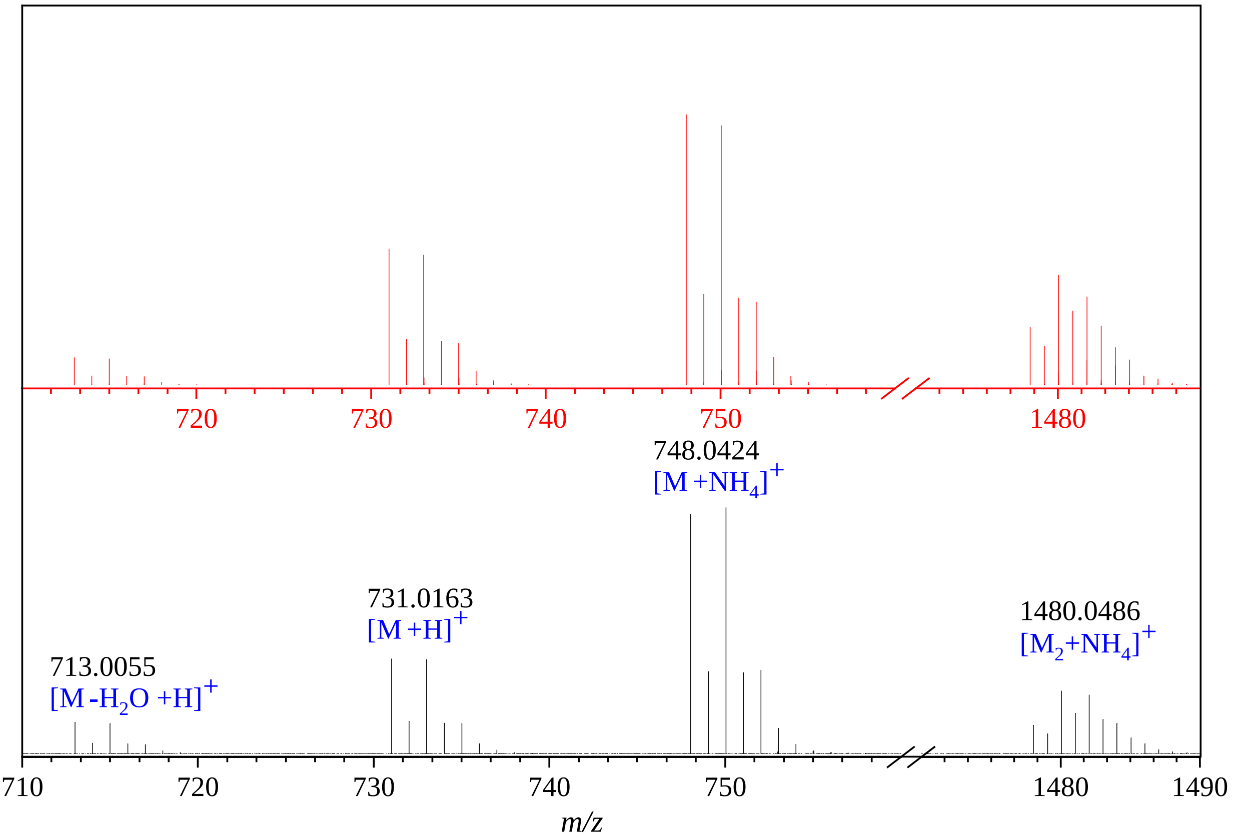

Figure S103 Simulated (red) and experimental spectra of (3a*R*,4*R*,8*R*,8a*R*,11a*R*,11b*S*,11c*R*)-4,8-dihydroxy-5-methoxy-6-methyl-2-(perfluorophenyl)-10-(2,4,6-trichlorophenyl)-3a,8,8a,11a,11b,11c-hexahydro-1*H*-pyrrolo-[3',4':2,3]naphtho[1,8-*ef*]isoindole-1,3,9,11-(2*H*,4*H*,10*H*)-tetraone (***endo*-4e**) (black) showing the isotopic patters of the major signals in the mass spectrum (refer to **Figure S103**).

Table S13 Collation of observed signals in the mass spectrum **Figure S103** and comparison with theoretically expected *m/z* values for the assigned signals. The abbreviations and fragmentation pattern are defined in **Scheme S11**.

Symbol	m/z^{exp}	m/z^{theor}	Δ_{ppm}	composition
$[M_2+NH_4]^+$	1480.0486	1480.0508	1.49	$C_{62}H_{40}Cl_6F_{10}N_5O_{14}^+$
$[M+NH_4]^+$	748.0424	748.0438	1.87	$C_{31}H_{22}Cl_3F_5N_3O_7^+$
$[M+H]^+$	731.0163	731.0173	1.37	$C_{31}H_{19}Cl_3F_5N_2O_7^+$
$[M-H_2O+H]^+$	713.0055	713.0067	1.68	$C_{31}H_{17}Cl_3F_5N_2O_6^+$
$[M_E+H]^+$	476.0212	476.0218	1.26	$C_{23}H_{17}Cl_3NO_4^+$
$[M_E-H_2O+H]^+$	458.0107	458.0112	1.09	$C_{23}H_{15}Cl_3NO_3^+$
$[M^=]^+$	209.0958	209.0961	1.43	$C_{15}H_{13}O^+$
$[M^=-C_2H_4O]^+$	165.0697	165.0699	1.21	$C_{13}H_9^+$

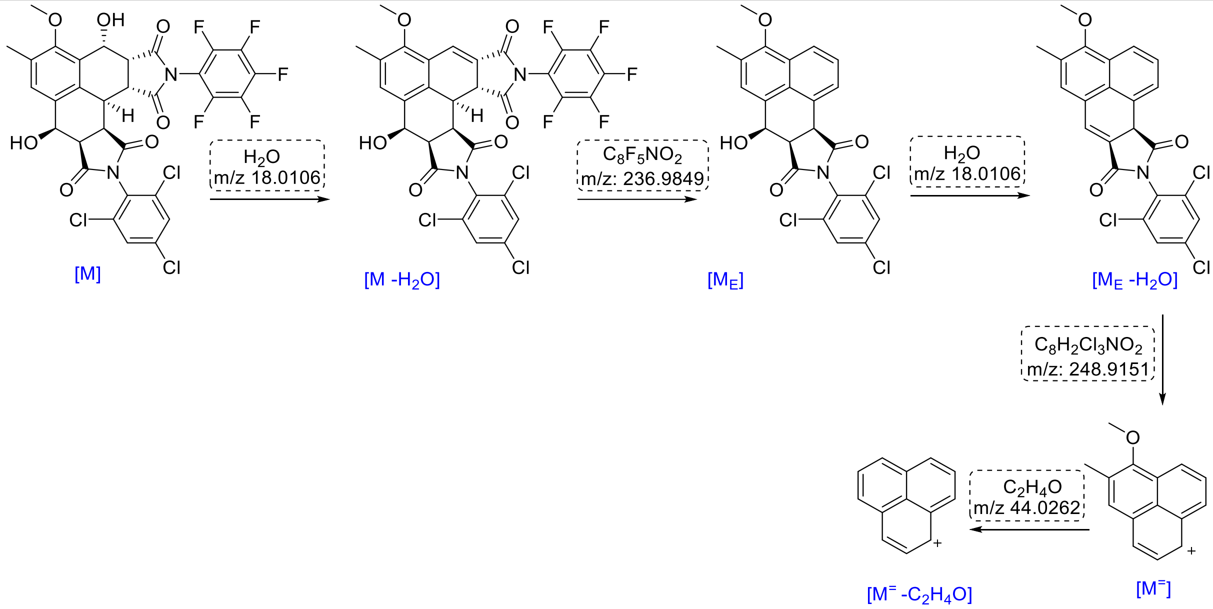

Scheme S11 Fragments identified in the ESI-MS spectrum **Figure S103** and corresponding abbreviations.

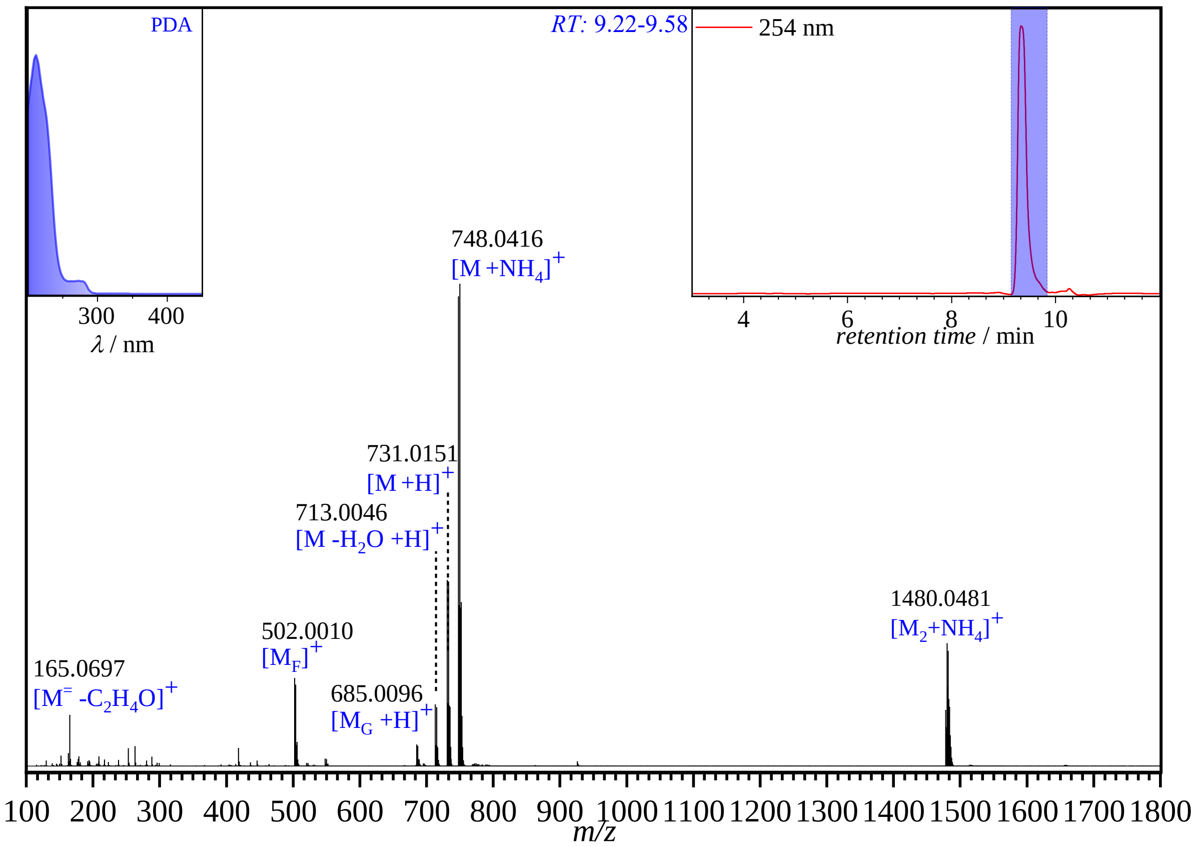

Figure S104 LC-trace (254 nm detector wavelength), accumulated mass-spectra and accumulated PDA spectra of (3a*R*,4*R*,8*R*,8a*R*,11a*R*,11b*R*,11c*R*)-4,8-dihydroxy-5-methoxy-6-methyl-2-(perfluorophenyl)-10-(2,4,6-trichlorophenyl)-3a,8,8a,11a,11b,11c-hexahydro-1*H*-pyrrolo-[3',4':2,3]naphtho[1,8-*ef*]isoindole-1,3,9,11(2*H*,4*H*,10*H*)-tetraone (***exo*-4e**).

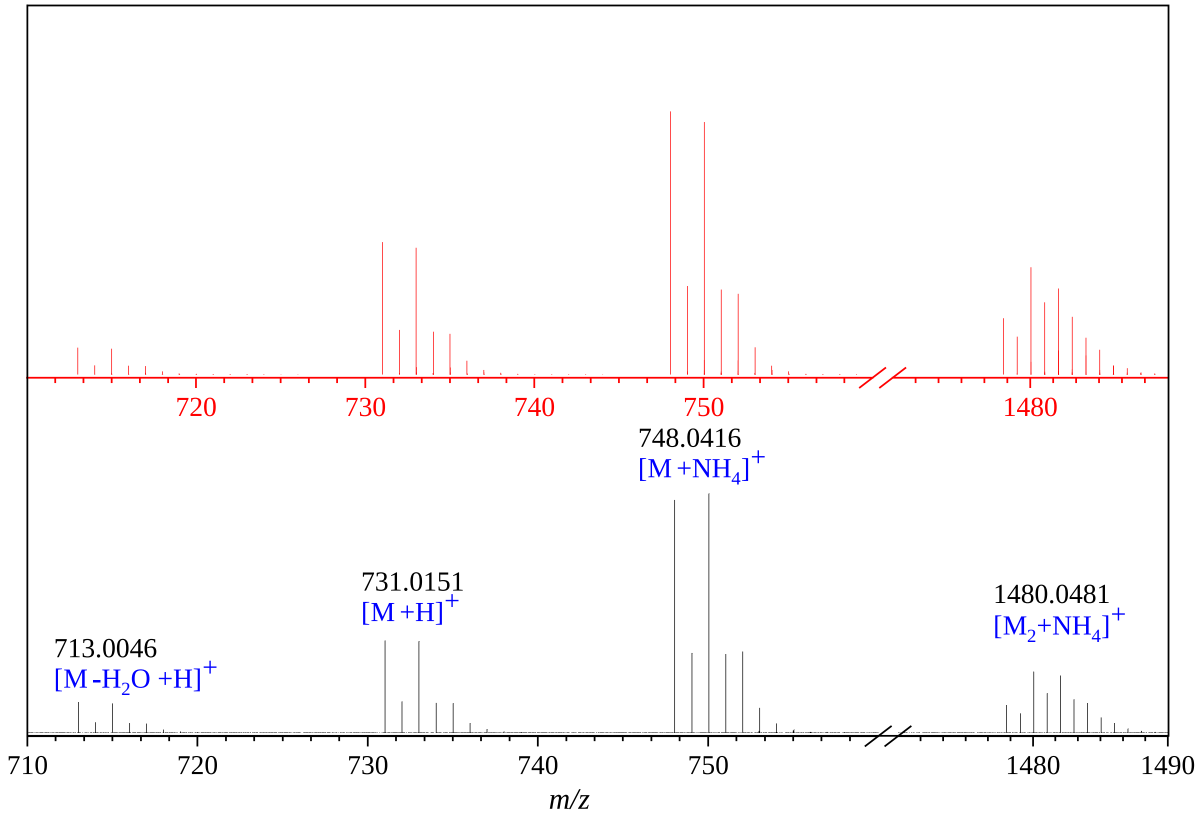

Figure S105 Simulated (red) and experimental spectra of (3a*R*,4*R*,8*R*,8a*R*,11a*R*,11b*R*,11c*R*)-4,8-dihydroxy-5-methoxy-6-methyl-2-(perfluorophenyl)-10-(2,4,6-trichlorophenyl)-3a,8,8a,11a,11b,11c-hexahydro-1*H*-pyrrolo[3',4':2,3]naphtho[1,8-*ef*]isoindole-1,3,9,11(2*H*,4*H*,10*H*)-tetraone (***exo*-4e**) (black) showing the isotopic patters of the major signals in the mass spectrum (refer to **Figure S104**).

Table S14 Collation of observed signals in the mass spectrum **Figure S104** and comparison with theoretically expected *m/z* values for the assigned signals. The abbreviations and fragmentation pattern are defined in **Scheme S12**.

Symbol	m/z^{exp}	m/z^{theor}	Δ_{ppm}	composition
$[M_2+NH_4]^+$	1480.0481	1480.0508	1.82	$C_{62}H_{40}Cl_6F_{10}N_5O_{14}^+$
$[M+NH_4]^+$	748.0416	748.0438	2.94	$C_{31}H_{22}Cl_3F_5N_3O_7^+$
$[M+H]^+$	731.0151	731.0173	3.01	$C_{31}H_{19}Cl_3F_5N_2O_7^+$
$[M-H_2O+H]^+$	713.0046	713.0067	2.95	$C_{31}H_{17}Cl_3F_5N_2O_6^+$
$[M_G+H]^+$	685.0096	685.0118	3.21	$C_{30}H_{17}Cl_3F_5N_2O_5^+$
$[M_F+H]^+$	501.9997	502.001	2.59	$C_{24}H_{15}Cl_3NO_5^+$
$[M^{=}-C_2H_4O]^+$	165.0697	165.0699	1.21	$C_{13}H_9^+$

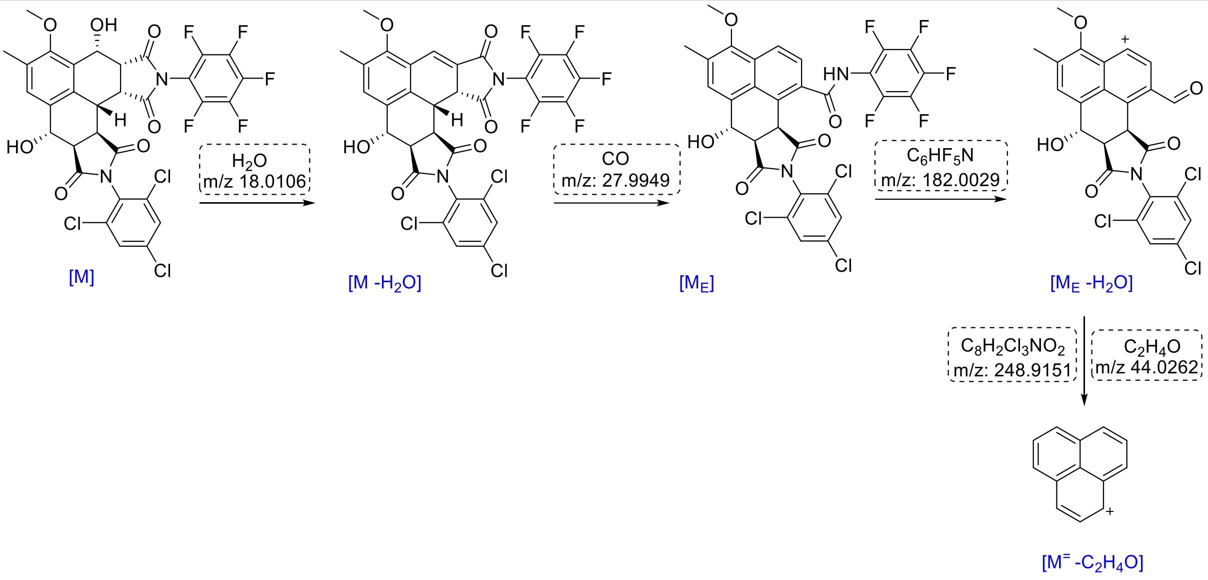

Scheme S12 Fragments identified in the ESI-MS spectrum **Figure S104** and corresponding abbreviations.

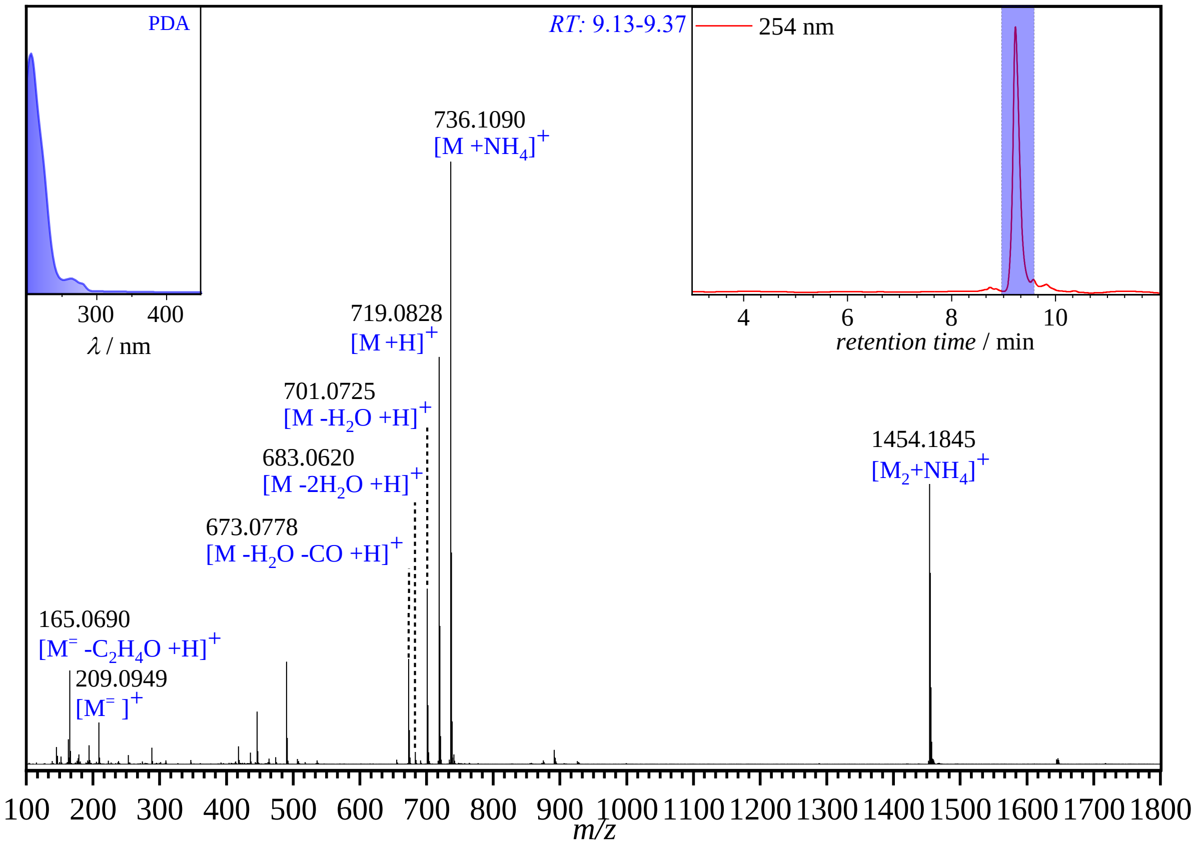

Figure S106 LC-trace (254 nm detector wavelength), accumulated mass-spectra and accumulated PDA spectra of (3a*R*,4*R*,8*R*,8a*R*,11a*R*,11b*R*,11c*R*)-4,8-dihydroxy-5-methoxy-6-methyl-2,10-bis(perfluorophenyl)-3a,8,8a,11a,11b,11c-hexahydro-1*H*-pyrrolo[3',4':2,3]naphtho[1,8-*ef*]-isoindole-1,3,9,11(2*H*,4*H*,10*H*)-tetraone (**4f**).

Table S15 Collation of observed signals in the mass spectrum **Figure S106** and comparison with theoretically expected *m/z* values for the assigned signals. The abbreviations and fragmentation pattern are defined in **Scheme S13**.

Symbol	m/z^{exp}	m/z^{theor}	Δ_{ppm}	composition
$[M_2 + NH_4]^+$	1454.1845	1454.1934	6.12	$C_{62}H_{36}F_{20}N_5O_{14}^+$
$[M + Na]^+$	736.1090	736.1136	6.25	$C_{31}H_{20}F_{10}N_3O_7^+$
$[M + H]^+$	719.0828	719.0871	5.98	$C_{31}H_{17}F_{10}N_2O_7^+$
$[M - H_2O + H]^+$	701.0725	701.0765	5.71	$C_{31}H_{15}F_{10}N_2O_6^+$
$[M - 2H_2O + H]^+$	683.062	683.0659	5.71	$C_{31}H_{13}F_{10}N_2O_5^+$
$[M - H_2O - CO + H]^+$	673.0778	673.0816	5.65	$C_{30}H_{15}F_{10}N_2O_5^+$
$[M^=]^+$	209.0949	209.0961	5.74	$C_{15}H_{13}O^+$
$[M^= - C_2H_4O]^+$	165.0690	165.0699	5.45	$C_{13}H_9^+$

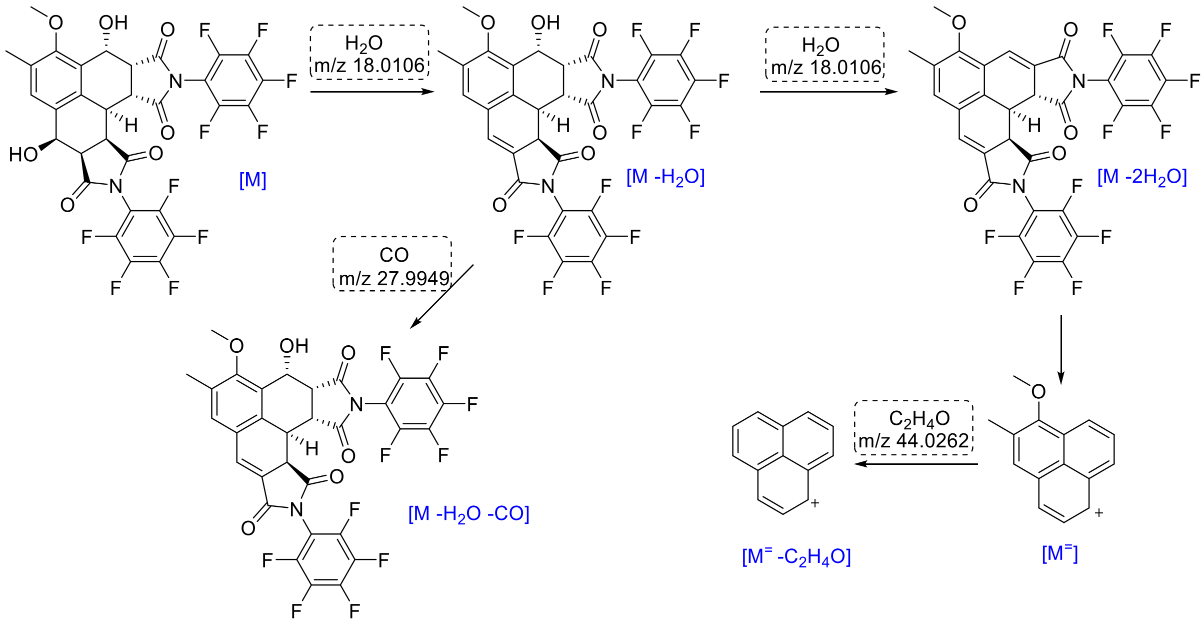

Scheme S13 Fragments identified in the ESI-MS spectrum **Figure S106** and corresponding abbreviations.

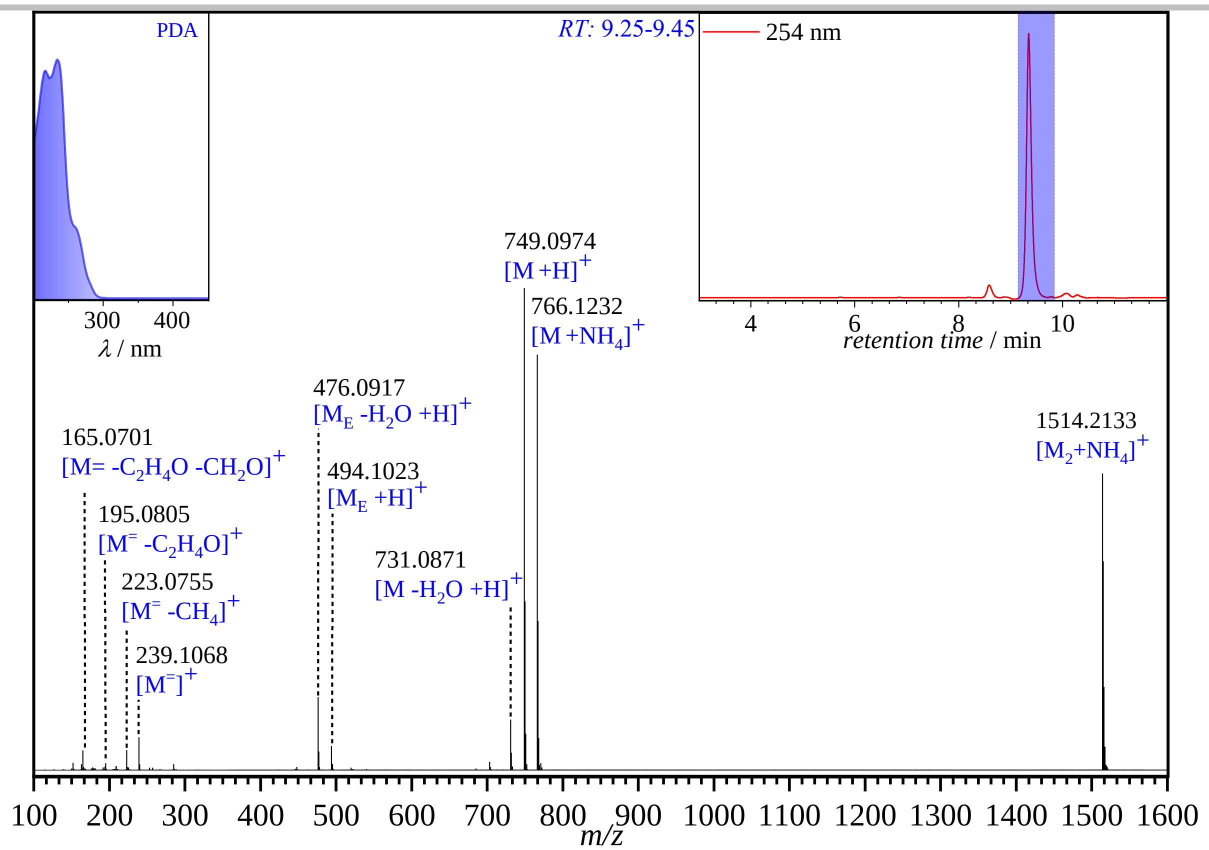

Figure S107 LC-trace (254 nm detector wavelength), accumulated mass-spectra and accumulated PDA spectra of (3a*R*,4*R*,8*R*,8a*R*,11a*R*,11c*R*)-4,8-dihydroxy-5,7-dimethoxy-6-methyl-2,10-bis(perfluorophenyl)-3a,8,8a,11a,11b,11c-hexahydro-1*H*-pyrrolo[3',4':2,3]naphtho[1,8-*ef*]isoindole-1,3,9,11(2*H*,4*H*,10*H*)-tetraone (**4g**).

Table S16 | Collation of observed signals in the mass spectrum **Figure S107** and comparison with theoretically expected *m/z* values for the assigned signals. The abbreviations and fragmentation pattern are defined in **Scheme S14**.

Symbol	m/z^{exp}	m/z^{theor}	Δ_{ppm}	composition
$[M_2 + NH_4]^+$	1514.2133	1514.2145	0.79	$C_{64}H_{40}F_{20}N_5O_{16}^+$
$[M + NH_4]^+$	766.1232	766.1242	1.31	$C_{32}H_{22}F_{10}N_3O_8^+$
$[M + H]^+$	749.0974	749.0976	0.27	$C_{32}H_{19}F_{10}N_2O_8^+$
$[M - H_2O + H]^+$	731.0871	731.0871	0.00	$C_{32}H_{17}F_{10}N_2O_7^+$
$[M_E + H]^+$	494.1023	494.1021	0.40	$C_{24}H_{17}F_5NO_5^+$
$[M_E - H_2O + H]^+$	476.0917	476.0916	0.21	$C_{24}H_{15}F_5NO_4^+$
$[M^=]^+$	239.1068	239.1067	0.42	$C_{16}H_{15}O_2^+$
$[M^= - CH_4]^+$	223.0755	223.0754	0.45	$C_{15}H_{11}O_2^+$
$[M^= - C_2H_4O]^+$	195.0805	195.0804	0.51	$C_{14}H_{11}O^+$
$[M^= - C_2H_4O - CH_2O]^+$	165.0701	165.0699	1.21	$C_{13}H_9^+$

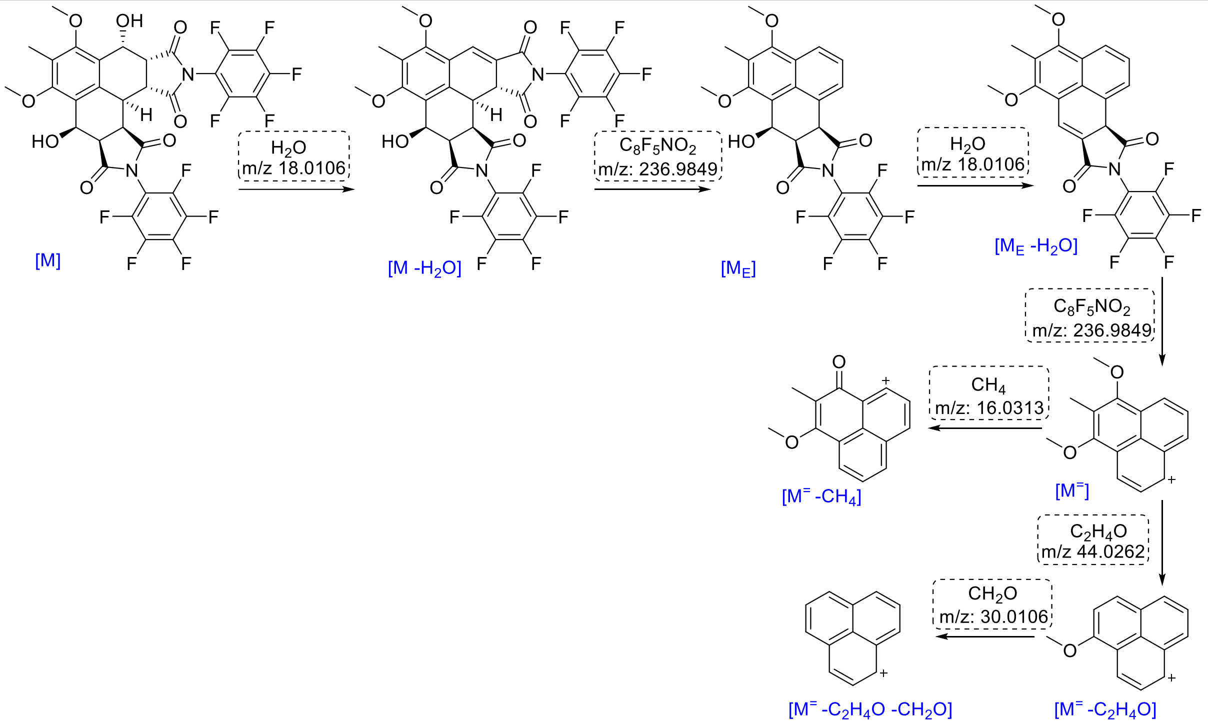

Scheme S14 Fragments identified in the ESI-MS spectrum **Figure S107** and corresponding abbreviations.

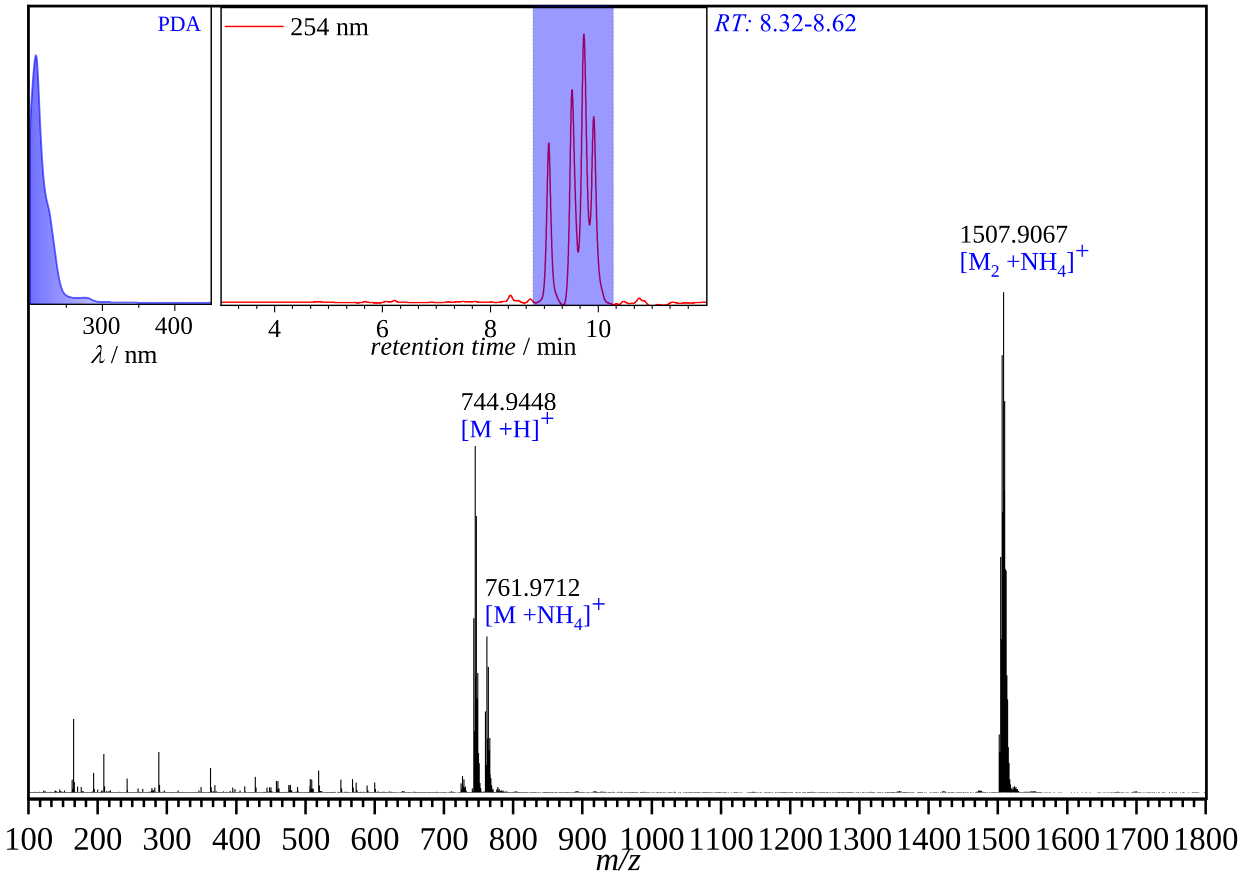

Figure S108 LC-trace (254 nm detector wavelength), accumulated mass-spectra and accumulated PDA spectra of product mixture (diastereomers) 4,8-dihydroxy-5-methoxy-6-methyl-2,10-bis(2,4,6-trichlorophenyl)-3a,8,8a,11a,11b,11c-hexahydro-1*H*-pyrrolo[3',4':2,3]naphtho[1,8-*ef*]-isoindole-1,3,9,11(2*H*,4*H*,10*H*)-tetraone (**4h**), containing the 4 possible endo/exo combinations. The individual products were not isolated.

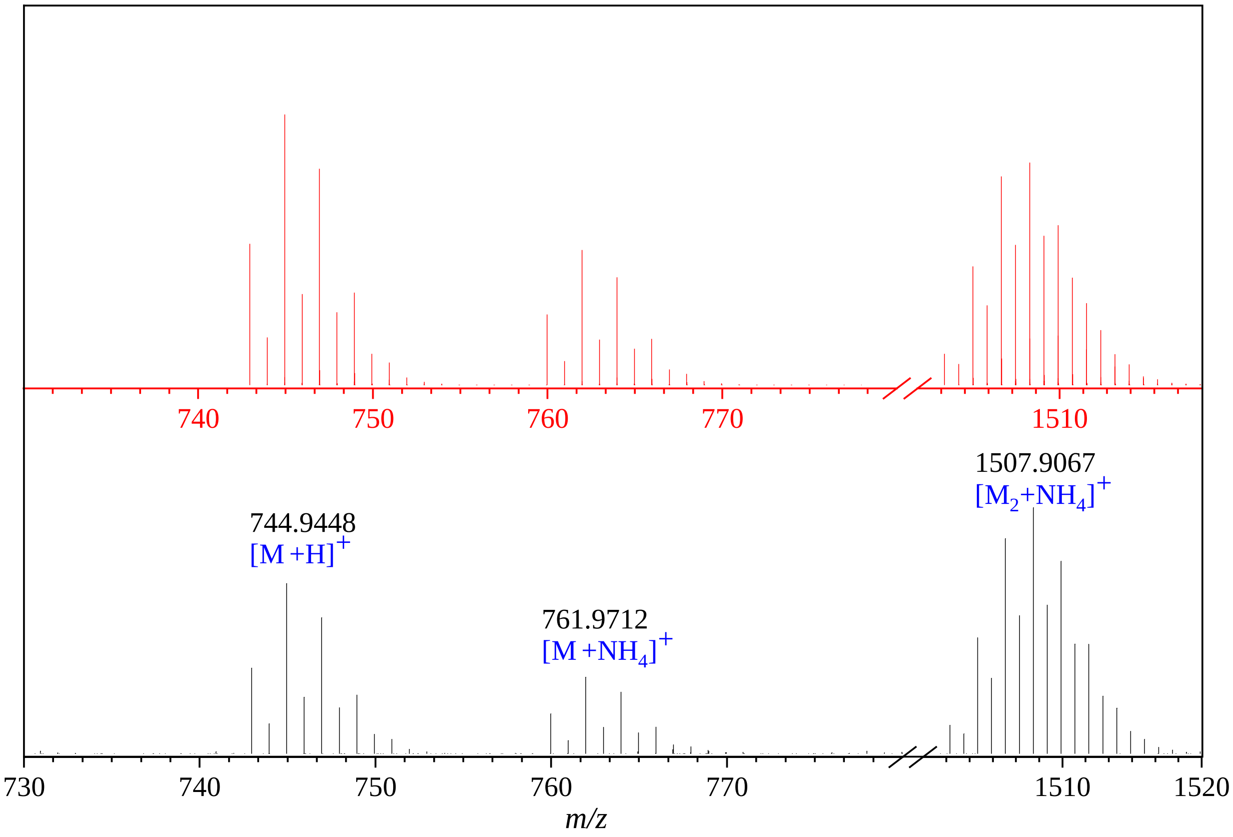

Figure S109 Simulated (red) and experimental spectra of product mixture (diastereomers) 4,8-dihydroxy-5-methoxy-6-methyl-2,10-bis(2,4,6-trichlorophenyl)-3a,8,8a,11a,11b,11c-hexahydro-1*H*-pyrrolo[3',4':2,3]naphtho[1,8-*ef*]isoindole-1,3,9,11(2*H*,4*H*,10*H*)-tetraone (**4h**) (black) showing the isotopic patters of the major signals in the mass spectrum (refer to **Figure S108**).

Table S17 Collation of observed signals in the mass spectrum **Figure S118** and comparison with theoretically expected *m/z* values for the assigned signals.

Symbol	m/z^{exp}	m/z^{theor}	Δ_{ppm}	composition
$[M_2 +NH_4]^+$	1507.9067	1507.9053	0.93	$C_{62}H_{44}Cl_{12}N_5O_{14}^+$
$[M +NH_4]^+$	761.9712	761.9710	0.26	$C_{31}H_{24}Cl_6N_3O_7^+$
$[M +H]^+$	744.9448	744.9445	0.40	$C_{31}H_{21}Cl_6N_2O_7^+$

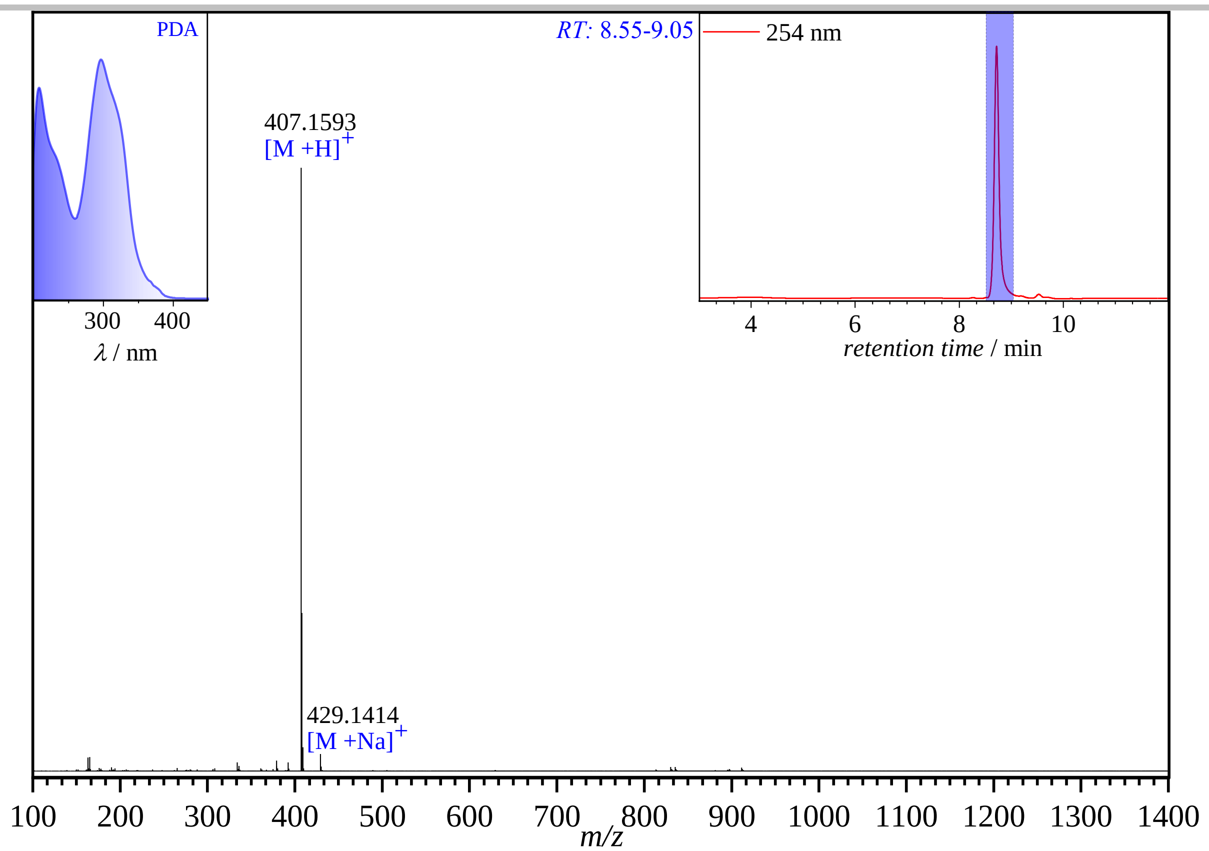

Figure S110 LC-trace (254 nm detector wavelength), accumulated mass-spectra and accumulated PDA spectra of (11a*R*,11b*R*,11c*R*)-2,10-diethyl-5-methoxy-6-methyl-11b,11c-dihydro-1*H*-pyrrolo[3',4':2,3]naphtho[1,8-*ef*]isoindole-1,3,9,11-(2*H*,10*H*,11a*H*)-tetraone (**5a**).

Table S18 Collation of observed signals in the mass spectrum **Figure S110** and comparison with theoretically expected *m/z* values for the assigned signals.

Symbol	m/z^{exp}	m/z^{theor}	Δ_{ppm}	composition
$[M +Na]^+$	429.1414	429.1421	1.63	$C_{23}H_{22}N_2NaO_5^+$
$[M +H]^+$	407.1593	407.1601	1.96	$C_{23}H_{23}N_2O_5^+$

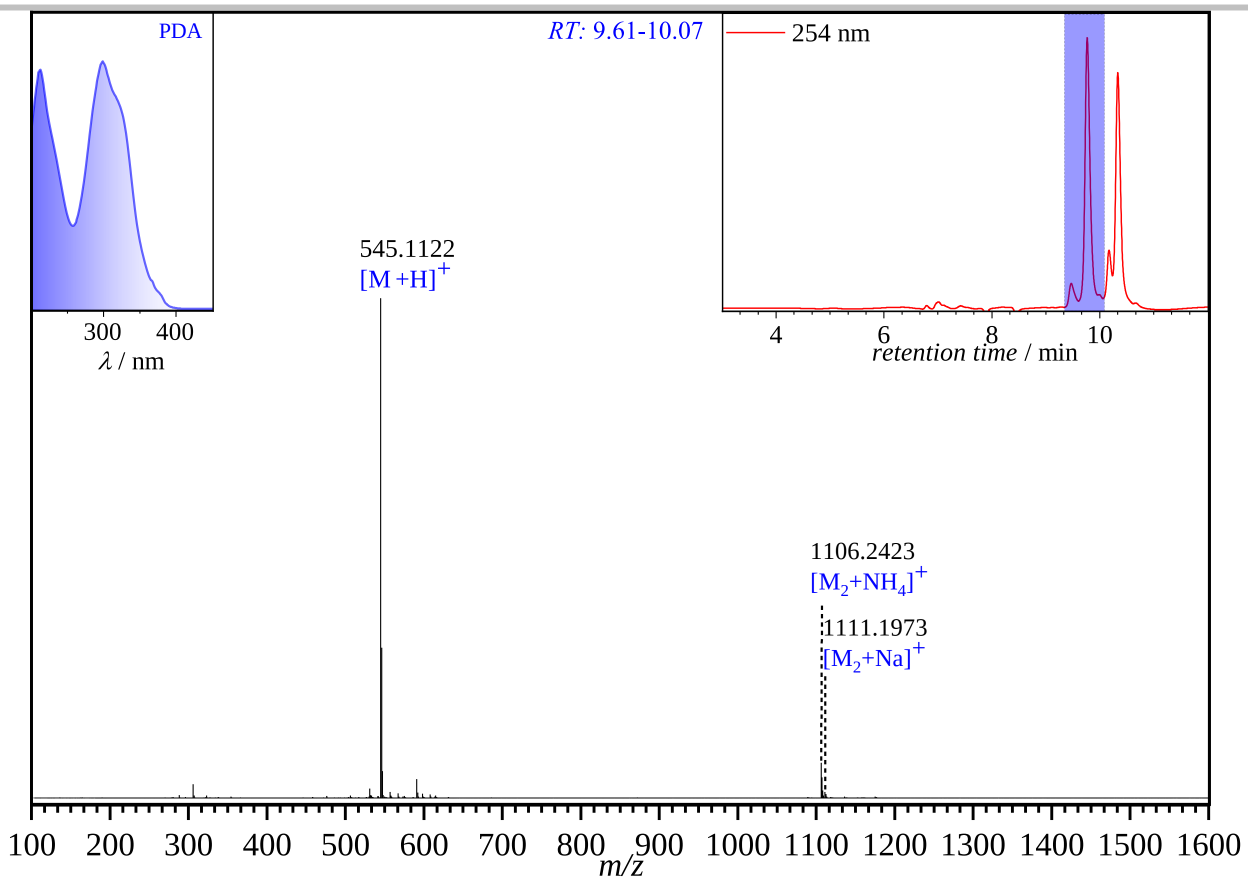

Figure S111 LC-trace (254 nm detector wavelength), accumulated mass-spectra and accumulated PDA spectra of (11a*R*,11b*S*,11c*R*)-2-ethyl-5-methoxy-6-methyl-10-(perfluorophenyl)-11b,11c-dihydro-1*H*-pyrrolo[3',4':2,3]naphtho[1,8-*ef*]isoindole-1,3,9,11(2*H*,10*H*,11a*H*)-tetraone (**5d**).The accumulated mass-spectra of the peaks RT = 10.09-10.52 is depicted in **Figure S112**. **5d** seems to be unstable to LCMS conditions.

Table S19 Collation of observed signals in the mass spectrum **Figure S111** and comparison with theoretically expected *m/z* values for the assigned signals.

Symbol	m/z^{exp}	m/z^{theor}	Δ_{ppm}	composition
$[M_2+Na]^+$	1111.1973	1111.2007	3.06	$C_{62}H_{24}F_{20}N_4NaO_{10}^+$
$[M_2+NH_4]^+$	1106.2423	1106.2454	2.80	$C_{54}H_{38}F_{10}N_5O_{10}^+$
$[M+H]^+$	545.1122	545.1130	1.47	$C_{27}H_{18}F_5N_2O_5^+$

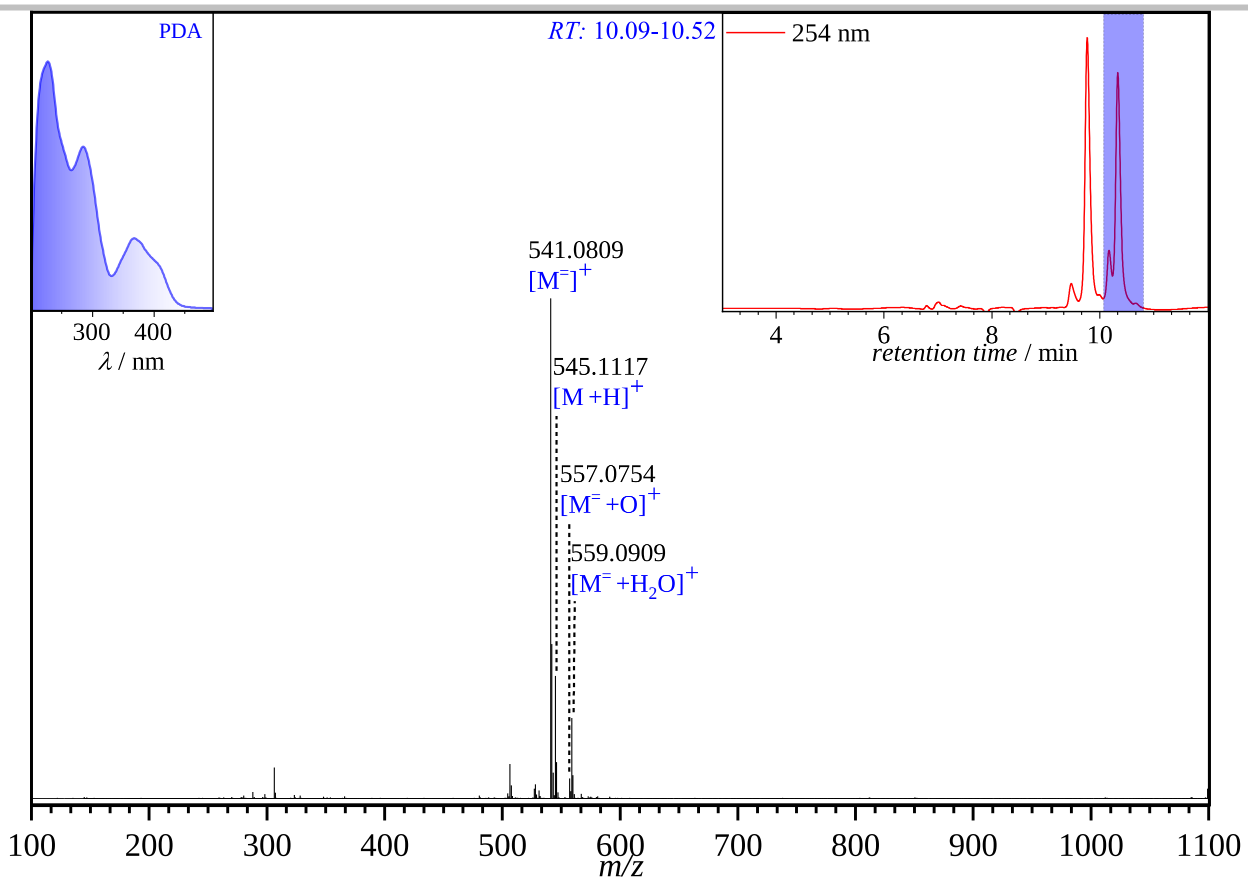

Figure S112 LC-trace (254 nm detector wavelength), accumulated mass-spectra and accumulated PDA spectra of (11a*R*,11b*S*,11c*R*)-2-ethyl-5-methoxy-6-methyl-10-(perfluorophenyl)-11b,11c-dihydro-1*H*-pyrrolo[3',4':2,3]naphtho[1,8-*ef*]isoindole-1,3,9,11(2*H*,10*H*,11a*H*)-tetraone (**5d**).

Table S20 Collation of observed signals in the mass spectrum **Figure S112** and comparison with theoretically expected *m/z* values for the assigned signals. The abbreviations and fragmentation pattern are defined in **Scheme S15Scheme S14**.

Symbol	m/z^{exp}	m/z^{theor}	Δ_{ppm}	composition
$[M^{=} +H_2O]^+$	559.0909	559.0923	2.50	$C_{27}H_{16}F_5N_2O_6^+$
$[M^{=} +O]^+$	557.0754	557.0767	2.33	$C_{27}H_{14}F_5N_2O_6^+$
$[M +H]^+$	545.1117	545.1130	2.38	$C_{27}H_{18}F_5N_2O_5^+$
$[M^{=}]^+$	541.0809	541.0817	1.48	$C_{27}H_{14}F_5N_2O_5^+$

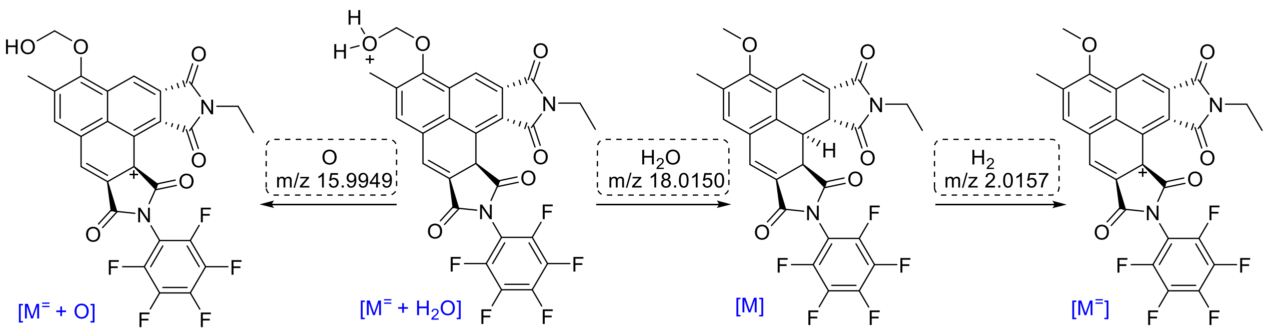

Scheme S15 Fragments identified in the ESI-MS spectrum **Figure S112** and corresponding abbreviations.

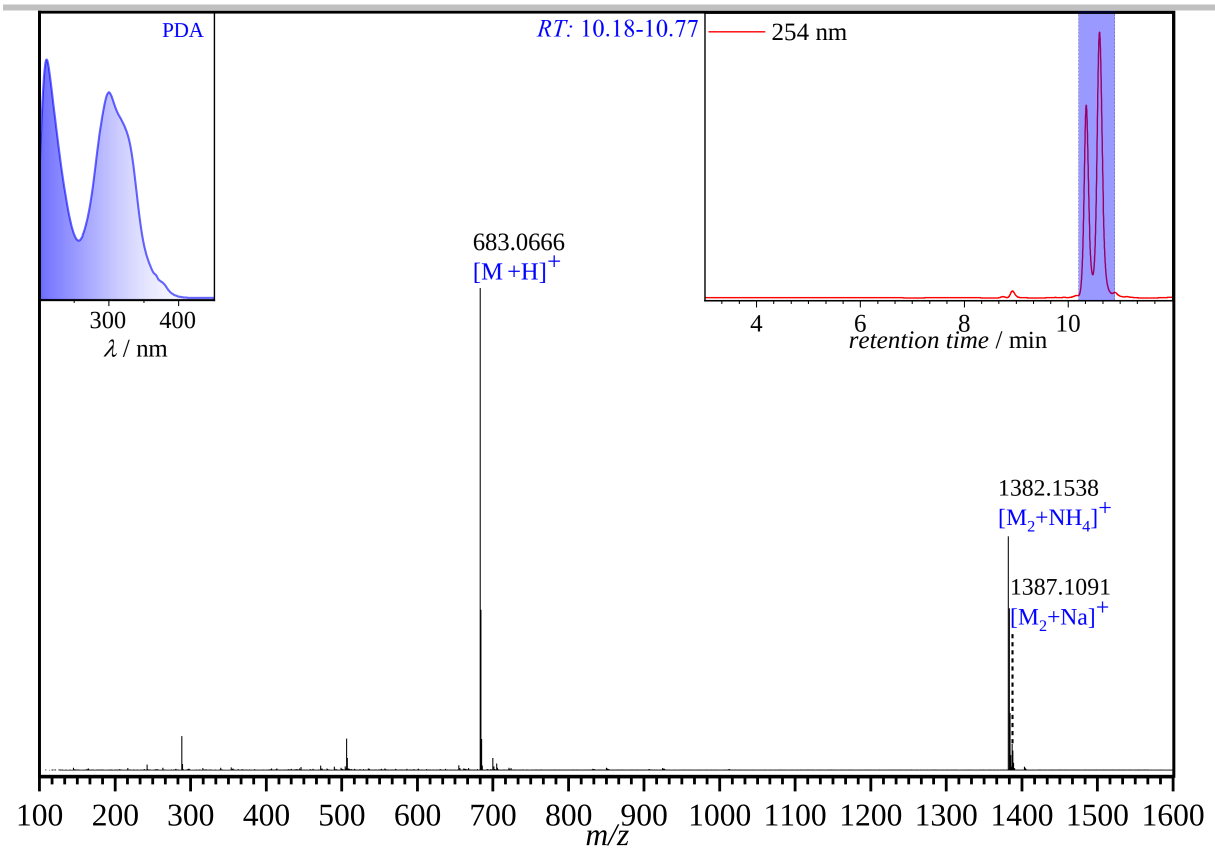

Figure S113 LC-trace (254 nm detector wavelength), accumulated mass-spectra and accumulated PDA spectra of (11a*R*,11b*R*,11c*R*)-5-methoxy-6-methyl-2,10-bis(perfluorophenyl)-11b,11c-dihydro-1*H*-pyrrolo[3',4':2,3]naphtho[1,8-*ef*]isoindole-1,3,9,11(2*H*,10*H*,11a*H*)-tetraone (**5f**).

Table S21 Collation of observed signals in the mass spectrum **Figure S113** and comparison with theoretically expected *m/z* values for the assigned signals.

Symbol	m/z^{exp}	m/z^{theor}	Δ_{ppm}	composition
$[M_2 + Na]^+$	1387.1091	1387.1065	1.87	$C_{62}H_{24}F_{20}N_4NaO_{10}^+$
$[M_2 + NH_4]^+$	1382.1538	1382.1511	1.95	$C_{62}H_{28}F_{20}N_5O_{10}^+$
$[M + H]^+$	683.0666	683.0659	1.02	$C_{31}H_{13}F_{10}N_2O_5^+$

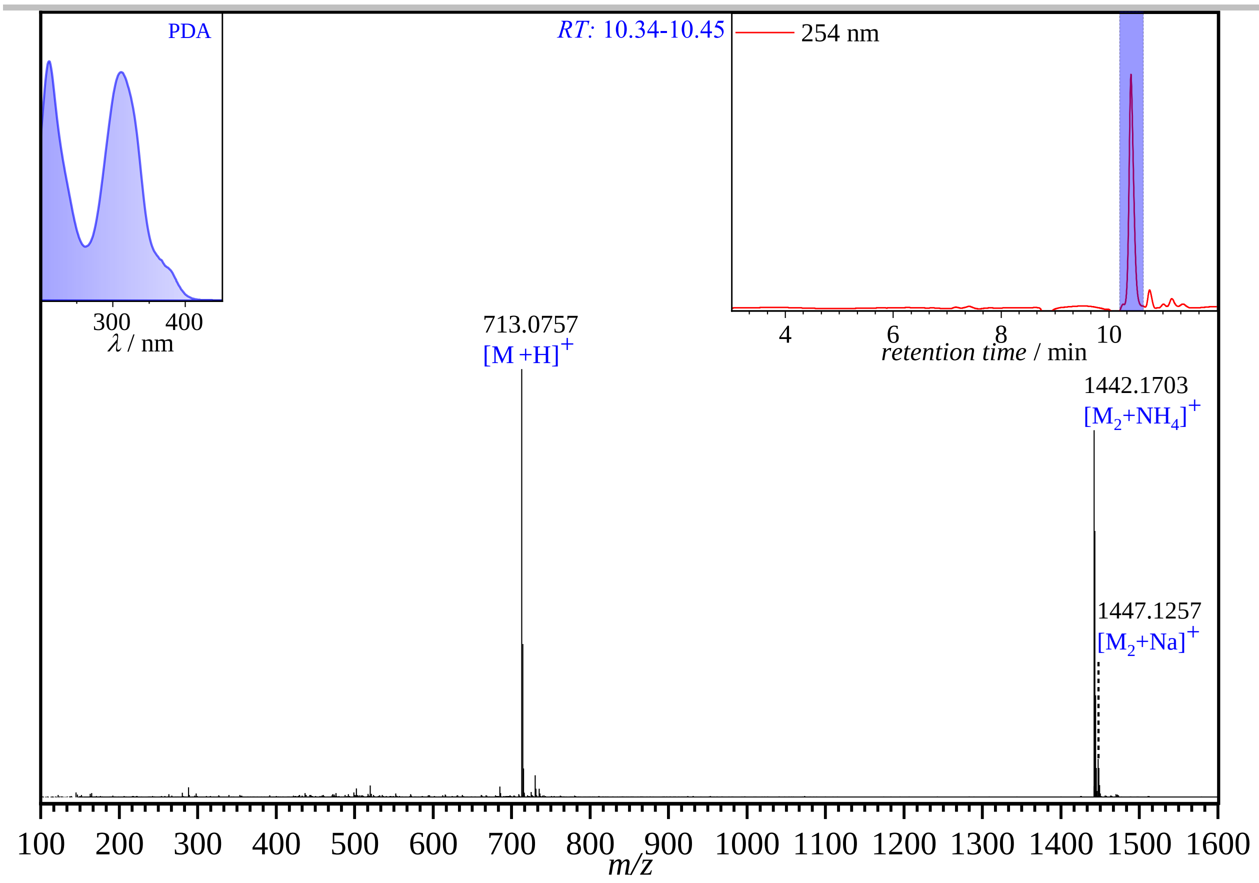

Figure S114 LC-trace (254 nm detector wavelength), accumulated mass-spectra and accumulated PDA spectra of (11a*R*,11c*R*)-5,7-dimethoxy-6-methyl-2,10-bis(perfluorophenyl)-11b,11c-dihydro-1*H*-pyrrolo[3',4':2,3]naphtho[1,8-*ef*]isoindole-1,3,9,11(2*H*,10*H*,11a*H*)-tetraone (**5g**).

Table S22 Collation of observed signals in the mass spectrum **Figure S114** and comparison with theoretically expected *m/z* values for the assigned signals.

Symbol	m/z^{exp}	m/z^{theor}	Δ_{ppm}	composition
$[M_2+Na]^+$	1447.1257	1447.1277	1.38	$C_{64}H_{28}F_{20}N_4NaO_{12}^+$
$[M_2+NH_4]^+$	1442.1703	1442.1723	1.39	$C_{64}H_{32}F_{20}N_5O_{12}^+$
$[M+H]^+$	713.0757	713.0765	1.12	$C_{32}H_{15}F_{10}N_2O_6^+$

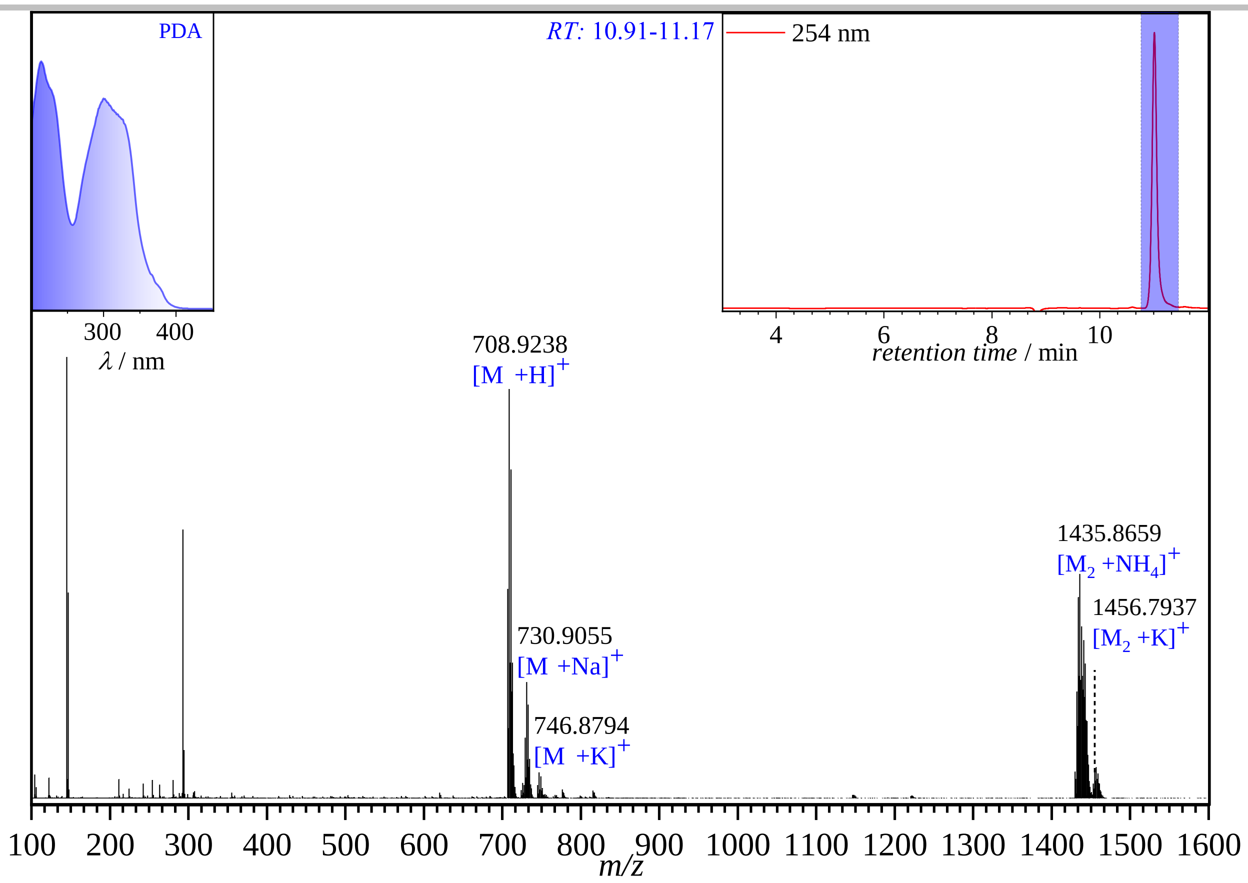

Figure S115 LC-trace (254 nm detector wavelength), accumulated mass-spectra and accumulated PDA spectra of (11a*R*,11b*R*,11c*R*)-5-methoxy-6-methyl-2,10-bis(2,4,6-trichlorophenyl)-11b,11c-dihydro-1*H*-pyrrolo[3',4':2,3]naphtho[1,8-*ef*]isoindole-1,3,9,11(2*H*,10*H*,11a*H*)-tetraone (**5h**).

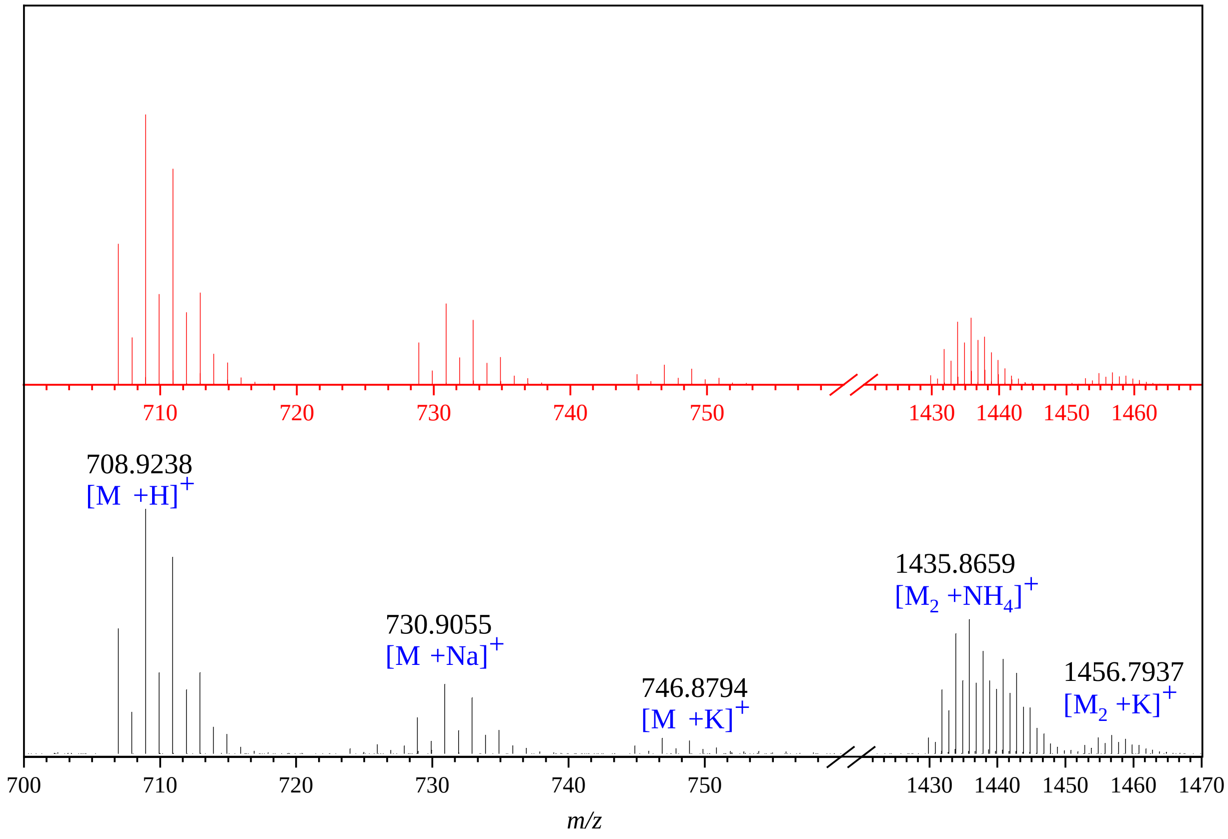

Figure S116 Simulated (red) and experimental spectra of (11a*R*,11b*R*,11c*R*)-5-methoxy-6-methyl-2,10-bis(2,4,6-trichlorophenyl)-11b,11c-dihydro-1*H*-pyrrolo[3',4':2,3]naphtho[1,8-*ef*]isoindole-1,3,9,11(2*H*,10*H*,11a*H*)-tetraone (**5h**) (black) showing the isotopic patters of the major signals in the mass spectrum (refer to **Figure S115**).

Table S23 Collation of observed signals in the mass spectrum **Figure S115** and comparison with theoretically expected *m/z* values for the assigned signals.

Symbol	m/z^{exp}	m/z^{theor}	Δ_{ppm}	composition
$[M_2 +K]^+$	1456.7937	1456.7924	0.89	$C_{62}H_{32}Cl_{12}KN_4O_{10}^+$
$[M_2 +NH_4]^+$	1435.8659	1435.8631	1.95	$C_{62}H_{36}Cl_{12}N_5O_{10}^+$
$[M +K]^+$	746.8794	746.8792	0.27	$C_{31}H_{16}Cl_6KN_2O_5^+$
$[M +Na]^+$	730.9055	730.9053	0.27	$C_{31}H_{16}Cl_6N_2NaO_5^+$
$[M +H]^+$	708.9238	708.9234	0.56	$C_{31}H_{17}Cl_6N_2O_5^+$

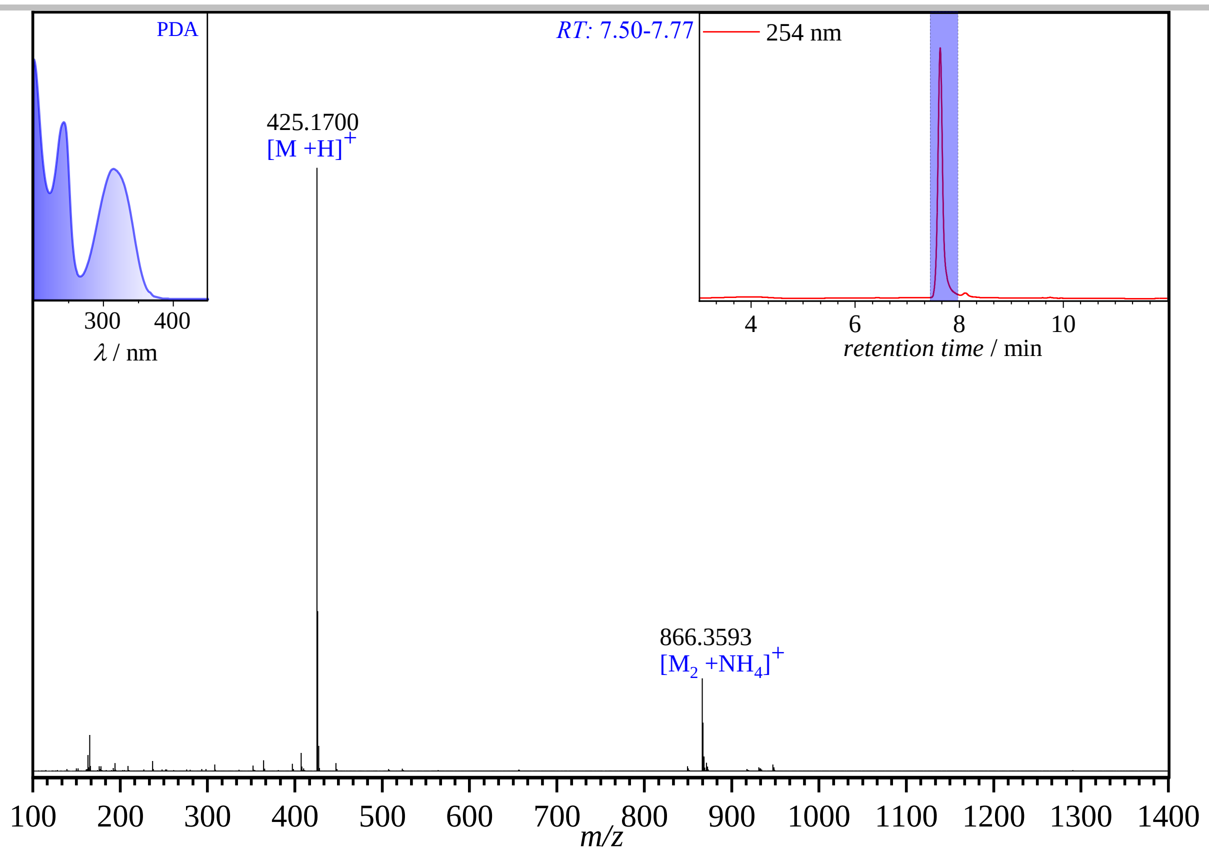

Figure S117 LC-trace (254 nm detector wavelength), accumulated mass-spectra and accumulated PDA spectra of (3a*R*,4*R*,11a*R*,11b*R*,11c*R*)-2,10-diethyl-4-hydroxy-7-methoxy-6-methyl-3a,11a,11b,11c-tetrahydro-1*H*-pyrrolo[3',4':2,3]naphtho[1,8-*ef*]isoindole-1,3,9,11(2*H*,4*H*,10*H*)-tetraone (**7a**).

Table S24 Collation of observed signals in the mass spectrum **Figure S117** and comparison with theoretically expected *m/z* values for the assigned signals.

Symbol	m/z^{exp}	m/z^{theor}	Δ_{ppm}	composition
$[M + NH_4]^+$	866.3593	866.3607	1.62	$C_{46}H_{52}N_5O_{12}^+$
$[M + H]^+$	425.1700	425.1707	1.65	$C_{23}H_{25}N_2O_6^+$

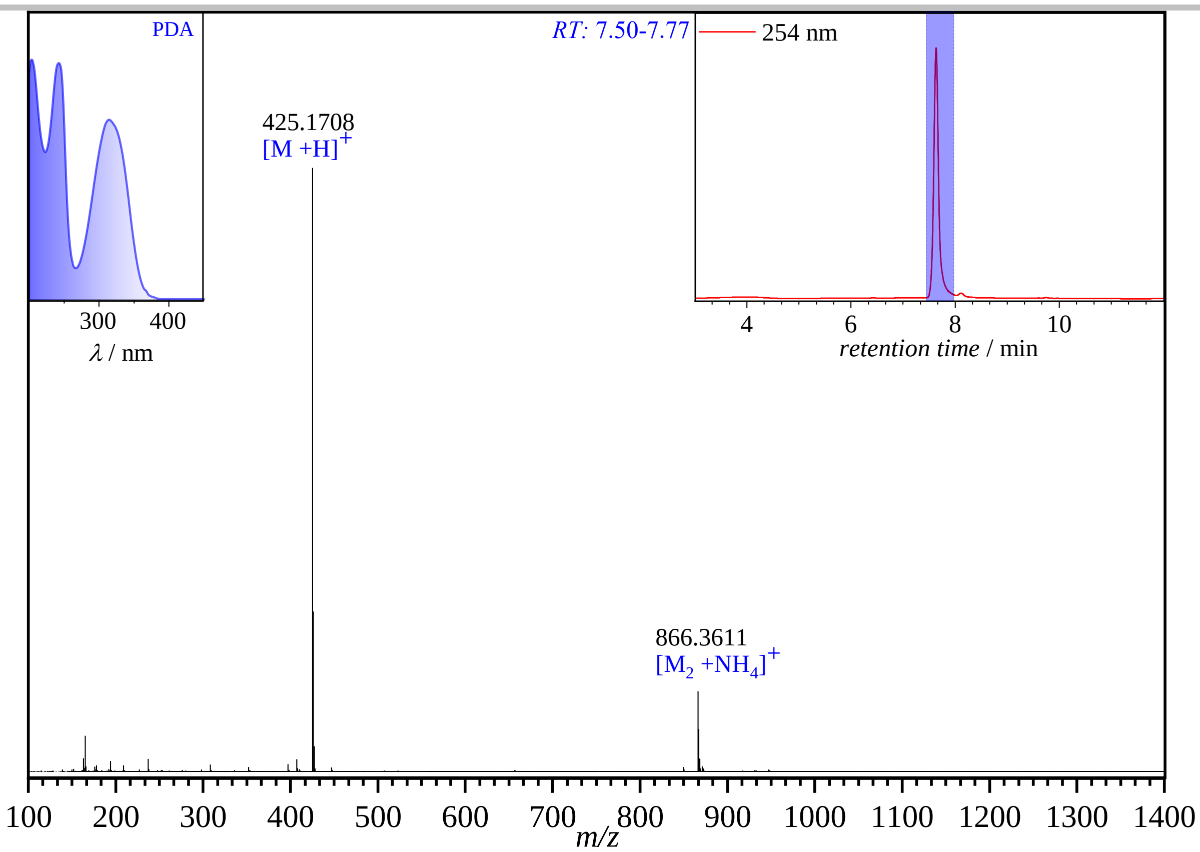

Figure S118 LC-trace (254 nm detector wavelength), accumulated mass-spectra and accumulated PDA spectra of (3a*R*,4*R*,11a*R*,11b*S*,11c*R*)-2,10-diethyl-4-hydroxy-5-methoxy-6-methyl-3a,11a,11b,11c-tetrahydro-1H-pyrrolo[3',4':2,3]naphtho[1,8-*ef*]isoindole-1,3,9,11(2*H*,4*H*,10*H*)-tetraone (**8a**).

Table S25 Collation of observed signals in the mass spectrum **Figure S118** and comparison with theoretically expected *m/z* values for the assigned signals.

Symbol	m/z^{exp}	m/z^{theor}	Δ_{ppm}	composition
$[M_2 + NH_4]^+$	866.3611	866.3607	0.46	$C_{46}H_{52}N_5O_{12}^+$
$[M + H]^+$	425.1708	425.1707	0.24	$C_{23}H_{25}N_2O_6^+$

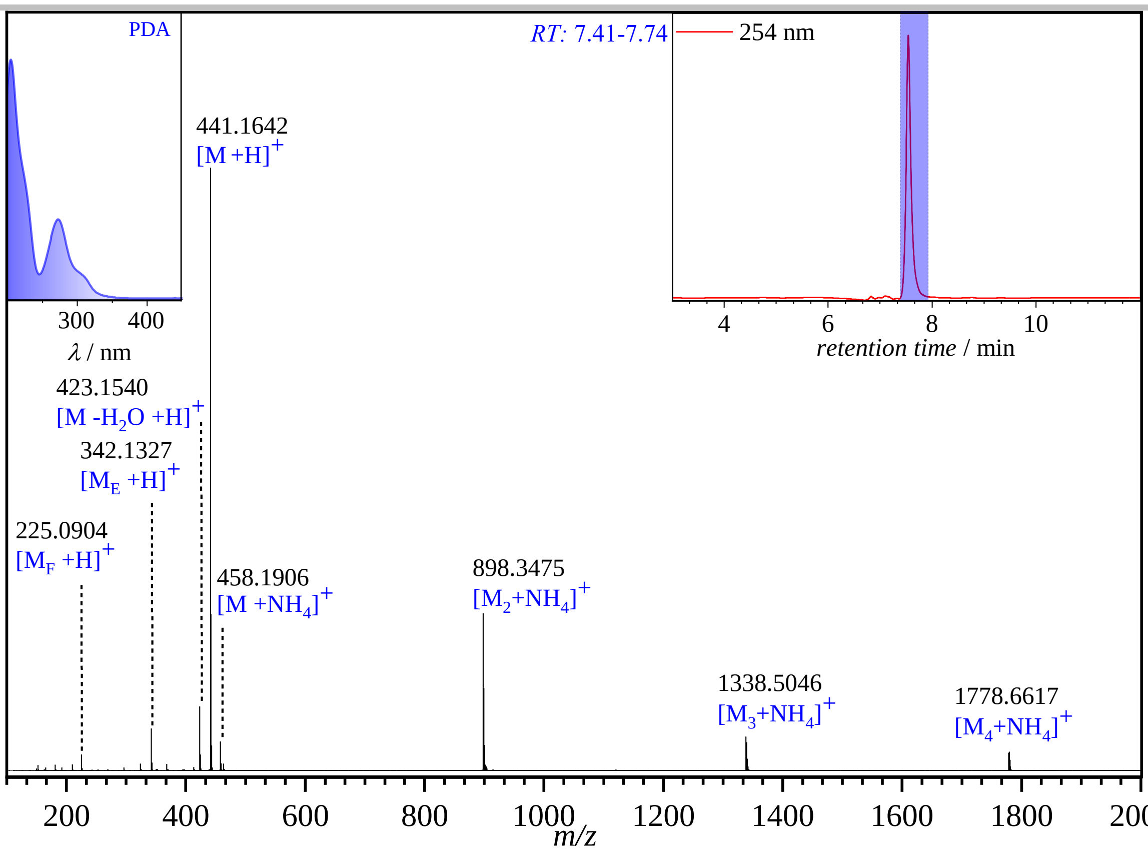

Figure S119 LC-trace (254 nm detector wavelength), accumulated mass-spectra and accumulated PDA spectra of (3a*R*,8*R*,8a*R*,11a*R*,11b*S*,11c*R*)-2,10-diethyl-8-hydroxy-7-methoxy-6-methyl-3a,8,8a,11a,11b,11c-hexahydro-1*H*-pyrrolo[3',4':2,3]-naphtho[1,8-*ef*]isoindole-1,3,4,9,11-(2*H*,10*H*)-pentaone (**9a**).

Table S26 Collation of observed signals in the mass spectrum **Figure S119** and comparison with theoretically expected *m/z* values for the assigned signals. The abbreviations and fragmentation pattern are defined in **Scheme S16**.

Symbol	m/z^{exp}	m/z^{theor}	Δ_{ppm}	composition
$[M_4 + NH_4]^+$	1778.6617	1778.6672	3.09	$C_{92}H_{100}N_9O_{28}^+$
$[M_3 + NH_4]^+$	1338.5046	1338.5089	3.21	$C_{69}H_{76}N_7O_{21}^+$
$[M_2 + NH_4]^+$	898.3475	898.3505	3.34	$C_{46}H_{52}N_5O_{14}^+$
$[M + NH_4]^+$	458.1906	458.1922	3.49	$C_{23}H_{28}N_3O_7^+$
$[M + H]^+$	441.1642	441.1656	3.17	$C_{23}H_{25}N_2O_7^+$
$[M - H_2O + H]^+$	423.154	423.1551	2.60	$C_{23}H_{23}N_2O_6^+$
$[M_E + H]^+$	342.1327	342.1336	2.63	$C_{19}H_{20}NO_5^+$
$[M_F + H]^+$	225.0904	225.091	2.67	$C_{15}H_{13}O_2^+$

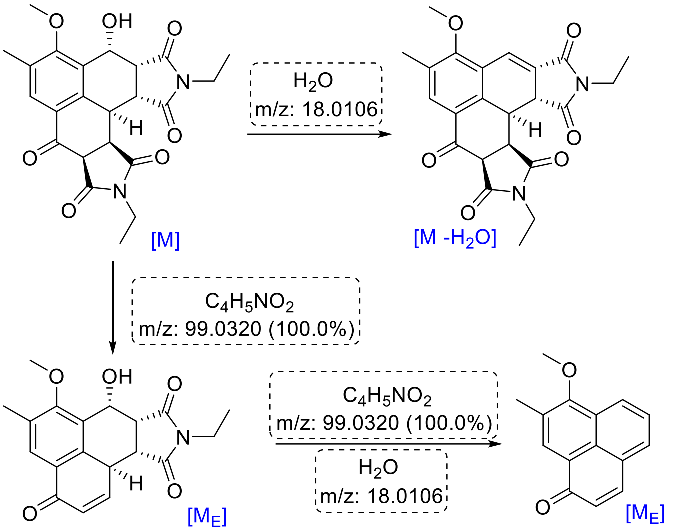

Scheme S16 Fragments identified in the ESI-MS spectrum **Figure S119** and corresponding abbreviations.

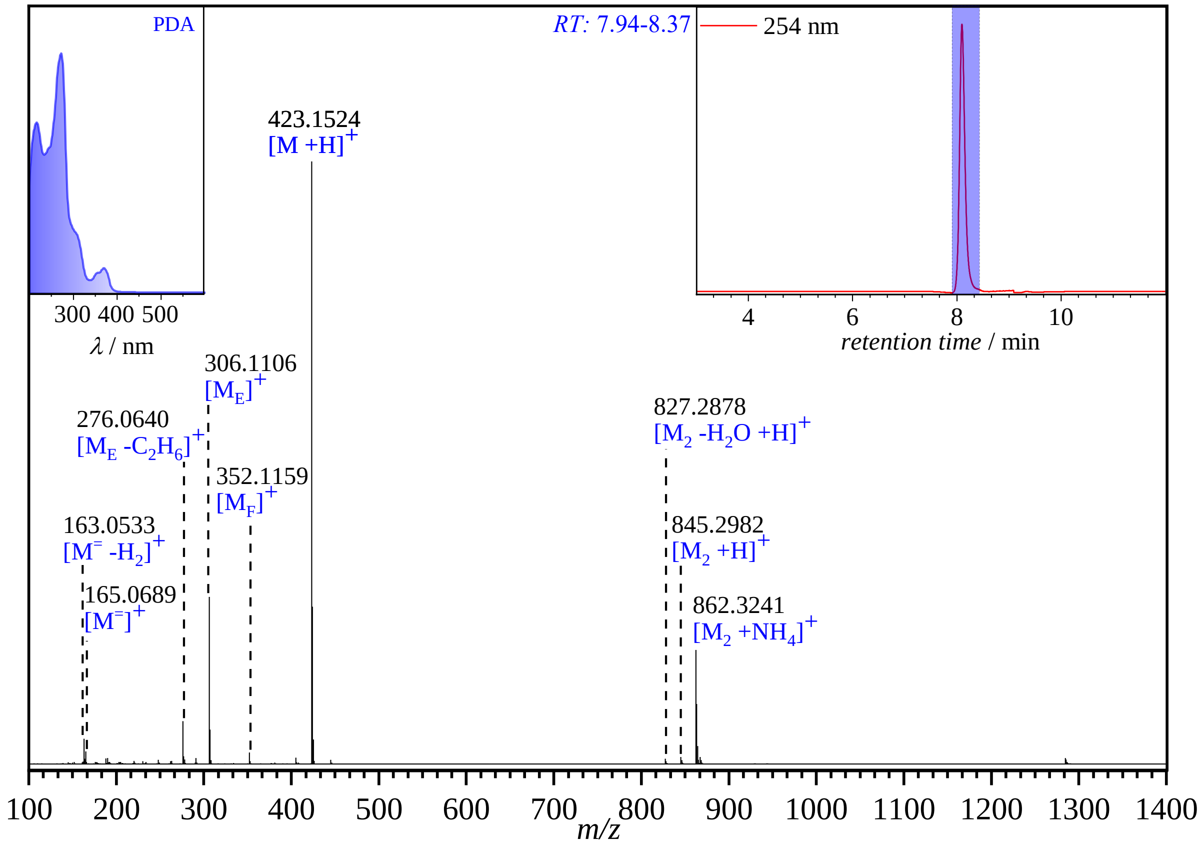

Figure S120 LC-trace (254 nm detector wavelength), accumulated mass-spectra and accumulated PDA spectra of (3a*R*,4*R*,11c*S*)-2,10-diethyl-4-hydroxy-5-methoxy-6-methyl-3a,11c-dihydro-1*H*-pyrrolo[3',4':2,3]naphtho[1,8-*ef*]isoindole-1,3,9,11(2*H*,4*H*,10*H*)-tetraone (**10a**).

Table S27 Collation of observed signals in the mass spectrum **Figure S120** and comparison with theoretically expected *m/z* values for the assigned signals. The abbreviations and fragmentation pattern are defined in **Scheme S17**.

Symbol	m/z^{exp}	m/z^{theor}	Δ_{ppm}	composition
$[M_2 + NH_4]^+$	862.3241	862.3294	6.15	$C_{46}H_{48}N_5O_{12}^+$
$[M_2 + H]^+$	845.2982	845.3028	5.44	$C_{46}H_{45}N_4O_{12}^+$
$[M_2 - H_2O + H]^+$	827.2878	827.2923	5.44	$C_{46}H_{43}N_4O_{11}^+$
$[M + H]^+$	423.1524	423.1551	6.38	$C_{23}H_{23}N_2O_6^+$
$[M_F]^+$	352.1159	352.1179	5.68	$C_{20}H_{18}NO_5^+$
$[M_E]^+$	306.1106	306.1125	6.21	$C_{19}H_{16}NO_3^+$
$[M_E - C_2H_6]^+$	276.0640	276.0655	5.43	$C_{17}H_{10}NO_3^+$
$[M^=]^+$	165.0689	165.0699	6.06	$C_{13}H_9^+$
$[M^=-H_2]^+$	163.0533	163.0542	5.52	$C_{13}H_7^+$

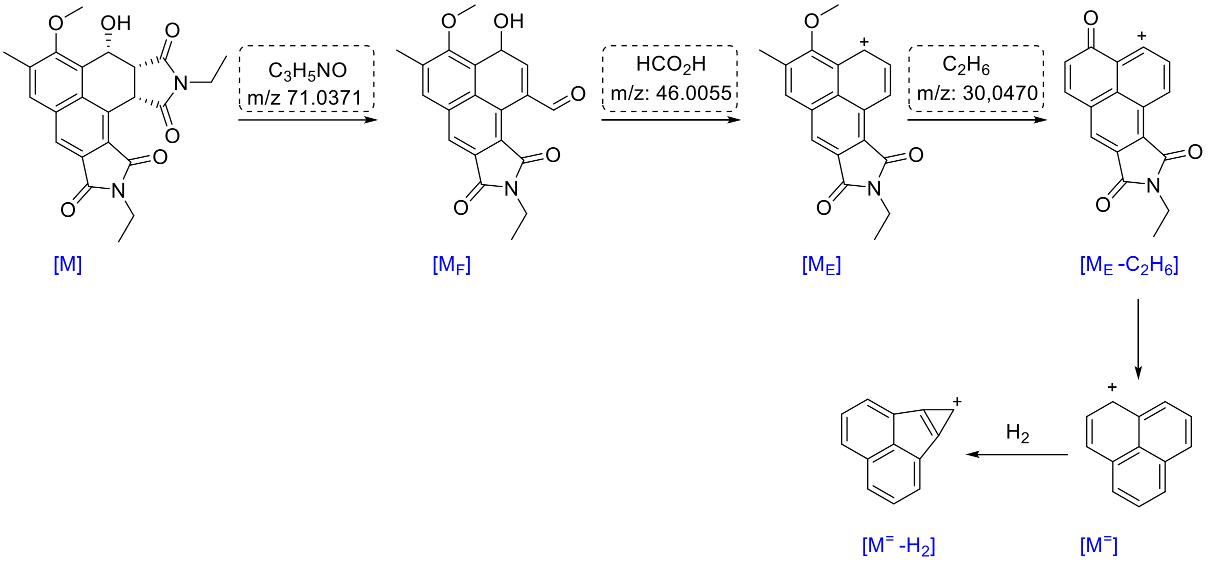

Scheme S17 Fragments identified in the ESI-MS spectrum **Figure S120** and corresponding abbreviations.

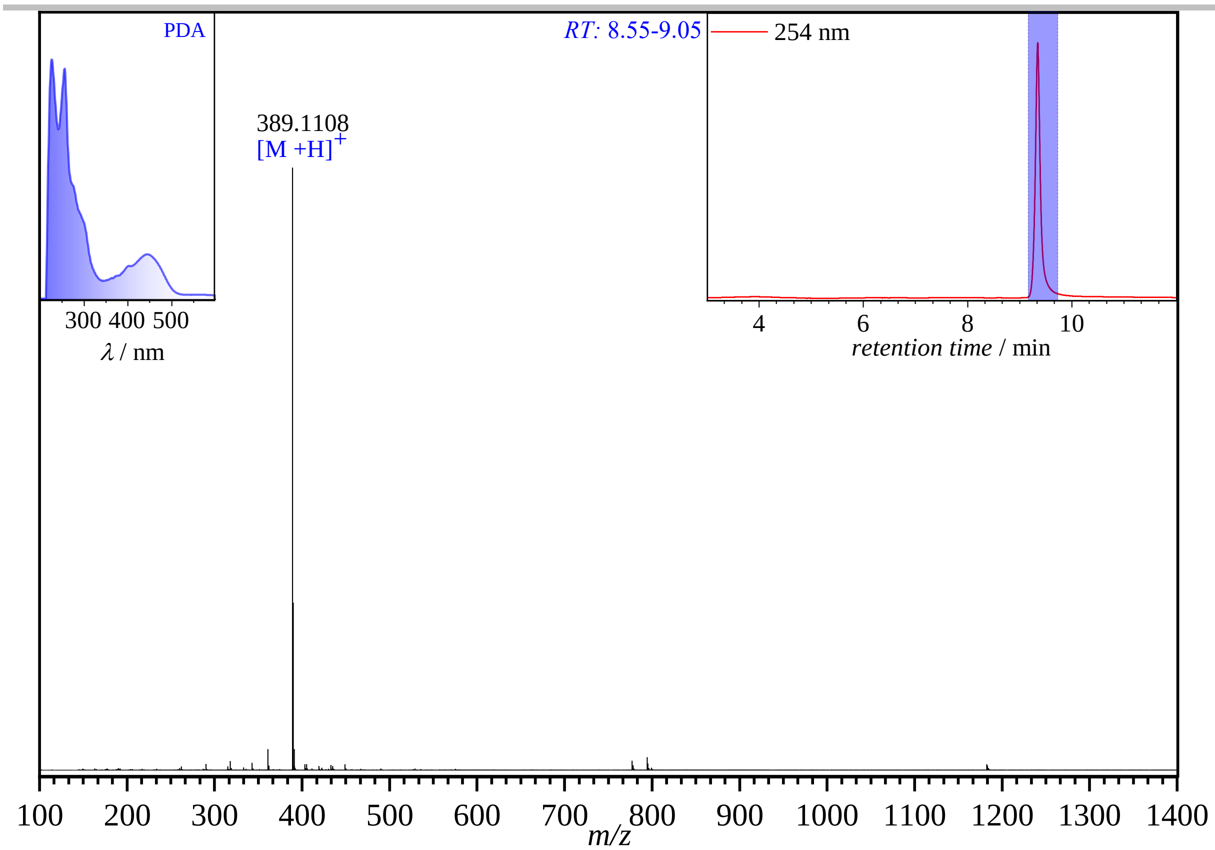

Figure S121 LC-trace (254 nm detector wavelength), accumulated mass-spectra and accumulated PDA spectra of 2,10-diethyl-6-methyl-1H-pyrrolo[3',4':2,3]naphtho[1,8-*ef*]isoindole-1,3,5,9,11(2*H*,10*H*)-pentaone (**11a**).

Table S28 Collation of observed signals in the mass spectrum **Figure S121** and comparison with theoretically expected *m/z* values for the assigned signals.

Symbol	m/z^{exp}	m/z^{theor}	Δ_{ppm}	composition
$[M+H]^+$	389.1108	389.1132	6.17	$C_{22}H_{17}N_2O_5^+$

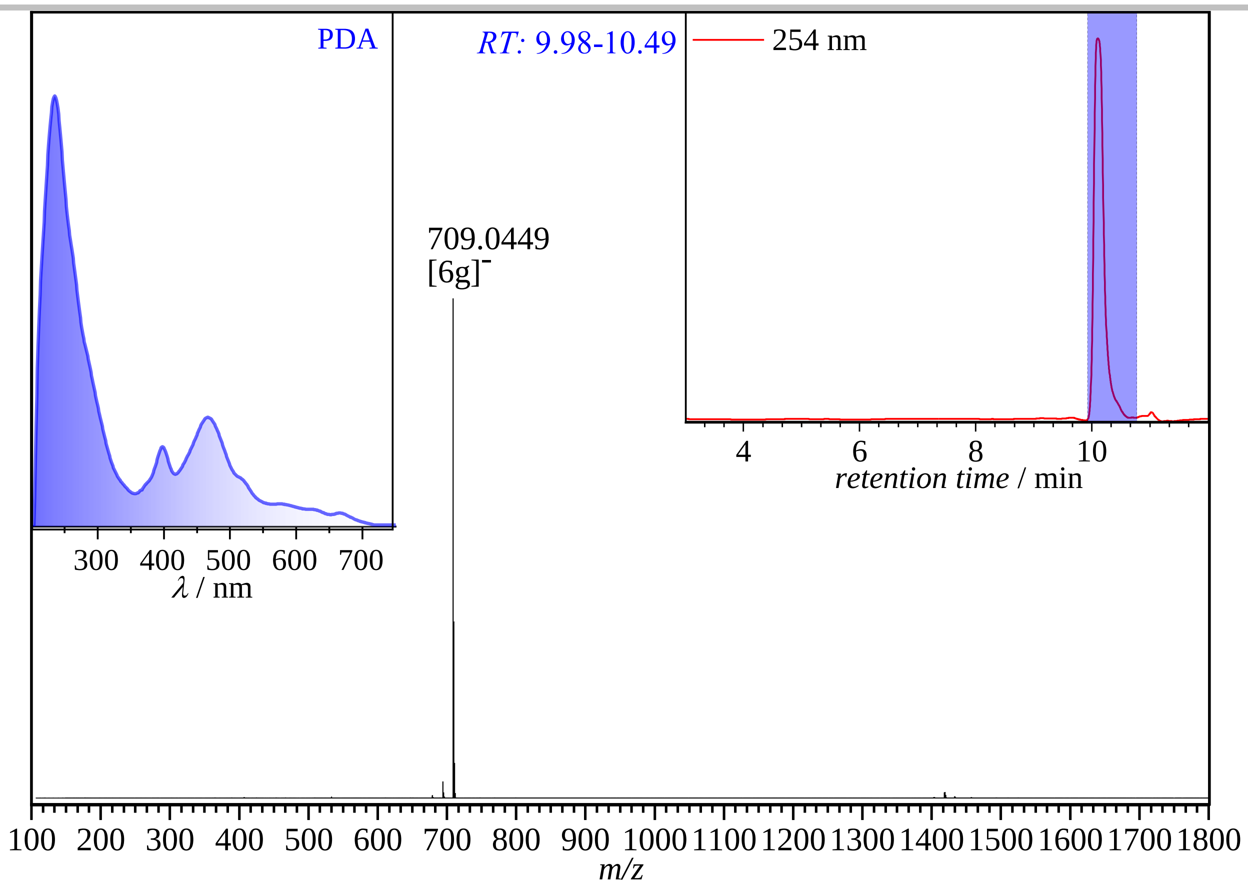

Figure S122 LC-trace (254 nm detector wavelength), accumulated mass-spectra and accumulated PDA spectra of (**6g**).

Table S29 Collation of observed signals in the mass spectrum **Figure S122** and comparison with theoretically expected *m/z* values for the assigned signals.

Symbol	m/z^{exp}	m/z^{theor}	Δ_{ppm}	composition
[M]$^{-}$	709.0449	709.0457	1.13	$C_{32}H_{11}F_{10}N_2O_6^{\bullet-}$

XV. References

[1] CrysAlis Pro Rigaku Oxford Diffraction, **2019**.
[2] G. M. Sheldrick, *Acta Crystallographica a-Foundation and Advances* **2015**, *71*, 3-8.
[3] G. Sheldrick, *Acta Crystallographica Section C* **2015**, *71*, 3-8.
[4] O. V. Dolomanov, L. J. Bourhis, R. J. Gildea, J. A. K. Howard, H. Puschmann, *J. Appl. Crystallogr.* **2009**, *42*, 339-341.
[5] D. Cordischi, M. Occhiuzzi, R. Dragone, *Appl. Magn. Reson.* **1999**, *16*, 427-445.
[6] S. Stoll, A. Schweiger, *Journal of Magnetic Resonance* **2006**, *178*, 42-55.
[7] T. Konakahara, Y. B. Kiran, Y. Okuno, R. Ikeda, N. Sakai, *Tetrahedron Lett.* **2010**, *51*, 2335-2338.
[8] M. Rowe, G. H. Teo, J. Horne, O. Al-Khayat, C. Neto, S. C. Thickett, *Aust. J. Chem.* **2016**, *69*, 725-734.
[9] J. P. Menzel, B. B. Noble, A. Lauer, M. L. Coote, J. P. Blinco, C. Barner-Kowollik, *J. Am. Chem. Soc.* **2017**, *139*, 15812-15820.
[10] M. F. Elsebai, M. Saleem, M. V. Tejesvi, M. Kajula, S. Mattila, M. Mehiri, A. Turpeinen, A. M. Pirttilä, *Nat. Prod. Rep.* **2014**, *31*, 628-645.
[11] B. Häupler, A. Wild, U. S. Schubert, *Adv. Energy Mater.* **2015**, *5*, 1402034.

XVI. Author Contributions

F.F. conceptualized the synthetic approach and executed the synthetic work. S.L.W performed the reaction and fluorescence quantum yield measurements, developed the Matlab® scripts and performed the calculations thereof. A.M. assisted with the interpretation of the EPR spectra. A.B. and J.M. performed the single-crystal XRD measurements and interpretation of the results. J.A.A. and J.P.B. assisted with CV measurements. S.V.K. contributed during the project conceptualization phase. T.W., J.P.B., C.B.-K. motivated the project and secured concept funding for *ortho*-quinodimethane driven photochemistry. All authors contributed to manuscript drafting and editing.
